# Supplementary material for: Conserved and specialized features of thalamocortical wiring revealed by single-cell projection mapping in mouse and marmoset
Source: bioRxiv. 2026 Jul 8:2026.07.07.736957. Preprint. [Version 1] doi: 10.64898/2026.07.07.736957 (PMC13371098; doi:10.64898/2026.07.07.736957)
Supplement: Supplement 6 — Supplementary File 1: zip file containing dissection slice images for the BARseq experiments [file media-6.zip › Supplementary File 1/Marmoset Dissections/Hazelnut_dissection_plan_annotated.pdf]

HEMISPHERE

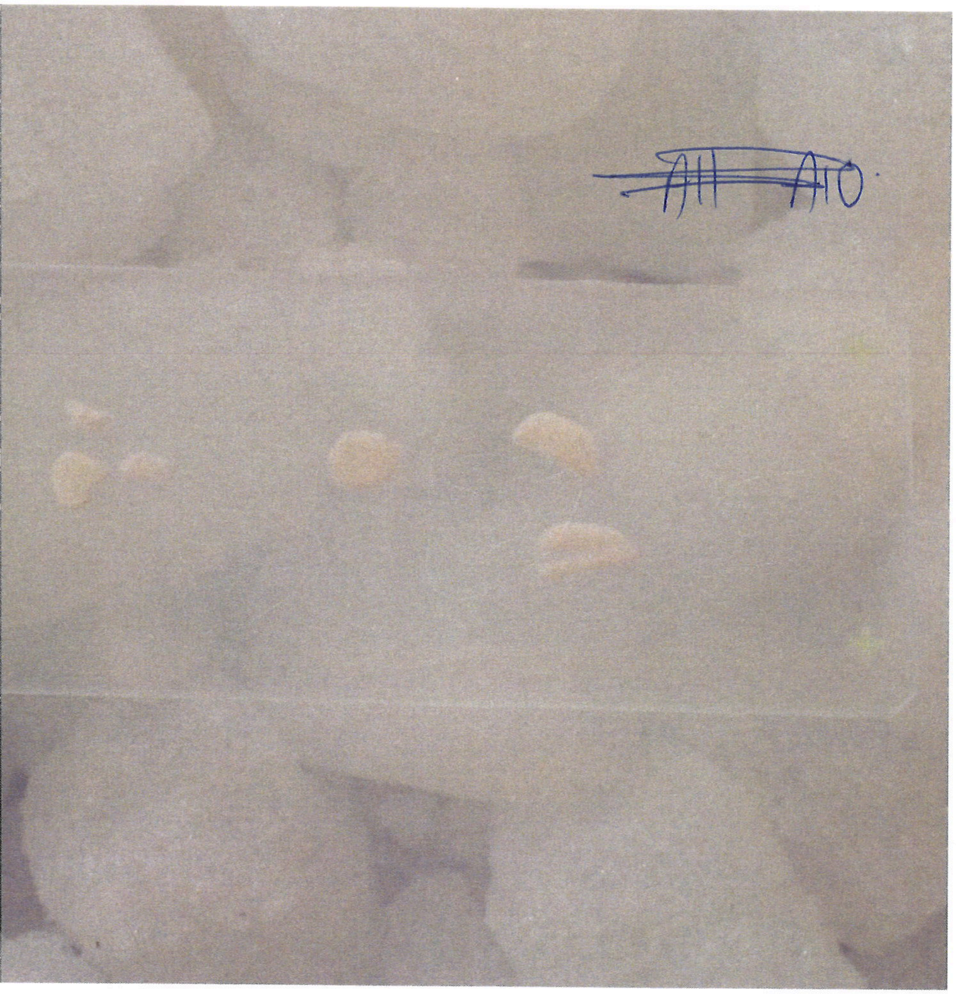

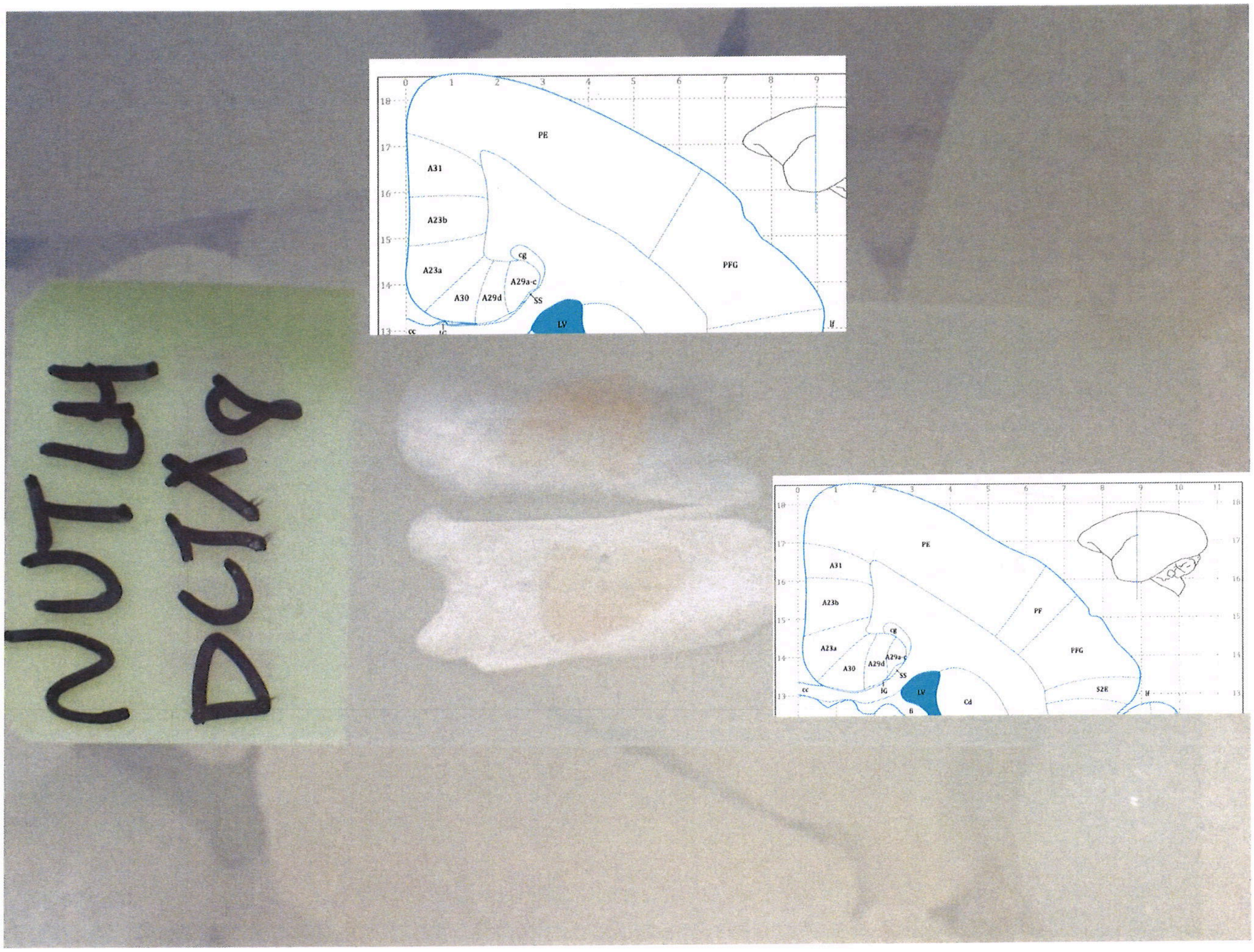
PE
A31
A23b
A23a
A30
A29d
A29a-c
cg
SS
LV
PF
PFG
S2E
Cd
IG
cc

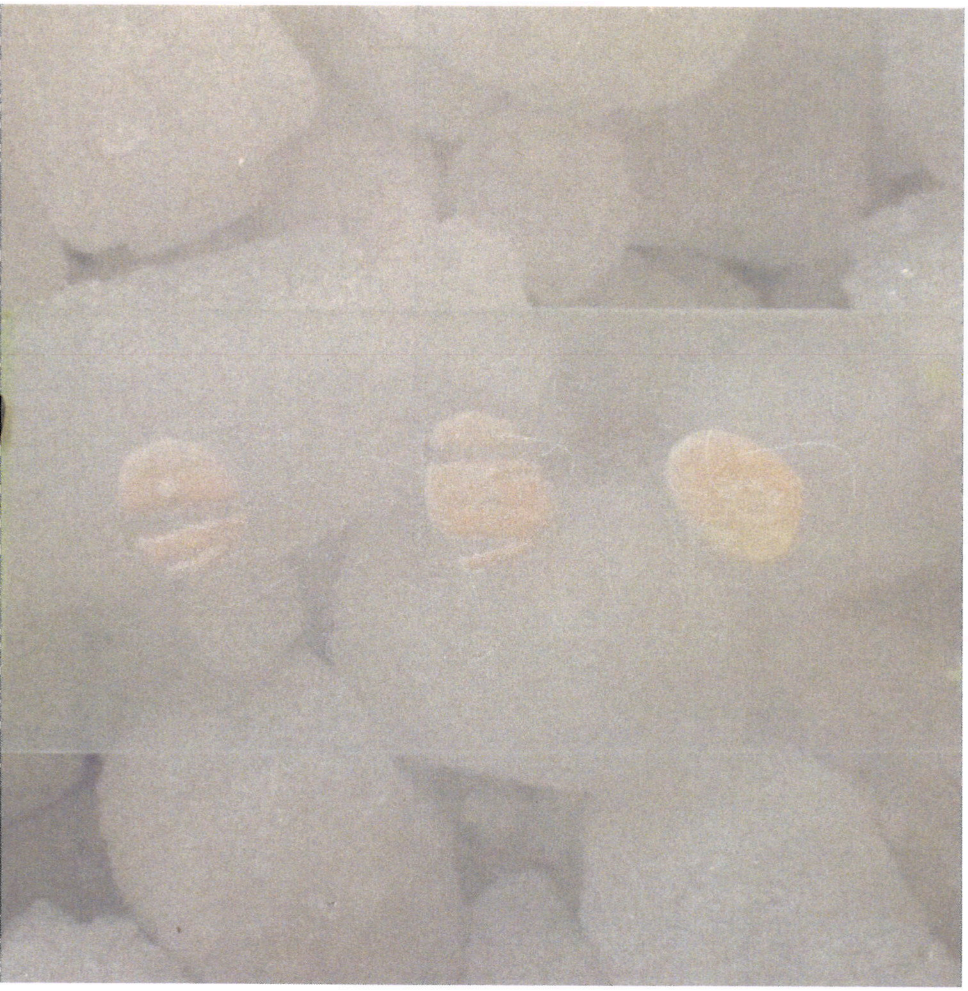

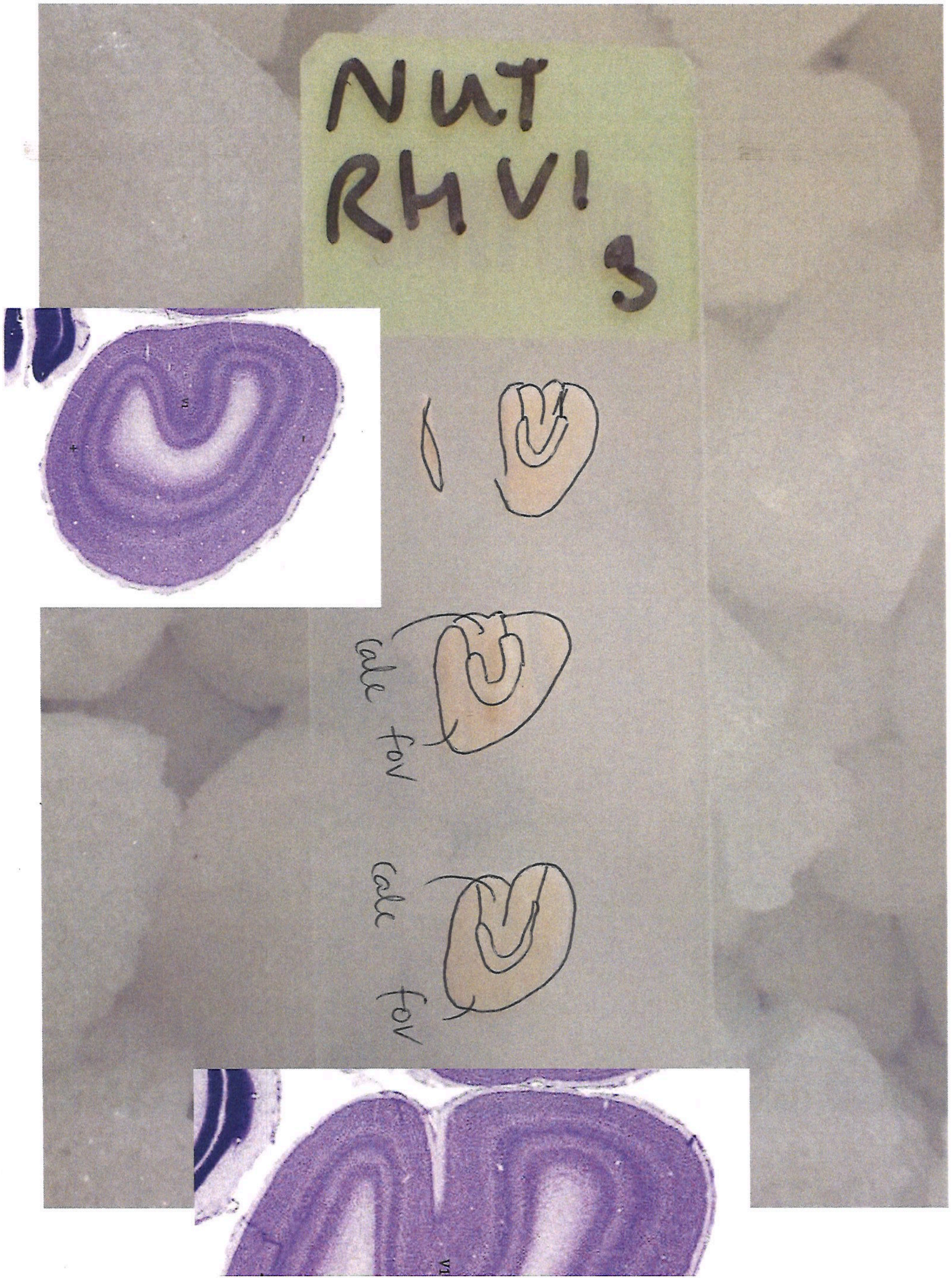
NUT
RHV1
3
calc
fov
calc
fov

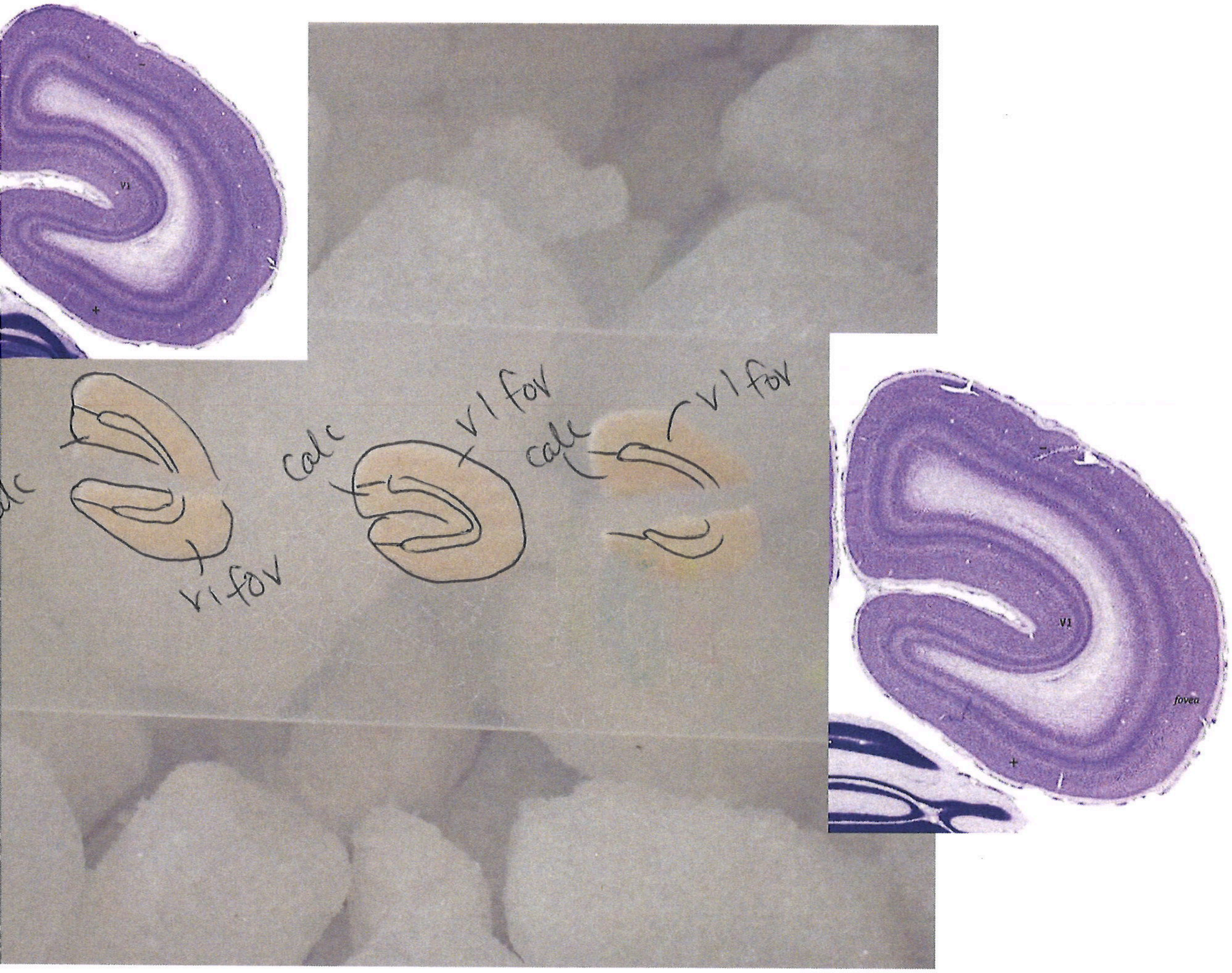
V1
v1 fov
calc
v1 fov
calc
v1 fov
V1
fovea

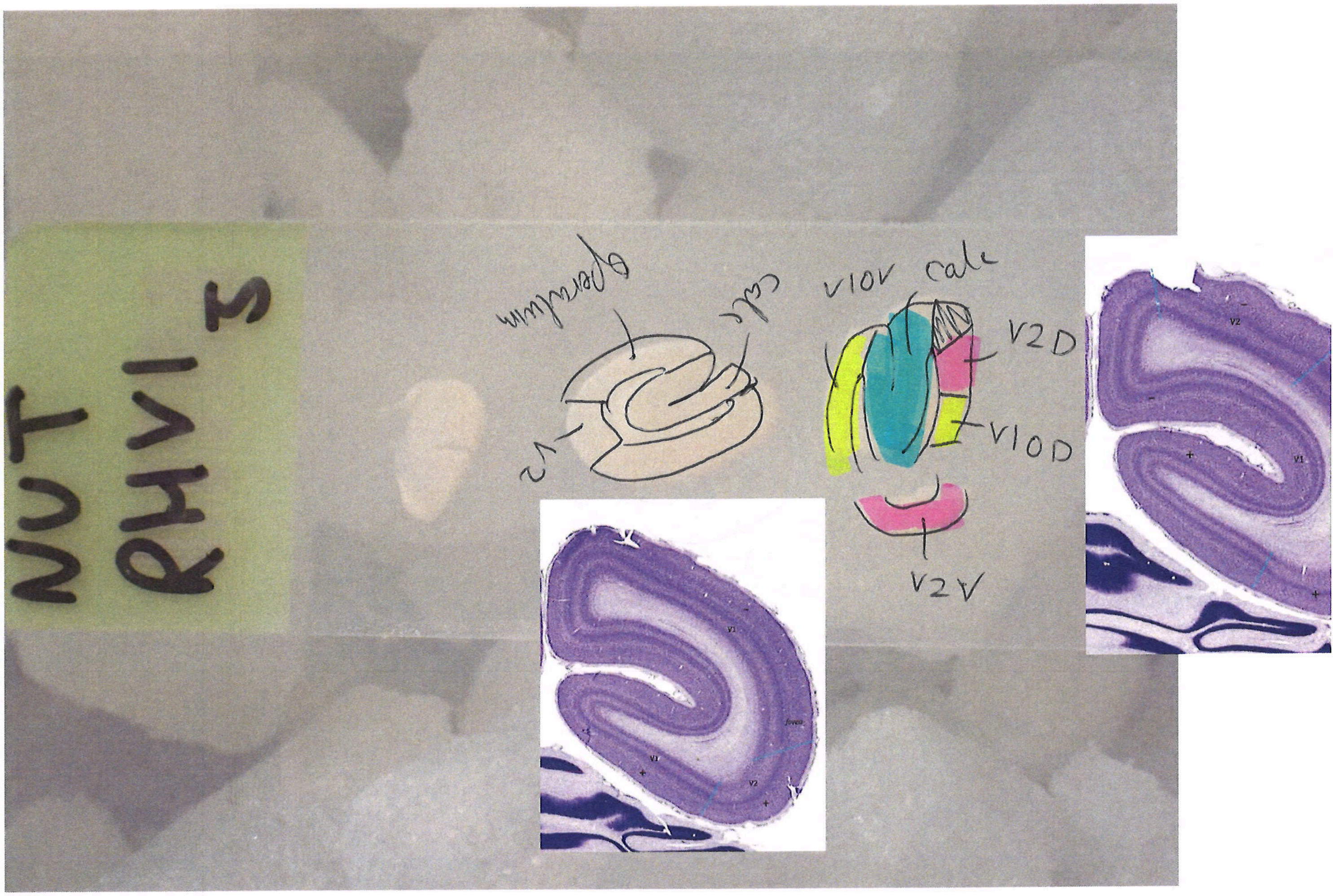
NUT
RHV1
3
operculum
calc
V2
V1OV
calc
V2D
V1OD
V2V

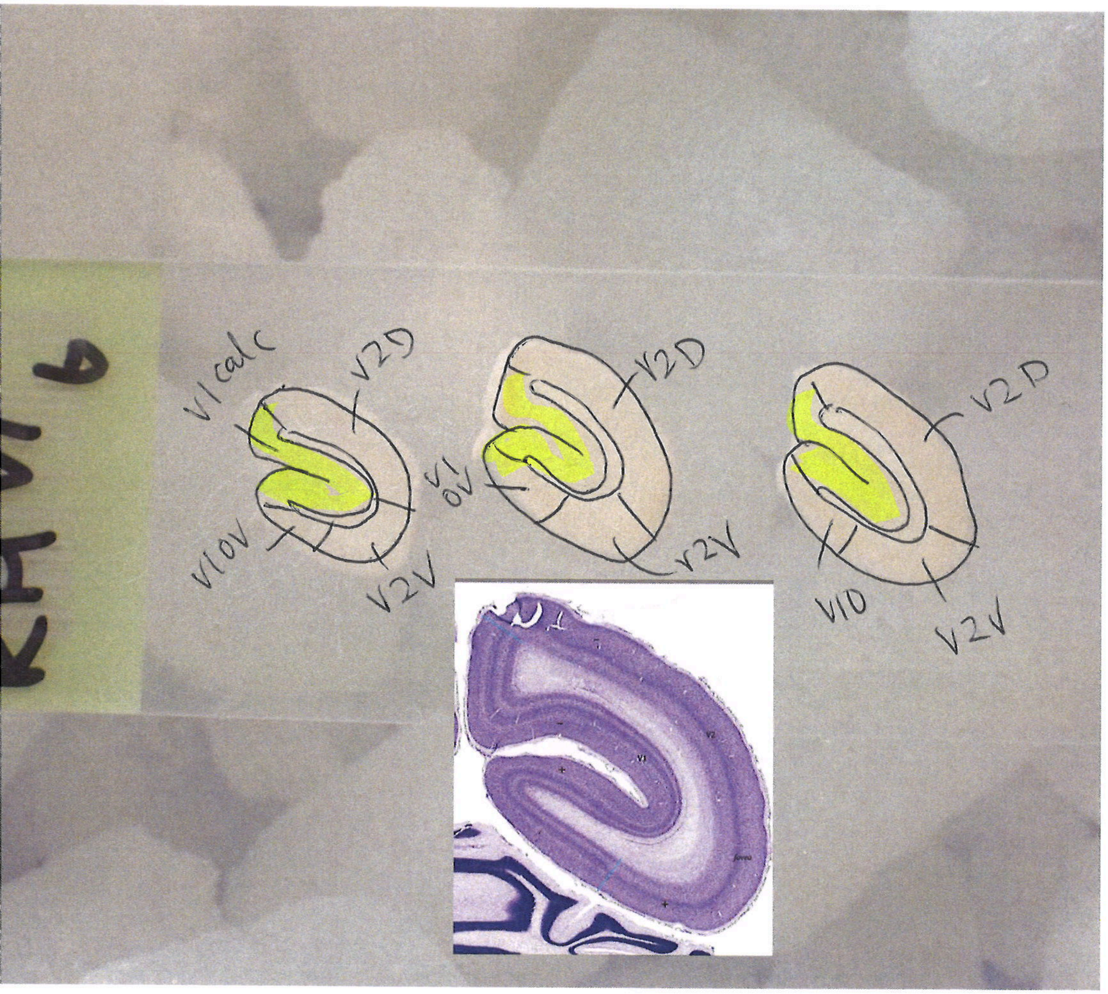
V1 calc
V2D
V1 OV
V2V
V1 OV
V2D
V2V
V2D
V1D
V2V

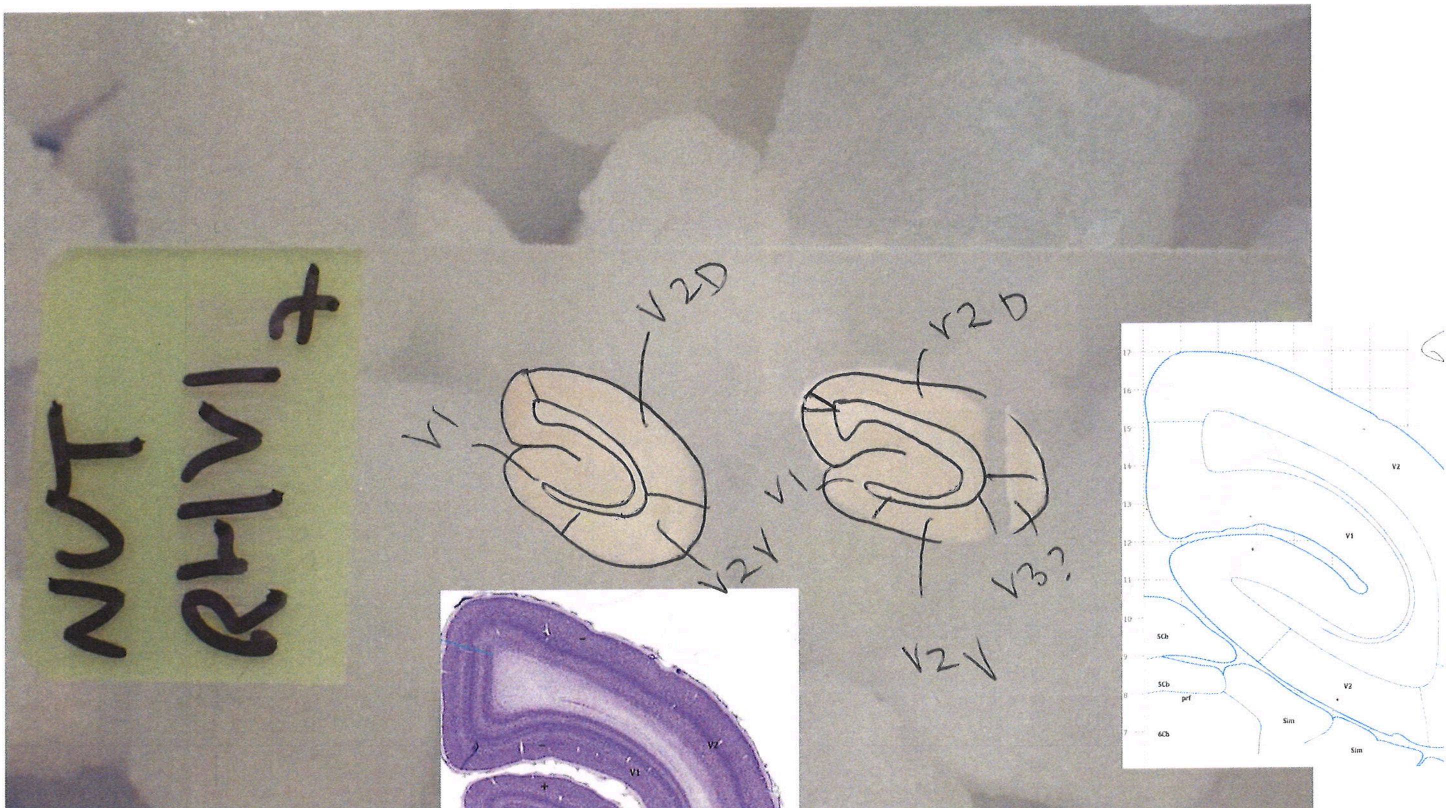

NUT
RH1V1+
V2D
V1
V2V
V2D
V1
V3??
V2V
V1
V2
fovea
V2
V1
V2
SCb
SCb
prf
6Cb
Sim
Sim

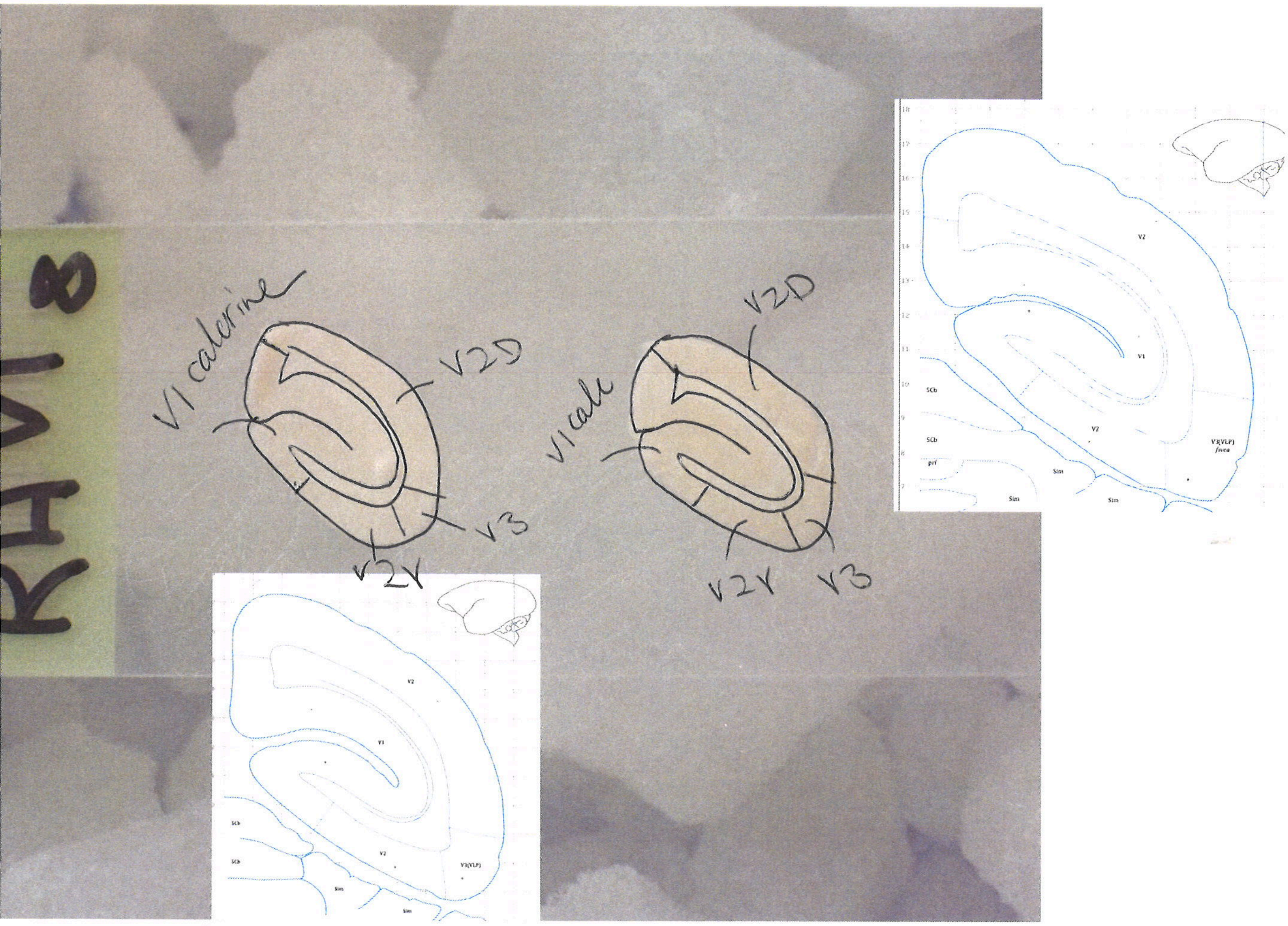
V1 calcarine
V2D
V3
V2V
V1 calc
V2D
V2V
V3

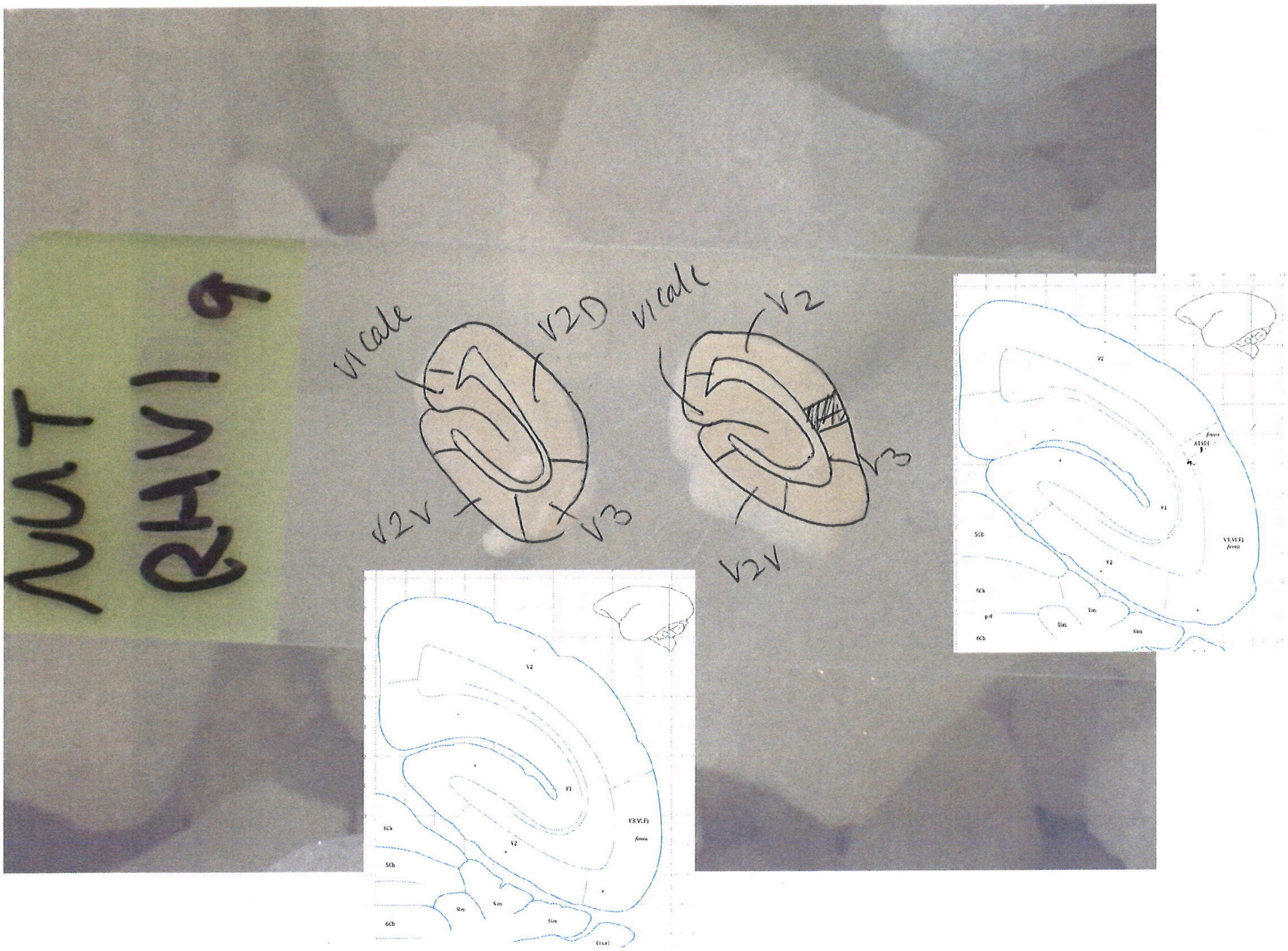
NUT
RHV1 9
V1 calc
V2D
V2V
V3
V1 calc
V2
V3
V2V

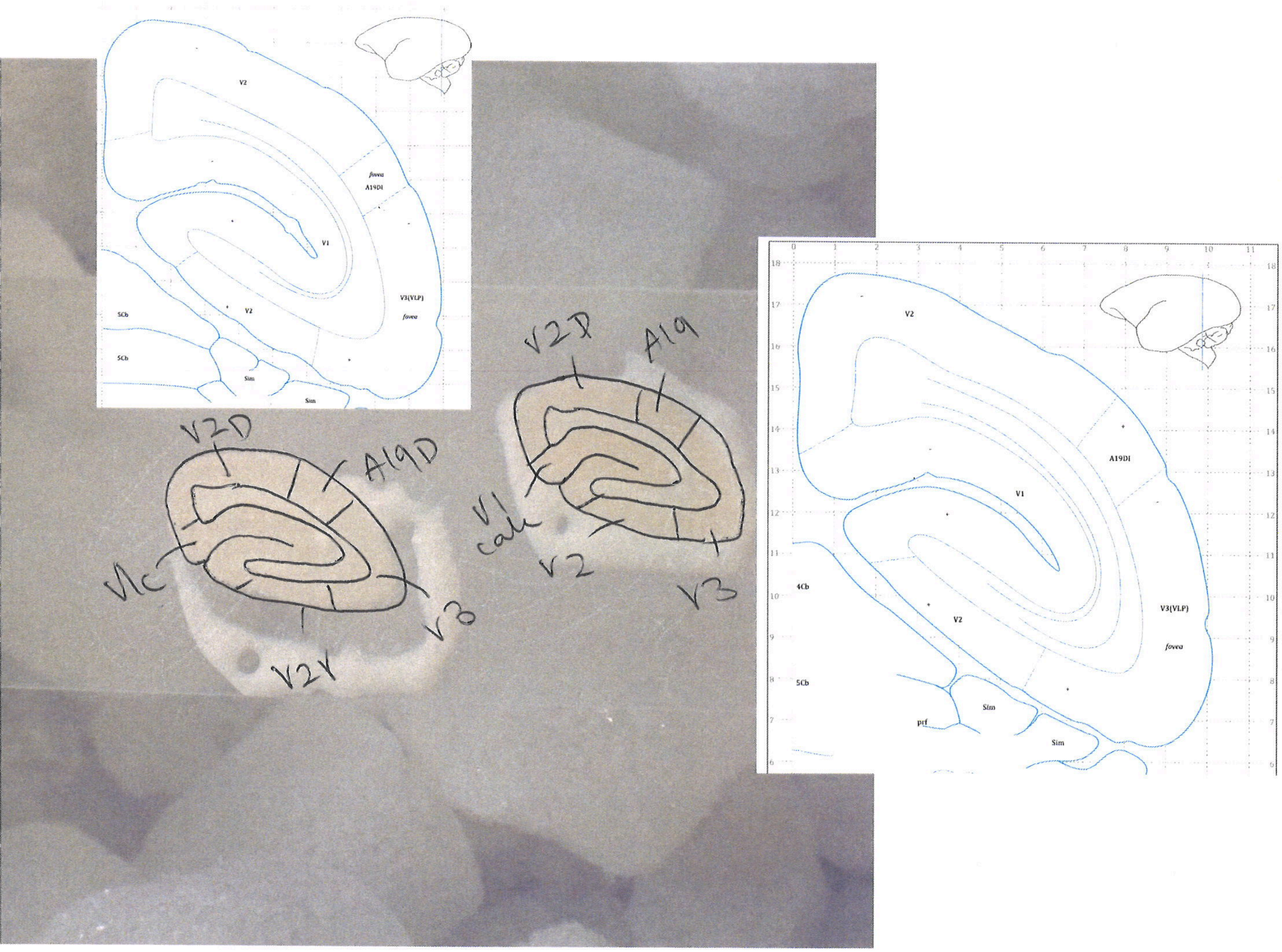

V2
fovea
A19DI
V1
5Cb
V2
V3(VLP)
fovea
5Cb
Sim
Sim
V2D
A19D
V1c
V3
V2V
V2D
A19
V1
calc
V2
V3
V2
A19DI
V1
4Cb
V2
V3(VLP)
fovea
5Cb
Sim
prf
Sim

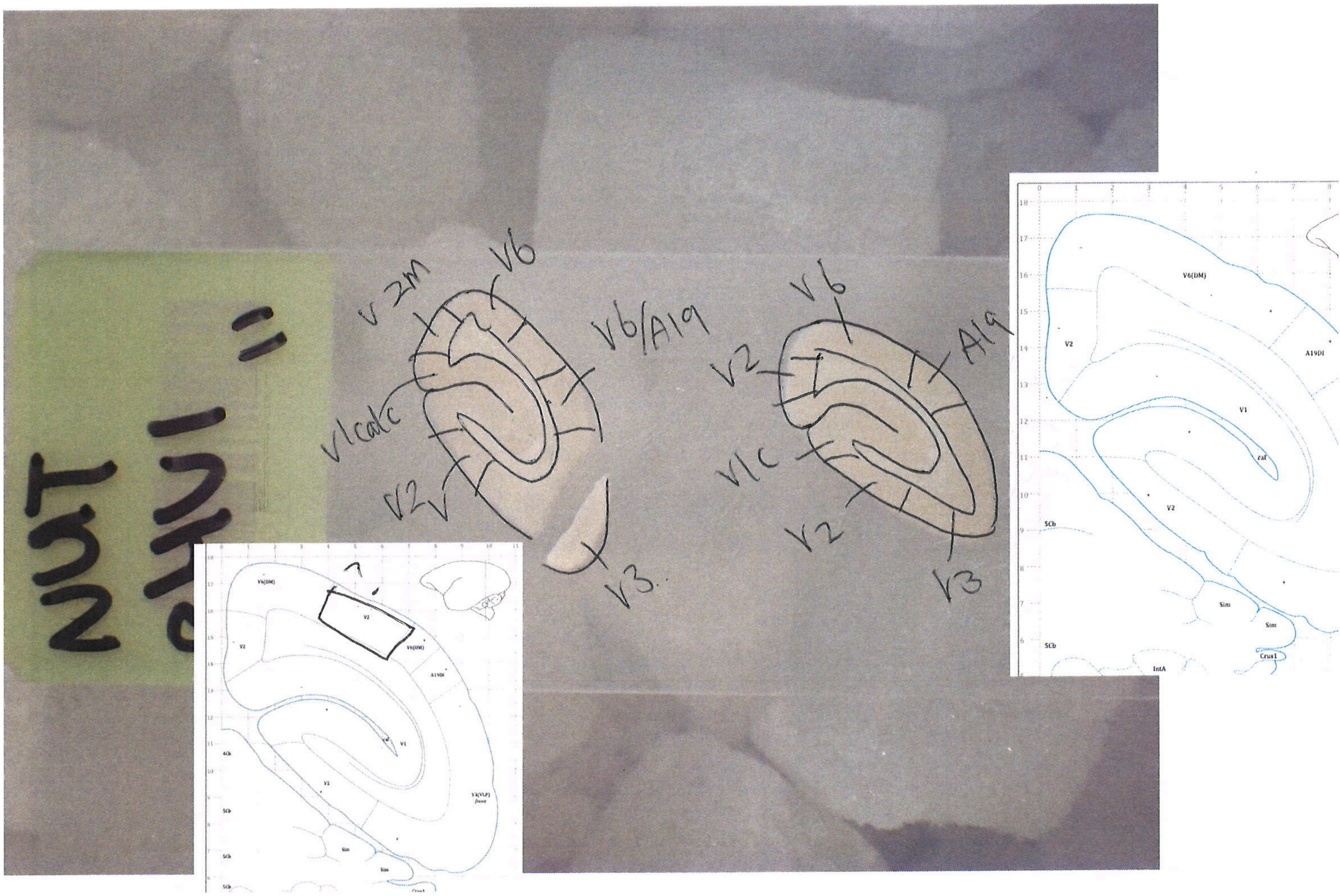
NUT
V2M
V6
V6/A19
V1calc
V2
V3.
V6
V2
A19
V1C
V2
V3
V6(DM)
V2
A19Dl
V1
cal
5Cb
Sim
IntA
Crus1
4Cb
V6(VLP)

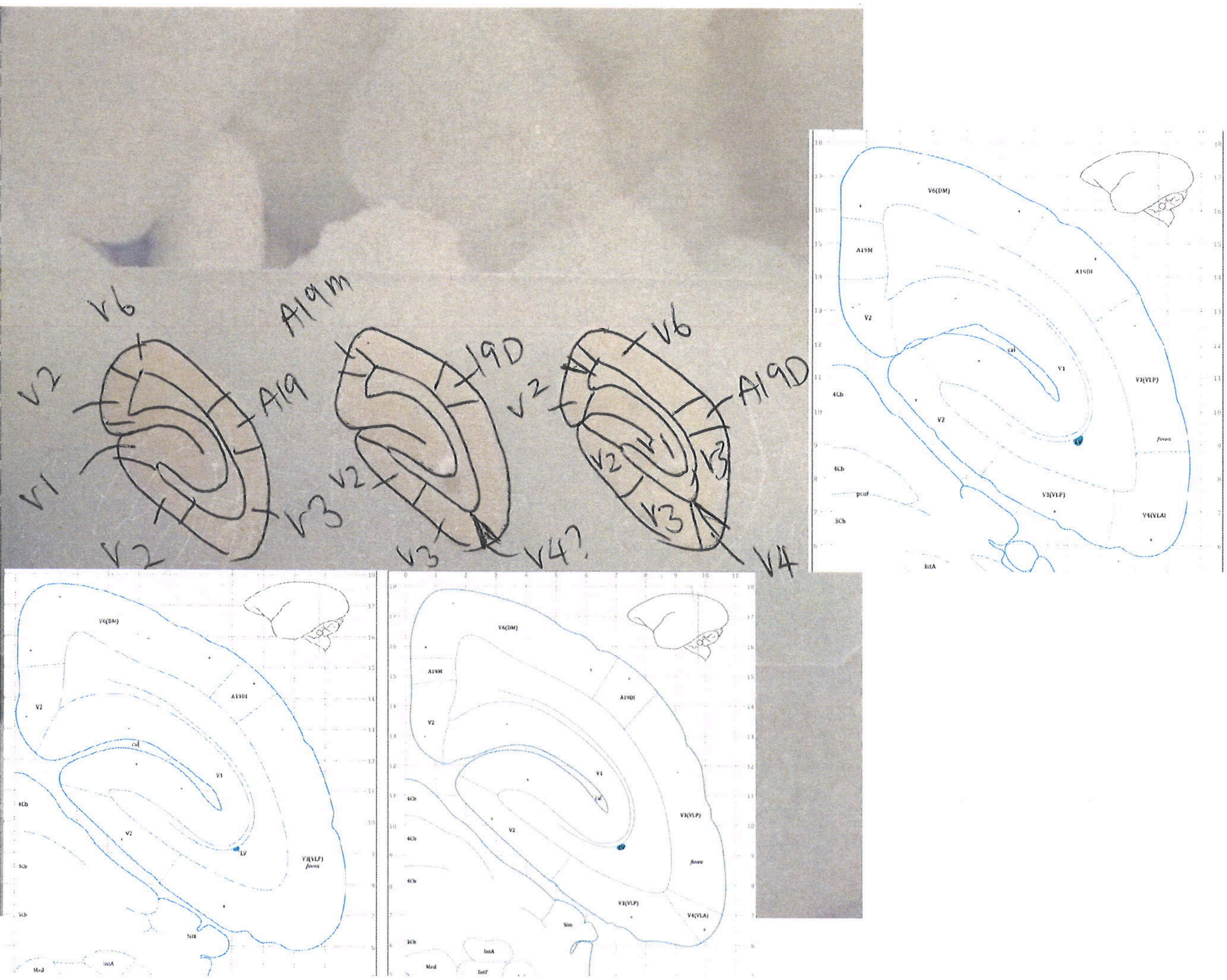
V6
A19m
V2
A19
19D
V6
V2
A19D
V1
V2
V1
V3
V2
V3
V3
V4?
V3
V4
V6(DM)
A19M
A19DI
V2
cal
V1
V3(VLP)
fovea
V4(VLA)
LV
4Cb
pcul
3Cb
IntA
Sim
Med

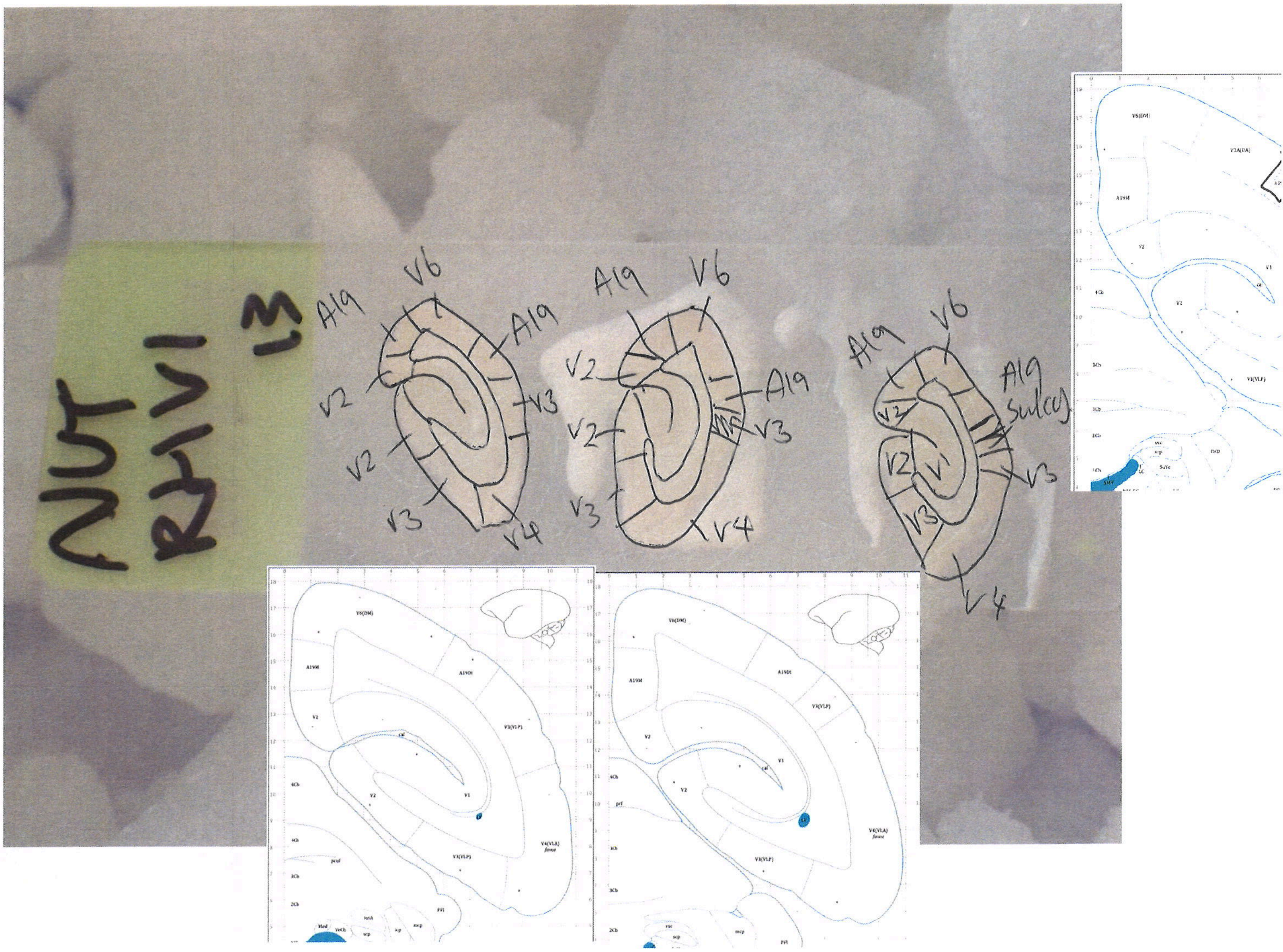

NUT
RHV1
13
A19
V6
V2
V3
V4
A19 sulc(cc)
V6(DM)
A19M
A19DI
V3(VLP)
V4(VLA) fovea
V1
V2
cal

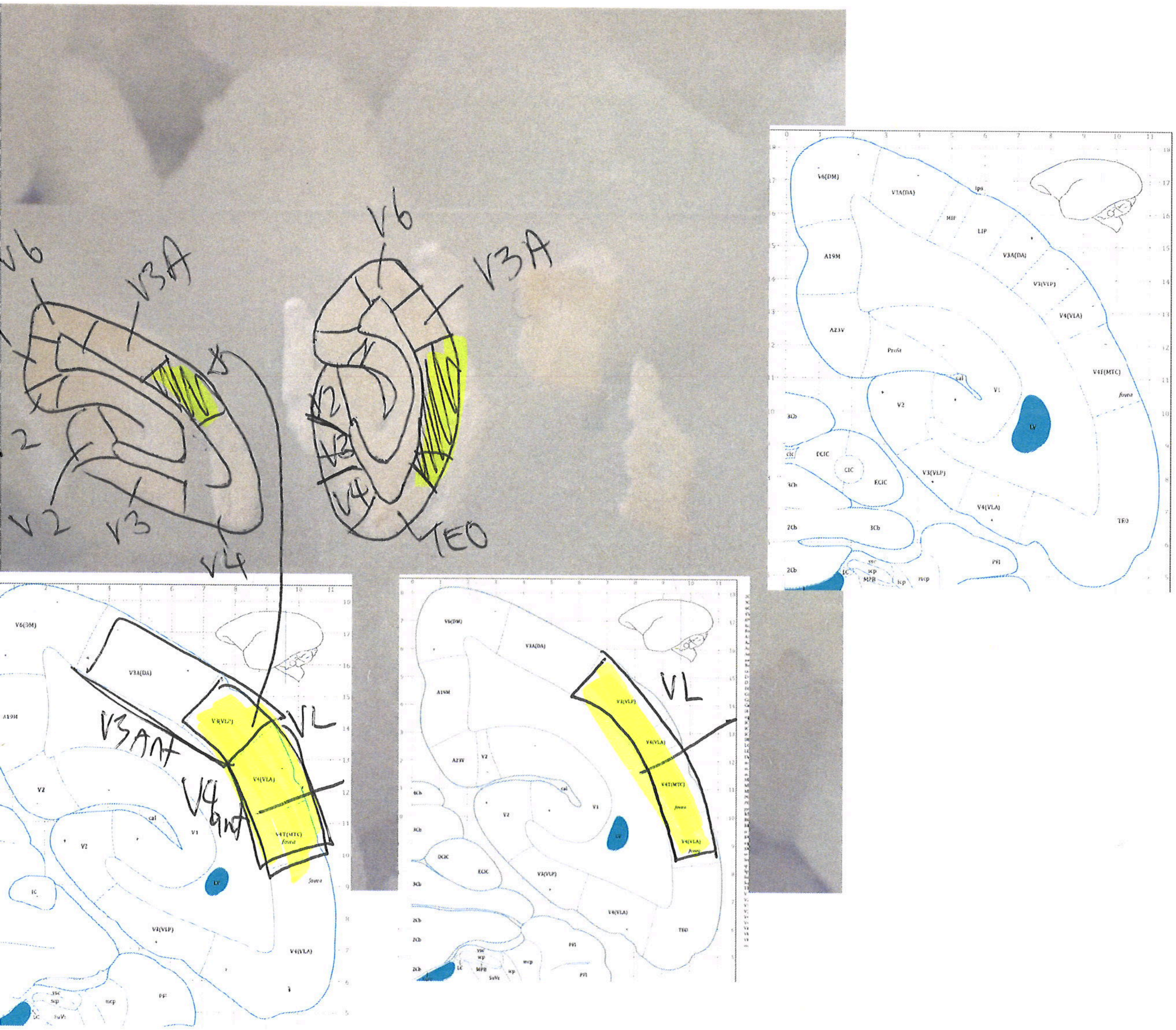

V6
V3A
V2
V3
V4
TEO
V3Ant
V4ant
VL
V6(DM)
V3A(DA)
V3(VLP)
V4(VLA)
V4T(MTC)
fovea
A19M
A23V
V1
V2
LV
TEO

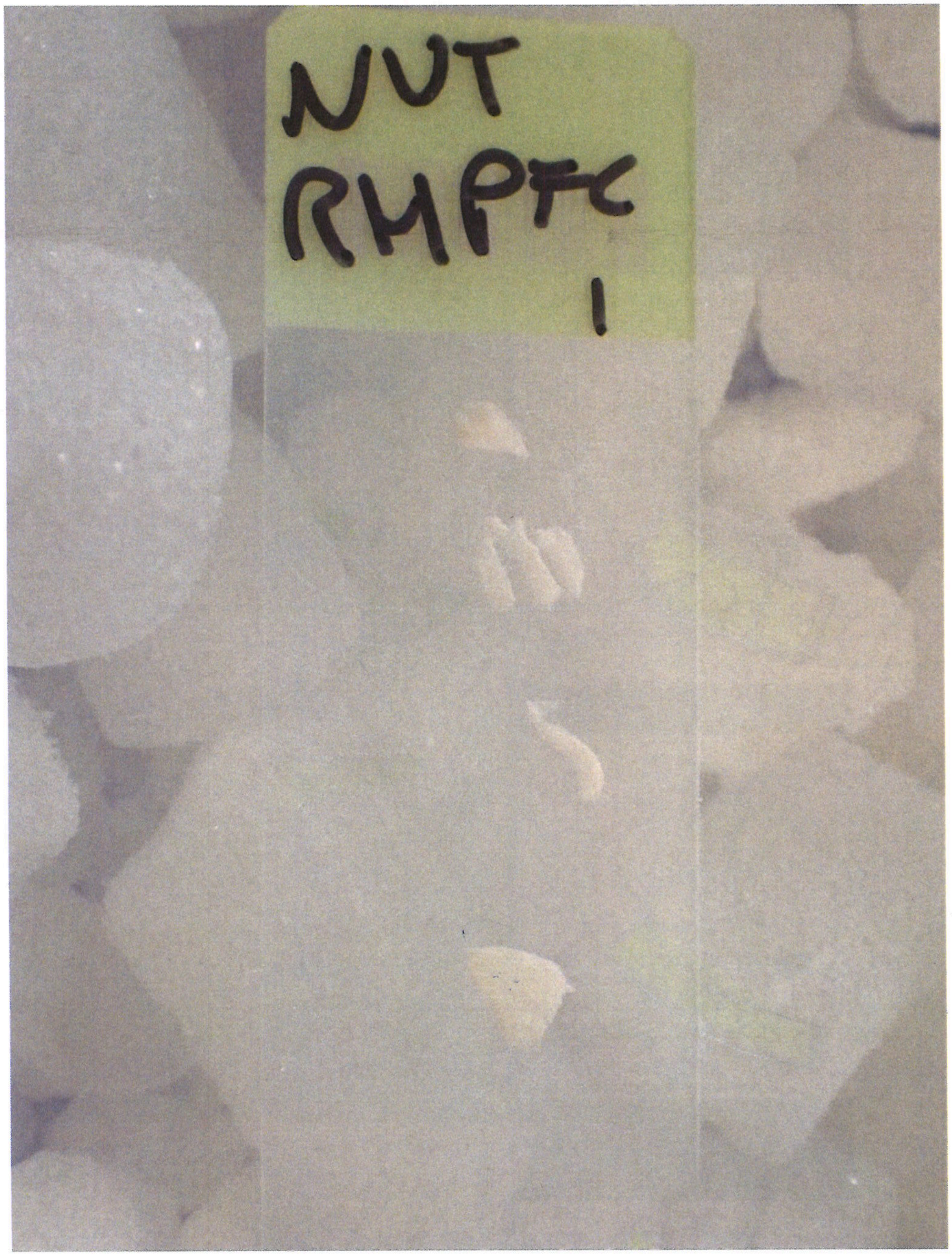
NUT
RMPFC
1

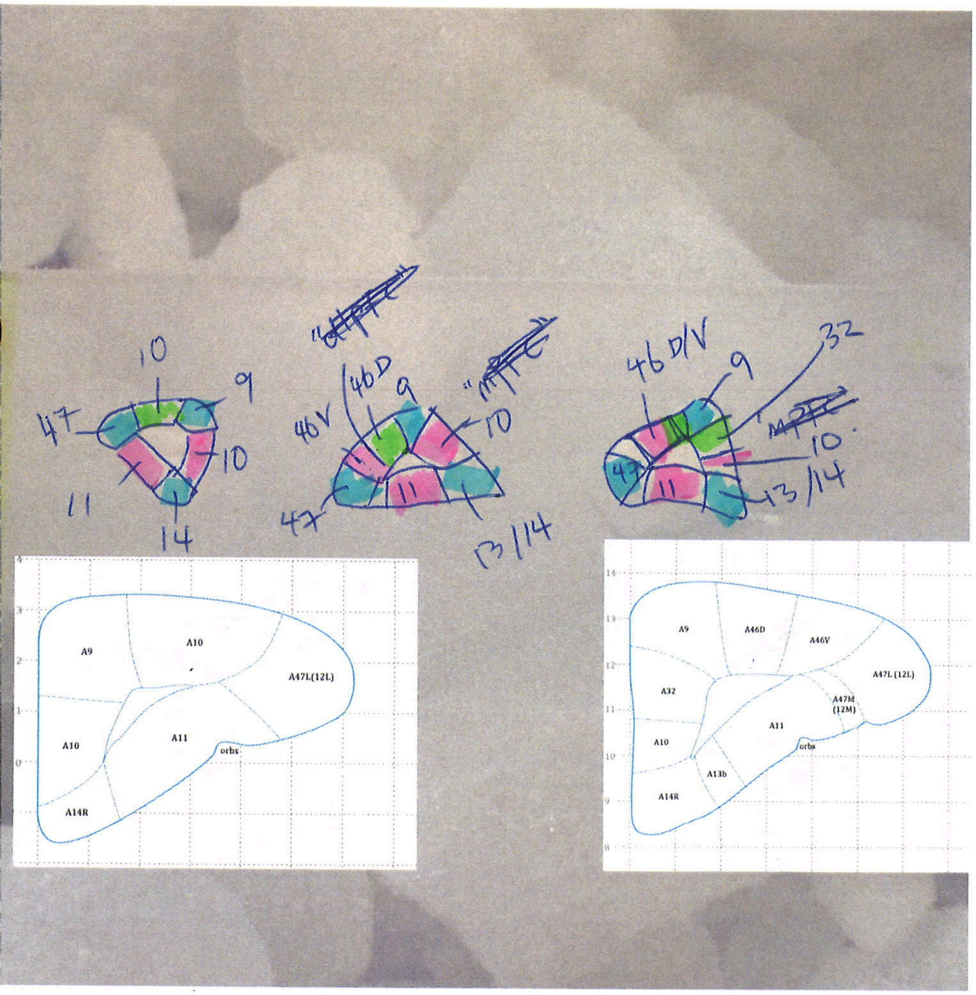
10
9
47
10
11
14
46V
46D
9
10
47
11
13/14
46 D/V
9
32
10
47
11
13/14
A9
A10
A47L(12L)
A10
A11
orbs
A14R
A9
A46D
A46V
A47L (12L)
A32
A47M
(12M)
A11
orbs
A10
A13b
A14R

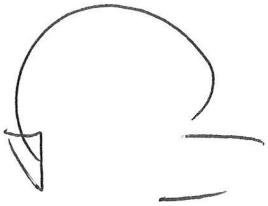

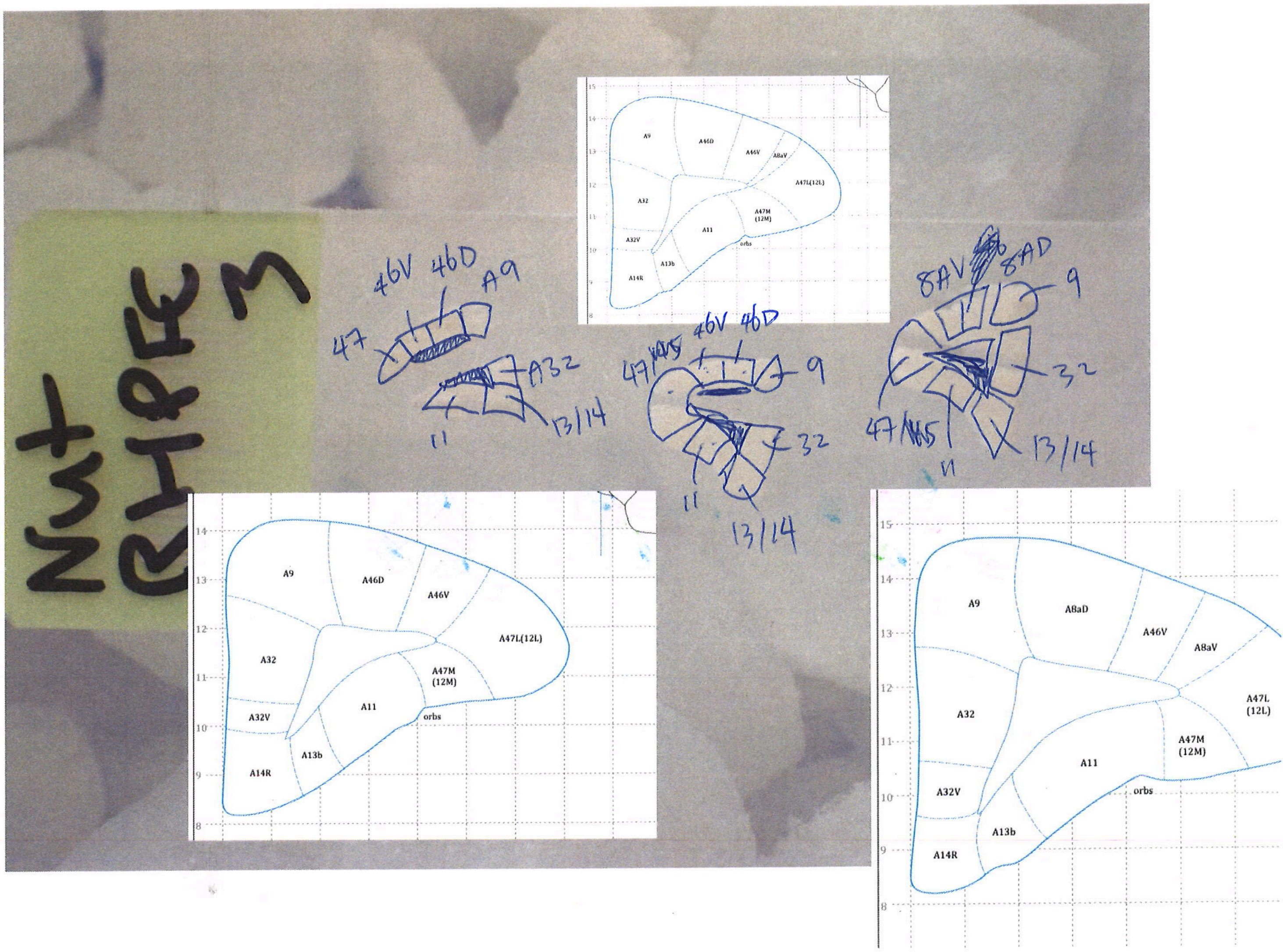
Nut RHPFC 3
A9
A46D
A46V
A8aV
A8aD
A47L(12L)
A47L
(12L)
A32
A47M
(12M)
A11
A32V
orbs
A14R
A13b
46V 46D A9
47
A32
11
13/14
47/45
9
32
8AV
8AD

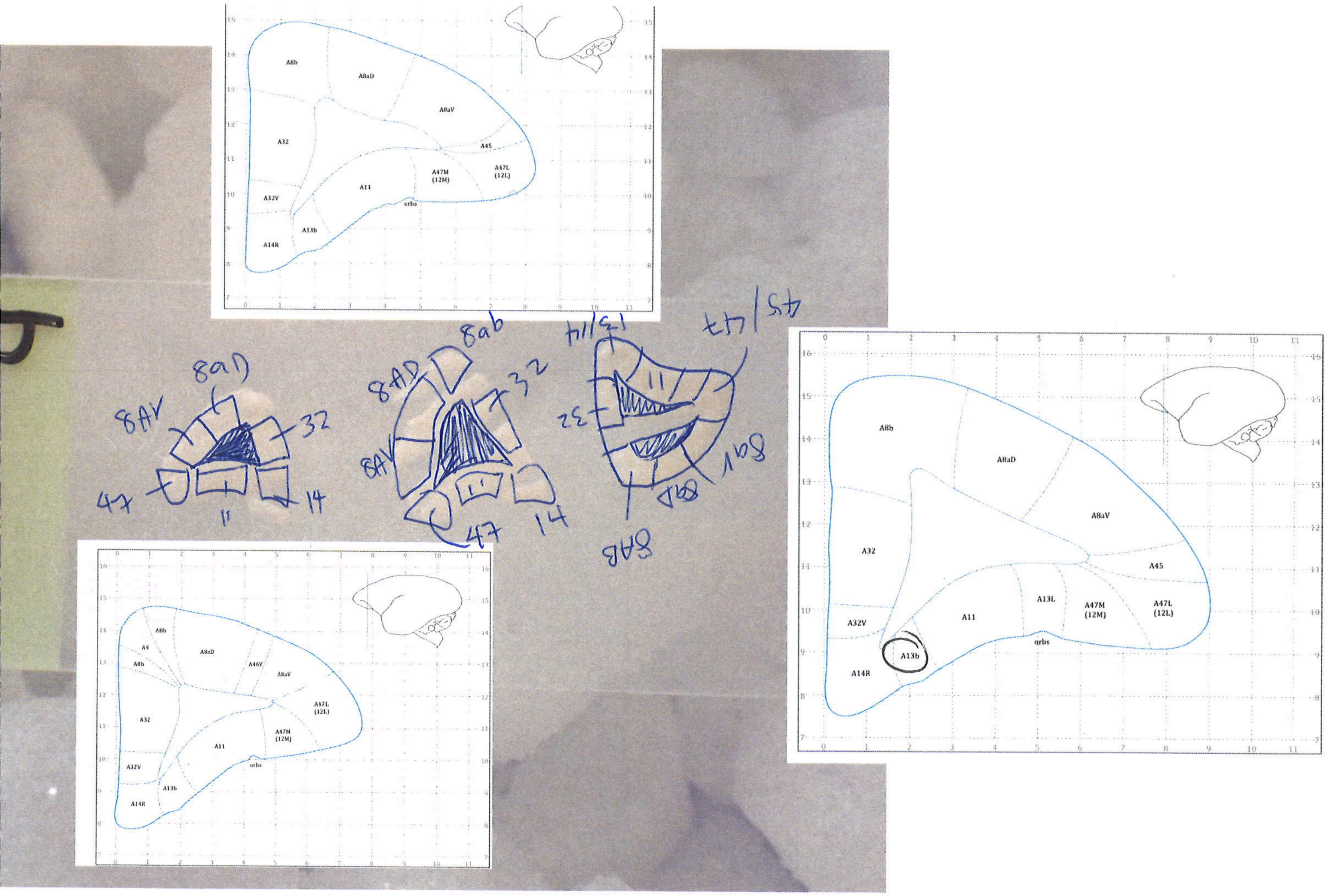
A8b
A8aD
A8aV
A45
A32
A47M (12M)
A47L (12L)
A11
A32V
orbs
A13b
A14R
8aD
8AV
32
47
11
14
8ab
8AD
A46V
A13L

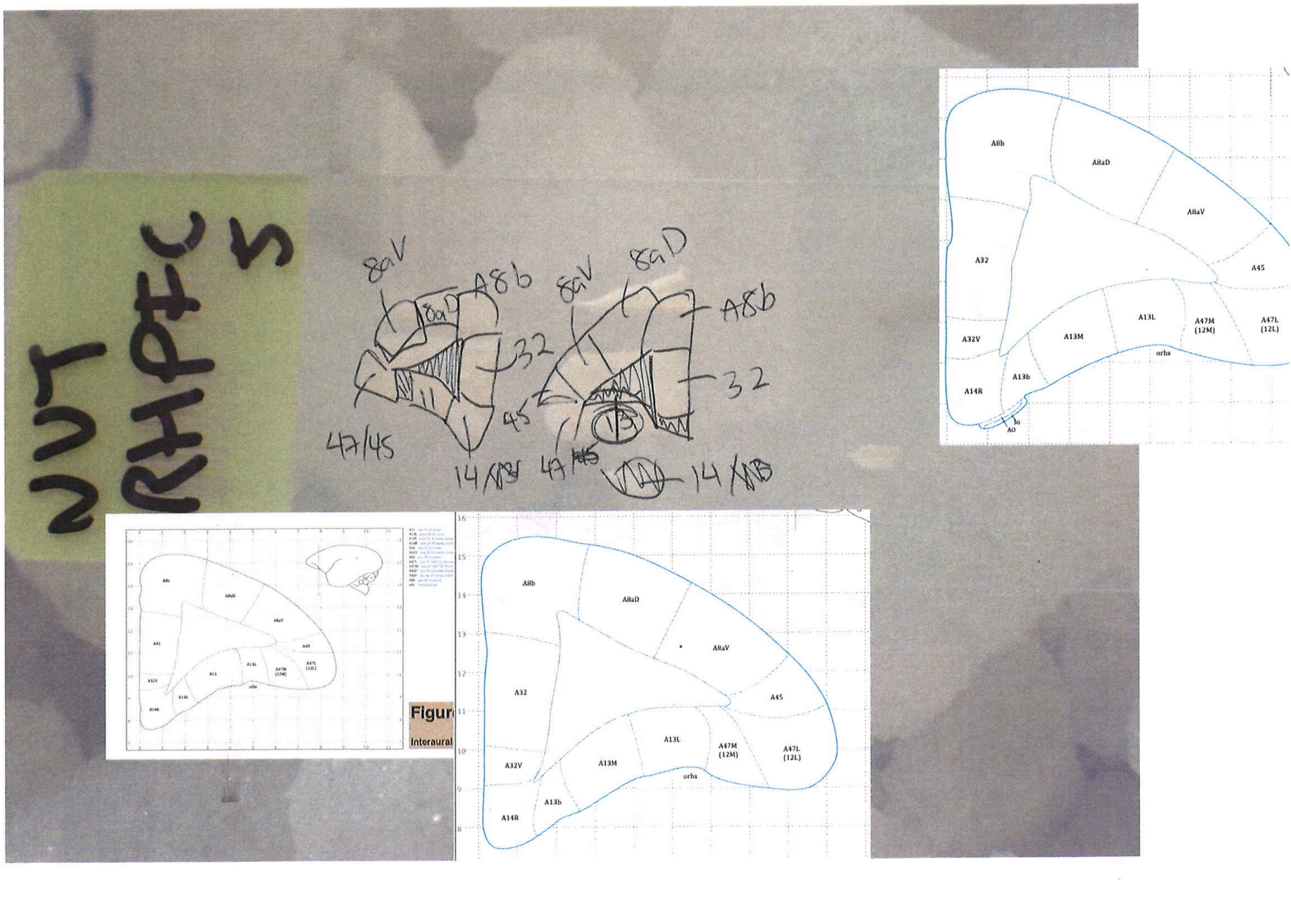
NUT
RHPFC
5
8aV
8aD
A8b
32
47/45
45
13
14/A8
A8b
A8aD
A8aV
A32
A45
A32V
A13M
A13L
A47M
(12M)
A47L
(12L)
orbs
A13b
A14R
A11
Figur
Interaural

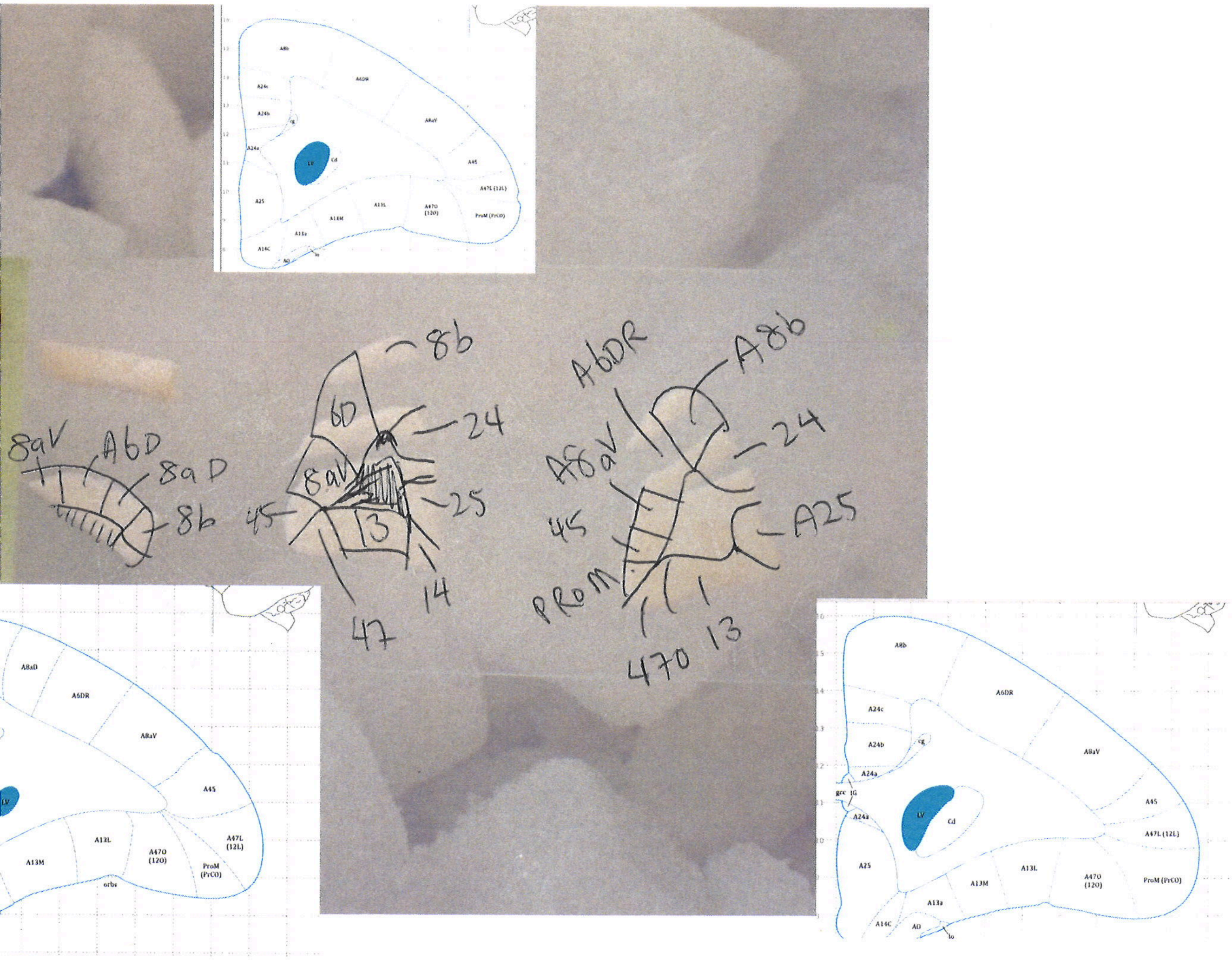
8aV
A6D
8aD
8b
45
6D
8aV
13
8b
24
25
14
47
A6DR
A8b
A8aV
24
45
A25
PROM
470
13
A8b
A6DR
A24c
A24b
cg
A24a
LV
Cd
A8aV
A45
A47L (12L)
A25
A13M
A13L
A47O (12O)
ProM (PrCO)
A13a
A14C
AO
A8aD
orbs

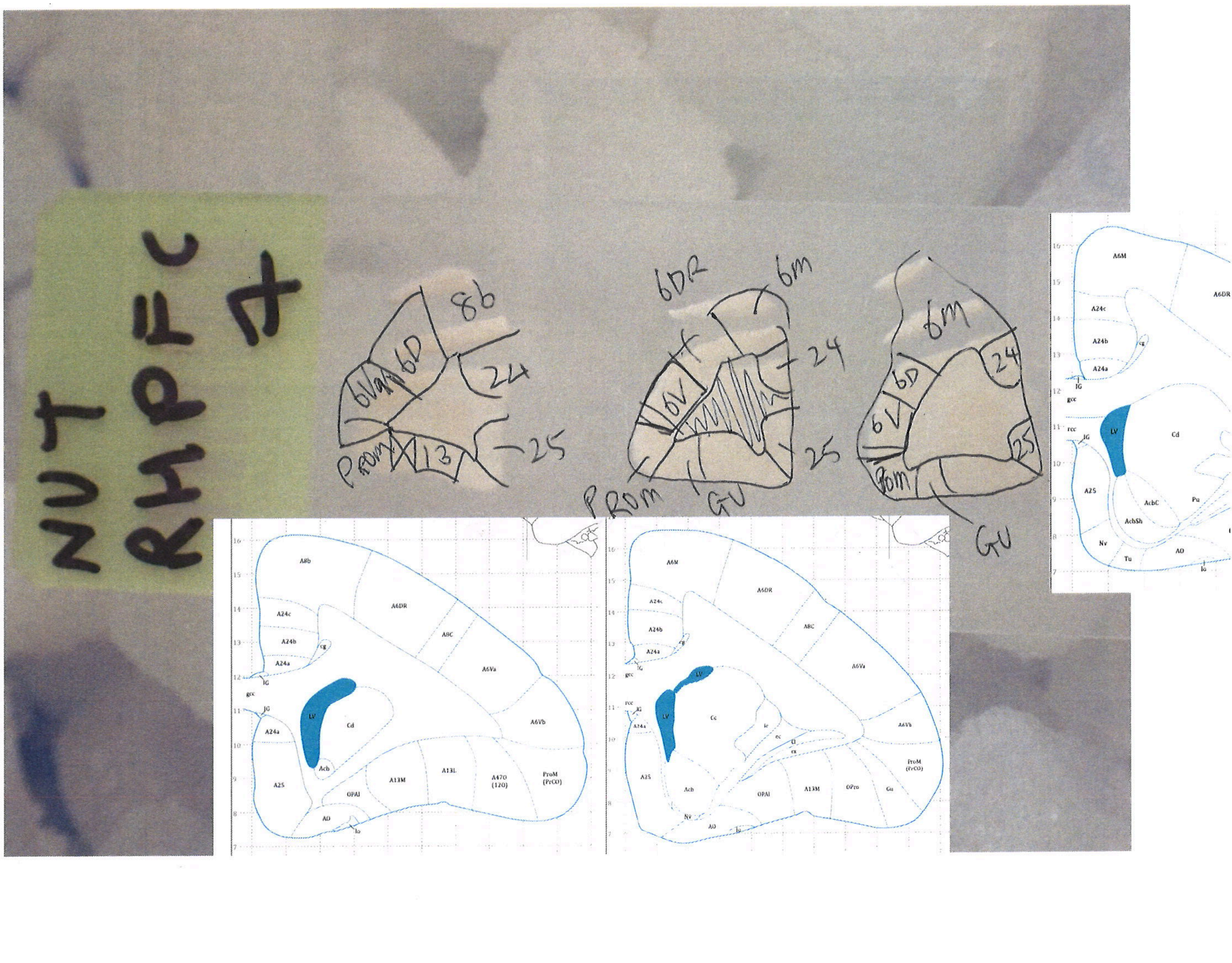

NUT
RHPFC
7
8b
6D
6Va
24
PROM
13
25
6DR
6m
6V
24
25
PROM
GU
6m
6D
24
6V
25
Pom
GU

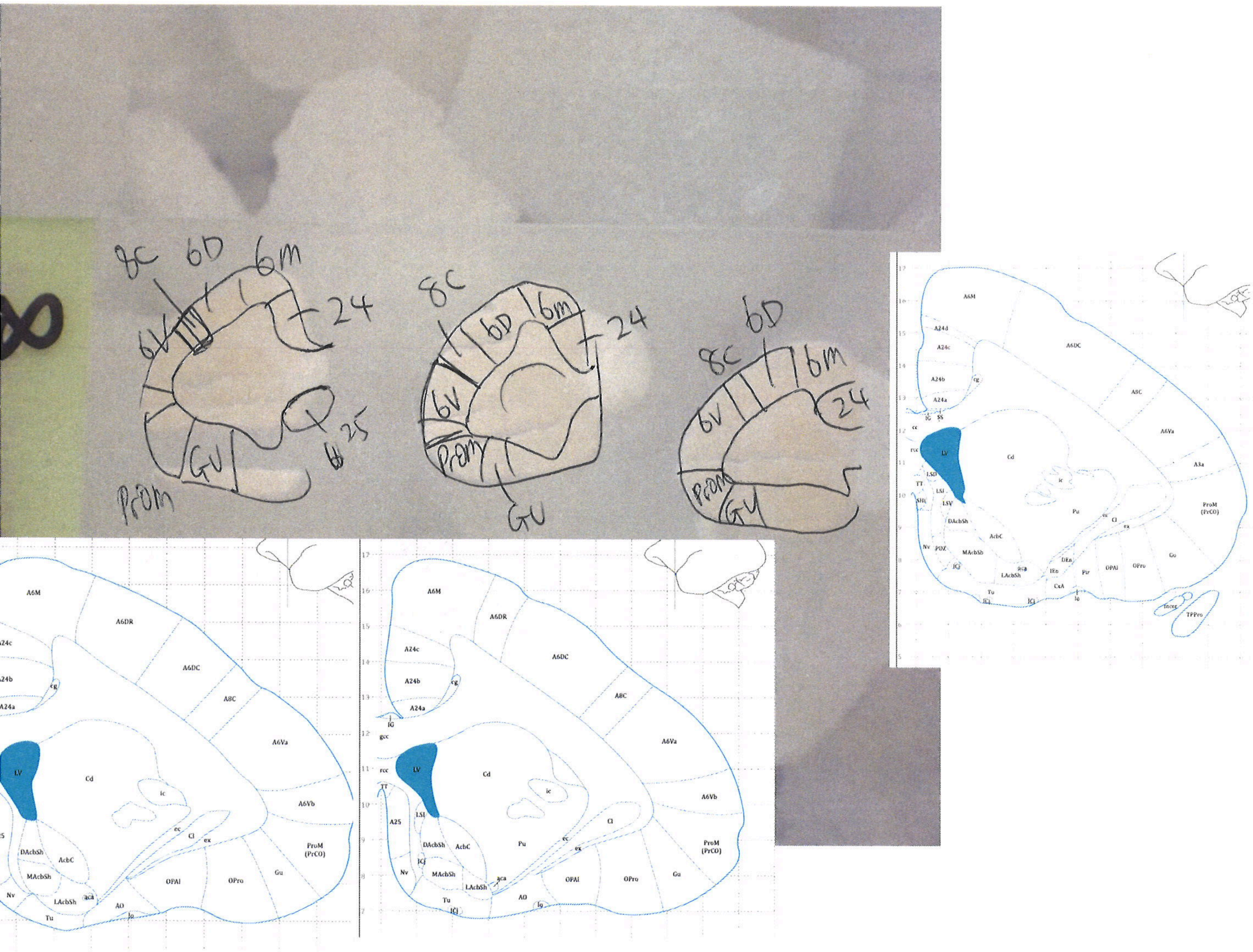
8C
6D
6m
24
6V
GU
ProM
25
ProM
A6M
A6DR
A6DC
A8C
A6Va
A6Vb
A24c
A24b
A24a
cg
IG
gcc
rcc
TT
LV
Cd
ic
LSI
A25
DAcbSh
AcbC
Pu
ec
Cl
ex
ProM
(PrCO)
MAcbSh
LAcbSh
aca
Nv
Tu
ICj
AO
lo
OPAI
OPro
Gu
A24d
A3a
LSD
LSV
SHi
PDZ
DEn
IEn
CxA
Pir
TPPro

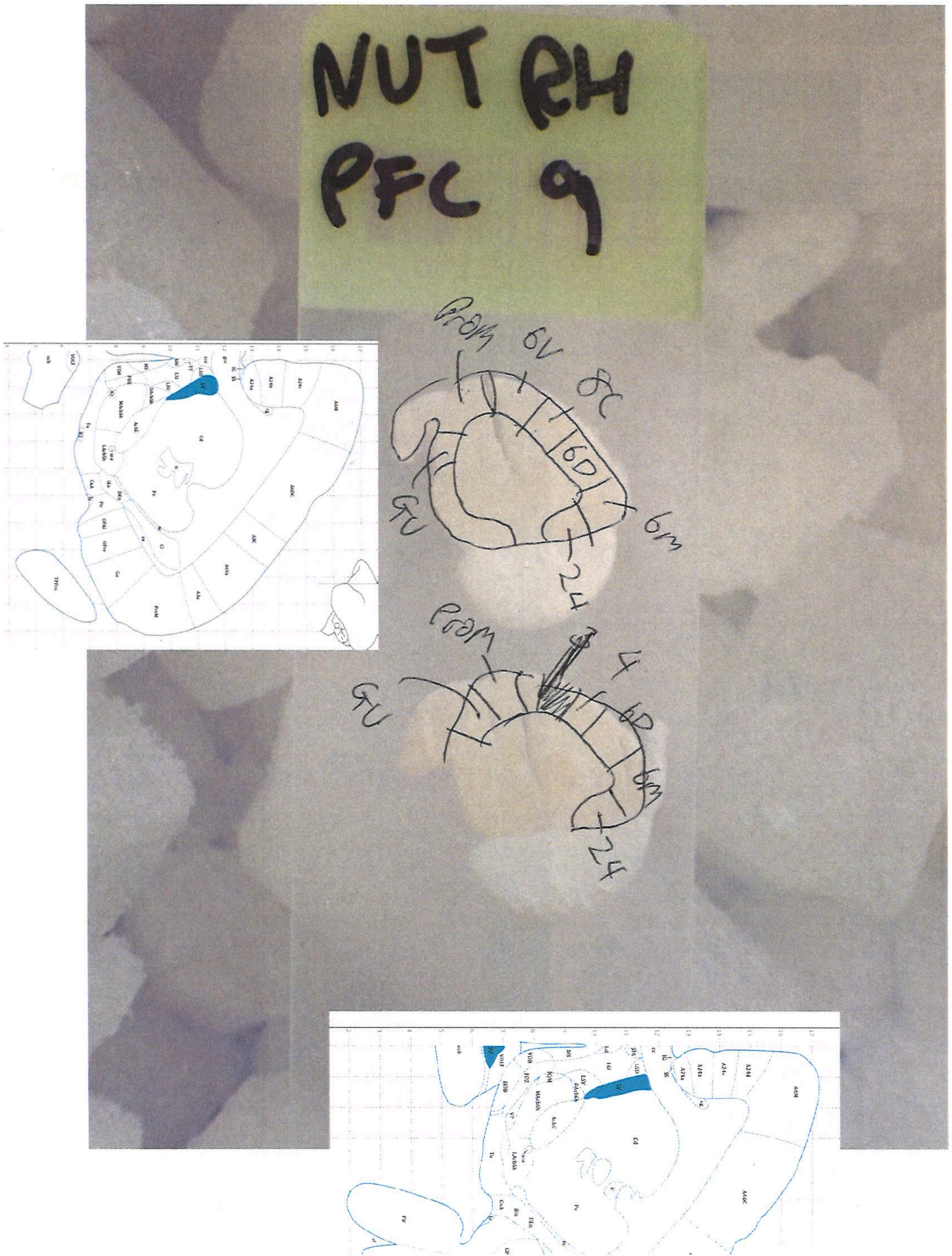
NUT RH
PFC 9

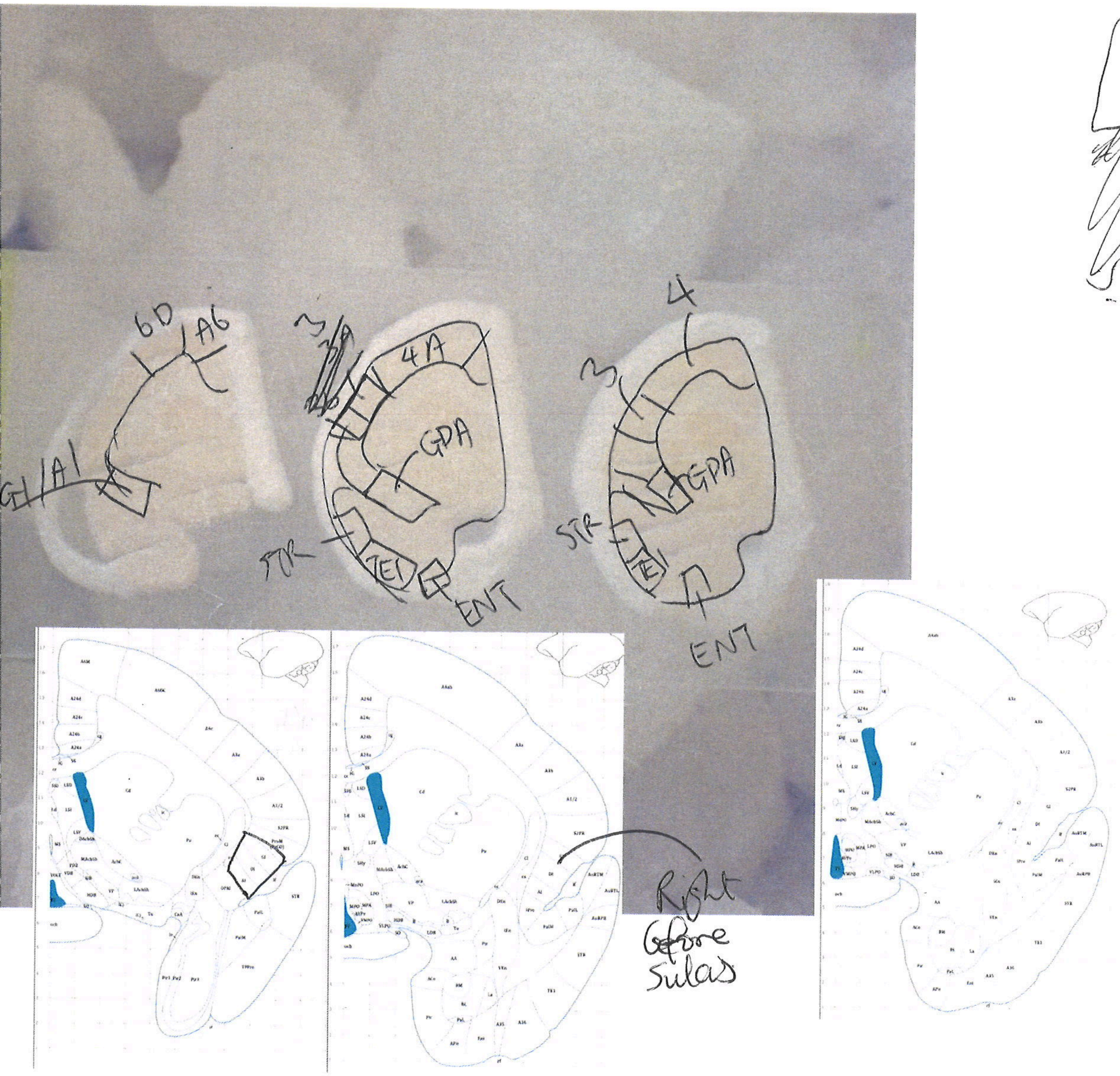
6D
A6
GI/AI
3/A
4A
GPA
STR
TEI
ENT
4
3
Right before Sulcus

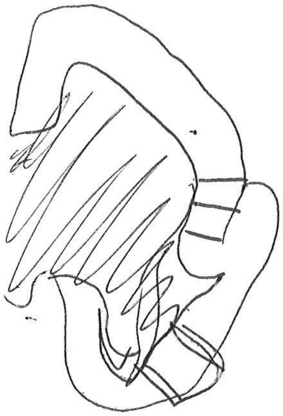

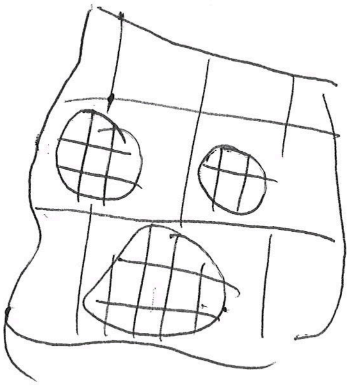

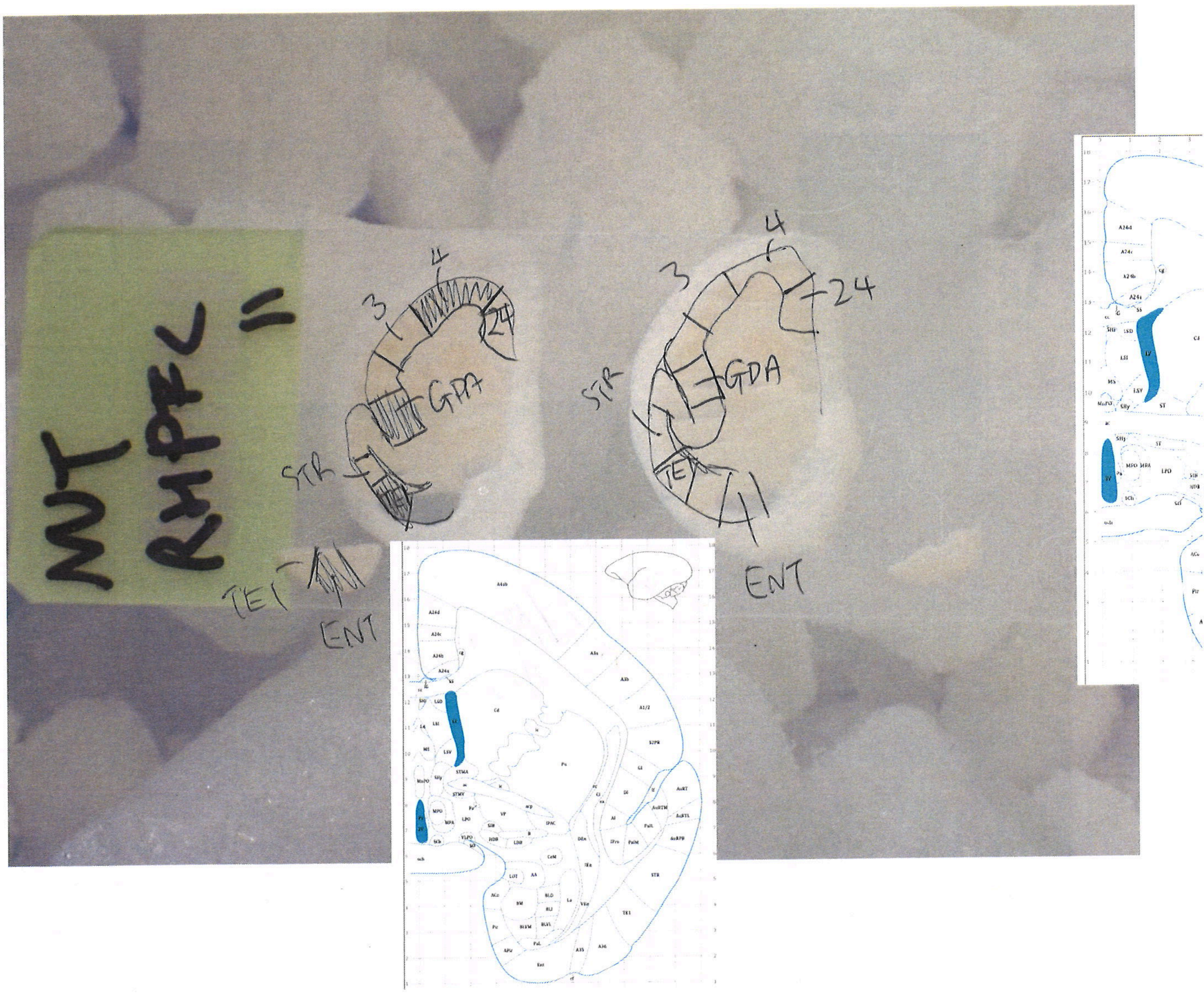
MUT
RMPFC
11
GDA
STR
TE
ENT
3
4
24

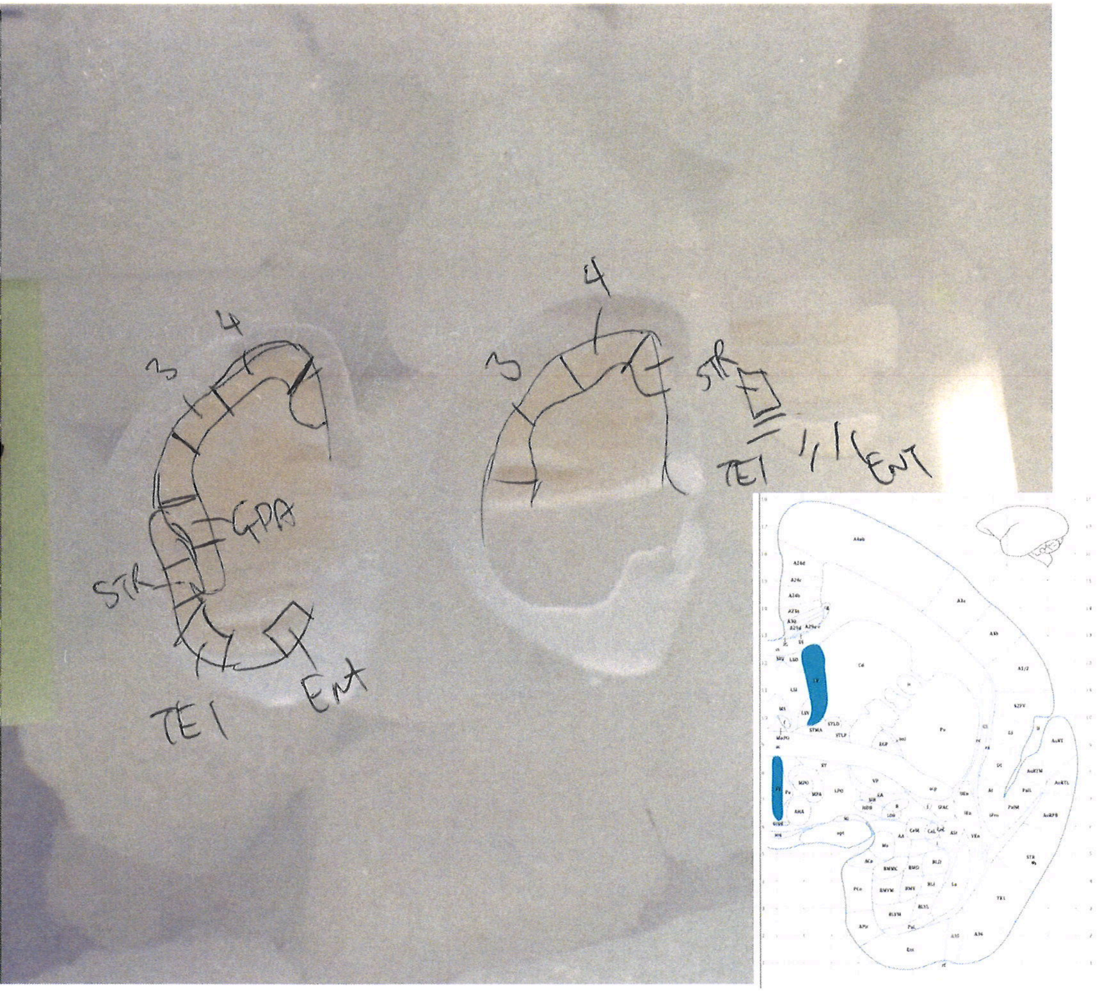
3
4
GPA
STR
TE1
Ent
3
4
STR
TE1
ENT

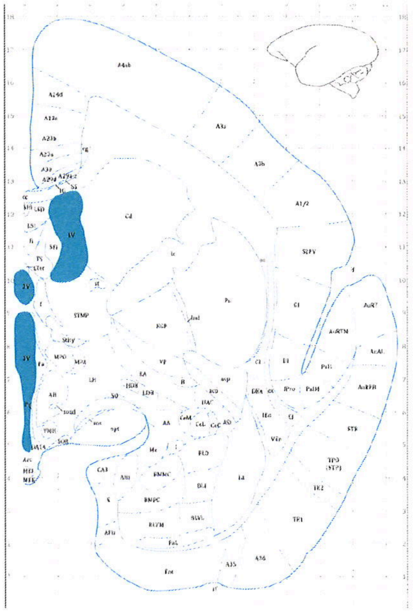

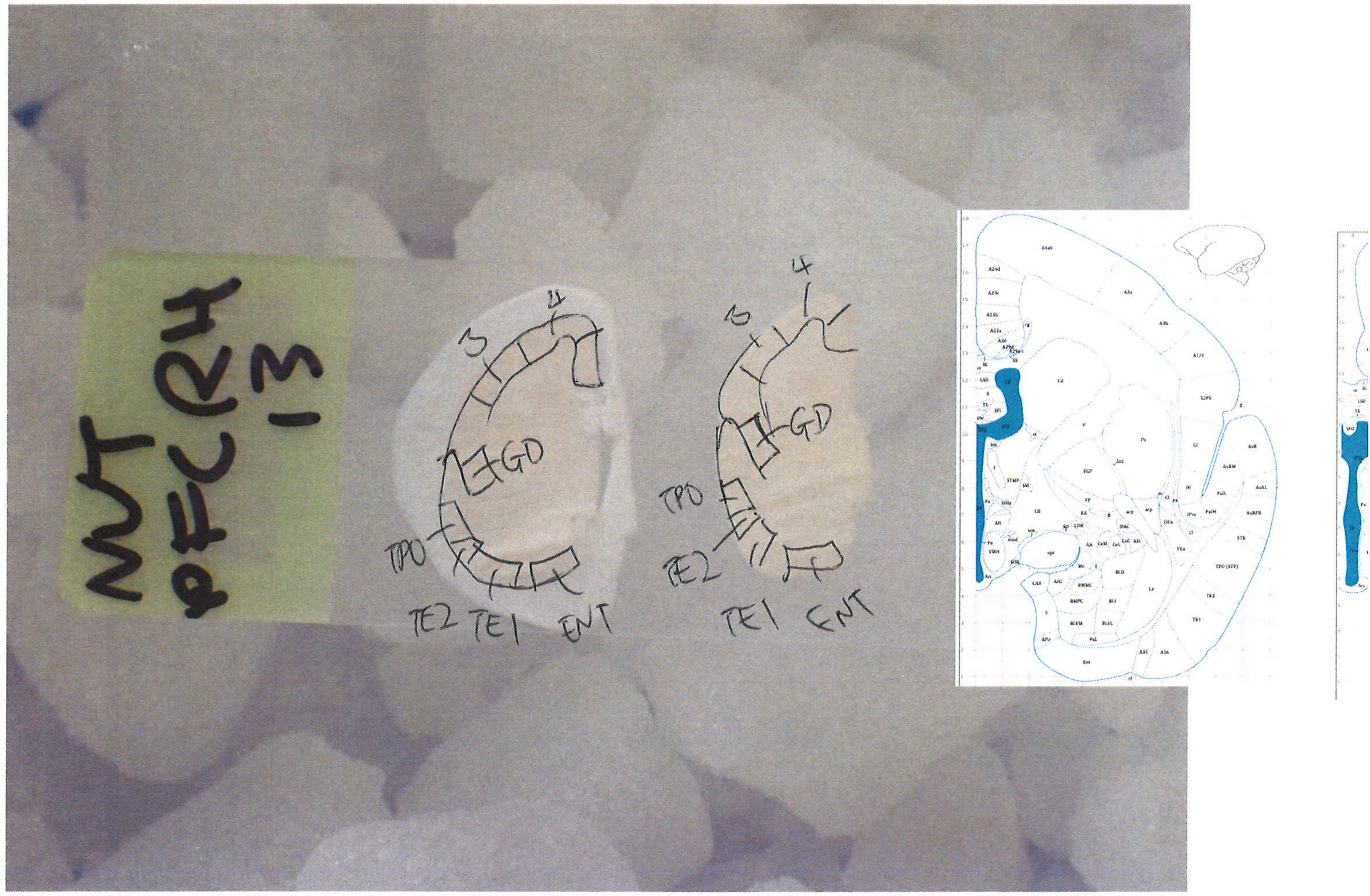

MUT
PFC RH
13
GD
TPO
TE2 TE1 ENT
3
2
GD
TPO
TE2
TE1 ENT
3
4

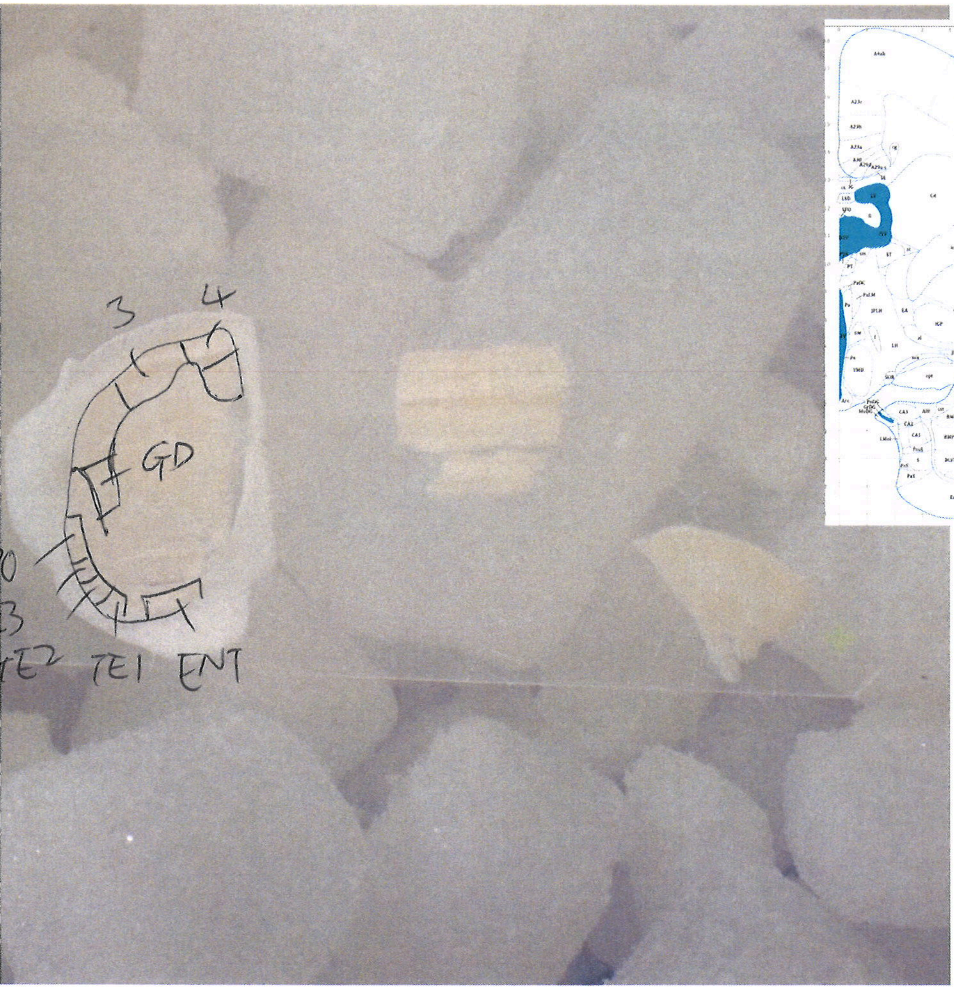
3
4
GD
TE1
ENT

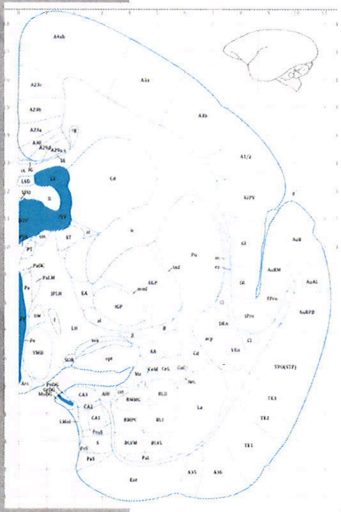

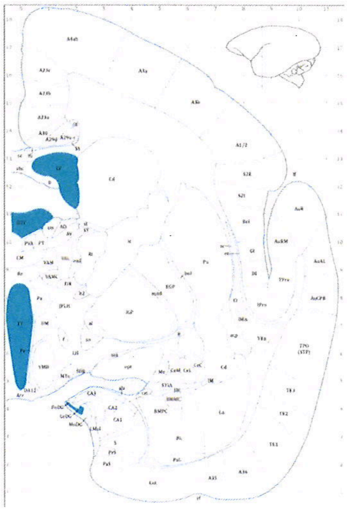

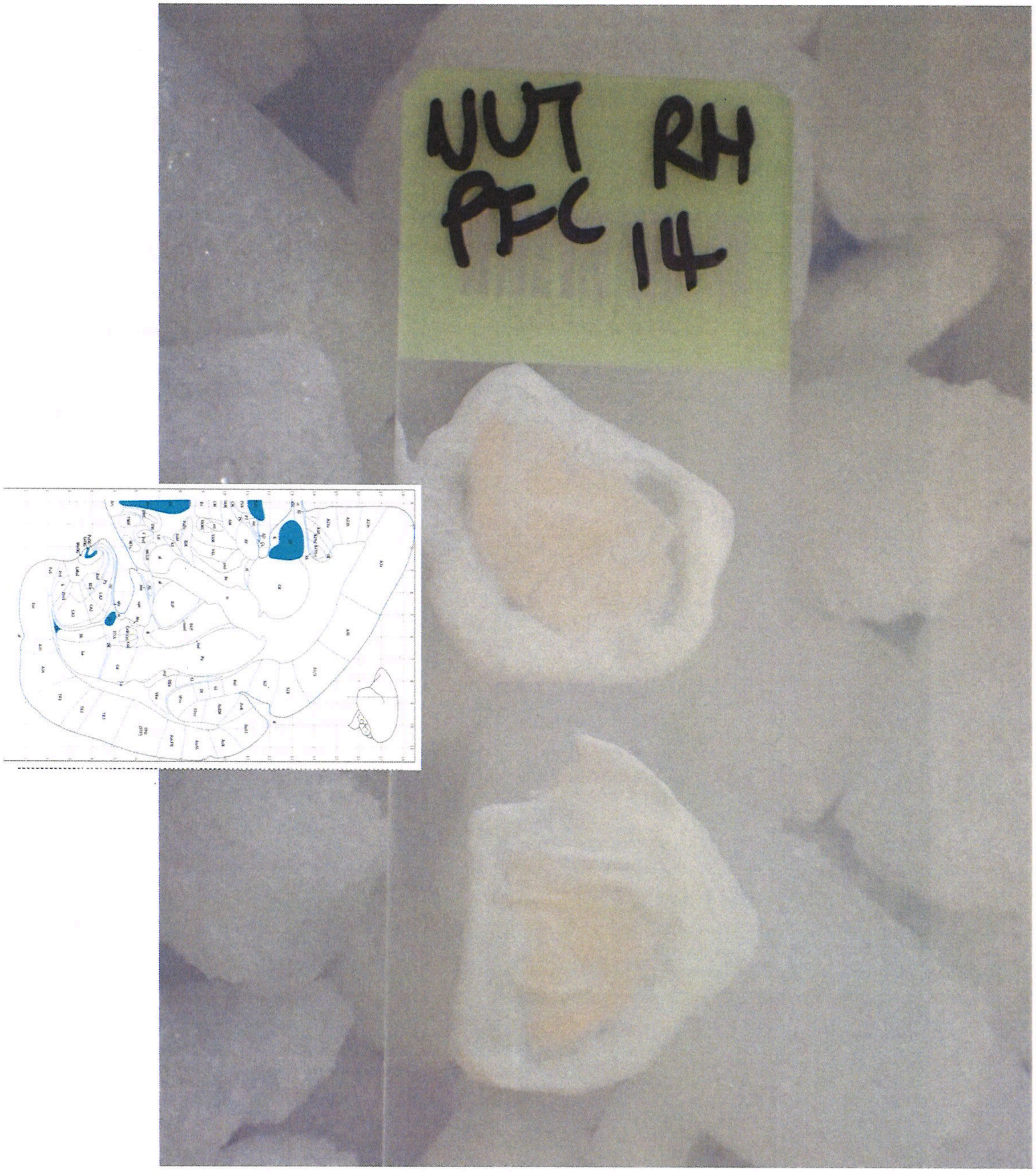
NUT RH
PFC 14

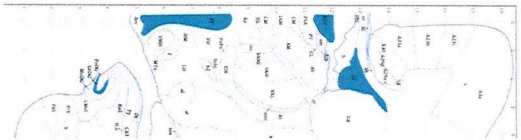

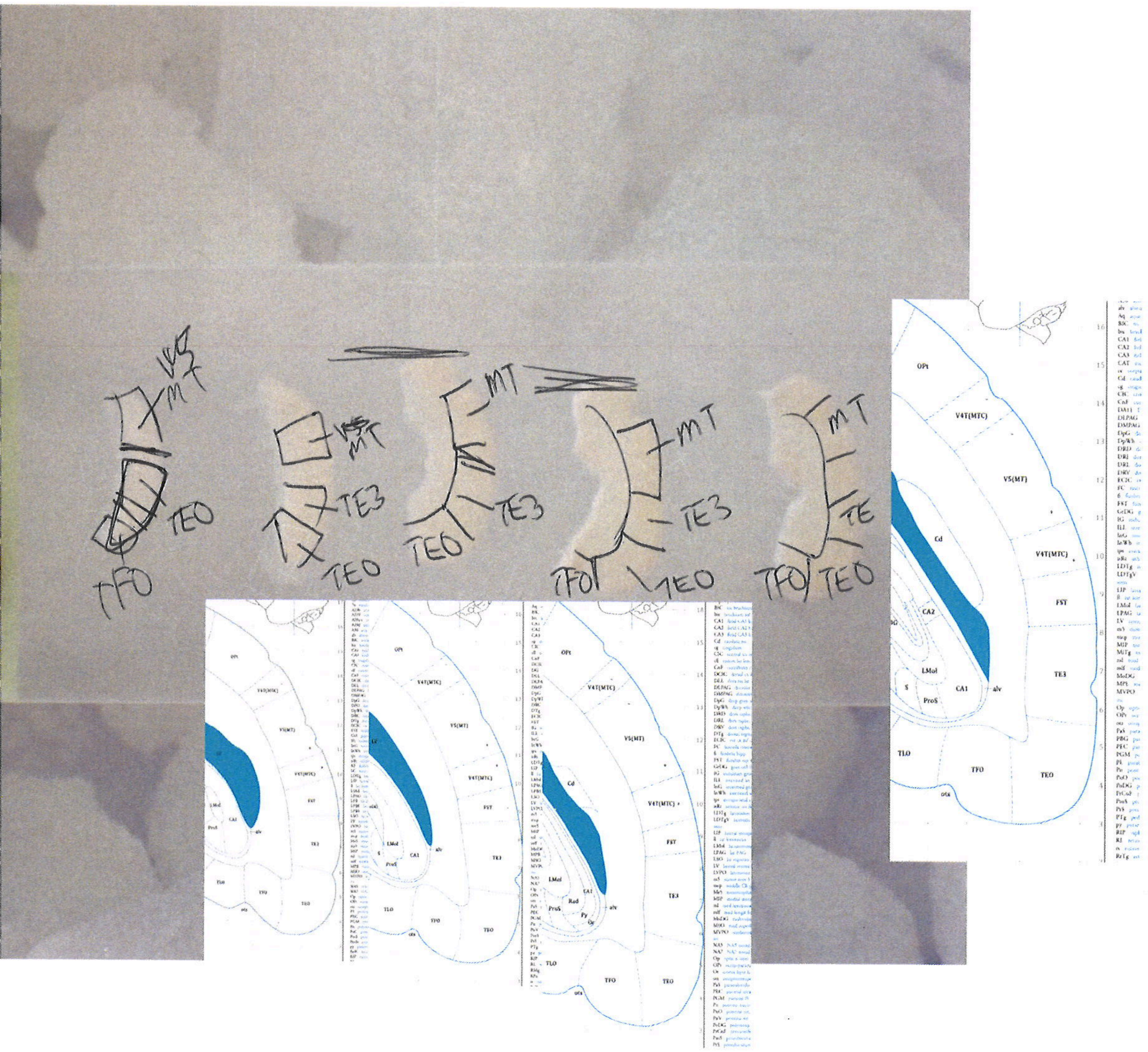

MT
TE3
TEO
TFO
OPt
V4T(MTC)
V5(MT)
FST
TLO
Cd
CA1
CA2
alv
LMol
ProS
S

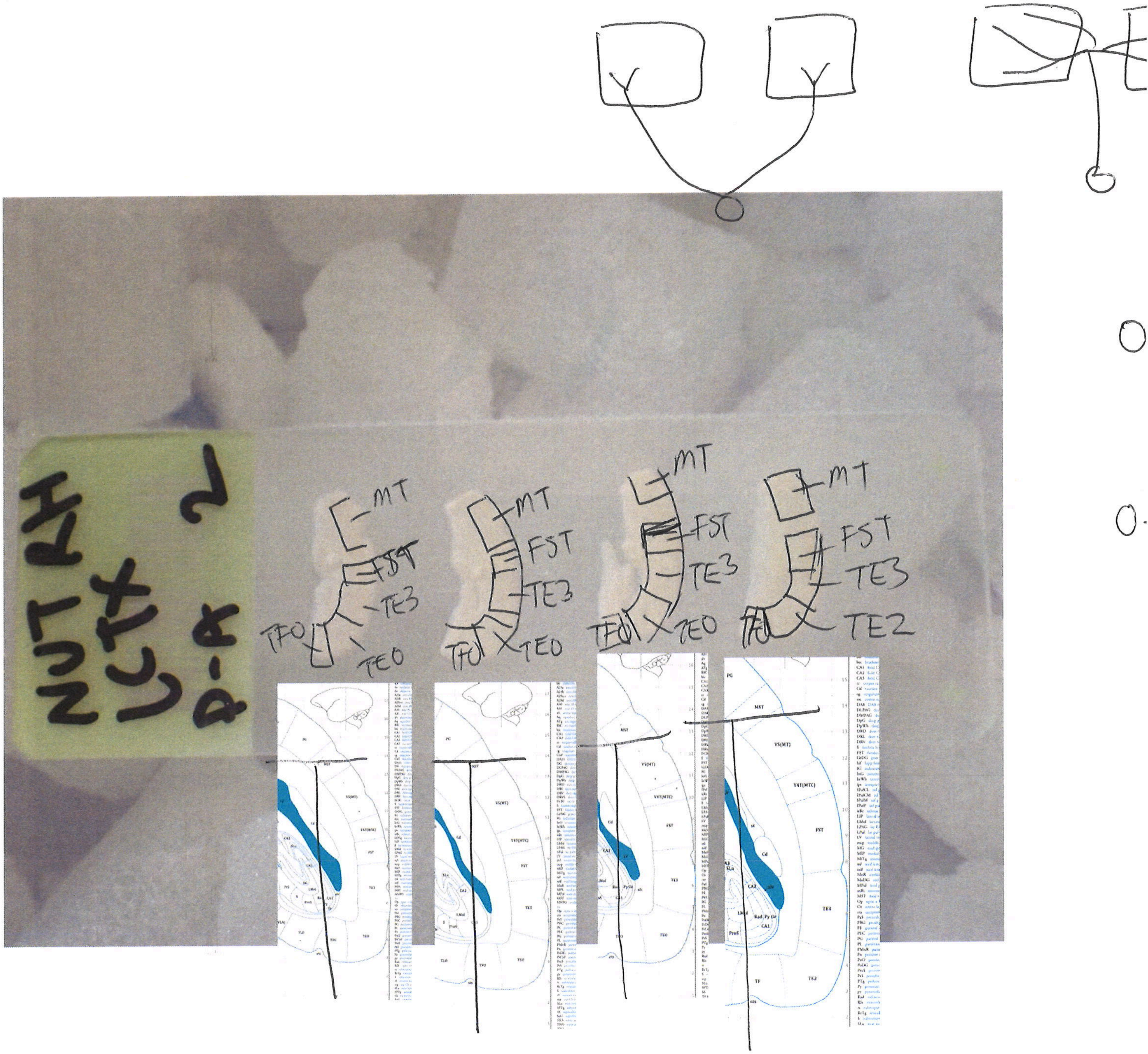

NUT RH
LCTX
P-A 2
MT
FST
TE3
TEO
TFO
TE2

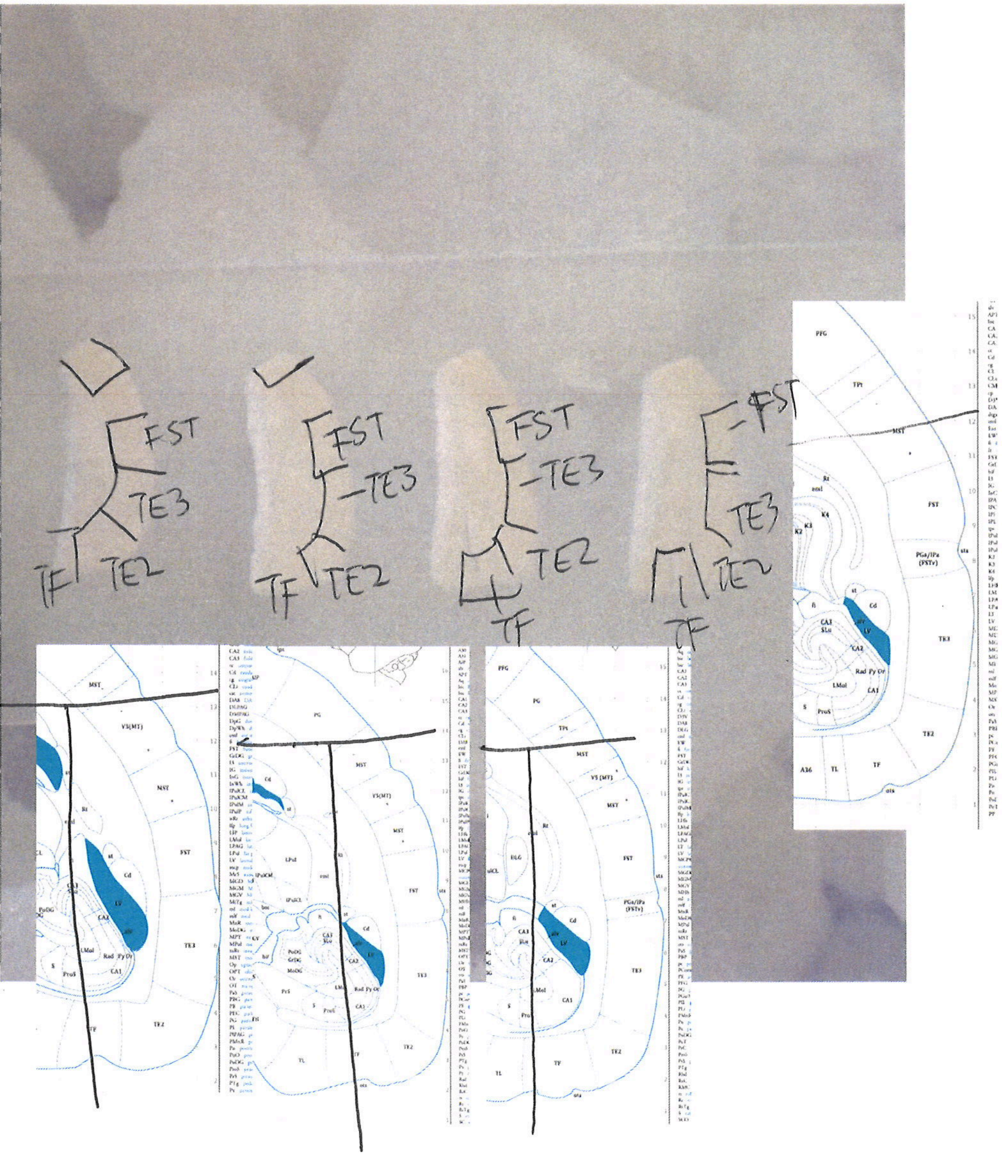
FST
TE3
TE2
TF
FST
TE3
TE2
TF
FST
TE3
TE2
TF
FST
TE3
TE2
TF

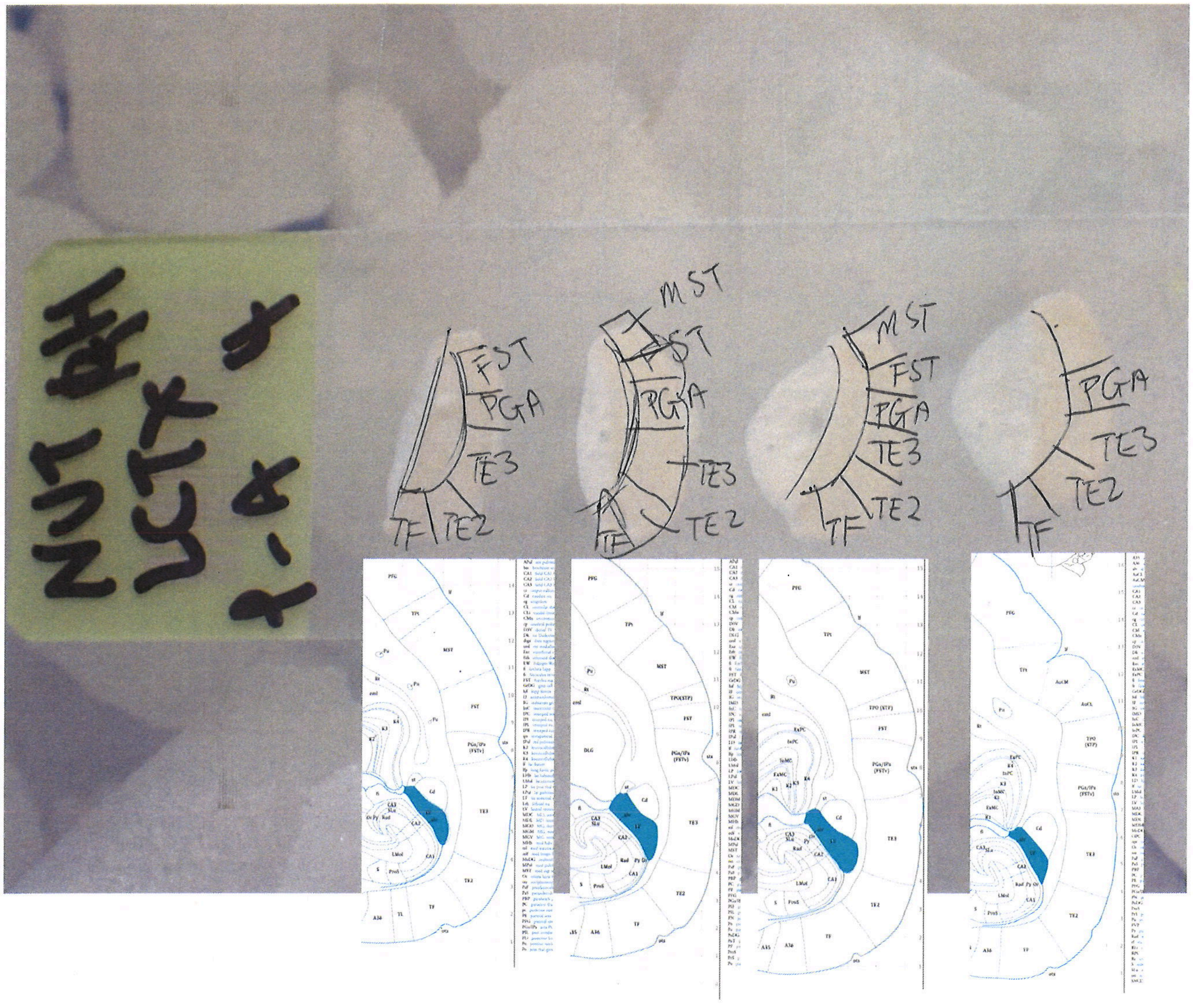
MST
FST
PGA
TE3
TE2
TF

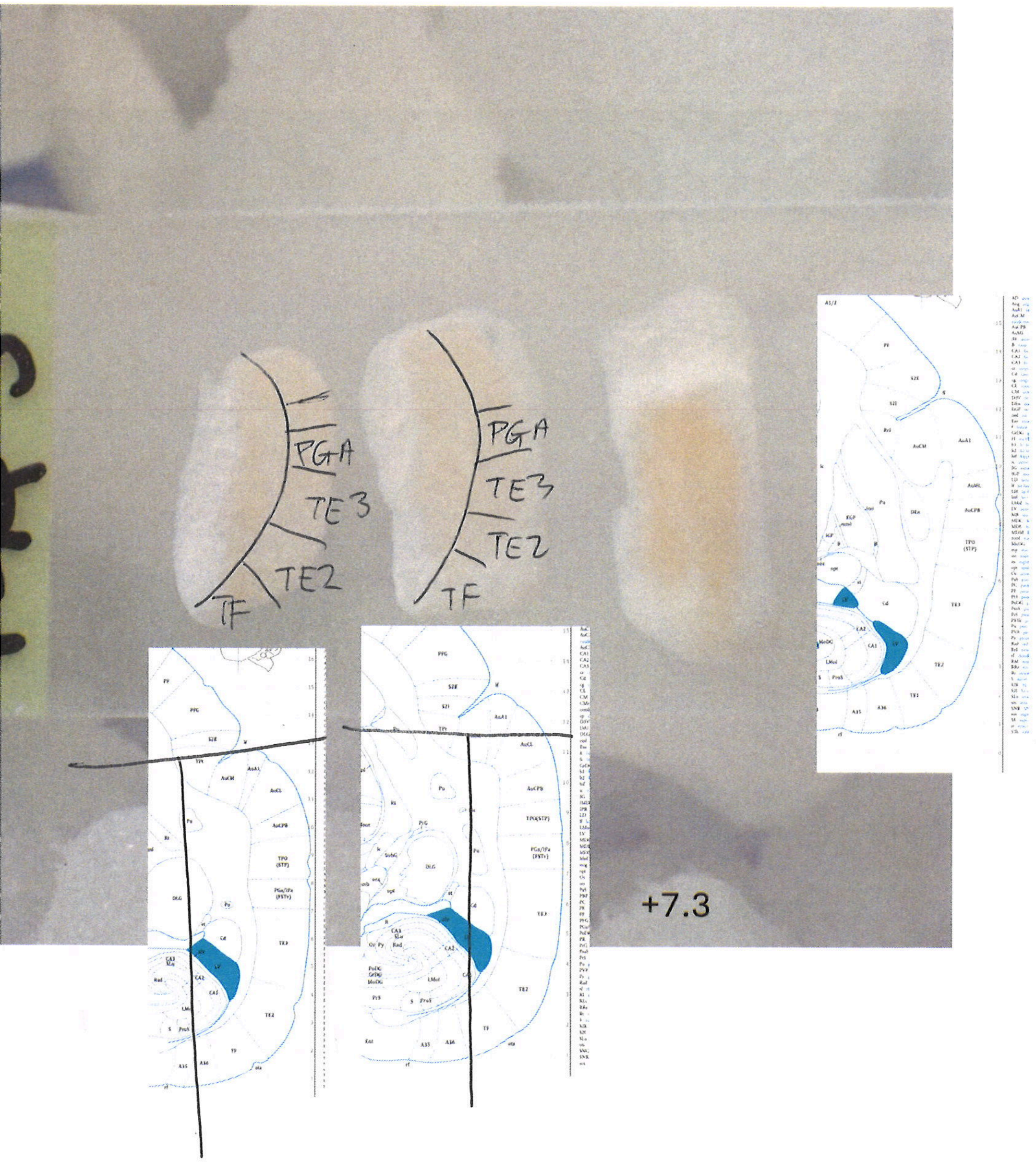
PGA
TE3
TE2
TF
PGA
TE3
TE2
TF
+7.3

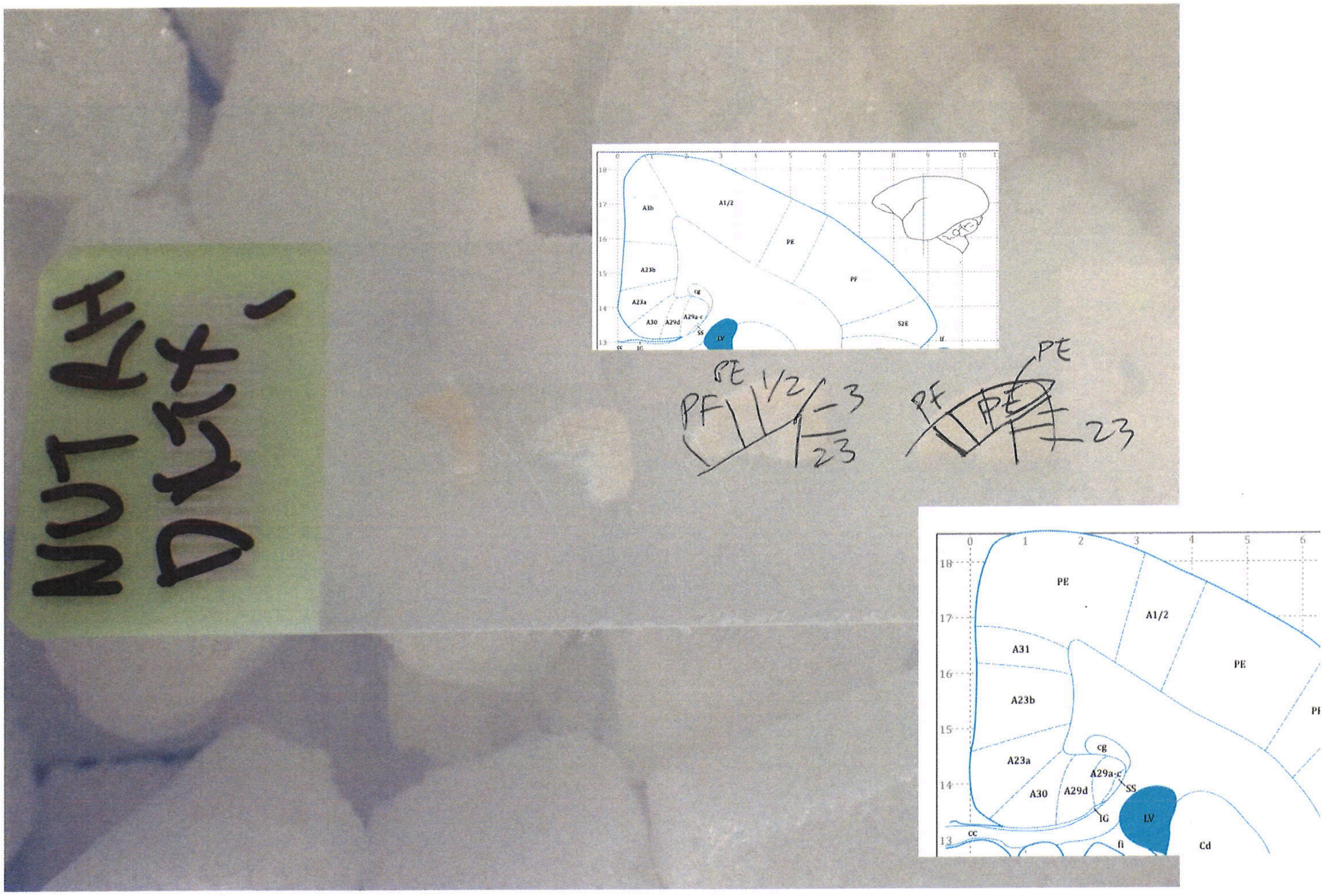

NUT RH
A1/2
A3b
PE
PF
A23b
A23a
A30
A29d
A29a-c
cg
SS
LV
S2E
A31
IG
cc
fi
Cd
PF
PE
1/2
3
23

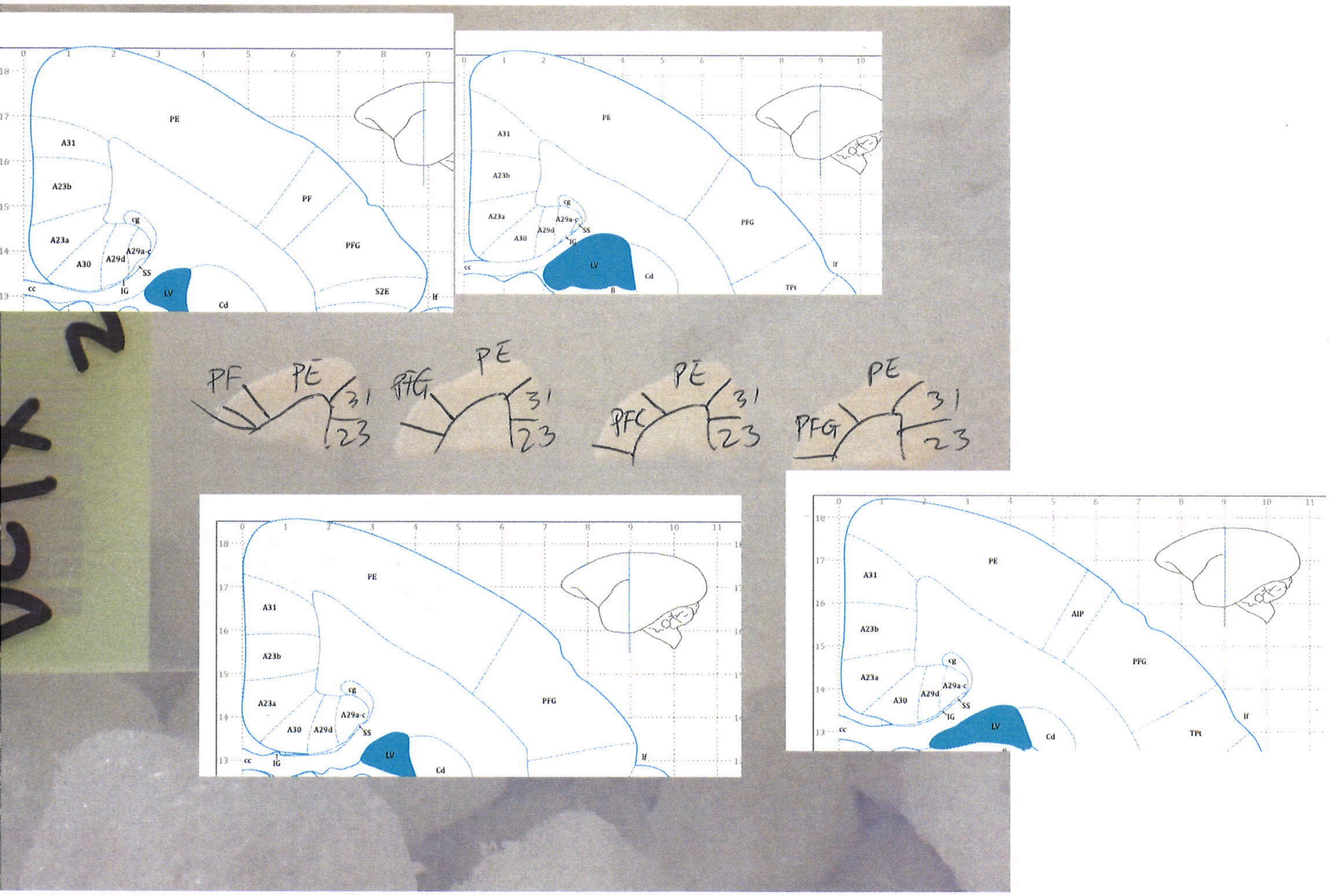

PE
A31
A23b
A23a
A30
A29d
A29a-c
cg
SS
IG
cc
LV
Cd
PF
PFG
S2E
lf
TPt
AIP
PF PE 31 23
PFG PE 31 23
PE 31 23
PFG PE 31 23

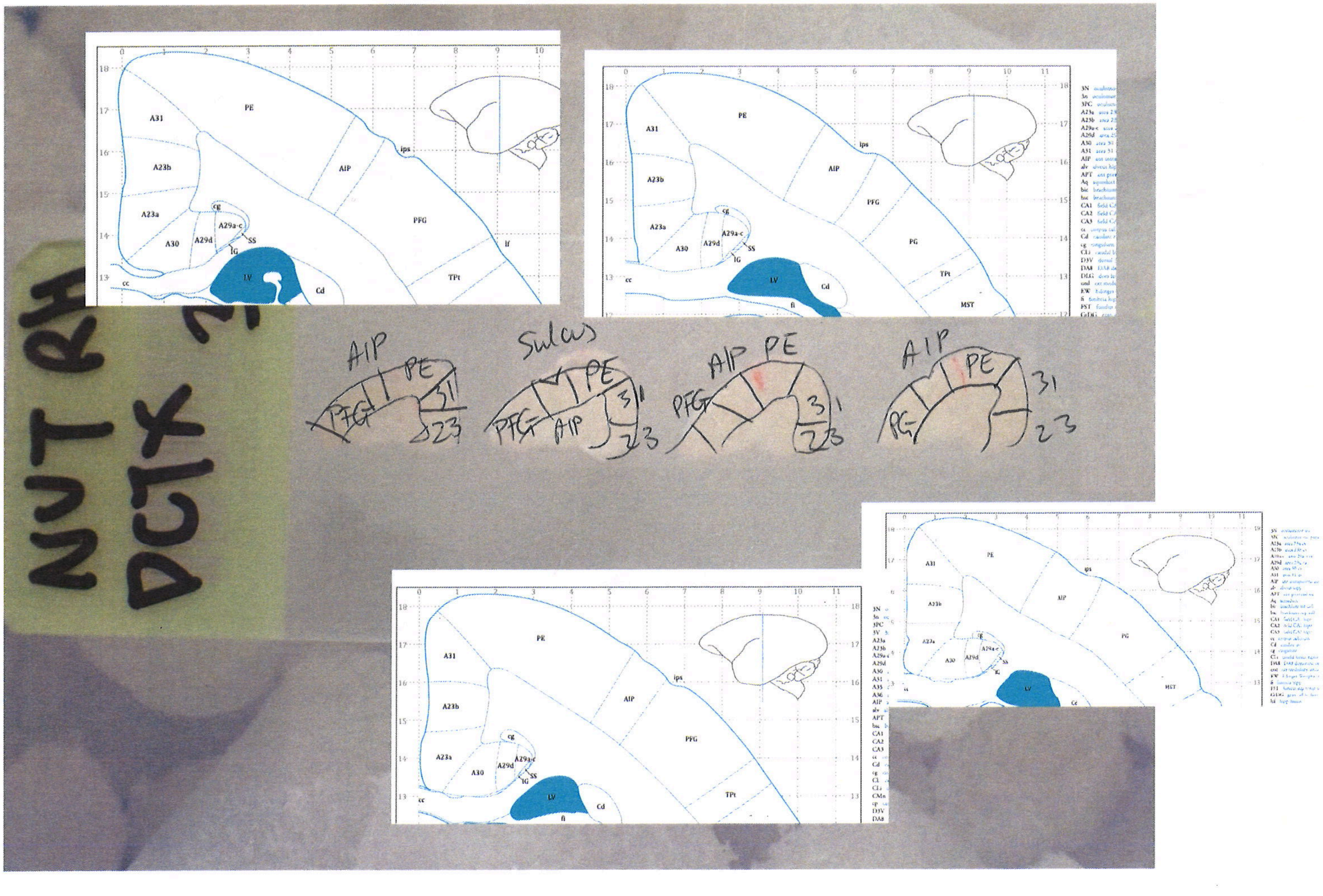
NUT RH
DCTX 3
AIP
PE
PFG
31
23
Sulcus
PG
A31
A23b
A23a
A30
A29d
A29a-c
SS
IG
cg
LV
Cd
cc
fi
ips
TPt
lf
MST

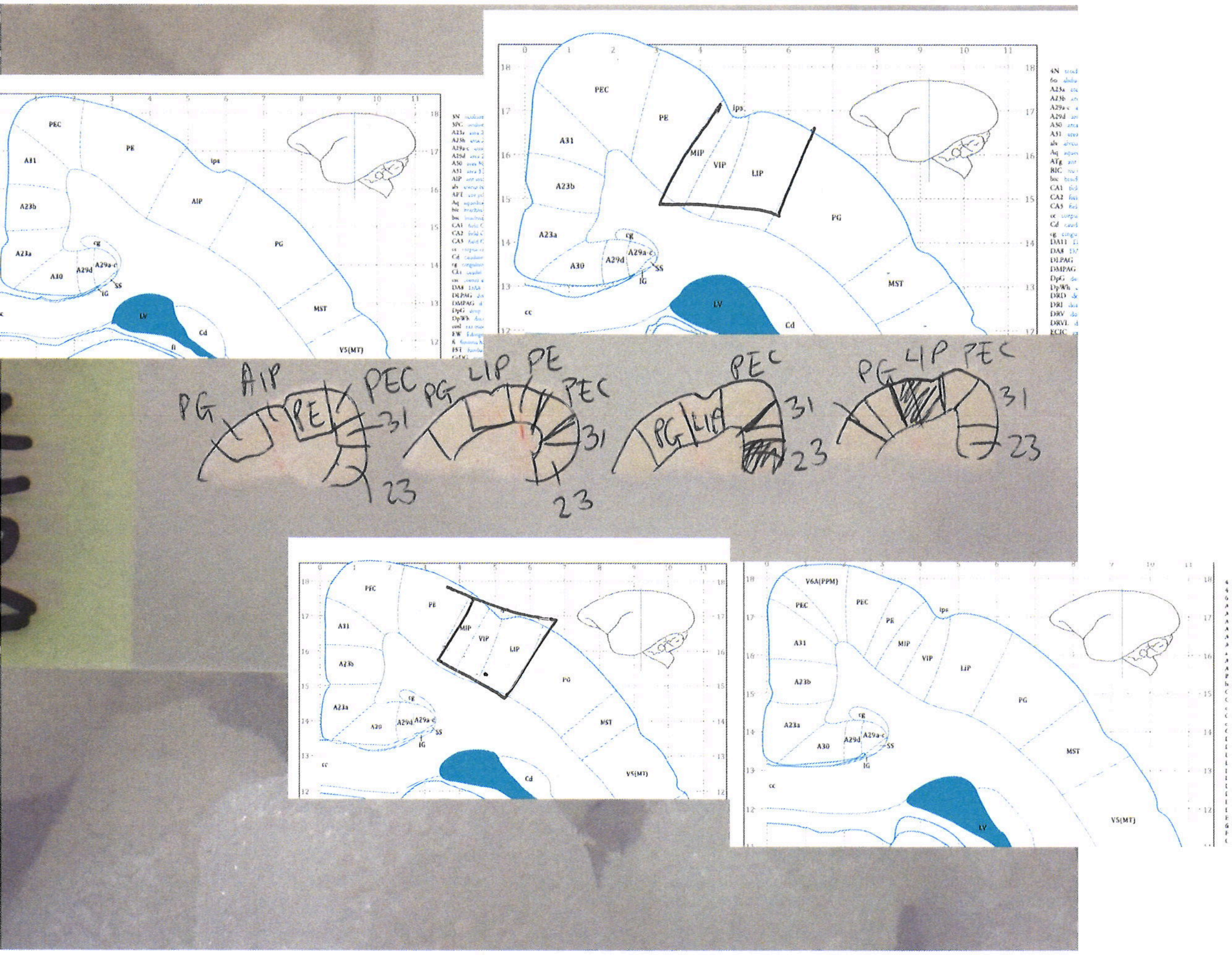
PG AIP PE PEC 31 23
PG LIP PE PEC 31 23
PEC PG LIP 31 23
PG LIP PEC 31 23
PEC
PE
ips
A31
A23b
A23a
A30
A29d
A29a-c
cg
SS
IG
AIP
PG
MIP
VIP
LIP
MST
LV
Cd
cc
V5(MT)
V6A(PPM)

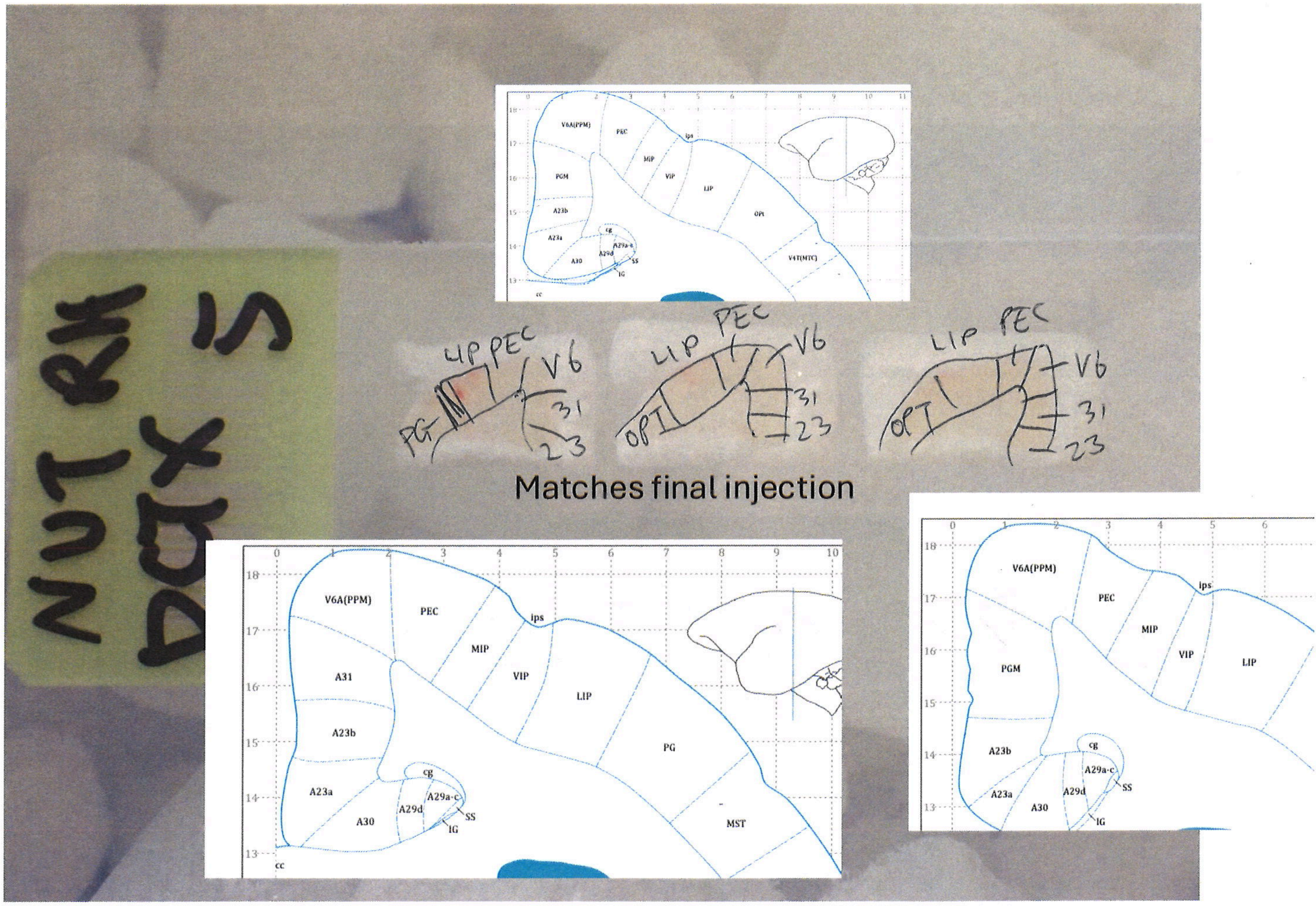
NUT RH
DAX 5
LIP PEC V6 PG 31 23
LIP PEC V6 OPT 31 23
LIP PEC V6 OPT 31 23
Matches final injection
V6A(PPM)
PEC
ips
MIP
VIP
LIP
PGM
A31
A23b
A23a
A30
A29d
A29a-c
cg
SS
IG
PG
MST
OPt
V4T(MTC)
cc

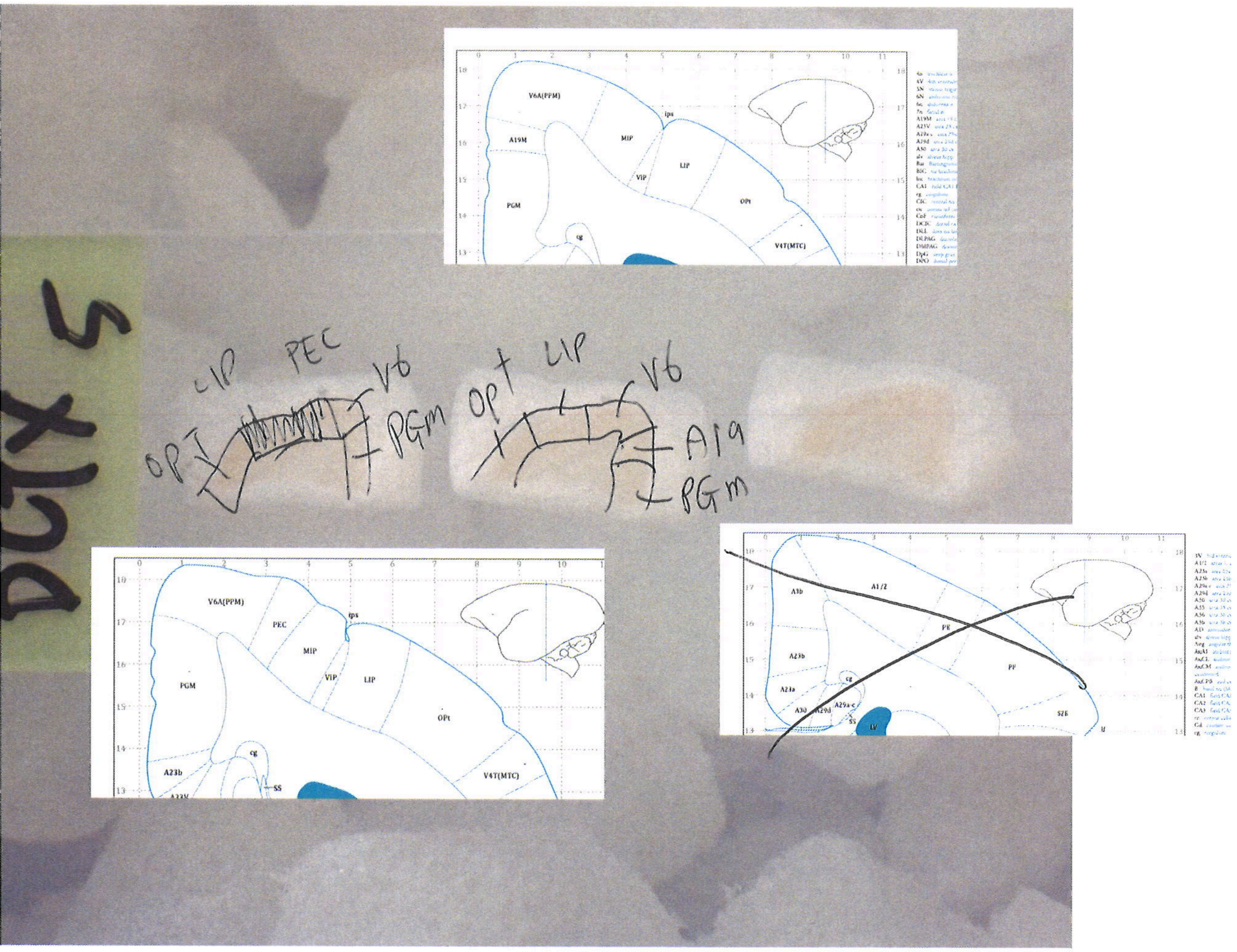

DCTX 5
LIP
PEC
V6
OPT
PGm
Opt
LIP
V6
A19
PGm
V6A(PPM)
ips
A19M
MIP
VIP
LIP
PGM
OPt
cg
V4T(MTC)
PEC
A23b
SS
A1/2
A3b
PE
PF
A23a
A30
A29a-c
S2E

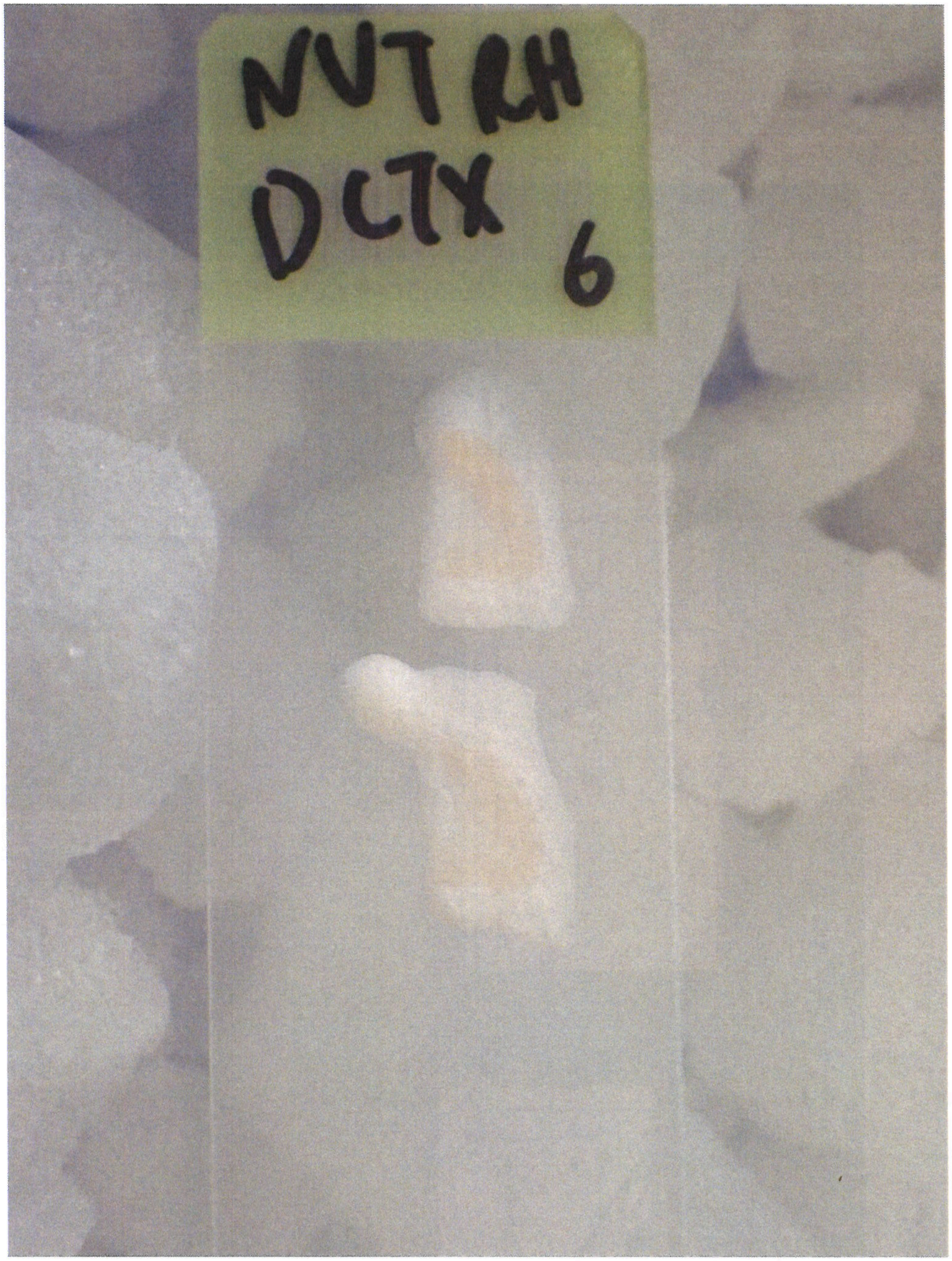
NVT RH
DCTX
6

HEMISPHERE

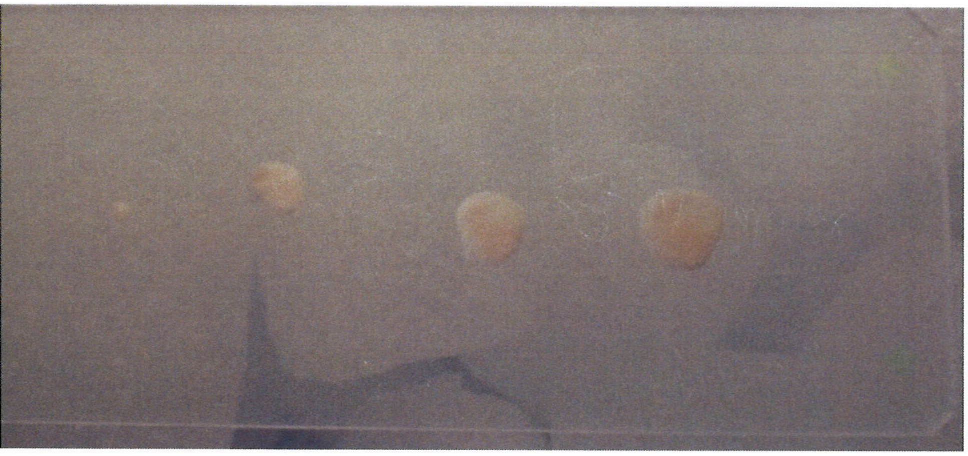

ALL V1

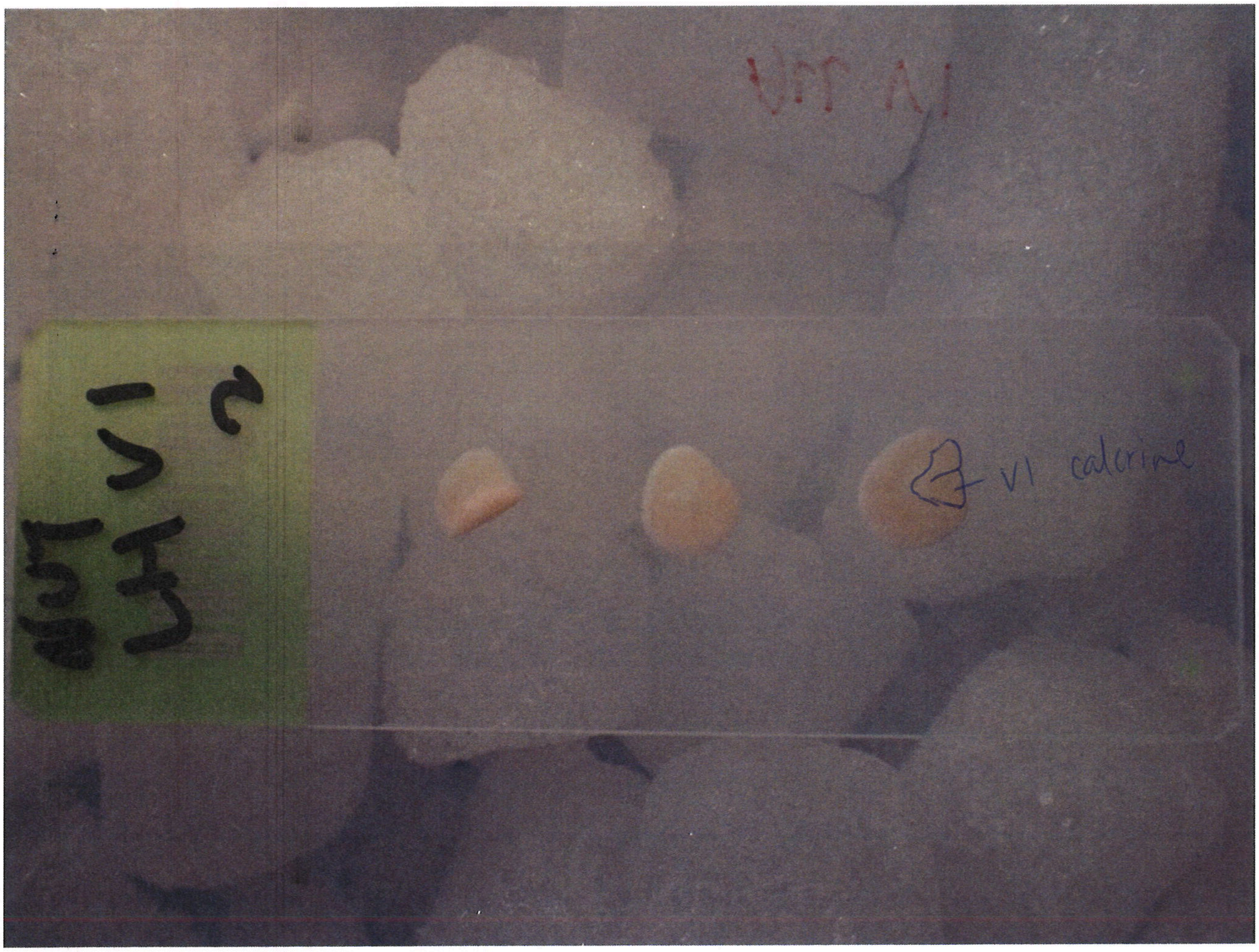

All V1

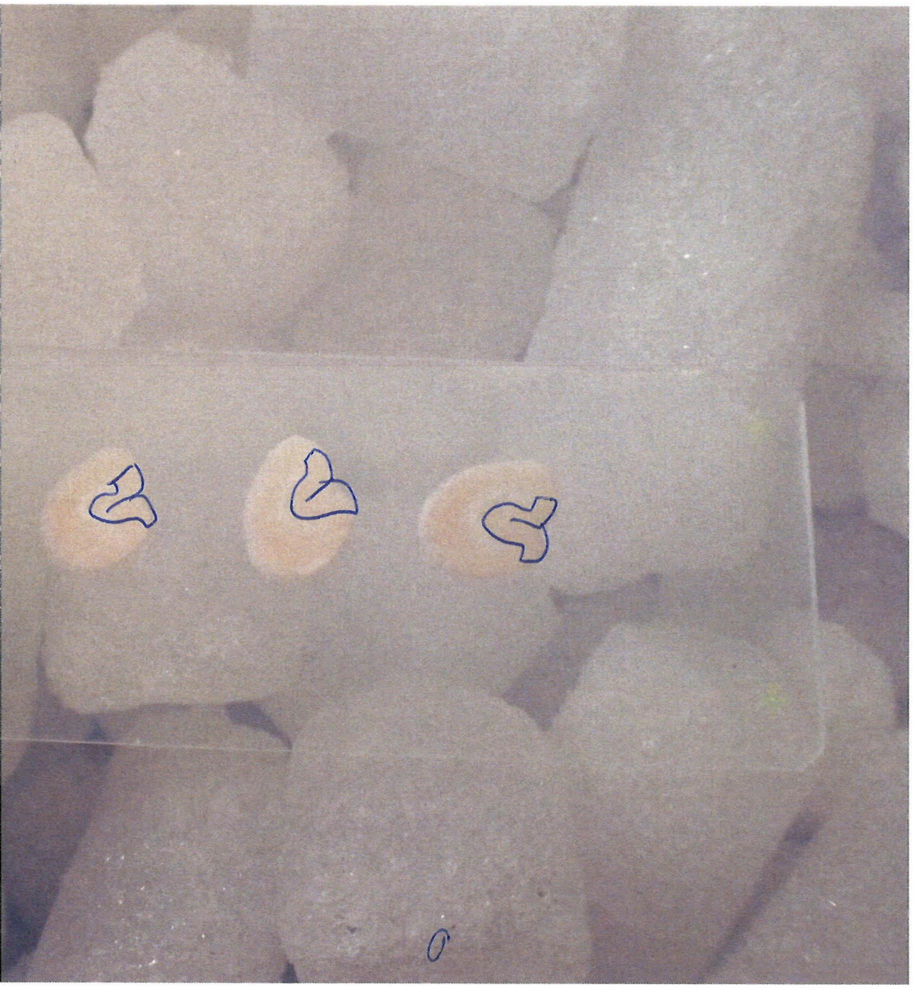

all V1

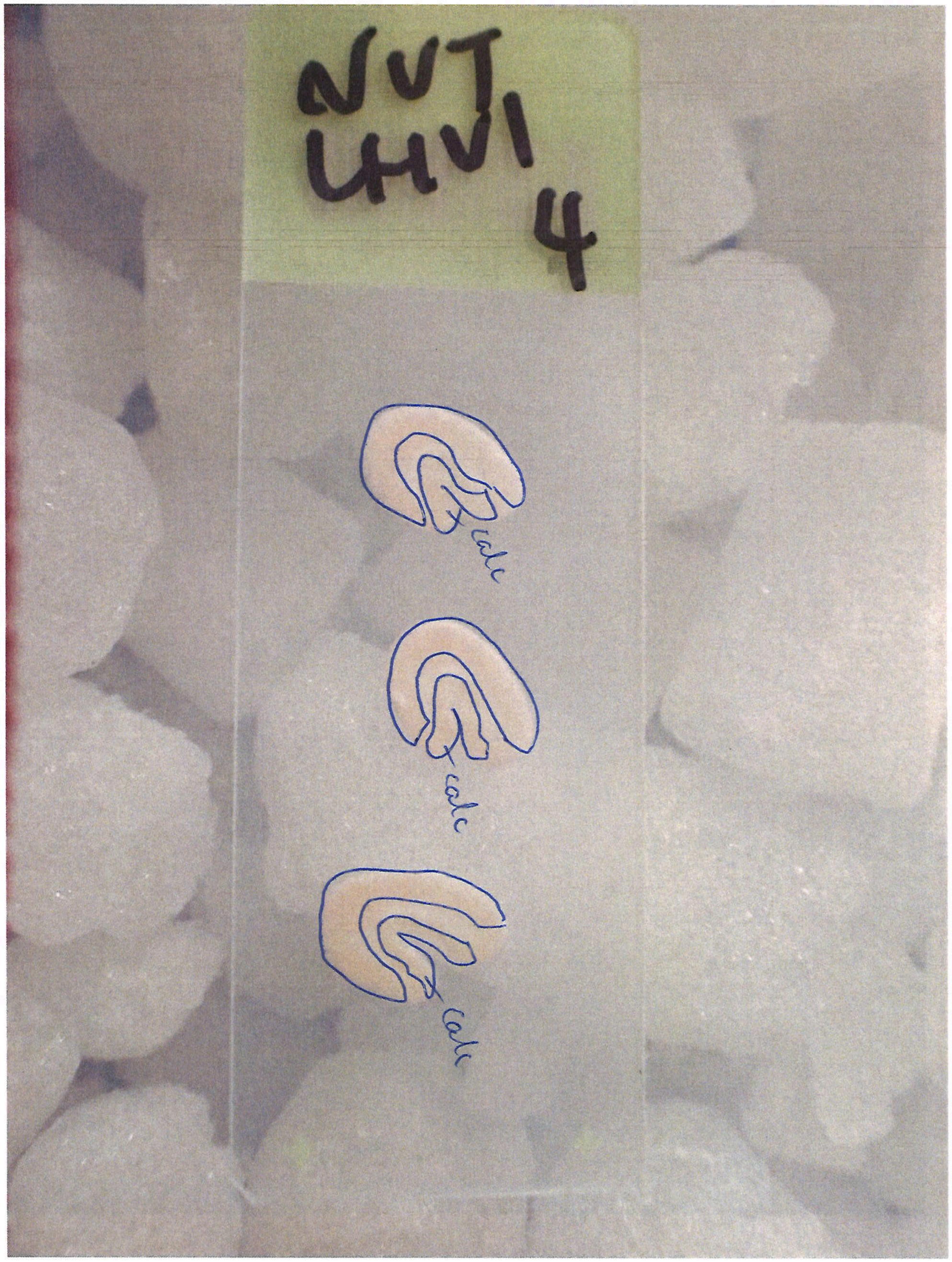
NVT
LHVI
4
calc
calc
calc

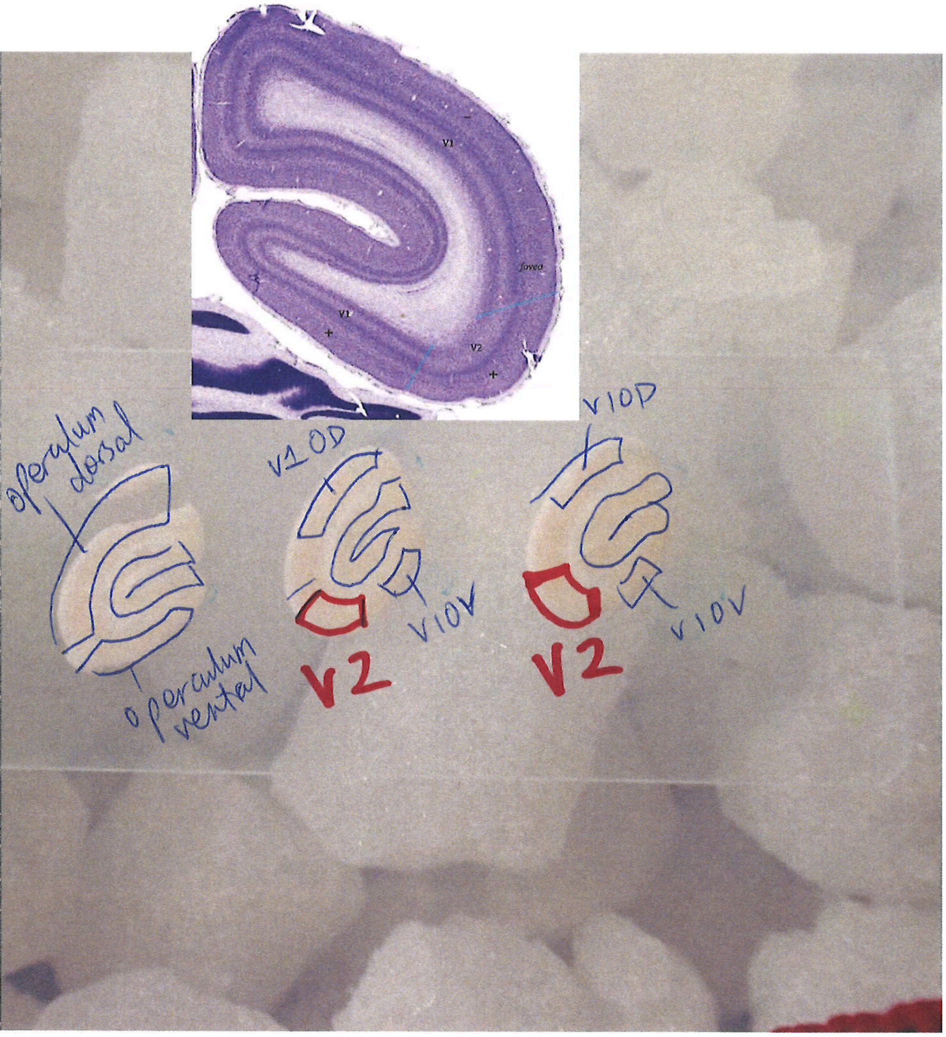

V1
fovea
V1
V2
operculum dorsal
operculum ventral
V1 OD
V1 OV
V2
V1 OD
V1 OV
V2

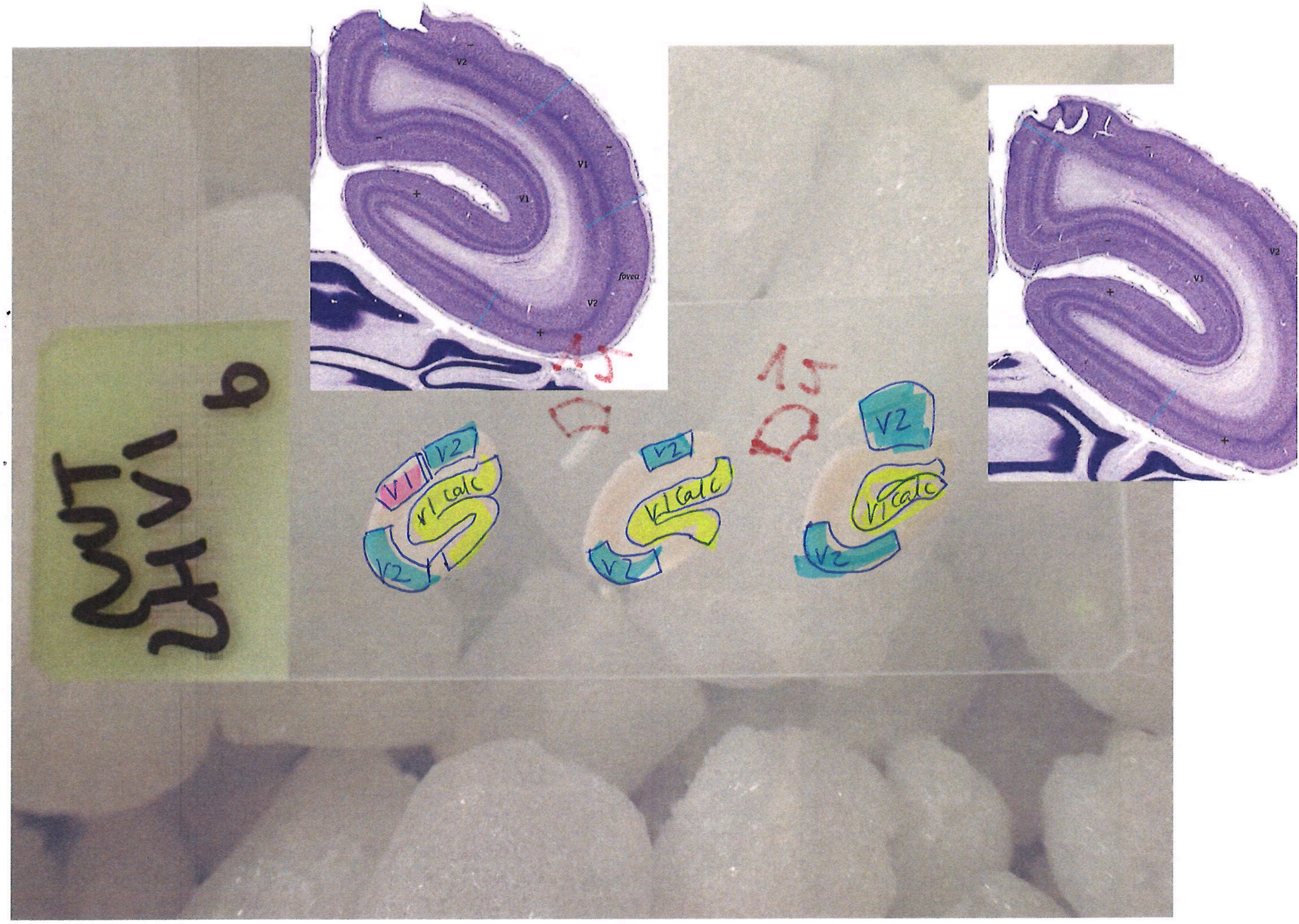

V2
V1
fovea
V1 calc

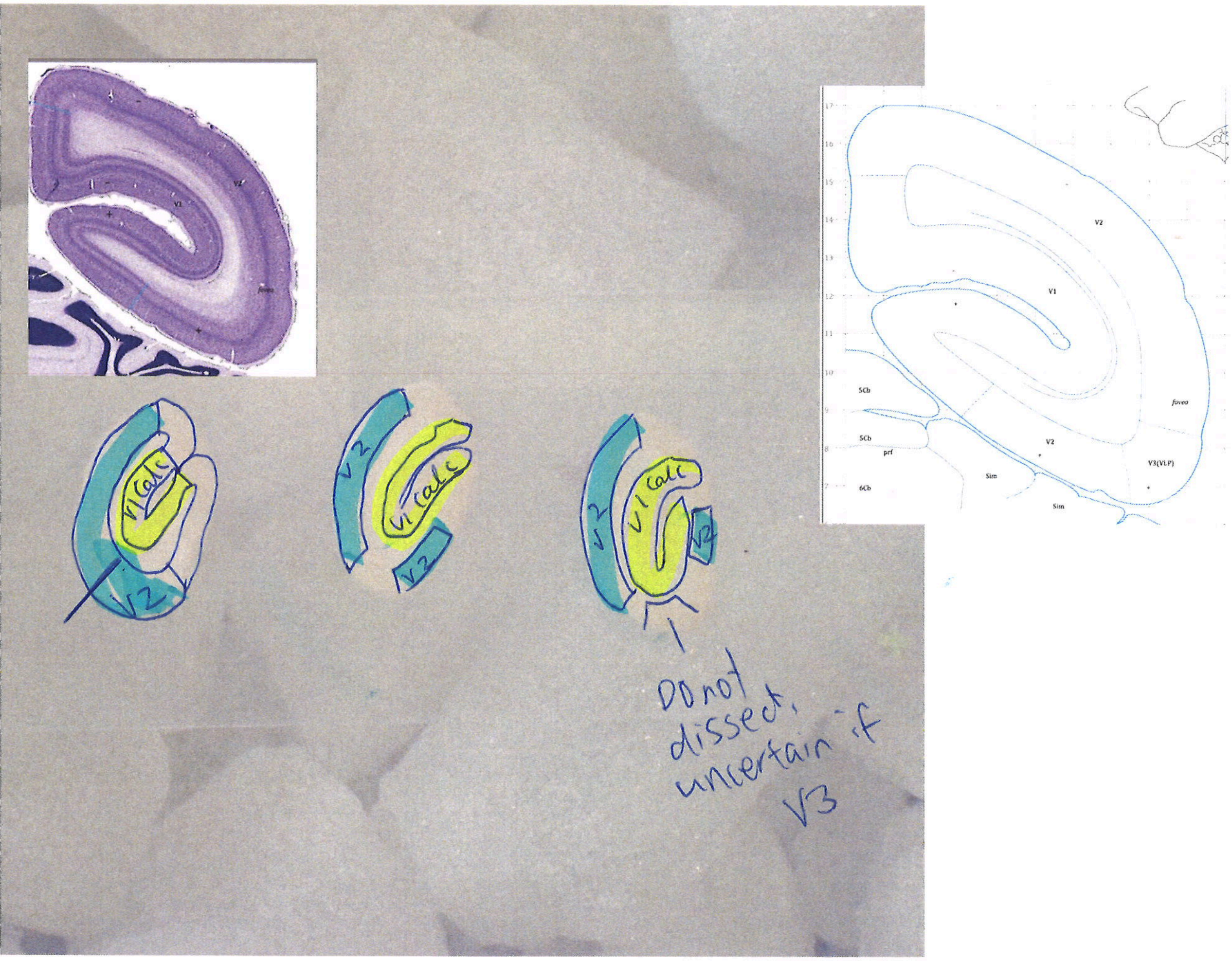
V1 calc
V2
Do not dissect, uncertain if V3
V2
V1
fovea
5Cb
5Cb
prf
6Cb
Sim
Sim
V3(VLP)

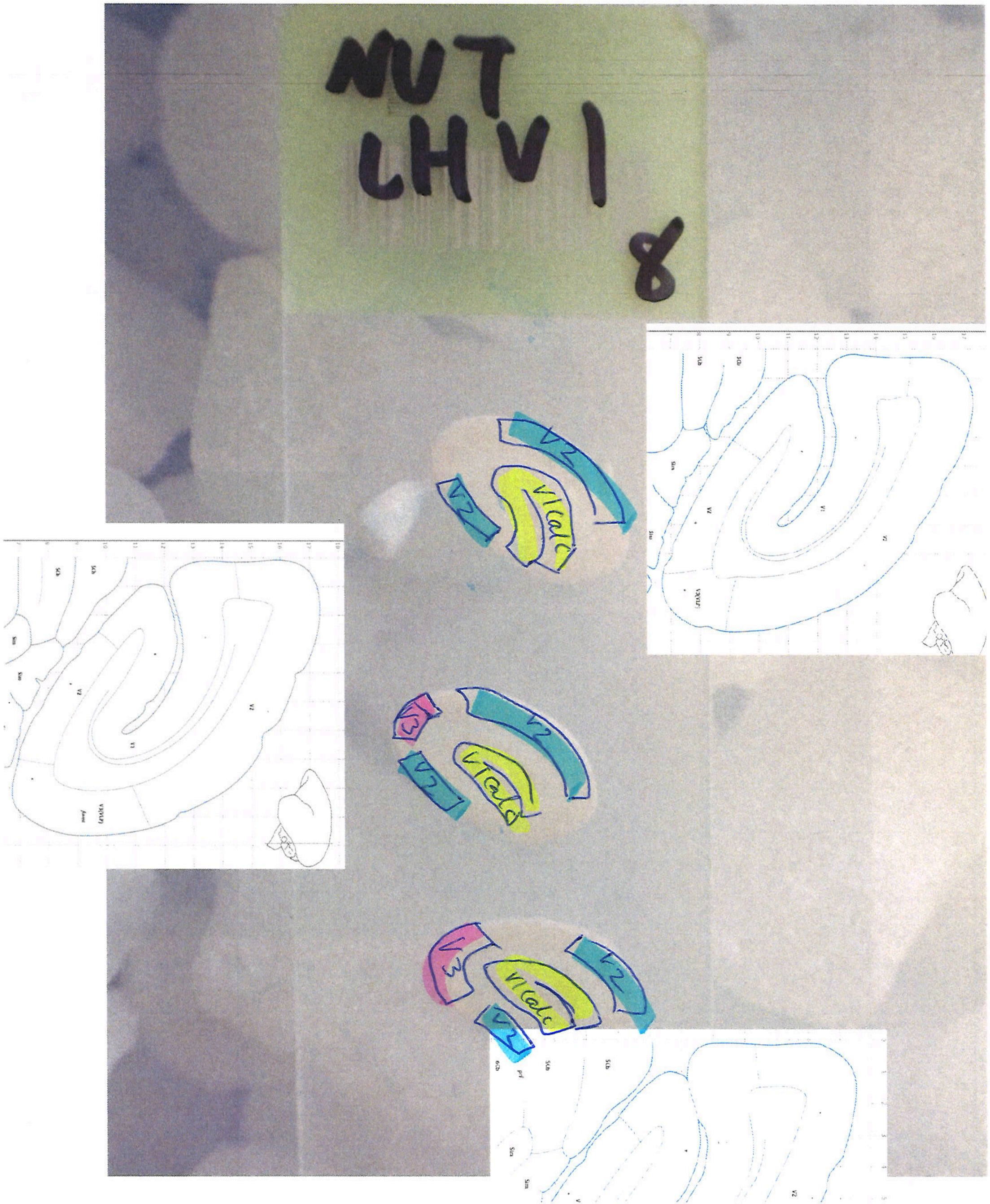
MUT
LHV1
8
V1a,b,c
V2
V3

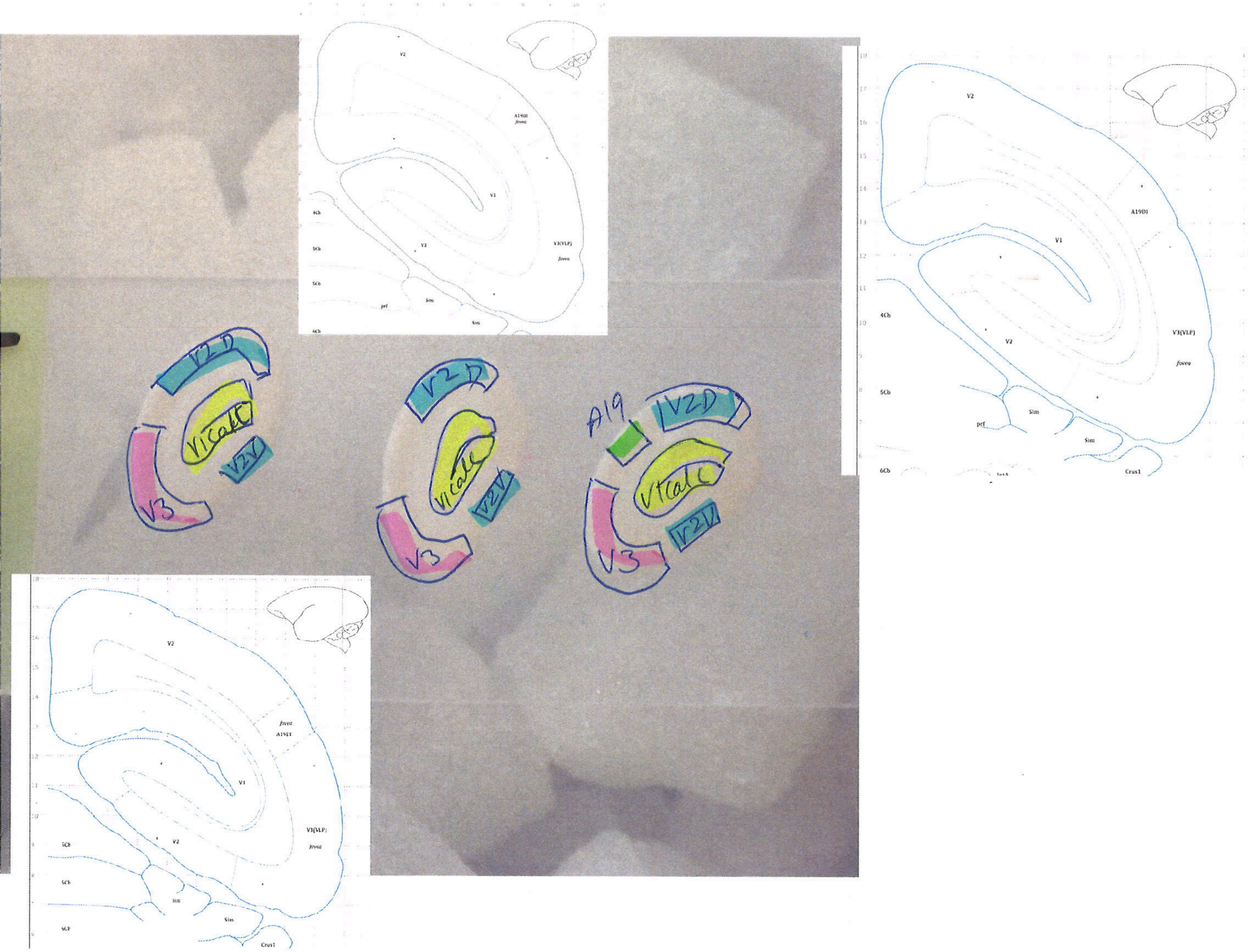

V2D
V1calc
V2V
V3
A19
V2
A19DI
fovea
V1
V3(VLP)
4Cb
5Cb
6Cb
prf
Sim
Crus1

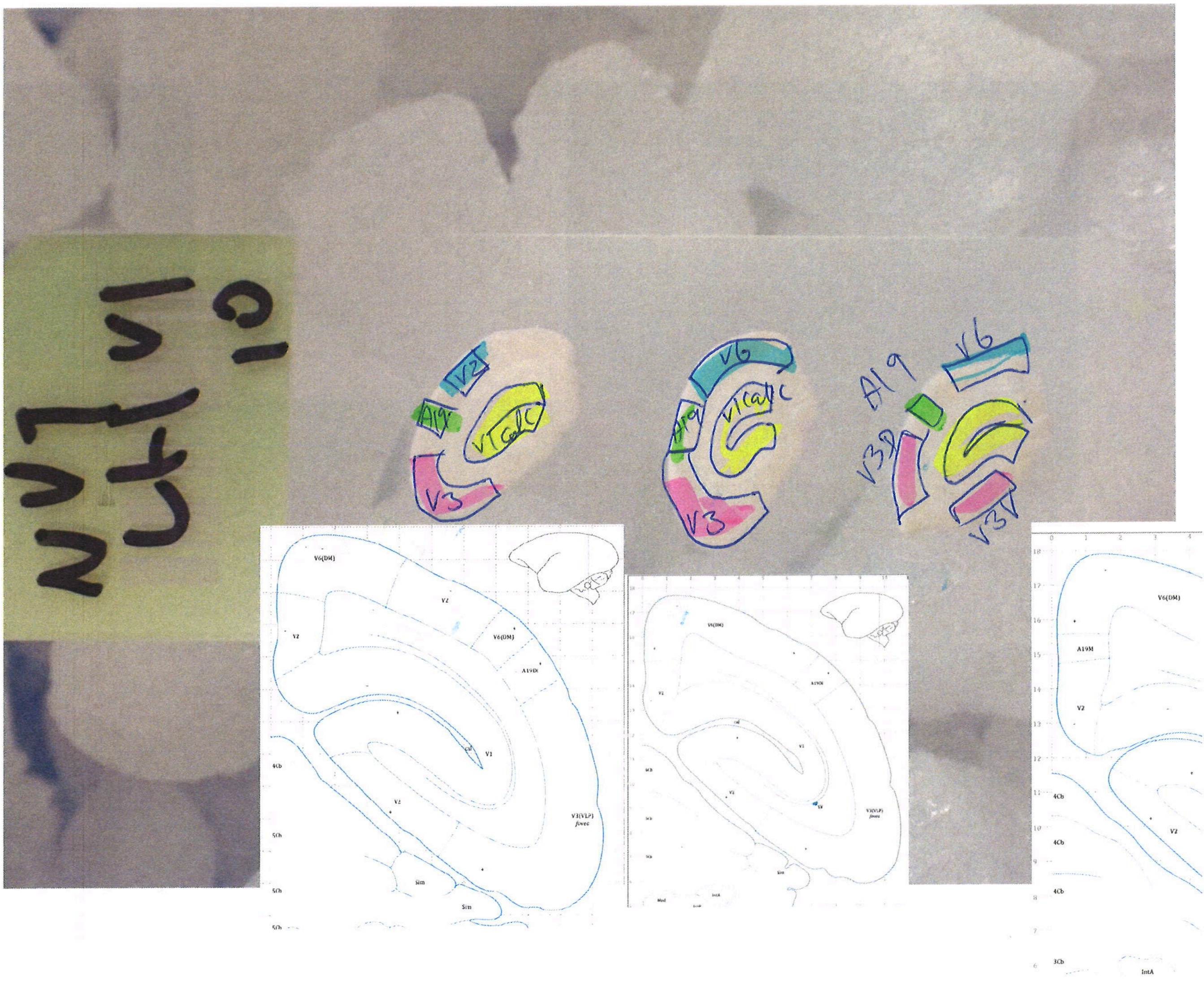
V2
A19
V1CalC
V3
V6
A19
V1CalC
V3
A19
V6
V3D
V3V
V6(DM)
V2
A19VDi
V1
V3(VLP)
fovea
A19M

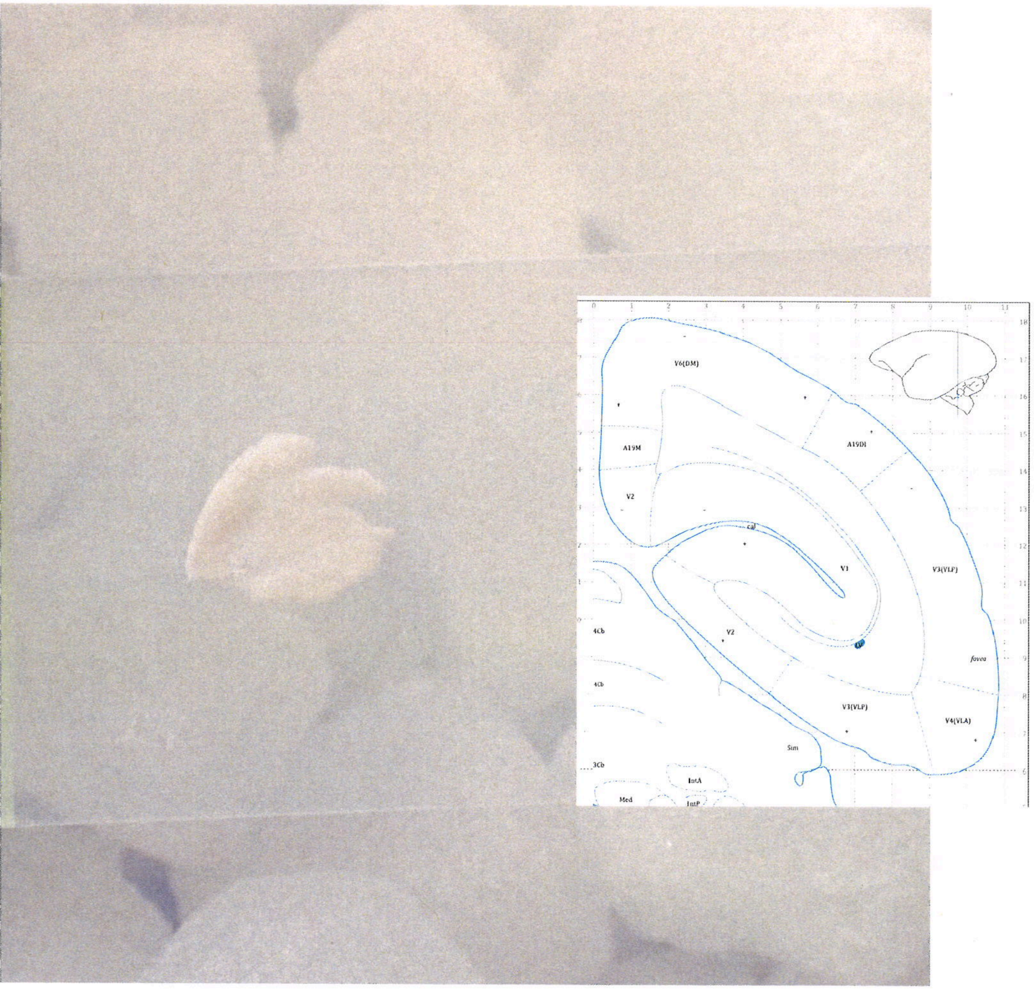
V6(DM)
A19M
A19DI
V2
cal
V1
V3(VLF)
V2
fovea
4Cb
4Cb
V3(VLP)
V4(VLA)
Sim
3Cb
IntA
Med
IntP

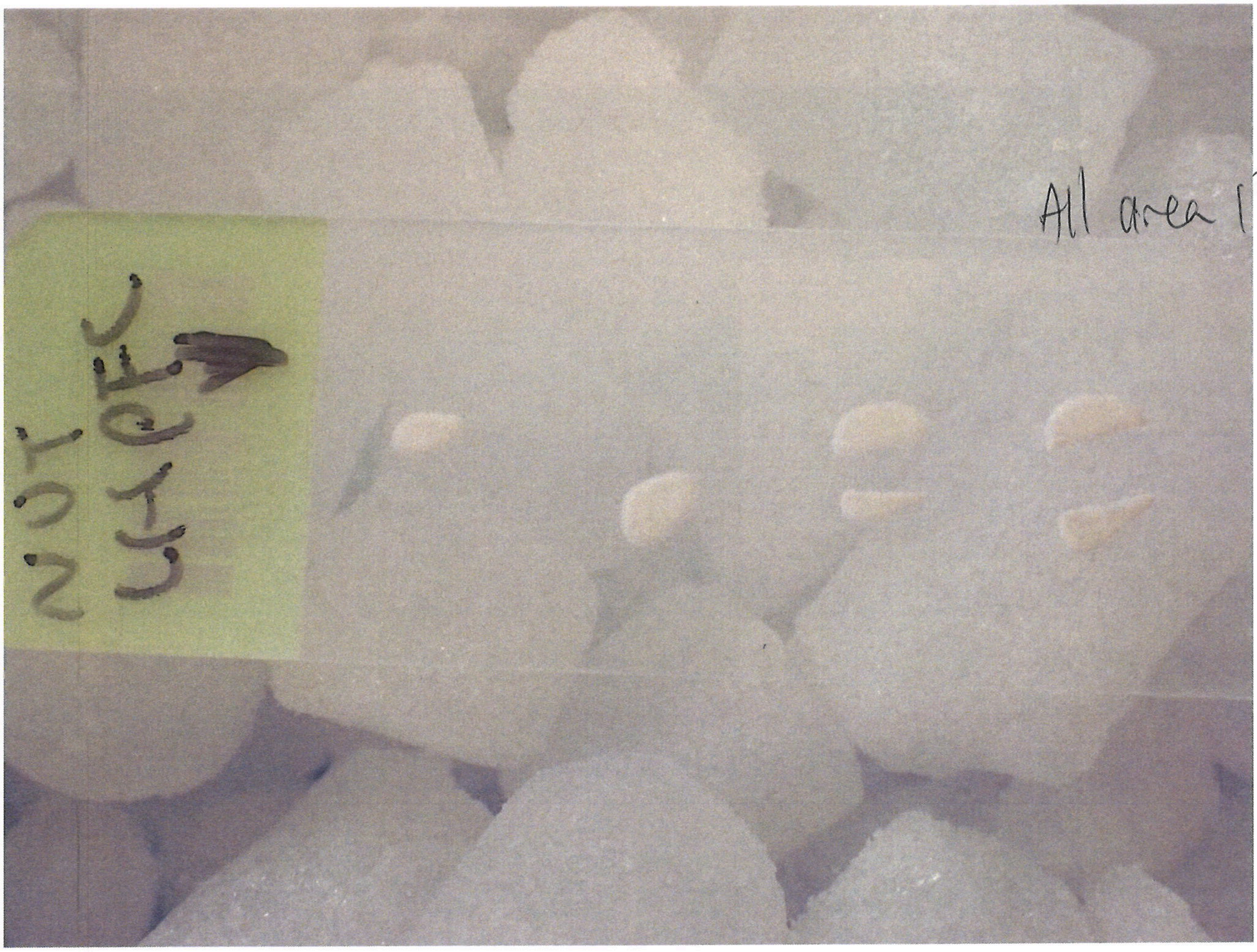

All area 10

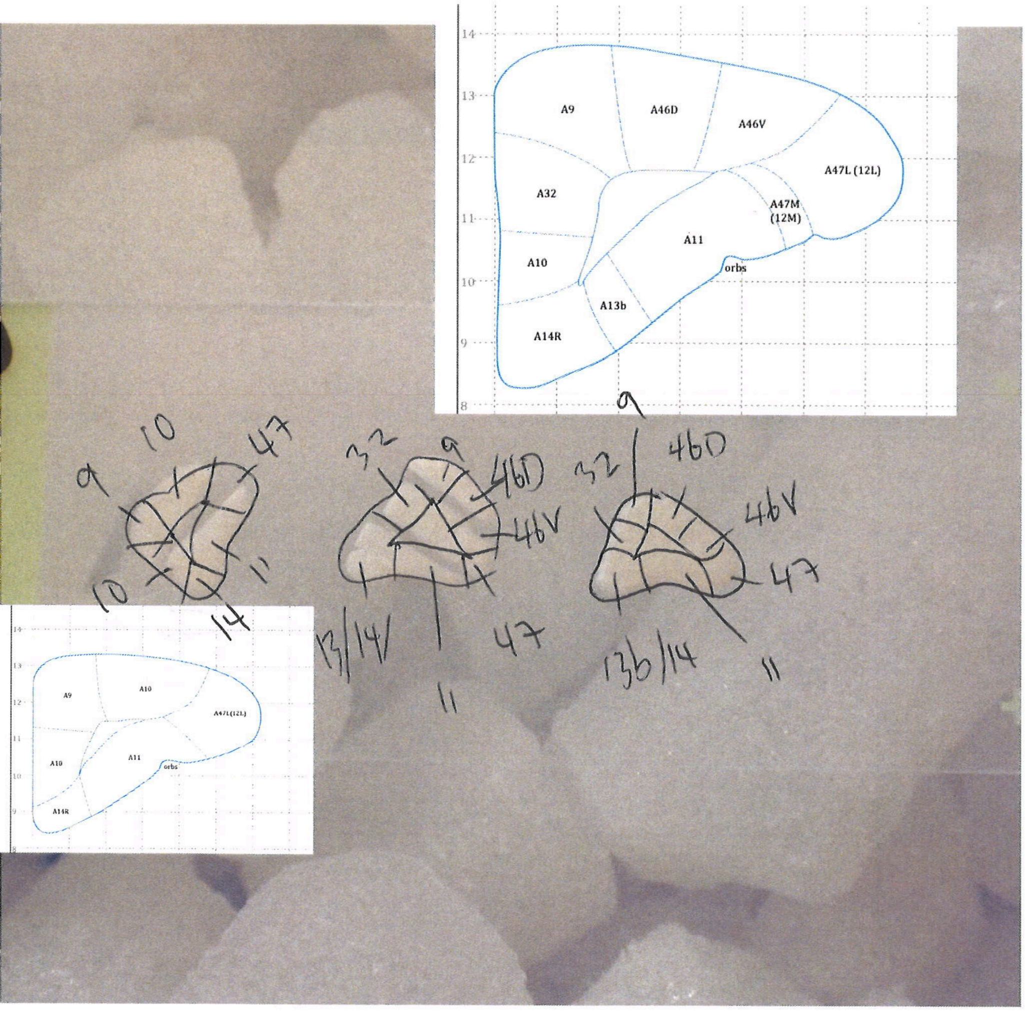
A9
A46D
A46V
A47L (12L)
A32
A47M
(12M)
A11
A10
orbs
A13b
A14R
9
10
47
10
11
14
32
9
46D
46V
13/14
11
47
9
32
46D
46V
47
13b/14
11
A9
A10
A47L (12L)
A10
A11
orbs
A14R

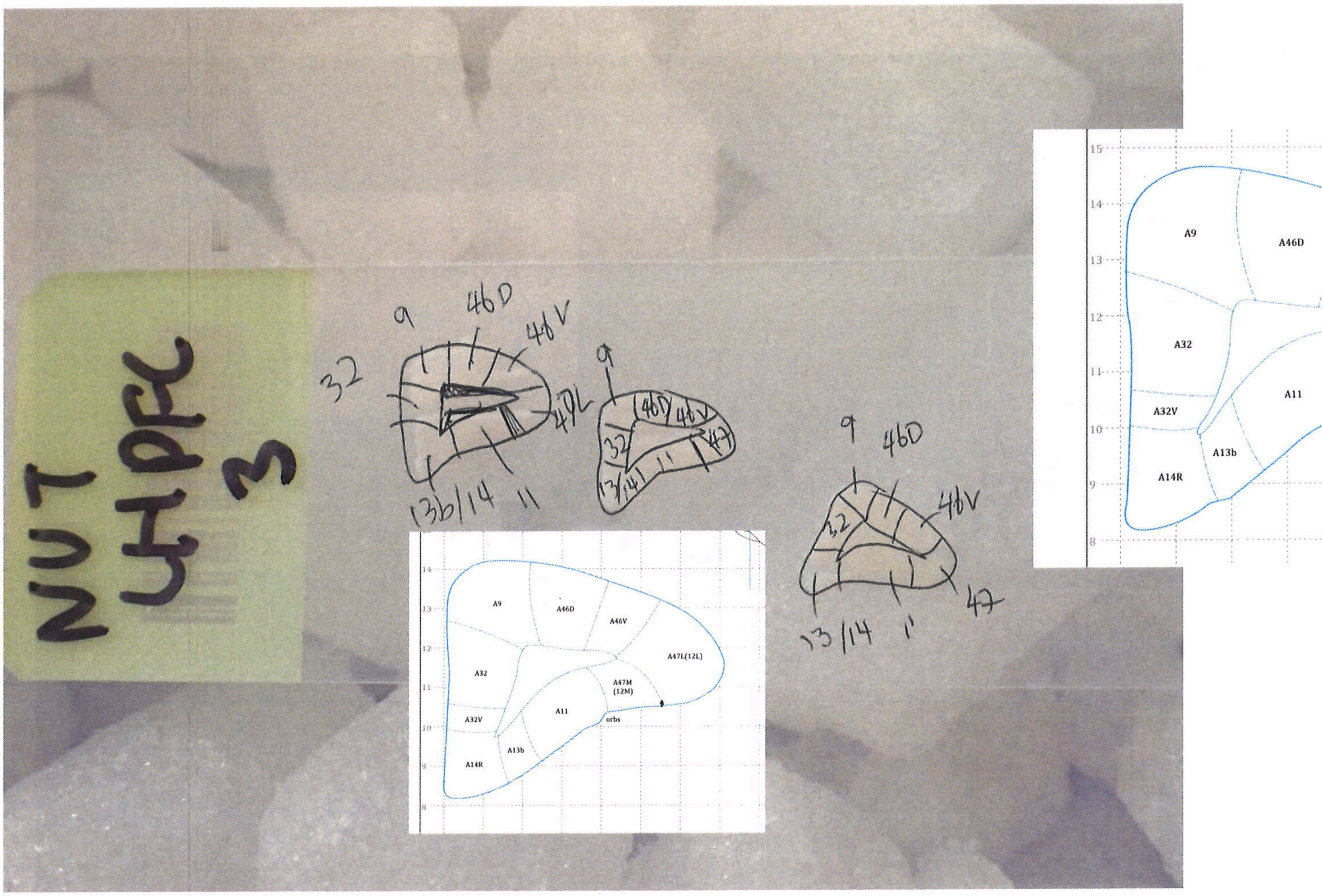

NUT
LHPFC
3
32
9
46D
46V
47L
13b/14
11
9
32
46D
46V
47
13/14
11
9
46D
46V
32
47
13/14
11
A9
A46D
A46V
A47L(12L)
A32
A47M
(12M)
A11
A32V
orbs
A14R
A13b
A9
A46D
A32
A11
A32V
A13b
A14R

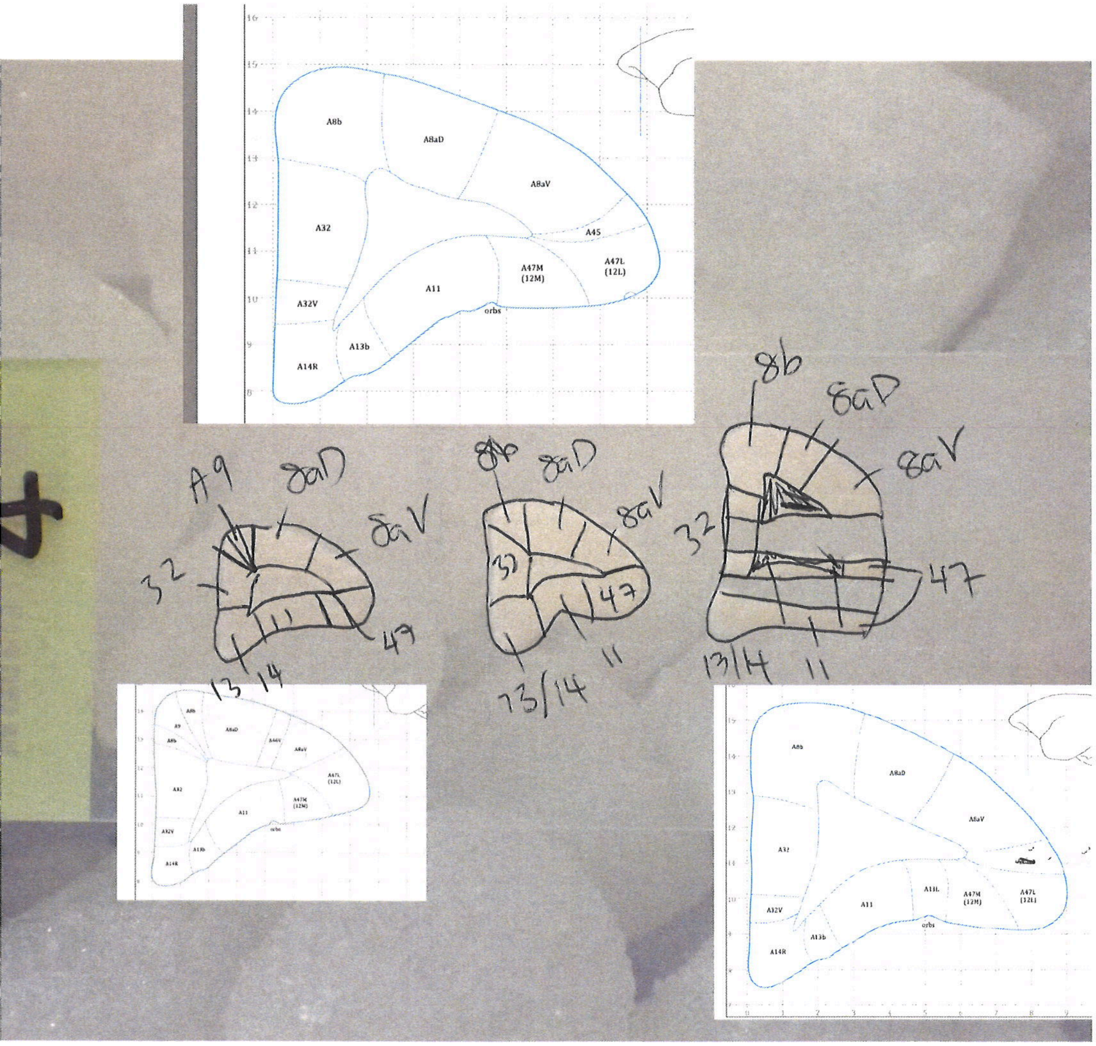
A8b
A8aD
A8aV
A45
A32
A47M
(12M)
A47L
(12L)
A11
A32V
orbs
A13b
A14R
A9
8aD
8aV
32
11
47
13
14
8b
13/14
A11L

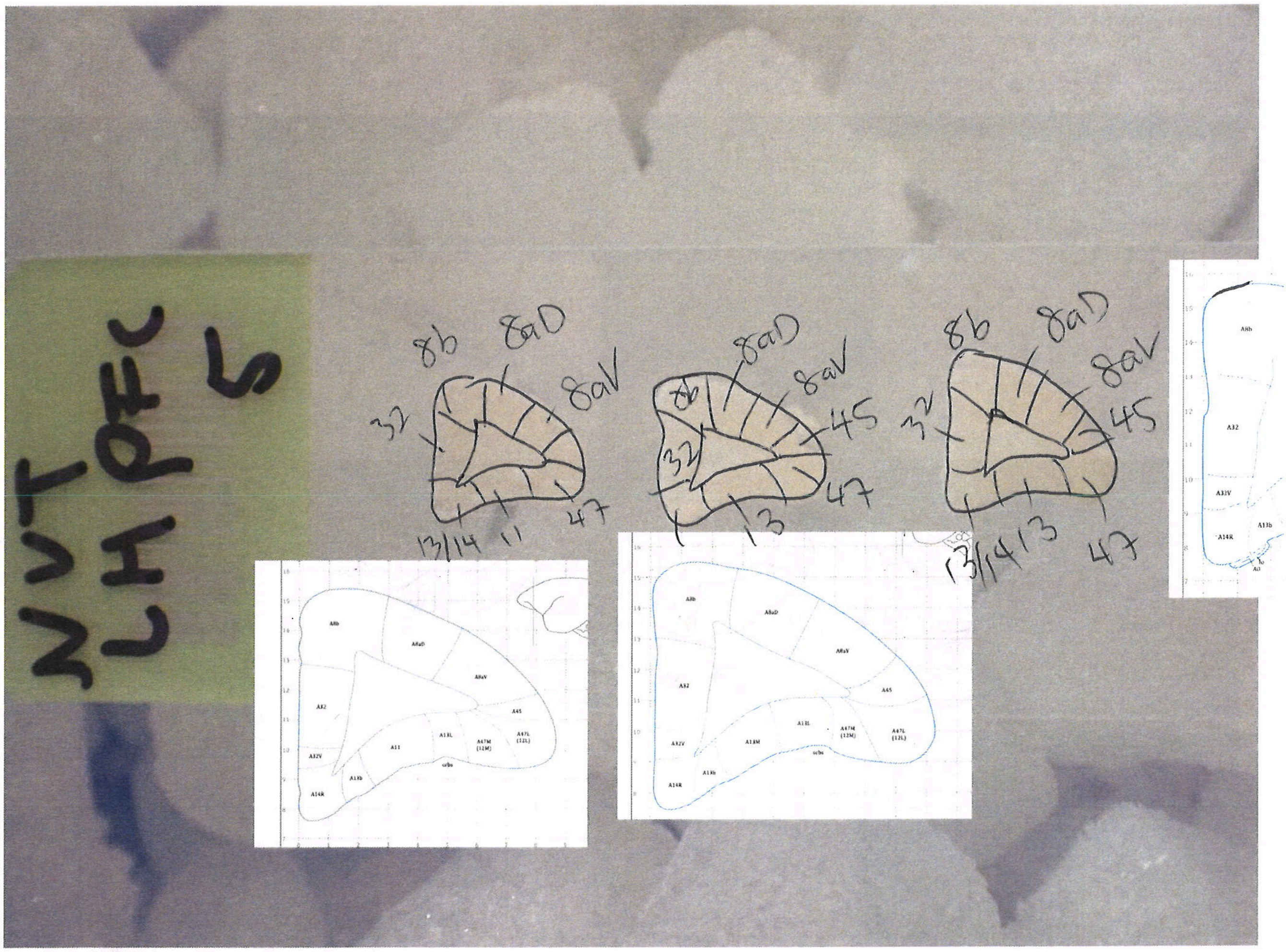
NVT
LH PFC
5
8b
8aD
8aV
32
45
47
13/14
11
13

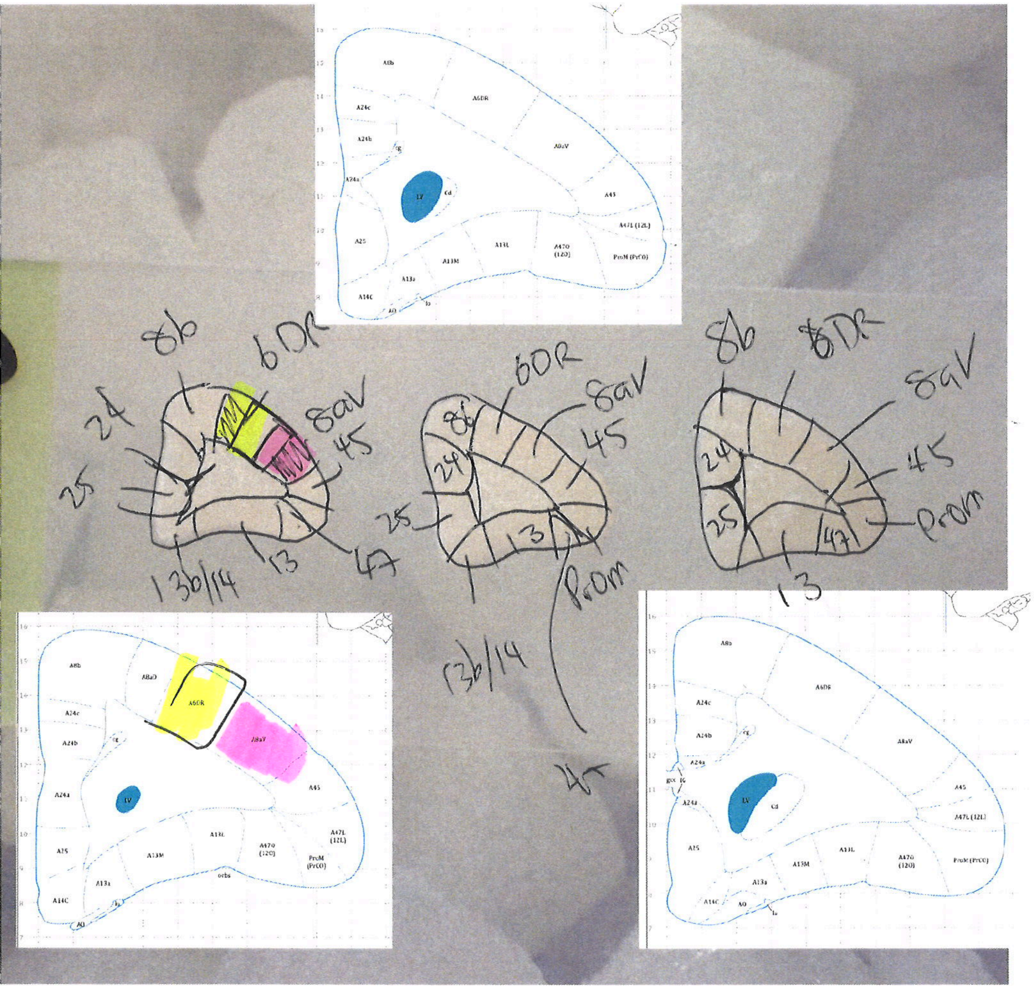
8b
6DR
8aV
45
24
25
13b/14
13
47
Prom

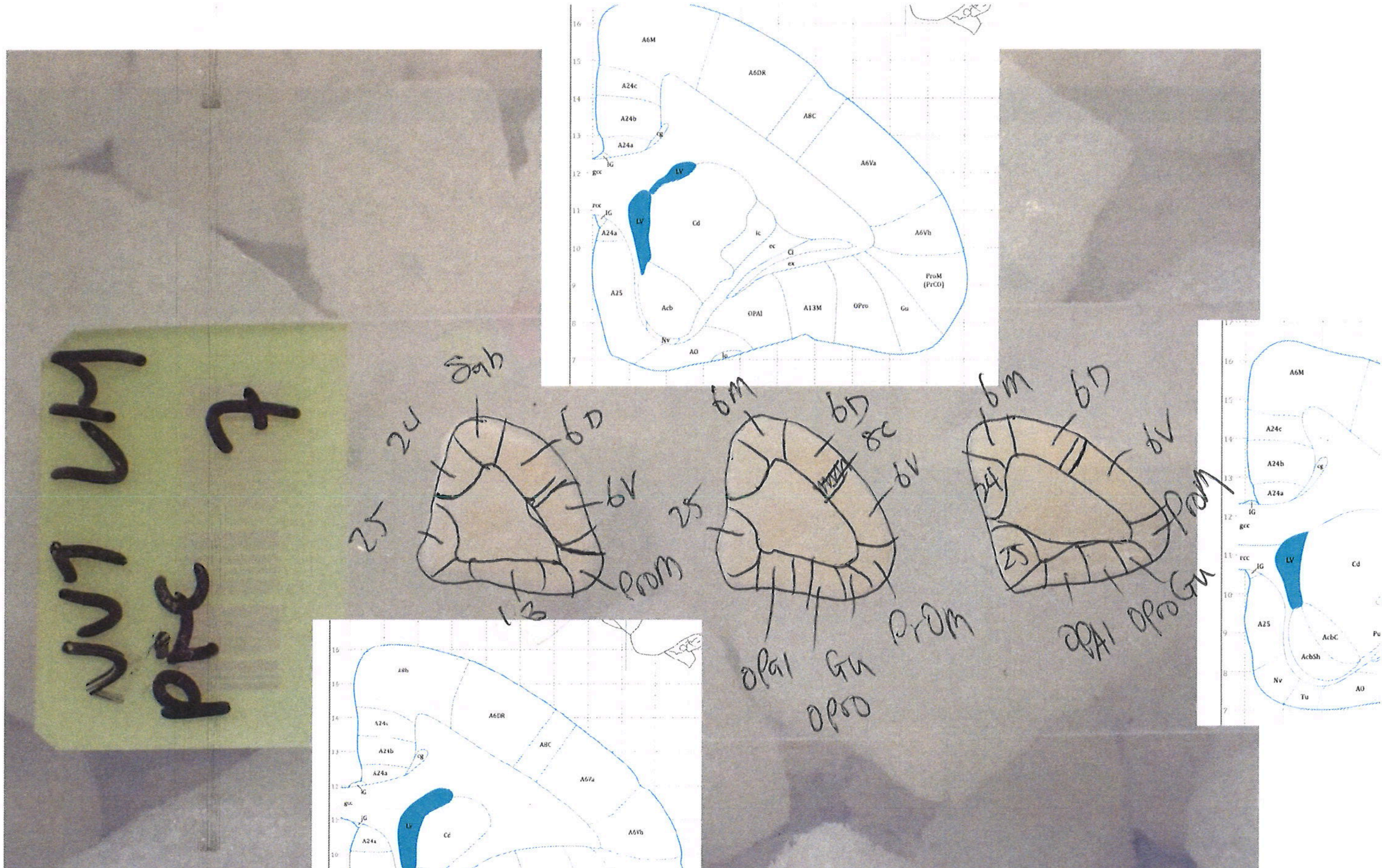

Sab
24
25
6D
6V
ProM
13
6M
8C
OPAl
Gu
OPro

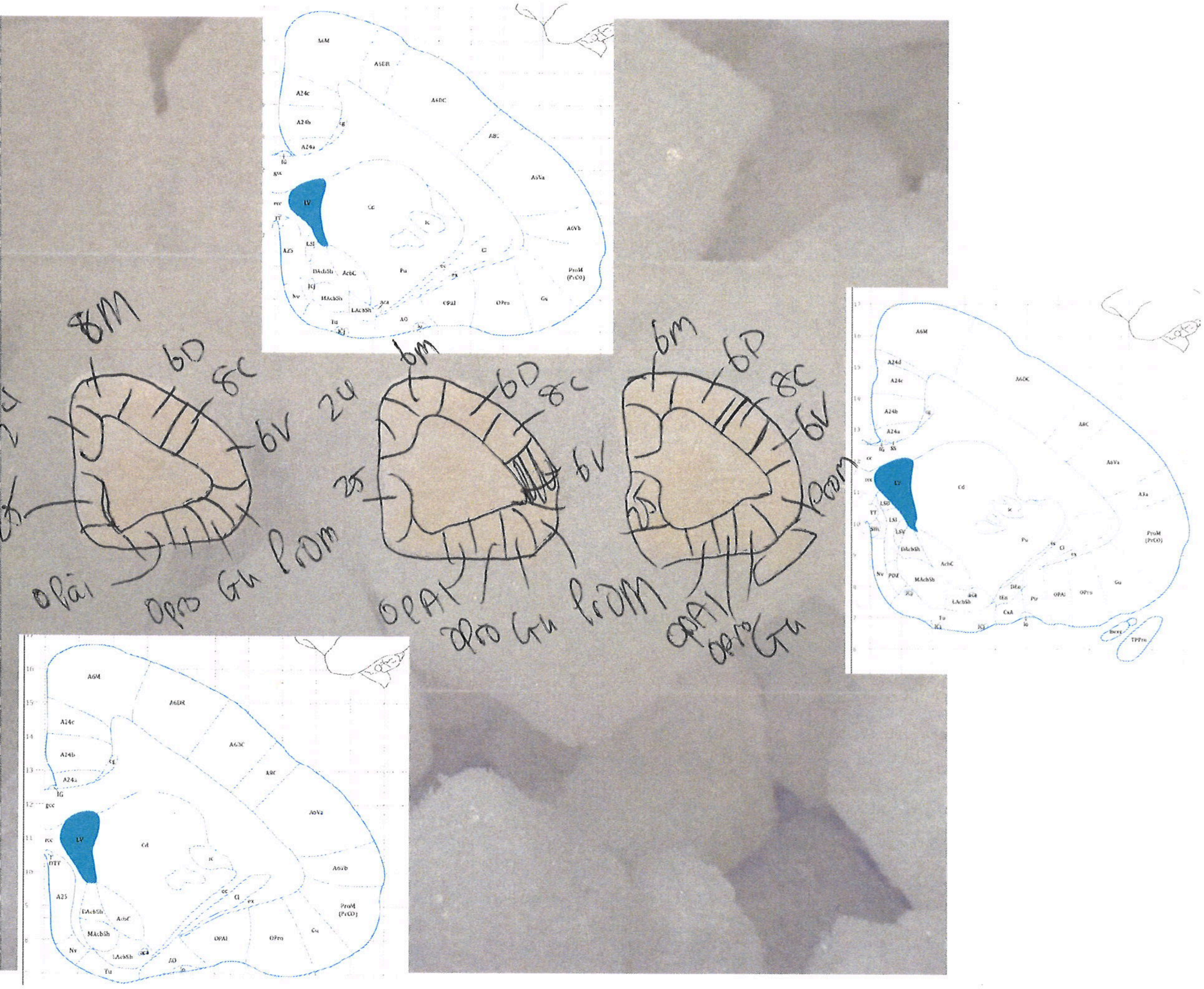

8M
6D
8C
6V
Opai
Opro Gu Prom
6m
6D
8C
6V
25
OPAI
Opro Gu Prom
6m
6P
8C
6V
Prom
OPAI
OPro Gu

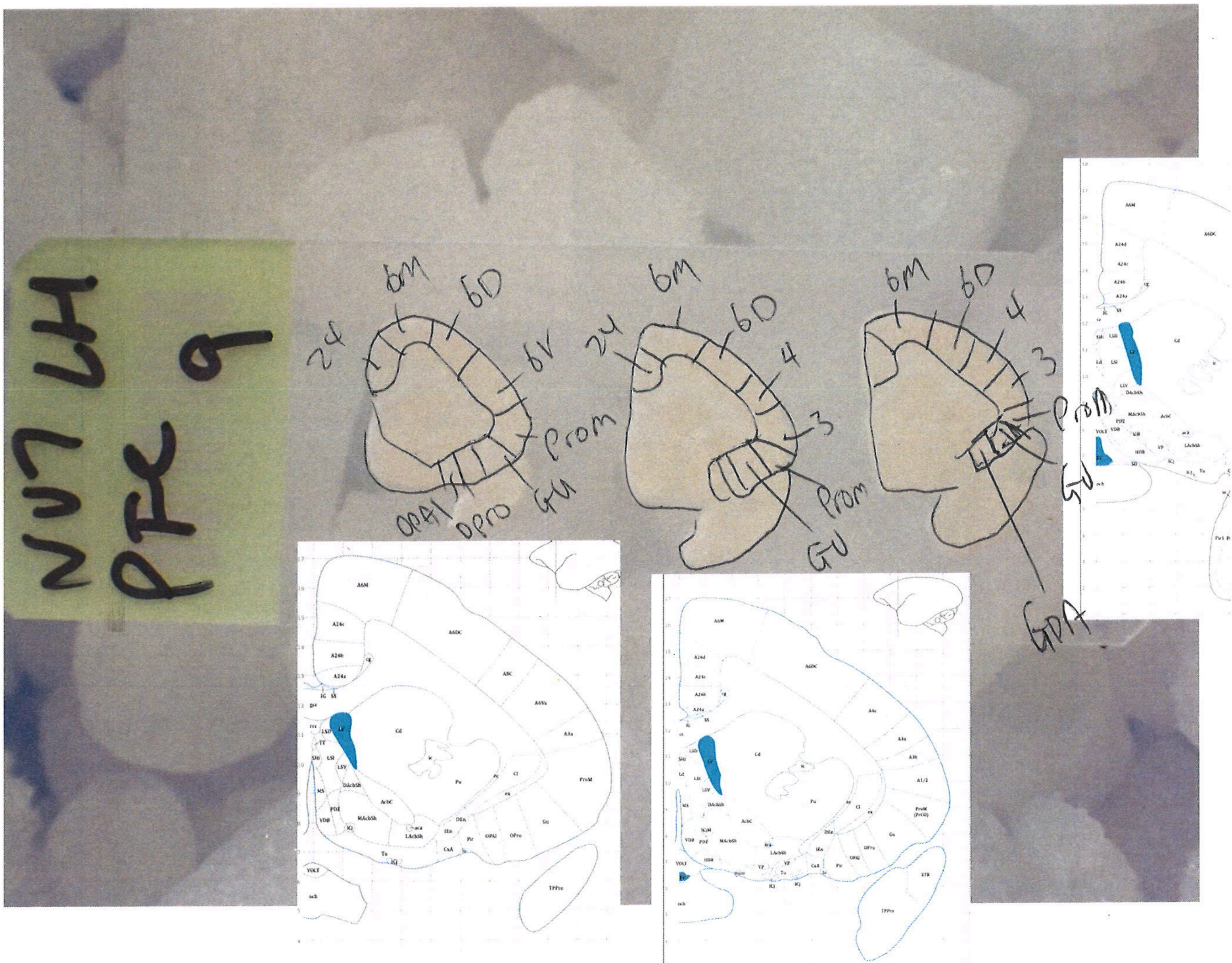
NU7 LH
PFC 9
24
6M
6D
6V
Prom
GU
OPAI
OPro
4
3
GPA

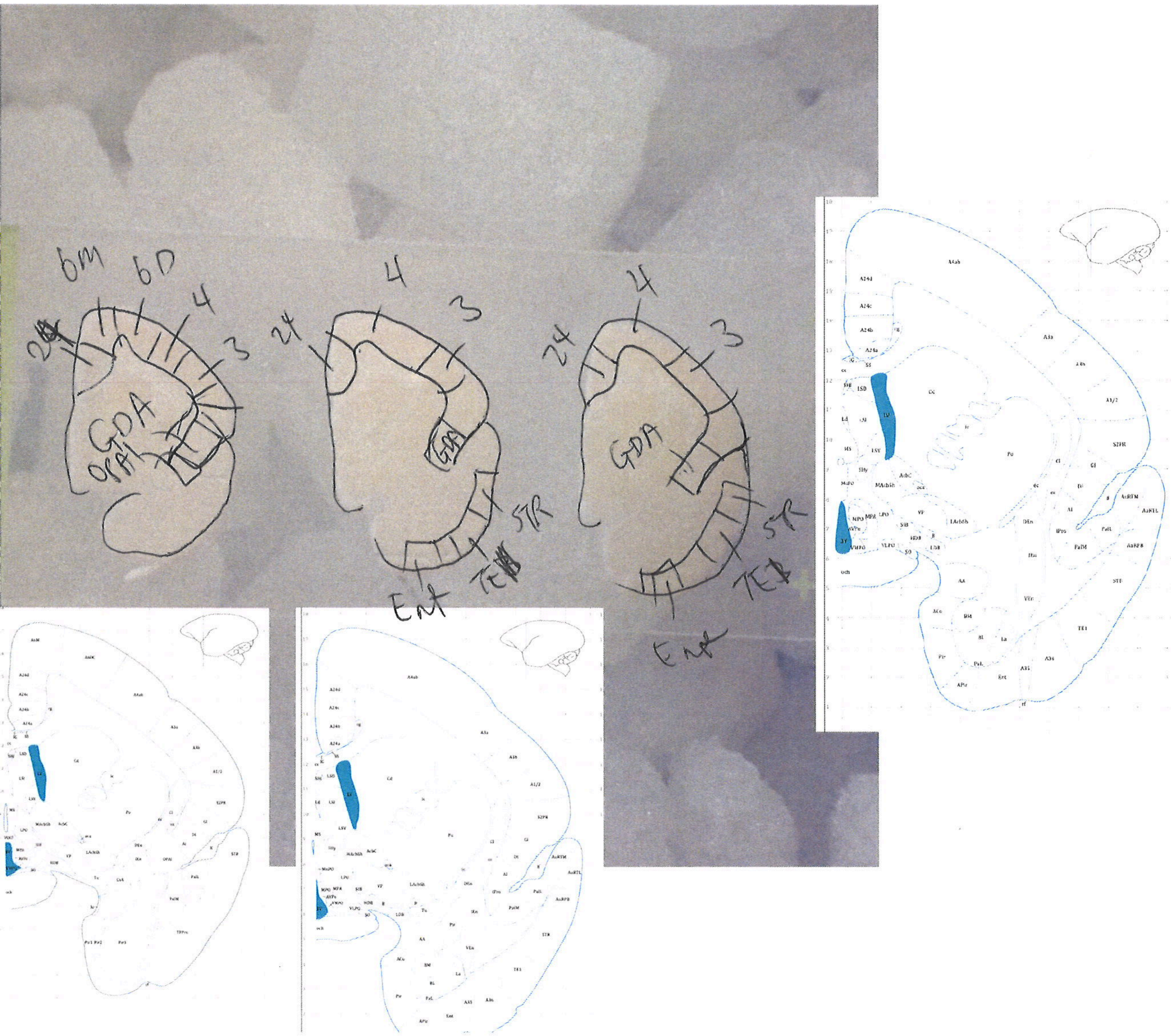

6M
6D
4
3
24
GDA
4
3
24
GDA
STR
TE
Ent
24
4
3
GDA
STR
TE
Ent

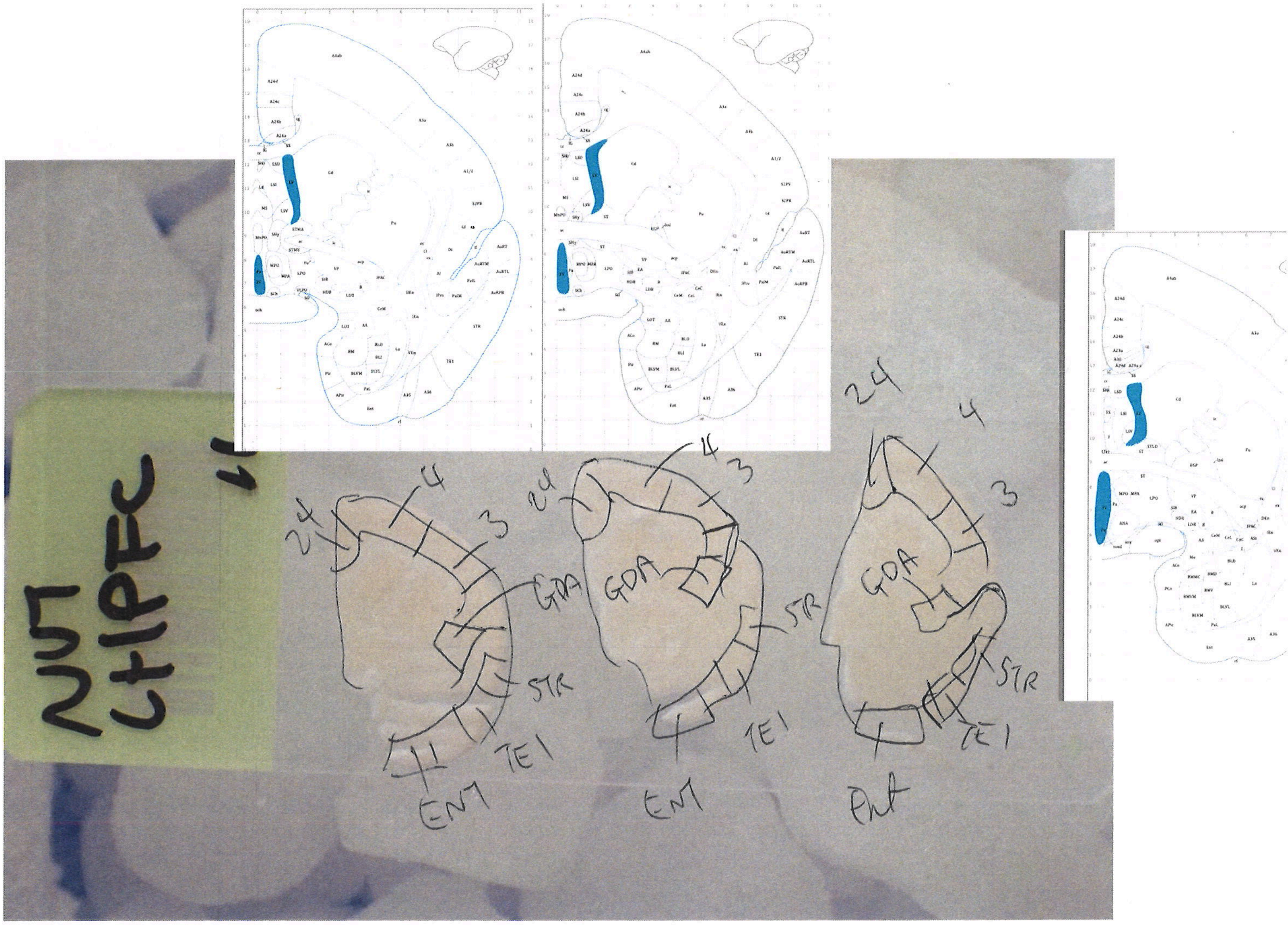
NUT
CHPFC
24
4
3
GDA
STR
TE1
ENT
PIR

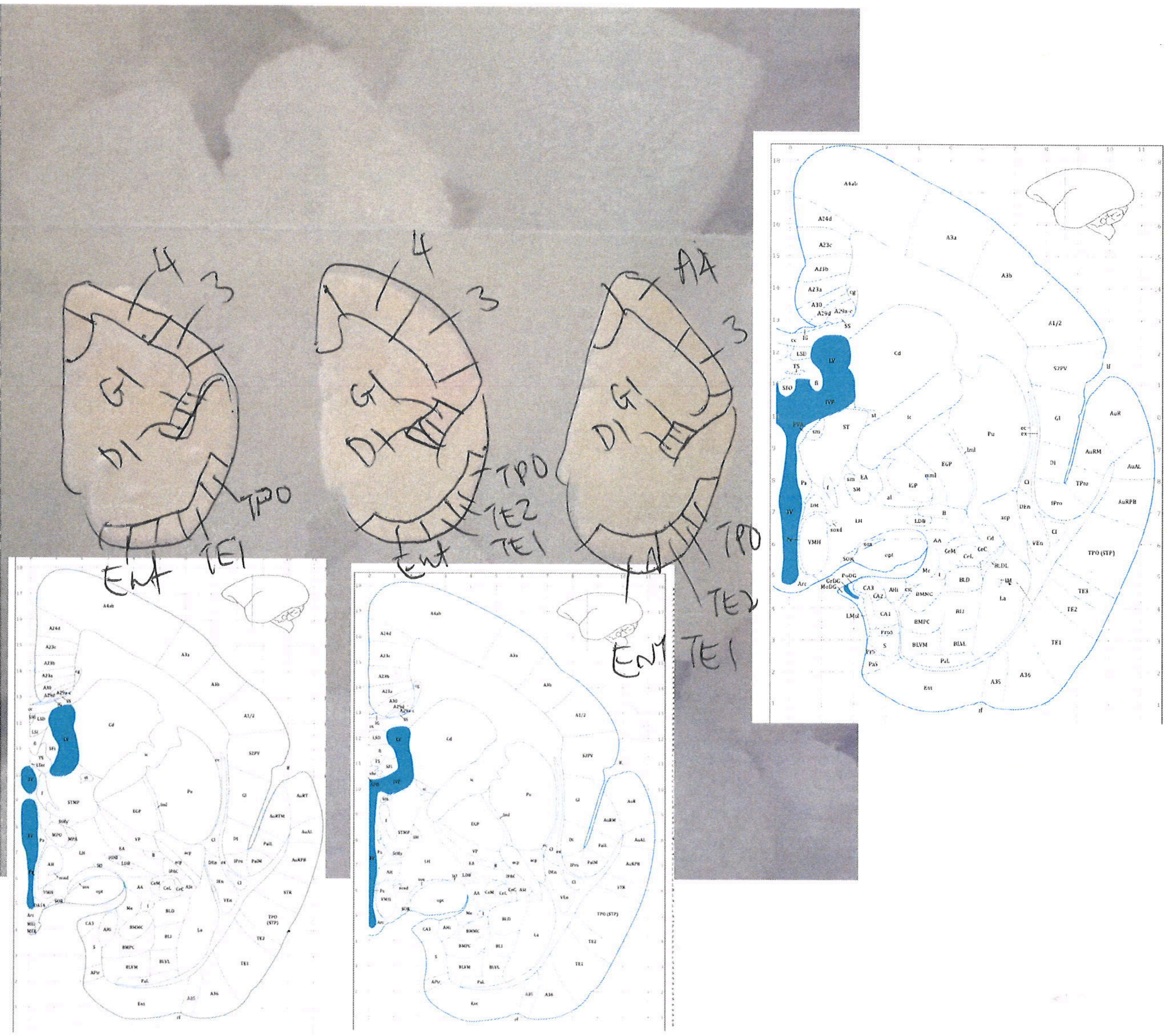
4
3
GI
DI
TPO
TE1
Ent
TE2
A4

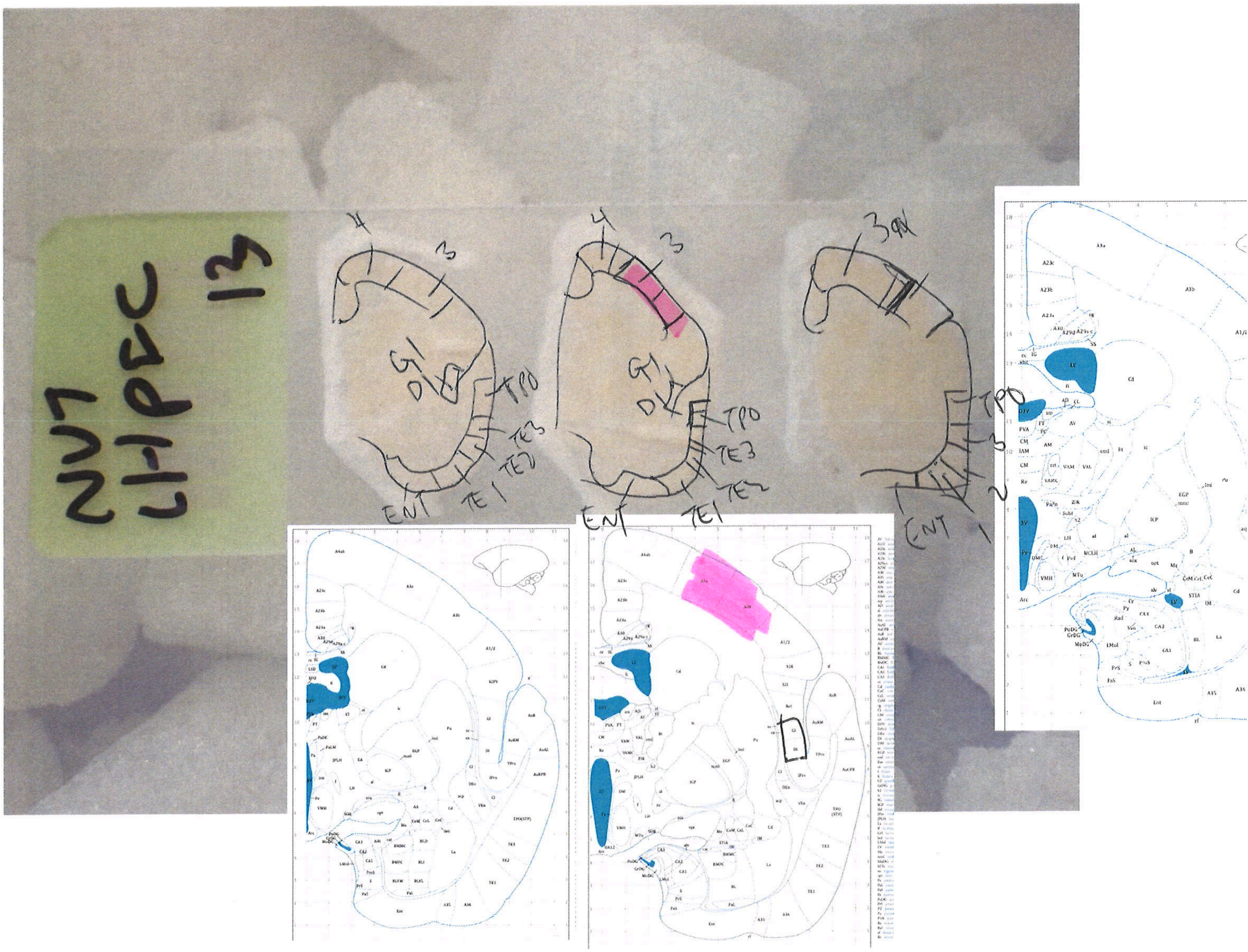
NV7
LHPEC
13
ENT
TE1
TE2
TE3
TPO
GI
DI

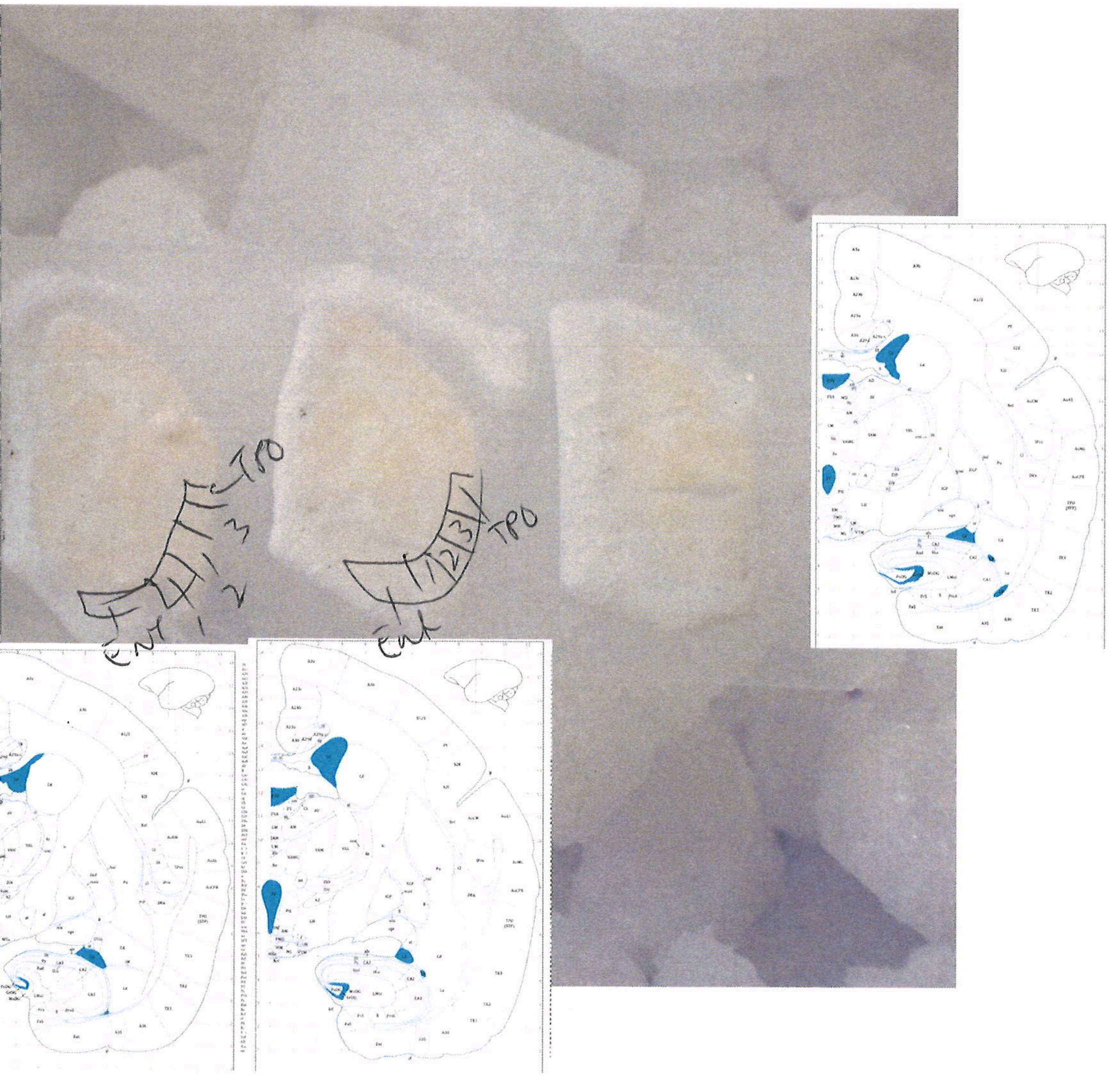
TPO
Ent
1
2
3
TPO
Ent
1
2
3

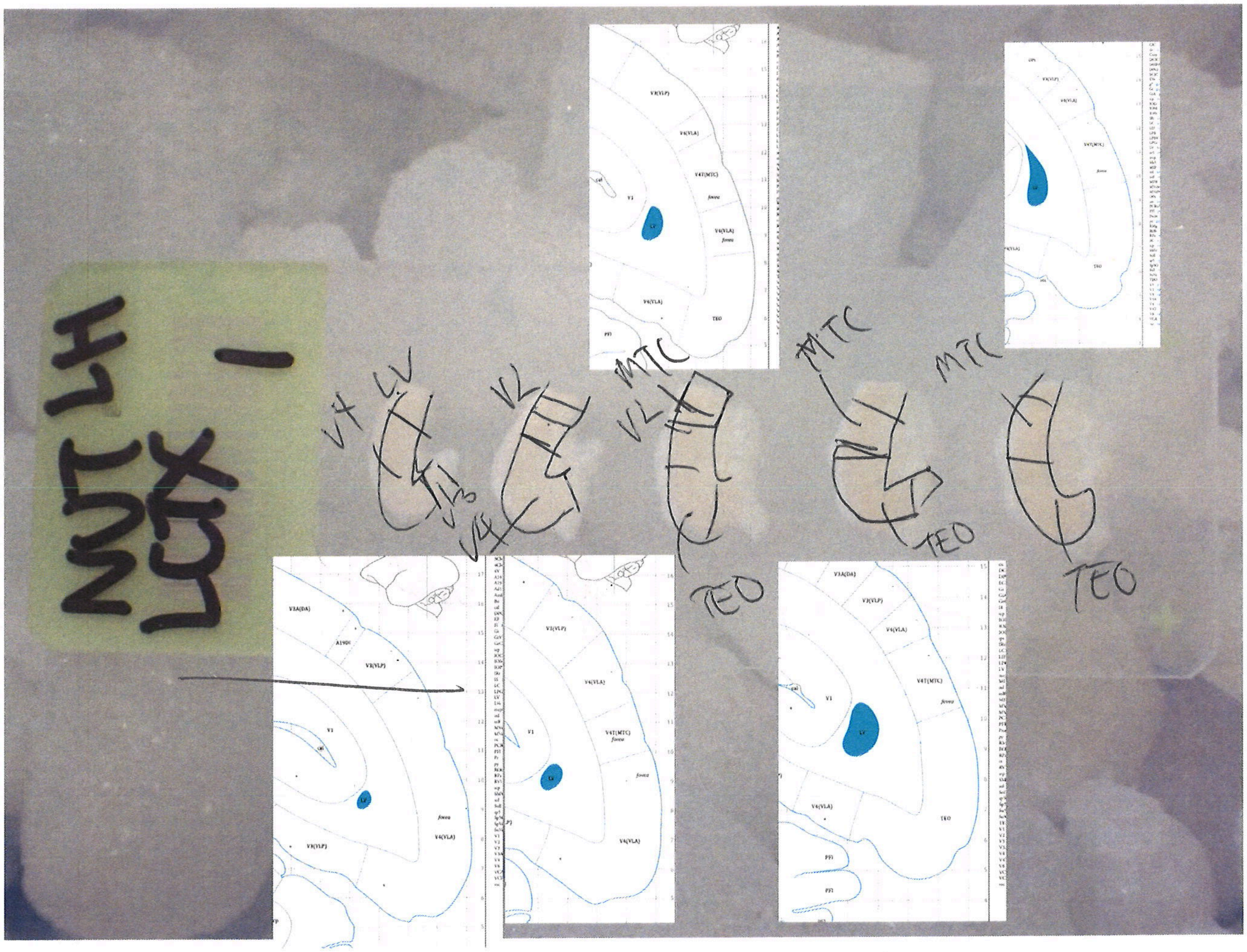
NUT LH
LCTX
1
V4
LV
V3
V2
V4
MTC
VL
TEO
MTC
TEO
MTC
TEO

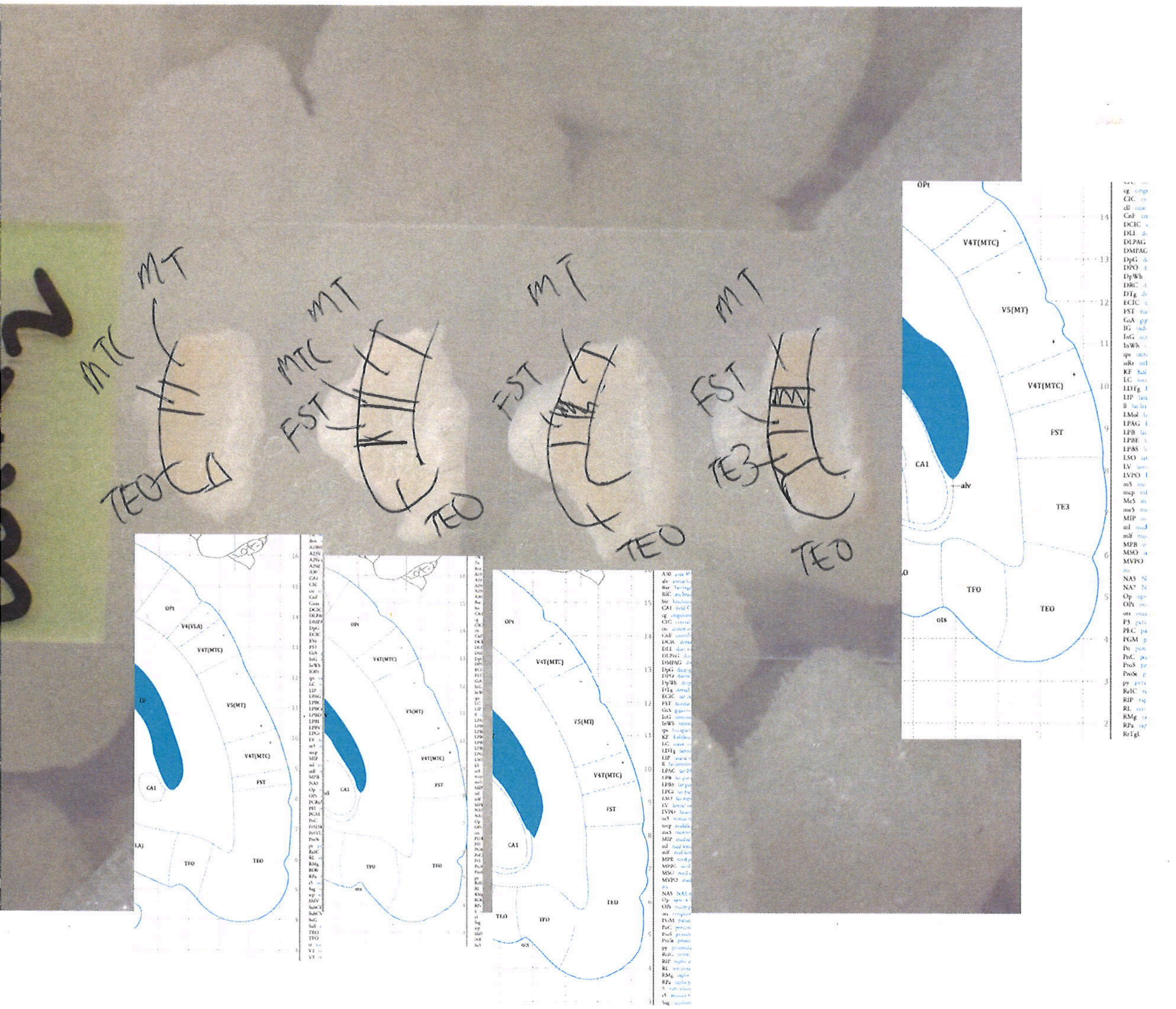
MT
MTC
FST
TEO
TE3
OPt
V4(VLA)
V4T(MTC)
V5(MT)
CA1
alv
TFO
ots

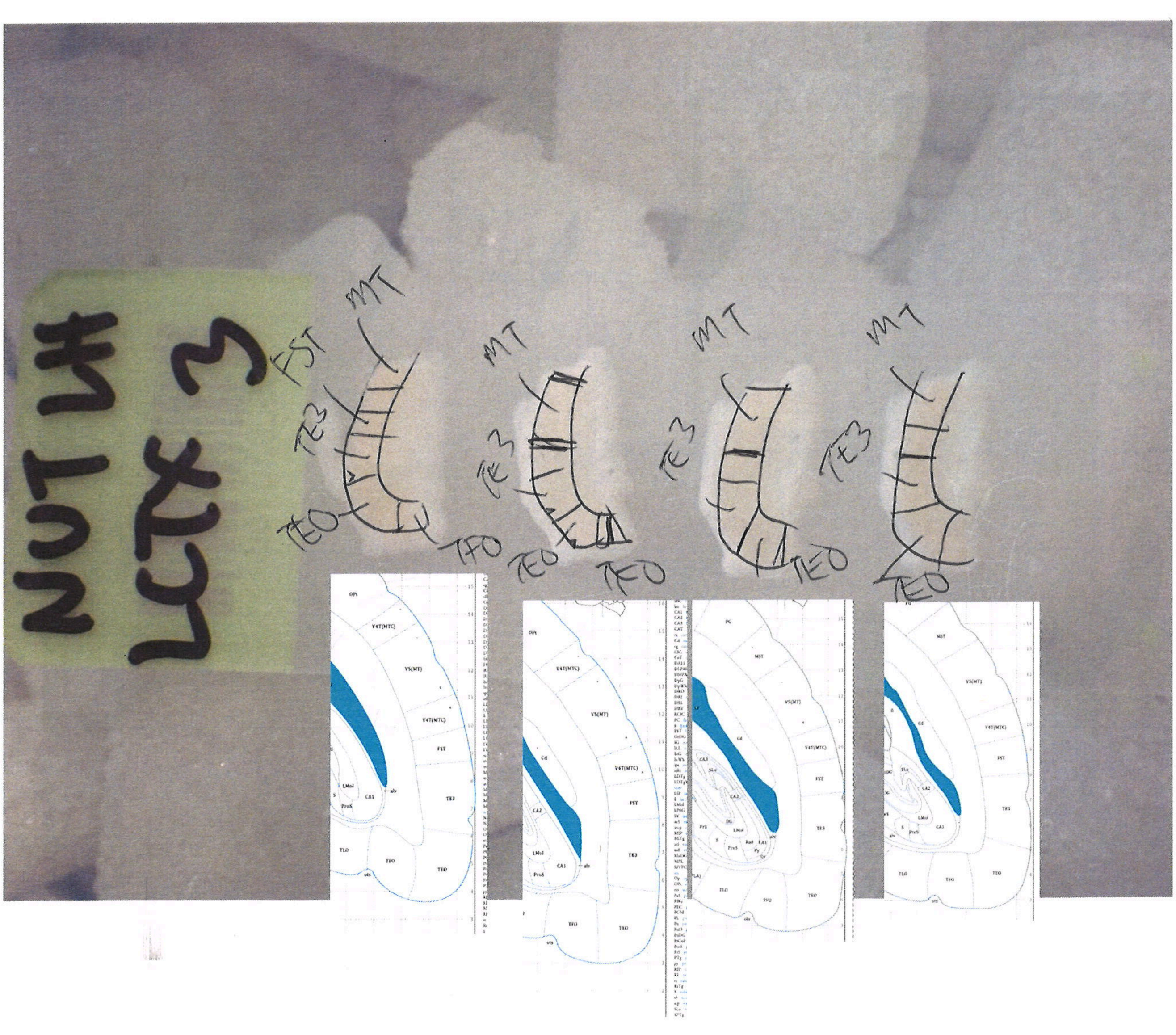
NUT LH
LCTX 3
FST
MT
TE3
TEO
TFO
MT
TE3
TEO
TEO
MT
TE3
TEO
MT
TE3
TEO

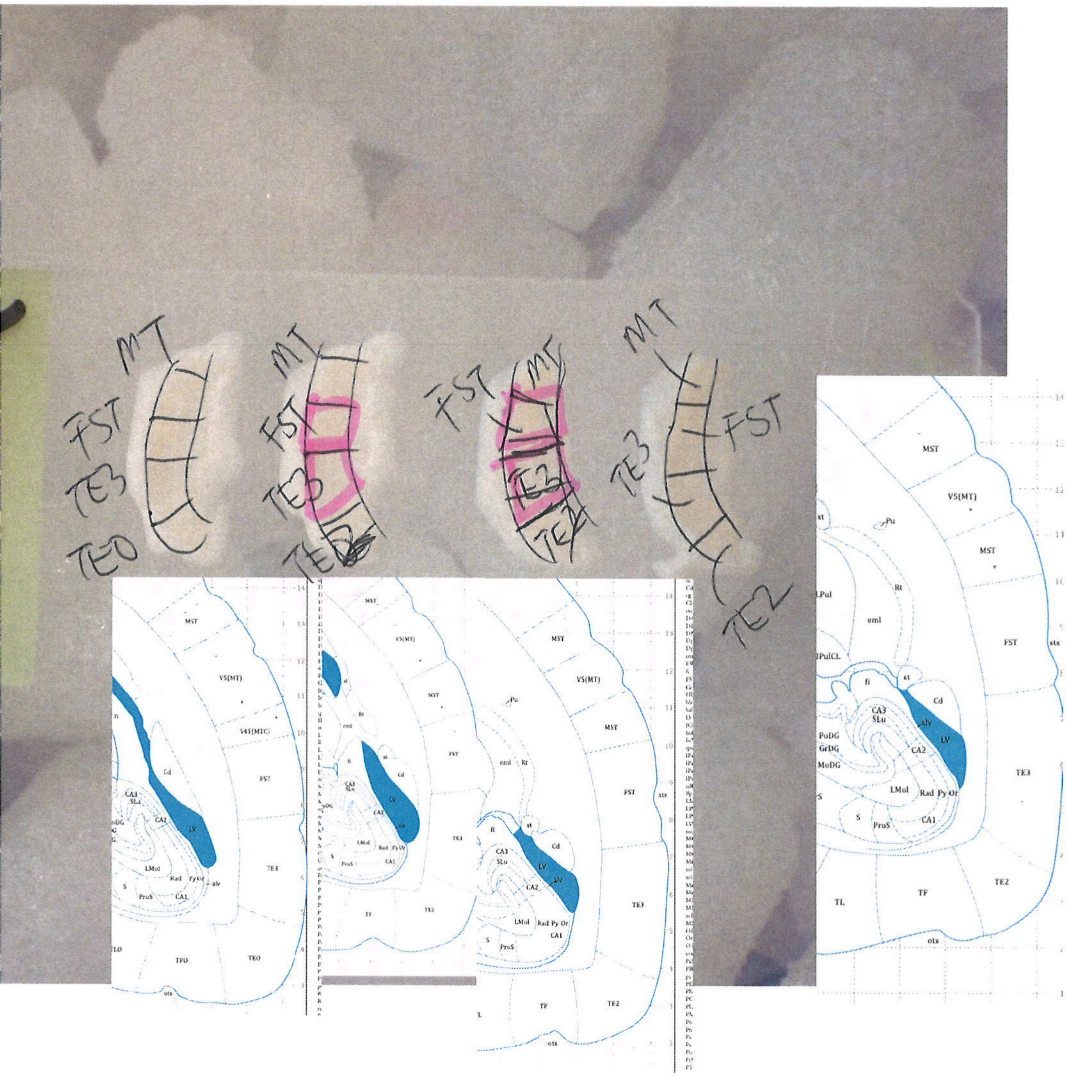
MT
FST
TE3
TEO
TE2
MST
V5(MT)
V4t(MTC)
Cd
LV
CA3
SLu
CA1
CA2
LMol
Rad
Py
Or
alv
S
ProS
TE3
TE2
TF
TL
TFO
TEO
ots
sts
Pu
Rt
eml
st
fi
LPul
LPulCL
PoDG
GrDG
MoDG

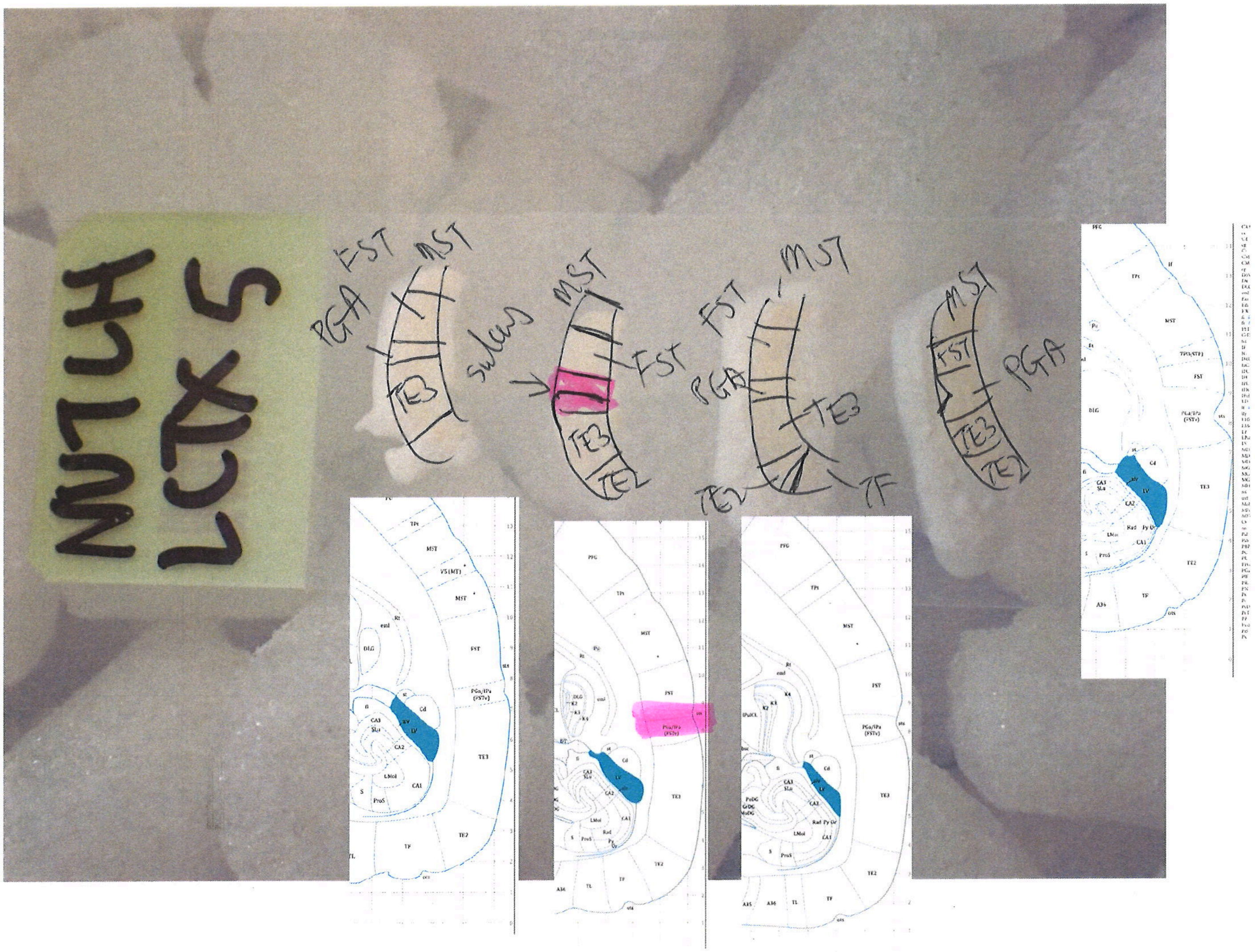
NUTLH
LCTX 5
FST
MST
PGA
TE3
sulcus
TE2
TF

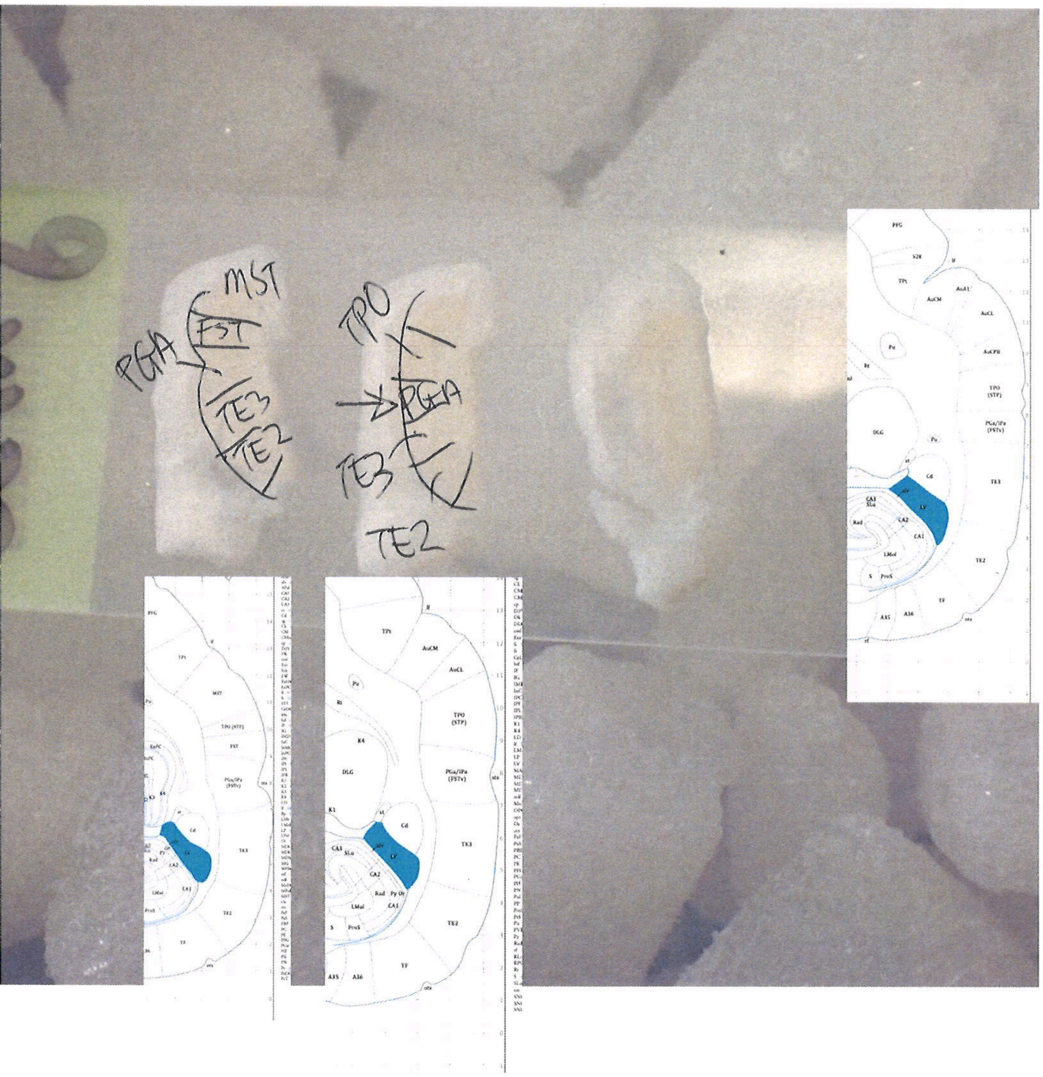
MST
PGA
FST
TE3
TE2
TPO
PGA
TE3
TE2

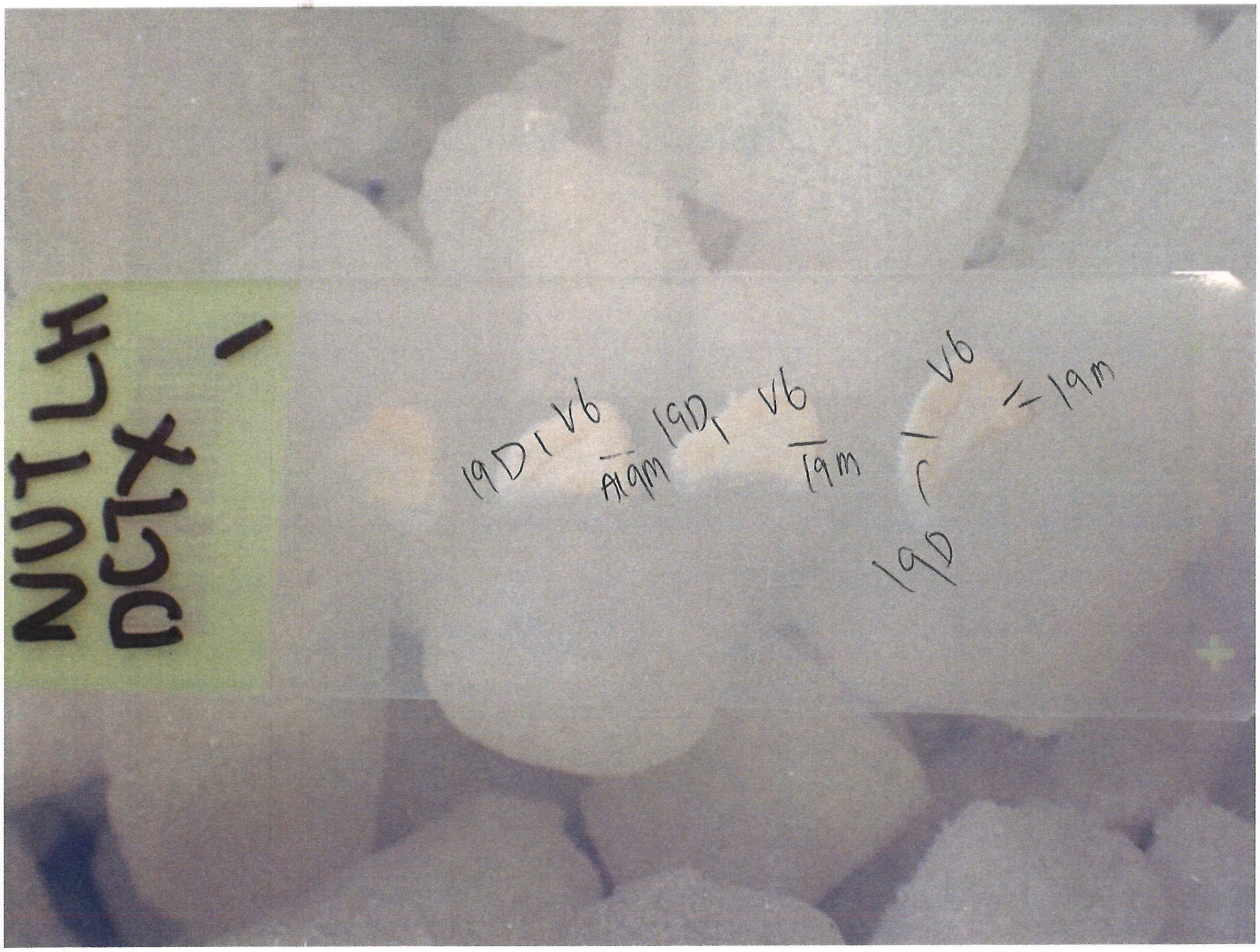
NUT LH
DCTX 1
19D 1 V6 A19m
19D 1 V6 19m
19D V6 19m

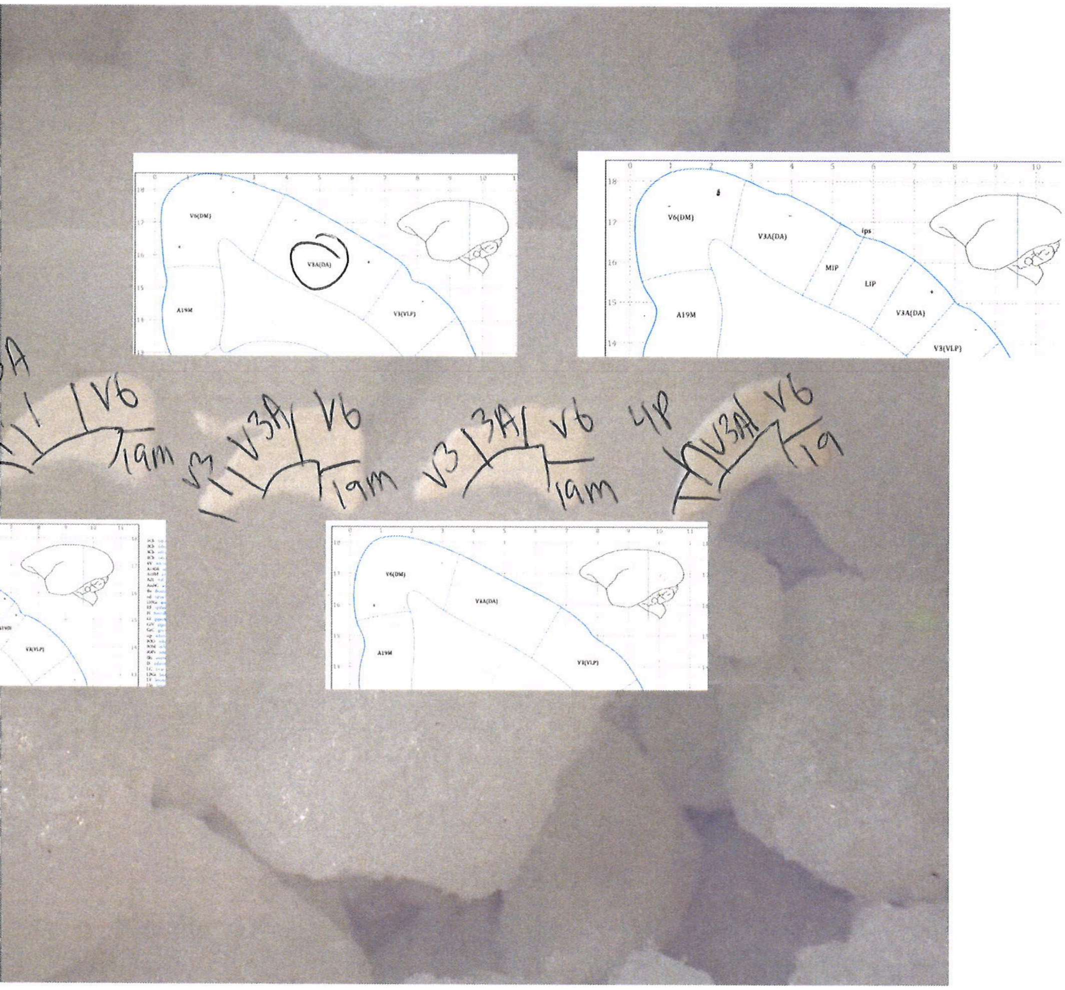

V6(DM)
V3A(DA)
A19M
V3(VLP)
ips
MIP
LIP
V6
V3A
19m
V3
LIP
19

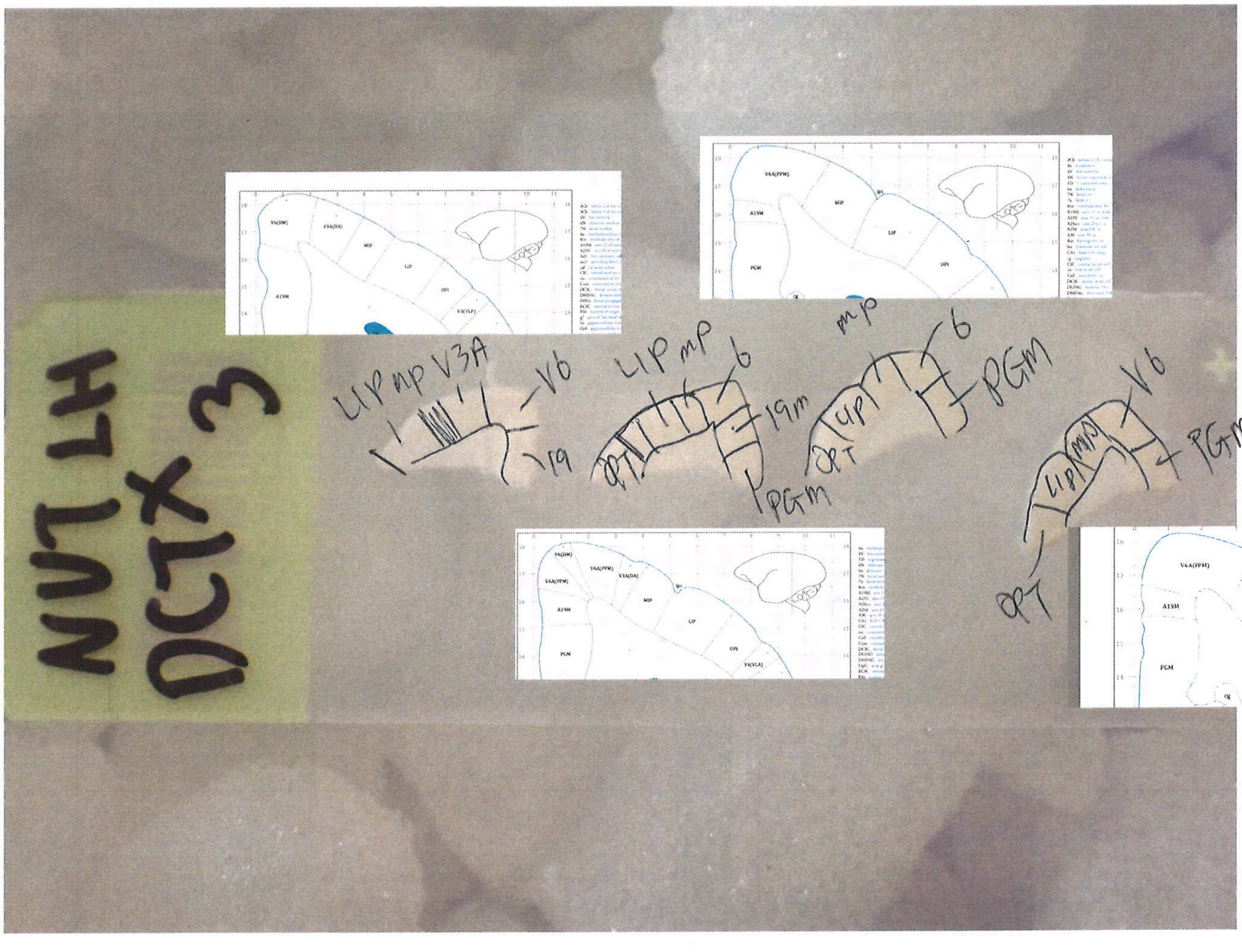
NUT LH
DCTX 3
LIP mp V3A
V6
19
LIP mp
6
19m
PGM
OPT
mp
6
LIP
PGM
OPT
V6
LIP
mp
PGM
OPT

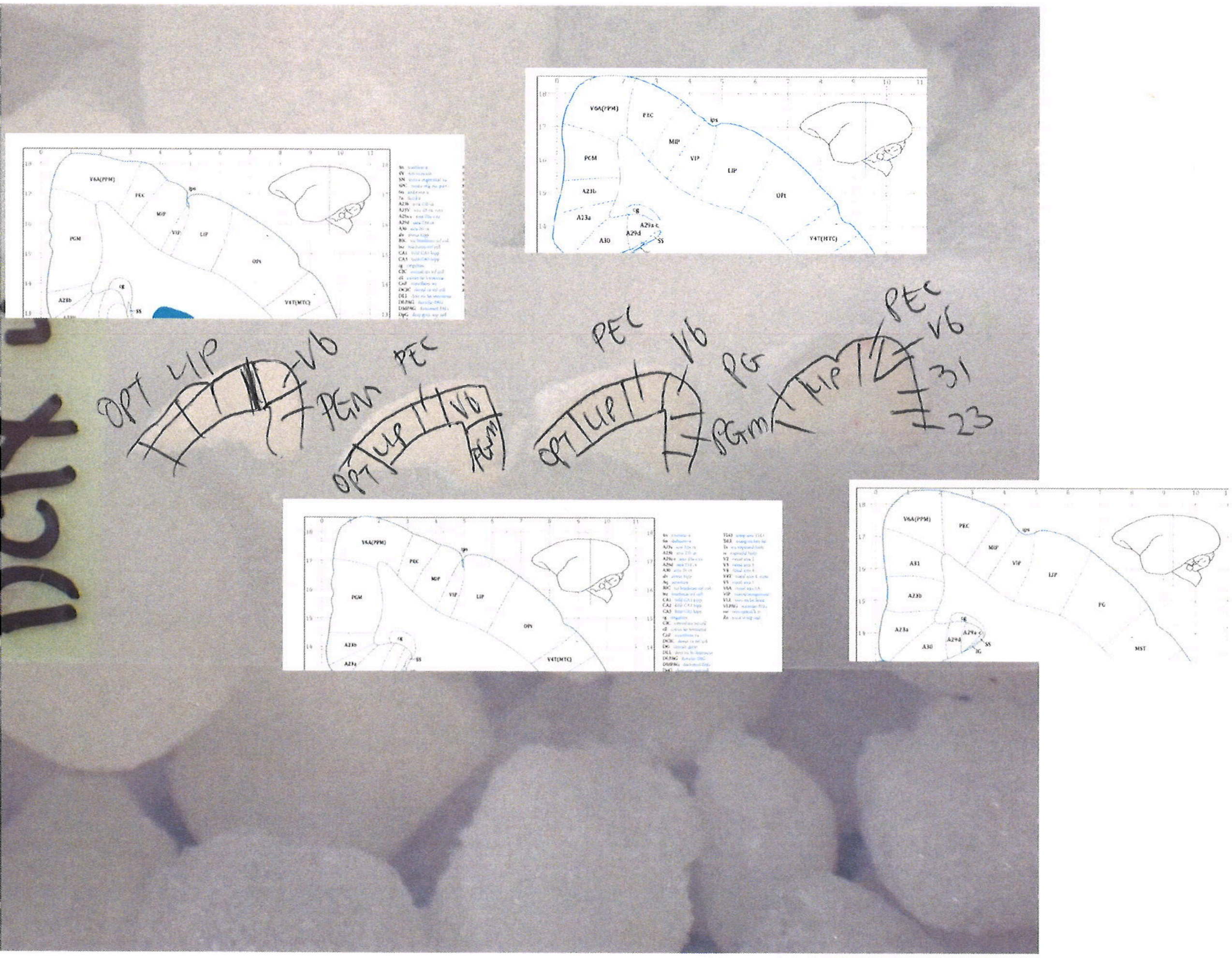

V6A(PPM)
PEC
MIP
VIP
LIP
OPt
PGM
A23a
A30
V4T(MTC)
PG
MST
A31
A23b
OPT
PGm
V6
31
23

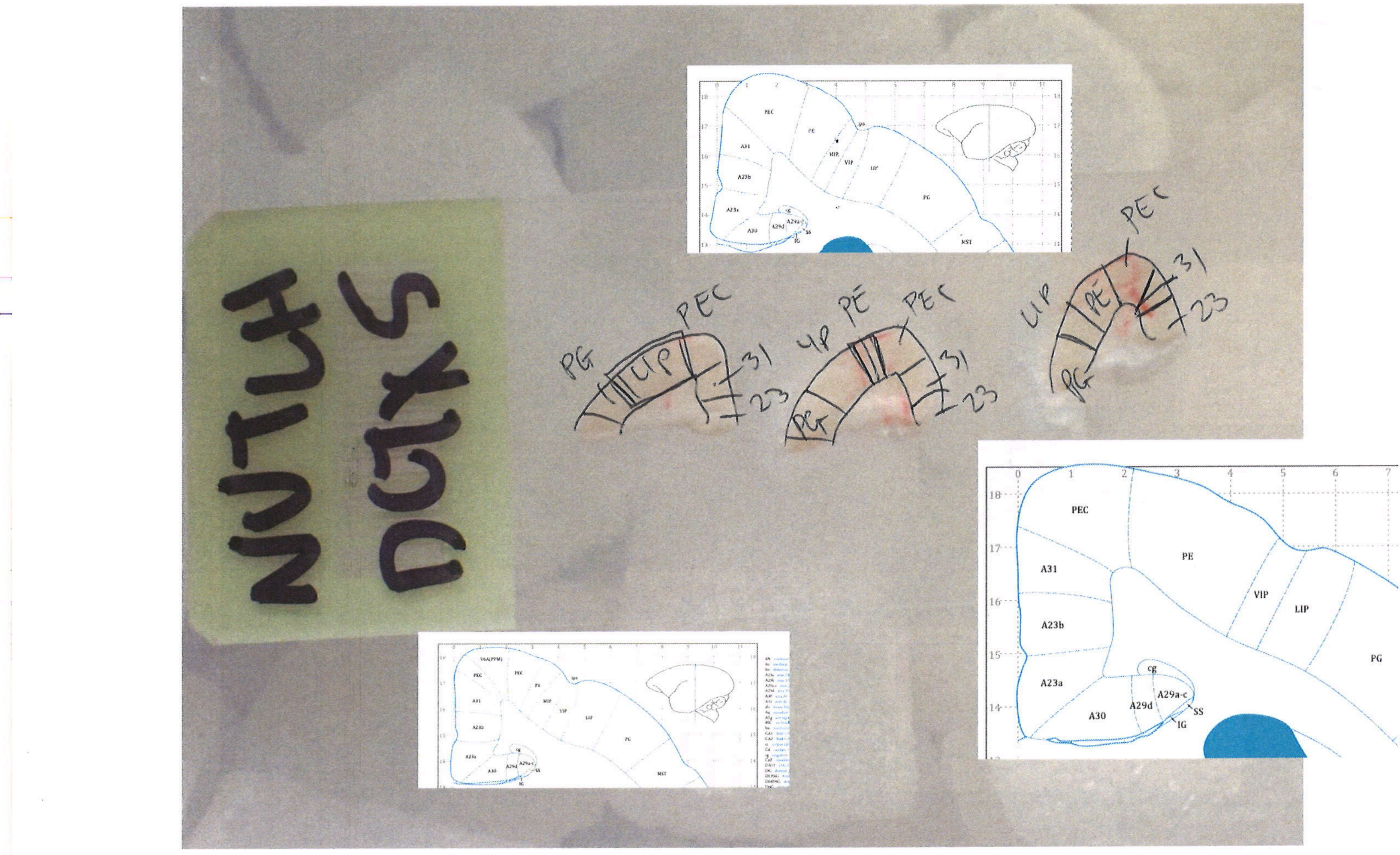

NUTLH
DG75
PG
LIP
PEC
31
23
VIP
PE
PEC
A31
A23b
A23a
A30
A29d
A29a-c
cg
SS
IG
VIP
LIP
PG

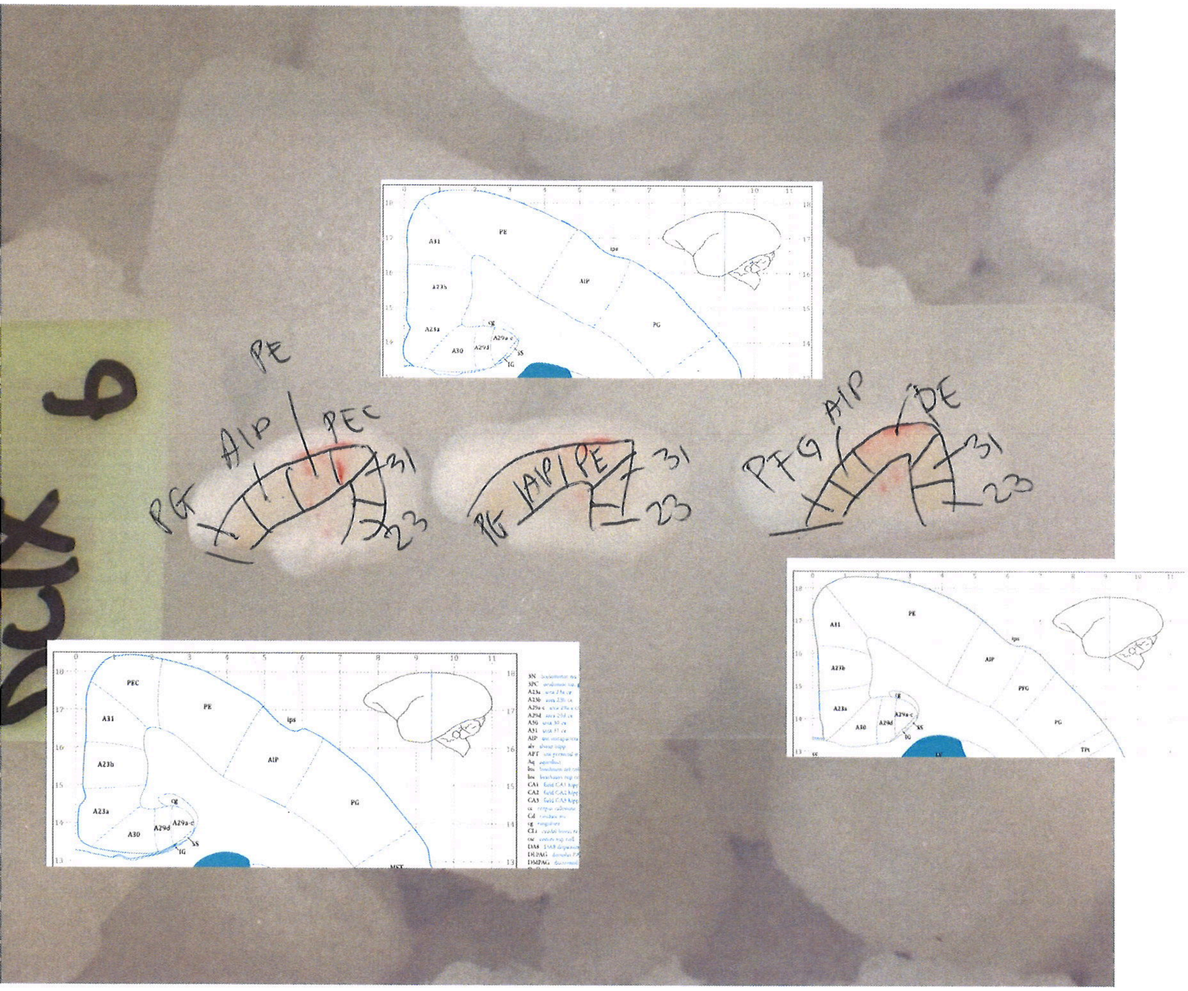
PE
AIP
PEC
PG
31
23
PFG
A31
A23b
A23a
A30
ips

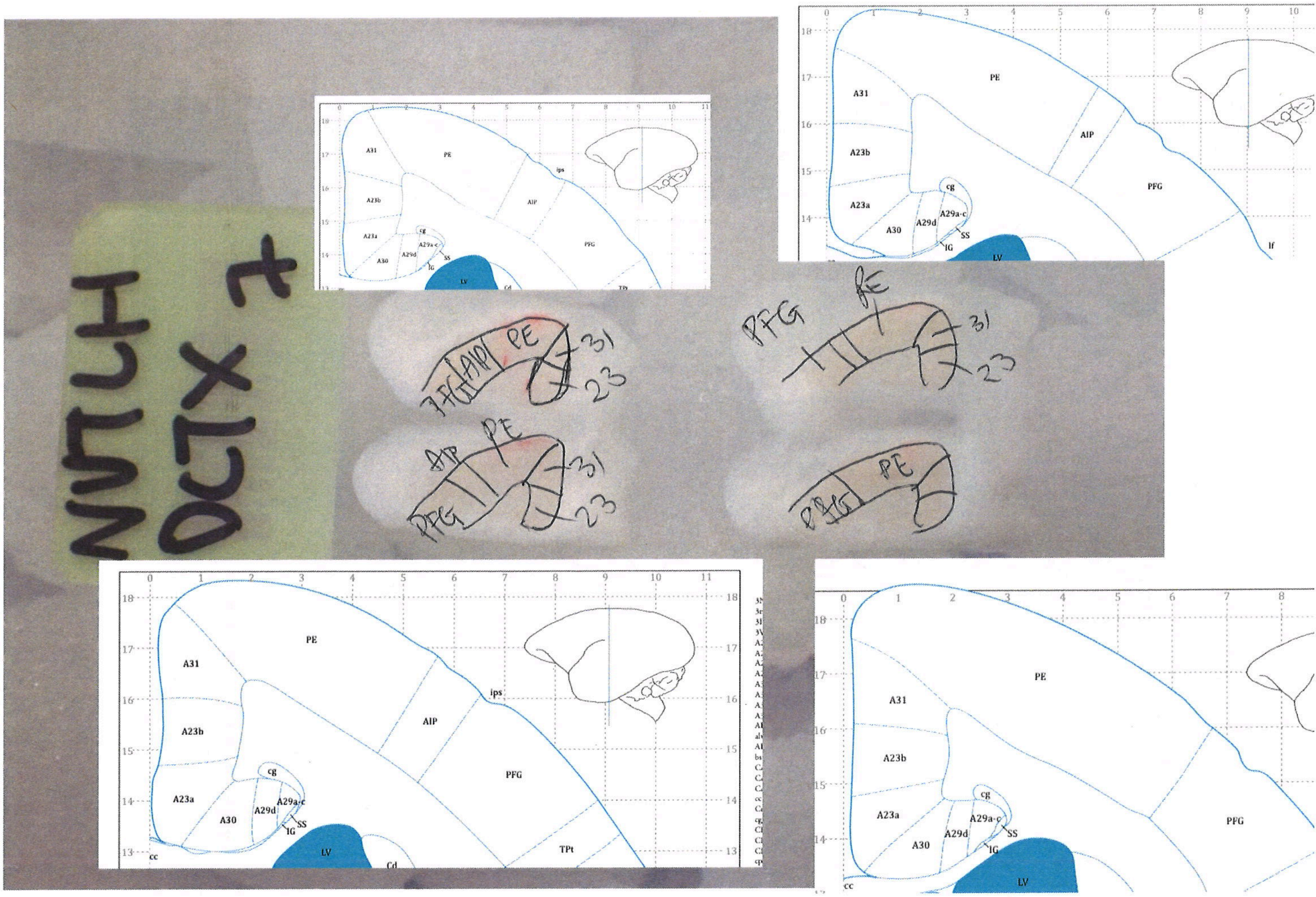
PE
A31
A23b
A23a
A30
A29d
A29a-c
cg
SS
IG
LV
Cd
AIP
PFG
ips
TPt
lf
cc
PFG
PE
31
23
AIP

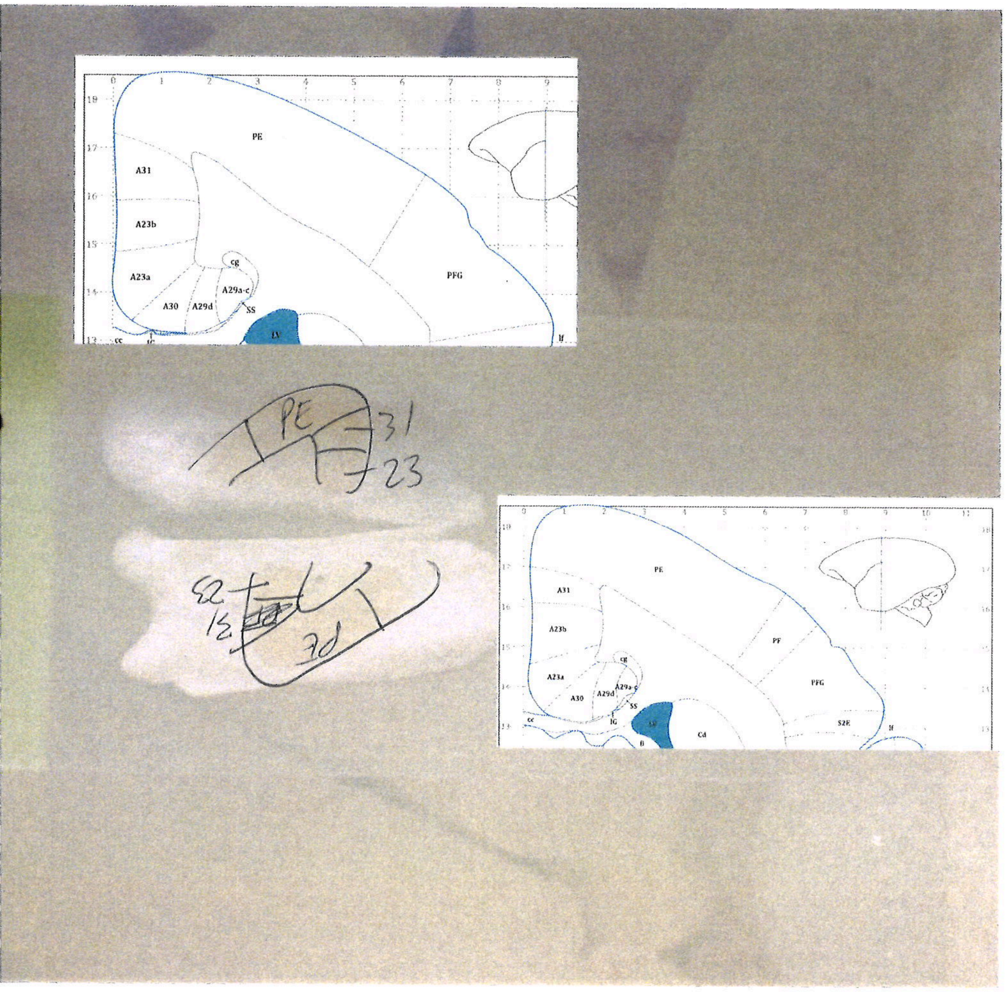
PE
A31
A23b
A23a
A30
A29d
A29a-c
cg
SS
LV
PFG
lf
PE
31
23
PE
A31
A23b
A23a
A30
A29d
SS
IG
Cd
PF
PFG
S2E
lf
