# Supplementary material for: Conserved and specialized features of thalamocortical wiring revealed by single-cell projection mapping in mouse and marmoset
Source: bioRxiv. 2026 Jul 8:2026.07.07.736957. Preprint. [Version 1] doi: 10.64898/2026.07.07.736957 (PMC13371098; doi:10.64898/2026.07.07.736957)
Supplement: Supplement 6 — Supplementary File 1: zip file containing dissection slice images for the BARseq experiments [file media-6.zip › Supplementary File 1/Marmoset Dissections/scan_xiaoyin.chen_2025-11-23-15-28-00.pdf]

frontal CTX : 1mm - 1.5mm
(3-5 sections)
other: 1.5mm - 2.5
(5-8 sections)

LH PFC

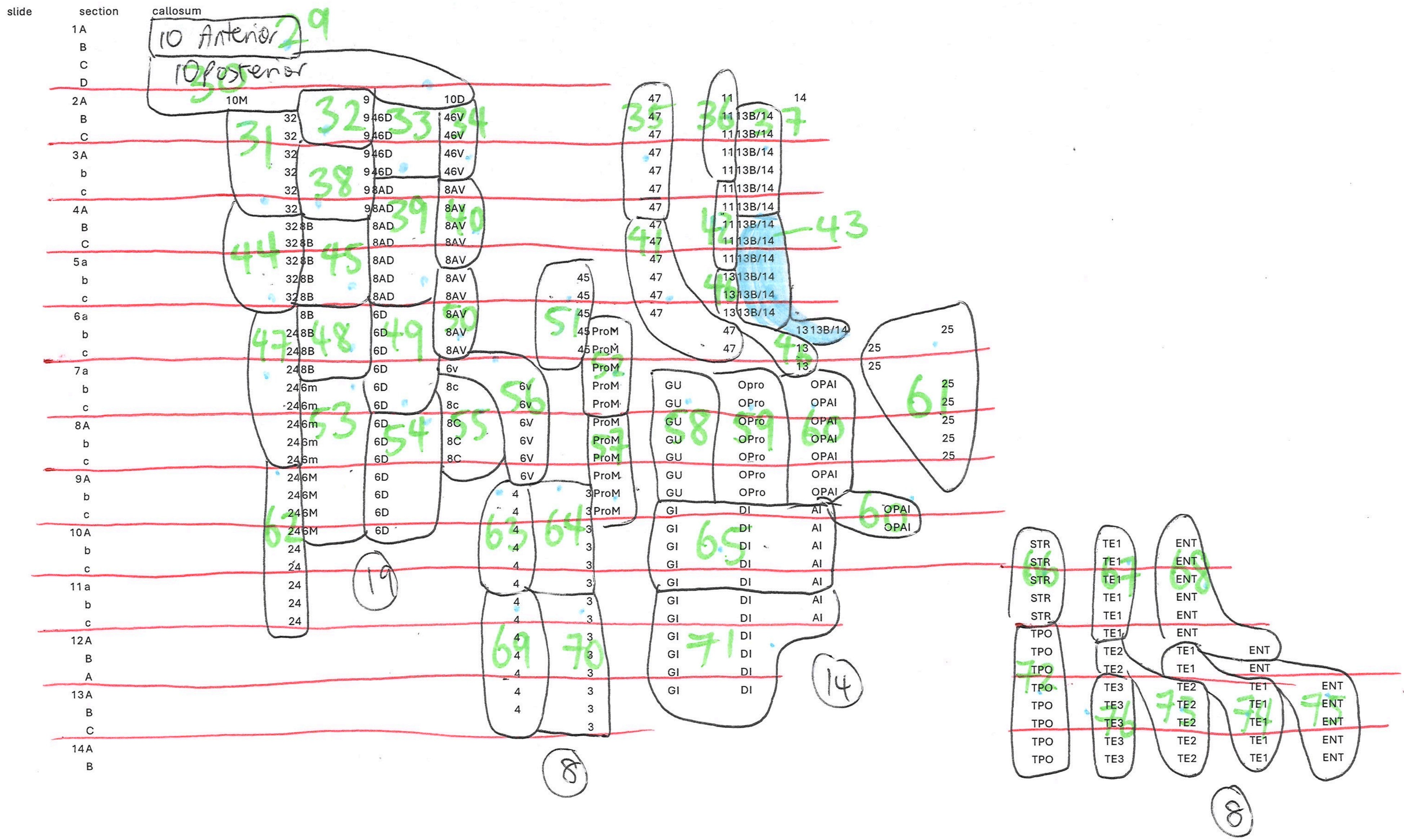

49 total tubes

LH lateral cortex

slide	section						
1	a	VL	V4				
	b	VL	V4				
	c	MTC	VL		TEO		
	d	MTC			TEO		
	e	MTC			TEO		
2	A	MT	MTC		TEO		
	B	MT	MTC	FST	TEO		
	C	MT		FST	TEO		
	D	MT		FST	TE3	TEO	
3	A	MT		FST	TE3	TEO	TFO
	B	MT			TE3	TEO	TFO
	C	MT			TE3	TEO	TFO
	D	MT			TE3	TEO	
4	A	MT		FST	TE3	TEO	
	B	MT		FST	TE3	TE2	
	C	MT		FST	TE3	TE2	
	D	MT		FST	TE3	TE2	
5	A	MST	FST	PGA	TE3	TE2	
	B	MST	FST	PGA	TE3	TE2	
	C	MST	FST	PGA	TE3	TE2	
	D	MST	FST	PGA	TE3	TE2	
6	A	MST	FST	PGA	TE3	TE2	
	B		TPO	PGA	TE3	TE2	
	C						

LH dorsal cortex

slide	section							
1	a							
	b	19M	V6			19D		
	c	19M	V6			19D		
	d	19M	V6			19D		
2	A	19M	V6		3A			
	B	19M	V6		3A			
	C	19M	V6		3A			
	D	19M	V6		3A	MIP	LIP	
3	A	19M	V6		3A	MIP	LIP	
	B	19M	V6			MIP	LIP	OPT
	C	PGM	V6			MIP	LIP	OPT
	D	PGM	V6			MIP	LIP	OPT
4	A	PGM	V6				LIP	OPT
	B	PGM	V6		PEC		LIP	OPT
	C	PGM	V6		PEC		LIP	OPT
	D		23	31	PEC		LIP	PG
5	A		23	31	PEC		LIP	PG
	B		23	31	PEC	PE	LIP	PG
	C		23	31	PEC	PE	LIP	PG
6	A		23	31	PEC	PE	AIP	PG
	B		23	31		PE	AIP	PG
	C		23	31		PE	AIP	PFG
7	A		23	31		PE	AIP	PFG
	B		23	31		PE	AIP	PFG
	C		23	31		PE		PFG
	D		23	31		PE		PFG
8	A		23	31		PE		
	B		23	31		PE		

Handwritten annotations: 29 (19M), 30 (V6), 31 (19D), 32 (3A), 33 (MIP), 34 (LIP), 35 (OPT), 36 (PGM), 37 (PEC), 38 (LIP), 39 (23), 40 (31), 41 (PG), 42 (PE), 43 (AIP), 44 (PFG), 45 (V3 V3 V3)

16 tubes

LH Visual cortex

slide	section	area	are	area		
	1 a	V1 fovea				
	1 b	v1 fovea				
	1 c	v1 fovea				
	1 d	v1 fovea				
	2 a	v1 fovea				
	2 b	v1 fovea				
	2 c	v1 fovea	v1 calcrine			
	3 a	v1 fovea	v1 calcrine			
	3 b	v1 fovea	v1 calcrine			
	3 c	v1 fovea	v1 calcrine			
	4 a	v1 fovea	v1 calcrine			
	4 b	v1 fovea	v1 calcrine			
	4 c	v1 fovea	v1 calcrine			
	5 a	V1 operculum dorsal	V1 opervulum ventral	V1 calcrine		
	5 b	V1 operculum dorsal	V1 opervulum ventral	V1 calcrine	V2 ventrolatera	
	5 c	V1 operculum dorsal	V1 opervulum ventral	V1 calcrine	V2 ventrolatera	
	6 a	V1 operculum dorsal	V2 dorsal	v2 ventral	V1 calcrine	
	6 b	V1 calcrine	V2 dorsal	v2 ventral		
	6 c	V1 calcrine	V2 dorsal	v2 ventral		
	7 a	V2 dorsal	v2 ventral	V1 calcrine		
	7 b	V2 dorsal	v2 ventral	V1 calcrine		
	7 c	V2 dorsal	v2 ventral	V1 calcrine		
	8 a	V2 dorsal	v2 ventral	V1 calcrine		
	b	V2 dorsal	v2 ventral	V1 calcrine	V3	
	c	V2 dorsal	v2 ventral	V1 calcrine	V3	
	9 a	V2 dorsal	v2 ventral	V1 calcrine	V3	
	b	V2 dorsal	v2 ventral	V1 calcrine	V3	
	c	V2 dorsal	v2 ventral	V1 calcrine	V3	A19
	10 a	V2 dorsal	V3	V1 calcrine	A19	
	b	V6	V3	V1 calcrine	A19	
	c	V6	V3 dorsal	V1 calcrine	A19	V3 ventral

Tube 1, V1 fovea #1

Tube 3, V1 fov #2

Tube 2, V1 calc #1

#4

#5

#6

calcrine #2, include 6B, 6C

V2 ventral

calcrine#3 [illegible]

#8

12

RH PFC

slide	section	Callosum	area	area														sulcus					
	1a			10																			
	b			10																			
	c			10																			
	d			10																			
	2a		10M			9		10D				47	11		14								
	b		10M			9	46D	46V				47	11	13B/14									
	c			32		9	46D	46V				47	11	13B/14									
	3A			32		9	46D	46V				47	11	13B/14									
	B			32		9	46D	46V				47	11	13B/14									
	C			32		9	8AD	8AV				47	11	13B/14									
	4A			32			8AD	8AV				47	11	13B/14									
	B			32	8B		8AD	8AV				47	11	13B/14									
	C			32	8B		8AD	8AV				47	11	13B/14									
	5a			32	8B		8AD	8AV				47	11	13B/14									
	b			32	8B		8AD	8AV		45		47	13	13B/14									
	6a				8B		8AD	6D	8AV														
	b			24	8B		6D	8AV		45		47	13	13B/14									
	c			24	8B		6D	8AV		45	ProM		47		13		25						
	7a			24	8B		6D	6v			ProM				13		25						
	b			24	6m		6D	6v			ProM	GU		Opro			25						
	c			24	6m		6D	6v			ProM	GU		OPro			25						
	8A			24	6m		6D	8C	6V		ProM	GU		OPro		OPAI		25					
	b			24	6m		6D	8C	6V		ProM	GU		OPro		OPAI		25					
	c			24	6m		6D	8C	6V		ProM	GU		OPro		OPAI							
	9A			24	6M		6D	8C	6V		ProM	GU		OPro		OPAI							
	b			24	6M		6D		4		ProM	GU		OPro		OPAI							
	10A			24	6M		6D		4	3	ProM	GI		DI		AI	OPAI						
	B			24					4	3		GI		DI		AI			STR	TE1	ENT		
	C			24					4	3		GI		DI		AI			STR	TE1	ENT		
	11A			24					4	3		GI		DI		AI			STR	TE1	ENT		
	B			24					4	3		GI		DI		AI			STR	TE1	ENT		
	12A			24					4	3		GI		DI		AI			STR	TE1	ENT		
	B								4	3									STR	TE1	ENT		
	13A								4	3		GI		DI					TPO	TE2	TE1	ENT	
	B								4	3		GI		DI					TPO	TE2	TE1	ENT	
	14A								4	3		GI		DI					TPO	TE3	TE2	TE1	ENT

total: 47

RH lateral cortex

slide	section						
	1 a	MT		TEO	TFO		
	b	MT	TE3	TEO			
	c	MT	TE3	TEO			
	d	MT	TE3	TEO	TFO		
	e	MT	TE3	TEO	TEO		
	2 A	MT	FST	TE3	TEO	TFO	
	B	MT	FST	TE3	TEO	TFO	
	C	MT	FST	TE3	TEO	TFO	
	D	MT	FST	TE3	TE2	TF	
	3 A	MT	FST	TE3	TE2	TF	
	B	MT	FST	TE3	TE2	TF	
	C	MT	FST	TE3	TE2	TF	
	D	MT	FST	TE3	TE2	TF	
	4 A	FST	PGA	TE3	TE2	TF	
	B	MST	FST	PGA	TE3	TE2	TF
	C	MST	FST	PGA	TE3	TE2	TF
	D			PGA	TE3	TE2	TF
	5 A			PGA	TE3	TE2	TF
	B			PGA	TE3	TE2	TF

46 49 47 48 50 51 52 53 54 55 56 57 58 59

14

RH dorsal cortex

slide	section							
	1A							
	B							
	C		3	1	PE	PF		
	D		23		PE	PF		
	2A		23	31	PE	PF		
	B		23	31	PE	PFG		
	C		23	31	PE	PFG		
	D		23	31	PE	PFG		
	3A		23	31	PE	AIP	PFG	
	B		23	31	PE	AIP	PFG	
	C		23	31	PE	AIP	PFG	
	D		23	31	PE	AIP	PFG	
	4A		23	31	PEC	PE	AIP	PG
	B		23	31	PEC	PE	LIP	PG
	C		23	31	PEC	PE	LIP	PG
	D		23	31	PEC	PE	LIP	PG
	5A		23	31	V6	PEC	LIP	PG
	B		23	31	V6	PEC	LIP	OPT
	C		23	31	V6	PEC	LIP	OPT
	6A	PGM			V6	PEC	LIP	OPT
	B	PGM	A19		V6		LIP	OPT

15

MIP 81

slides B, C 6AB.

RH visual cortex

slide	section										
	1A	V1 fovea									
	B	v1 fovea									
	C	V1 fovea									
	2A	v1 fovea									
	B	V1 fovea									
	C	v1 fovea									
	3A	V1 calcrine	V1 fovea								
	B	V1 calcrine	V1 fovea								
	C	V1 calcrine	V1 fovea								
	4A	V1 calcrine	V1 fovea								
	B	V1 calcrine	V1 fovea								
	C	V1 calcrine	V1 fovea								
	5a										
	b	V1 calcrine	v1OD'	V2	V1OV						
	c	V1 calcrine	v2D	v1OD	V2 V	V1OV					
	6a	V1 calcrine	v2D		V2 V	V1OV					
	b	V1 calcrine	v2D		V2 V	V1OV					
	c	V1 calcrine	v2D		V2 V	V1OV					
	7a	V1 calcrine	v2D		V2 V						
	b	V1 calcrine	v2D	V3?	V2 V						
	8a	V1 calcrine	v2D	V3	V2 V						
	b	V1 calcrine	v2D	V3	V2 V						
	9a	V1 calcrine	v2D	V3	V2 V						
	b	V1 calcrine	v2D	A19D(?)	V3	V2 V					
	10a	V1 calcrine	v2D	A19D	V3	V2 V					
	b	V1 calcrine	v2D	A19D	V3	V2 V					
	11a	V1 calcrine	v2M	V6	V2D	A19D	V3	V2V			
	b	V1 calcrine	v2M	V6		A19D	V3	V2V			
	12a	V1 calcrine	v2M	V6		A19D	V3	V2V			
	b	V1 calcrine	v2M	A19M	V6	A19D	V3D	V4(?)	V3V	V2V	
	c	V1 calcrine	v2M	A19M	V6	A19D	V3D	V4	V3V	V2V	
	13a	V1 calcrine	v2M	A19M	V6	A19D	V3D	V4	V3V	V2V	
	b	V1 calcrine	v2M	A19M	V6	A19D	V3D	V4	V3V	V2V	
	c	V1 calcrine	v2M	A19M	V6	A19D	V3D	V4	V3V	V2V	
	14a	V1 calcrine	v2M	A19M	V6	V3A	VL	V4	V3V	V2V	
	b	V1 calcrine	v2M	A19M	V6	V3A	VL	V4	TEO	V3V	V2V
