# Supplementary material for: Conserved and specialized features of thalamocortical wiring revealed by single-cell projection mapping in mouse and marmoset
Source: bioRxiv. 2026 Jul 8:2026.07.07.736957. Preprint. [Version 1] doi: 10.64898/2026.07.07.736957 (PMC13371098; doi:10.64898/2026.07.07.736957)
Supplement: Supplement 6 — Supplementary File 1: zip file containing dissection slice images for the BARseq experiments [file media-6.zip › Supplementary File 1/Marmoset Dissections/tissue printout.pdf]

LH PFC

slide	section	callosum																
1	A																	
	B																	
	C																	
	D																	
2	A		10M	9		10D		47	11	14								
	B		32	9	46D	46V		47	11	13B/14								
	C		32	9	46D	46V		47	11	13B/14								
3	A		32	9	46D	46V		47	11	13B/14								
	b		32	9	46D	46V		47	11	13B/14								
	c		32	9	8AD	8AV		47	11	13B/14								
4	A		32	9	8AD	8AV		47	11	13B/14								
	B		32	8B	8AD	8AV		47	11	13B/14								
	C		32	8B	8AD	8AV		47	11	13B/14								
5	a		32	8B	8AD	8AV		47	11	13B/14								
	b		32	8B	8AD	8AV	45	47	13	13B/14								
	c		32	8B	8AD	8AV	45	47	13	13B/14								
6	a			8B	6D	8AV	45	47	13	13B/14								
	b		24	8B	6D	8AV	45	ProM	47	13	13B/14	25						
	c		24	8B	6D	8AV	45	ProM	47	13	25							
7	a		24	8B	6D	6v		ProM		13	25							
	b		24	6m	6D	8c	6v	ProM	GU	Opro	OPAI	25						
	c		24	6m	6D	8c	6v	ProM	GU	OPro	OPAI	25						
8	A		24	6m	6D	8C	6V	ProM	GU	OPro	OPAI	25						
	b		24	6m	6D	8C	6V	ProM	GU	OPro	OPAI	25						
	c		24	6m	6D	8C	6V	ProM	GU	OPro	OPAI	25						
9	A		24	6M	6D		6V	ProM	GU	OPro	OPAI							
	b		24	6M	6D	4	3	ProM	GU	OPro	OPAI							
	c		24	6M	6D	4	3	ProM	GI	DI	AI	OPAI						
10	A		24	6M	6D	4	3		GI	DI	AI	OPAI						
	b		24			4	3		GI	DI	AI			STR	TE1	ENT		
	c		24			4	3		GI	DI	AI			STR	TE1	ENT		
11	a		24			4	3		GI	DI	AI			STR	TE1	ENT		
	b		24			4	3		GI	DI	AI			STR	TE1	ENT		
	c		24			4	3		GI	DI	AI			STR	TE1	ENT		
12	A					4	3		GI	DI				TPO	TE1	ENT		
	B					4	3		GI	DI				TPO	TE2	TE1	ENT	
	A					4	3		GI	DI				TPO	TE2	TE1	ENT	
13	A					4	3		GI	DI				TPO	TE3	TE2	TE1	ENT
	B					4	3							TPO	TE3	TE2	TE1	ENT
	C						3							TPO	TE3	TE2	TE1	ENT
14	A													TPO	TE3	TE2	TE1	ENT
	B													TPO	TE3	TE2	TE1	ENT

LH lateral cortex

slide	section						
1	a	VL	V4				
	b	VL	V4				
	c	MTC	VL		TEO		
	d	MTC			TEO		
	e	MTC			TEO		
2	A	MT	MTC		TEO		
	B	MT	MTC	FST	TEO		
	C	MT		FST	TEO		
	D	MT		FST	TE3	TEO	
3	A	MT		FST	TE3	TEO	TFO
	B	MT			TE3	TEO	TFO
	C	MT			TE3	TEO	TFO
	D	MT			TE3	TEO	
4	A	MT		FST	TE3	TEO	
	B	MT		FST	TE3	TE2	
	C	MT		FST	TE3	TE2	
	D	MT		FST	TE3	TE2	
5	A	MST	FST	PGA	TE3	TE2	
	B	MST	FST	PGA	TE3	TE2	
	C	MST	FST	PGA	TE3	TE2	
	D	MST	FST	PGA	TE3	TE2	
6	A	MST	FST	PGA	TE3	TE2	
	B		TPO	PGA	TE3	TE2	
	C						

LH dorsal cortex

slide	section						
1	a						
	b	19M	V6		19D		
	c	19M	V6		19D		
	d	19M	V6		19D		
2	A	19M	V6	3A			
	B	19M	V6	3A			
	C	19M	V6	3A			
	D	19M	V6	3A	MIP	LIP	
3	A	19M	V6	3A	MIP	LIP	
	B	19M	V6		MIP	LIP	OPT
	C	PGM	V6		MIP	LIP	OPT
	D	PGM	V6		MIP	LIP	OPT
4	A	PGM	V6			LIP	OPT
	B	PGM	V6	PEC		LIP	OPT
	C	PGM	V6	PEC		LIP	OPT
	D	23	31	PEC		LIP	PG
5	A	23	31	PEC		LIP	PG
	B	23	31	PEC	PE	LIP	PG
	C	23	31	PEC	PE	LIP	PG
6	A	23	31	PEC	PE	AIP	PG
	B	23	31		PE	AIP	PG
	C	23	31		PE	AIP	PFG
7	A	23	31		PE	AIP	PFG
	B	23	31		PE	AIP	PFG
	C	23	31		PE		PFG
	D	23	31		PE		PFG
8	A	23	31		PE		
	B	23	31		PE		

LH Visual cortex

slide	section	area	are	area		
1	a	V1 fovea				
1	b	v1 fovea				
1	c	v1 fovea				
1	d	v1 fovea				
2	a	v1 fovea				
2	b	v1 fovea				
2	c	v1 fovea	v1 calcrine			
3	a	v1 fovea	v1 calcrine			
3	b	v1 fovea	v1 calcrine			
3	c	v1 fovea	v1 calcrine			
4	a	v1 fovea	v1 calcrine			
4	b	v1 fovea	v1 calcrine			
4	c	v1 fovea	v1 calcrine			
5	a	V1 operculum dorsal	V1 opervulum ventral	V1 calcrine		
5	b	V1 operculum dorsal	V1 opervulum ventral	V1 calcrine	V2 ventrolatera	
5	c	V1 operculum dorsal	V1 opervulum ventral	V1 calcrine	V2 ventrolatera	
6	a	V1 operculum dorsal	V2 dorsal	v2 ventral	V1 calcrine	
6	b	V1 calcrine	V2 dorsal	v2 ventral		
6	c	V1 calcrine	V2 dorsal	v2 ventral		
7	a	V2 dorsal	v2 ventral	V1 calcrine		
7	b	V2 dorsal	v2 ventral	V1 calcrine		
7	c	V2 dorsal	v2 ventral	V1 calcrine		
8	a	V2 dorsal	v2 ventral	V1 calcrine		
	b	V2 dorsal	v2 ventral	V1 calcrine	V3	
	c	V2 dorsal	v2 ventral	V1 calcrine	V3	
9	a	V2 dorsal	v2 ventral	V1 calcrine	V3	
	b	V2 dorsal	v2 ventral	V1 calcrine	V3	
	c	V2 dorsal	v2 ventral	V1 calcrine	V3	A19
10	a	V2 dorsal	V3	V1 calcrine	A19	
	b	V6	V3	V1 calcrine	A19	
	c	V6	V3 dorsal	V1 calcrine	A19	V3 ventral

RH PFC

slide	section	Callosum	area	area									sulcus					
1	a		10															
	b		10															
	c		10															
	d		10															
2	a		10M	9		10D		47	11	14								
	b		10M	9	46D	46V		47	11	13B/14								
	c		32	9	46D	46V		47	11	13B/14								
3	A		32	9	46D	46V		47	11	13B/14								
	B		32	9	46D	46V		47	11	13B/14								
	C		32	9	8AD	8AV		47	11	13B/14								
4	A		32		8AD	8AV		47	11	13B/14								
	B		32	8B	8AD	8AV		47	11	13B/14								
	C		32	8B	8AD	8AV		47	11	13B/14								
5	a		32	8B	8AD	8AV		47	11	13B/14								
	b		32	8B	8AD	8AV	45	47	13	13B/14								
6	a			8B	8AD	6D	8AV											
	b		24	8B	6D	8AV	45	47	13	13B/14	25							
	c		24	8B	6D	8AV	45	ProM	47	13	25							
7	a		24	8B	6D	6v		ProM		13	25							
	b		24	6m	6D	6v		ProM	GU	Opro	25							
	c		24	6m	6D	6v		ProM	GU	OPro	25							
8	A		24	6m	6D	8C	6V	ProM	GU	OPro	OPAI	25						
	b		24	6m	6D	8C	6V	ProM	GU	OPro	OPAI	25						
	c		24	6m	6D	8C	6V	ProM	GU	OPro	OPAI							
9	A		24	6M	6D	8C	6V	ProM	GU	OPro	OPAI							
	b		24	6M	6D	4		ProM	GU	OPro	OPAI							
10	A		24	6M	6D	4	3	ProM	GI	DI	AI	OPAI						
	B		24			4	3		GI	DI	AI			STR	TE1	ENT		
	C		24			4	3		GI	DI	AI			STR	TE1	ENT		
11	A		24			4	3		GI	DI	AI			STR	TE1	ENT		
	B		24			4	3		GI	DI	AI			STR	TE1	ENT		
12	A		24			4	3		GI	DI	AI			STR	TE1	ENT		
	B					4	3							STR	TE1	ENT		
13	A					4	3		GI	DI				TPO	TE2	TE1	ENT	
	B					4	3		GI	DI				TPO	TE2	TE1	ENT	
14	A					4	3		GI	DI				TPO	TE3	TE2	TE1	ENT

RH lateral cortex

slide	section						
1	a	MT		TEO	TFO		
	b	MT	TE3	TEO			
	c	MT	TE3	TEO			
	d	MT	TE3	TEO	TFO		
	e	MT	TE3	TEO	TFO		
2	A	MT	FST	TE3	TEO	TFO	
	B	MT	FST	TE3	TEO	TFO	
	C	MT	FST	TE3	TEO	TFO	
	D	MT	FST	TE3	TE2	TF	
3	A	MT	FST	TE3	TE2	TF	
	B	MT	FST	TE3	TE2	TF	
	C	MT	FST	TE3	TE2	TF	
	D	MT	FST	TE3	TE2	TF	
4	A	FST	PGA	TE3	TE2	TF	
	B	MST	FST	PGA	TE3	TE2	TF
	C	MST	FST	PGA	TE3	TE2	TF
	D			PGA	TE3	TE2	TF
5	A			PGA	TE3	TE2	TF
	B			PGA	TE3	TE2	TF

RH dorsal cortex

slide	section						
1	A						
	B						
	C	3	1	PE	PF		
	D	23		PE	PF		
2	A	23	31	PE	PF		
	B	23	31	PE	PFG		
	C	23	31	PE	PFG		
	D	23	31	PE	PFG		
3	A	23	31	PE	AIP	PFG	
	B	23	31	PE	AIP	PFG	
	C	23	31	PE	AIP	PFG	
	D	23	31	PE	AIP	PFG	
4	A	23	31	PEC	PE	AIP	PG
	B	23	31	PEC	PE	LIP	PG
	C	23	31	PEC	PE	LIP	PG
	D	23	31	PEC	PE	LIP	PG
5	A	23	31	V6	PEC	LIP	PG
	B	23	31	V6	PEC	LIP	OPT
	C	23	31	V6	PEC	LIP	OPT
6	A	PGM		V6	PEC	LIP	OPT
	B	PGM	A19	V6		LIP	OPT

RH visual cortex

slide	section										
1	A	V1 fovea									
	B	v1 fovea									
	C	V1 fovea									
2	A	v1 fovea									
	B	V1 fovea									
	C	v1 fovea									
3	A	V1 calcrine	V1 fovea								
	B	V1 calcrine	V1 fovea								
	C	V1 calcrine	V1 fovea								
4	A	V1 calcrine	V1 fovea								
	B	V1 calcrine	V1 fovea								
	C	V1 calcrine	V1 fovea								
5	a										
	b	V1 calcrine	v1OD	V2	V1OV						
	c	V1 calcrine	v2D	v1OD	V2 V	V1OV					
6	a	V1 calcrine	v2D		V2 V	V1OV					
	b	V1 calcrine	v2D		V2 V	V1OV					
	c	V1 calcrine	v2D		V2 V	V1OV					
7	a	V1 calcrine	v2D		V2 V						
	b	V1 calcrine	v2D	V3?	V2 V						
8	a	V1 calcrine	v2D	V3	V2 V						
	b	V1 calcrine	v2D	V3	V2 V						
9	a	V1 calcrine	v2D	V3	V2 V						
	b	V1 calcrine	v2D	A19D(?)	V3	V2 V					
10	a	V1 calcrine	v2D	A19D	V3	V2 V					
	b	V1 calcrine	v2D	A19D	V3	V2 V					
11	a	V1 calcrine	v2M	V6	V2D	A19D	V3	V2V			
	b	V1 calcrine	v2M	V6		A19D	V3	V2V			
12	a	V1 calcrine	v2M	V6		A19D	V3	V2V			
	b	V1 calcrine	v2M	A19M	V6	A19D	V3D	V4(?)	V3V	V2V	
	c	V1 calcrine	v2M	A19M	V6	A19D	V3D	V4	V3V	V2V	
13	a	V1 calcrine	v2M	A19M	V6	A19D	V3D	V4	V3V	V2V	
	b	V1 calcrine	v2M	A19M	V6	A19D	V3D	V4	V3V	V2V	
	c	V1 calcrine	v2M	A19M	V6	A19D	V3D	V4	V3V	V2V	
14	a	V1 calcrine	v2M	A19M	V6	V3A	VL	V4	V3V	V2V	
	b	V1 calcrine	v2M	A19M	V6	V3A	VL	V4	TEO	V3V	V2V
