# Supplementary material for: Conserved and specialized features of thalamocortical wiring revealed by single-cell projection mapping in mouse and marmoset
Source: bioRxiv. 2026 Jul 8:2026.07.07.736957. Preprint. [Version 1] doi: 10.64898/2026.07.07.736957 (PMC13371098; doi:10.64898/2026.07.07.736957)
Supplement: Supplement 6 — Supplementary File 1: zip file containing dissection slice images for the BARseq experiments [file media-6.zip › Supplementary File 1/Mouse Dissections/818353_dissection_annotation.pdf]

Sectioned from anterior : left side is right side

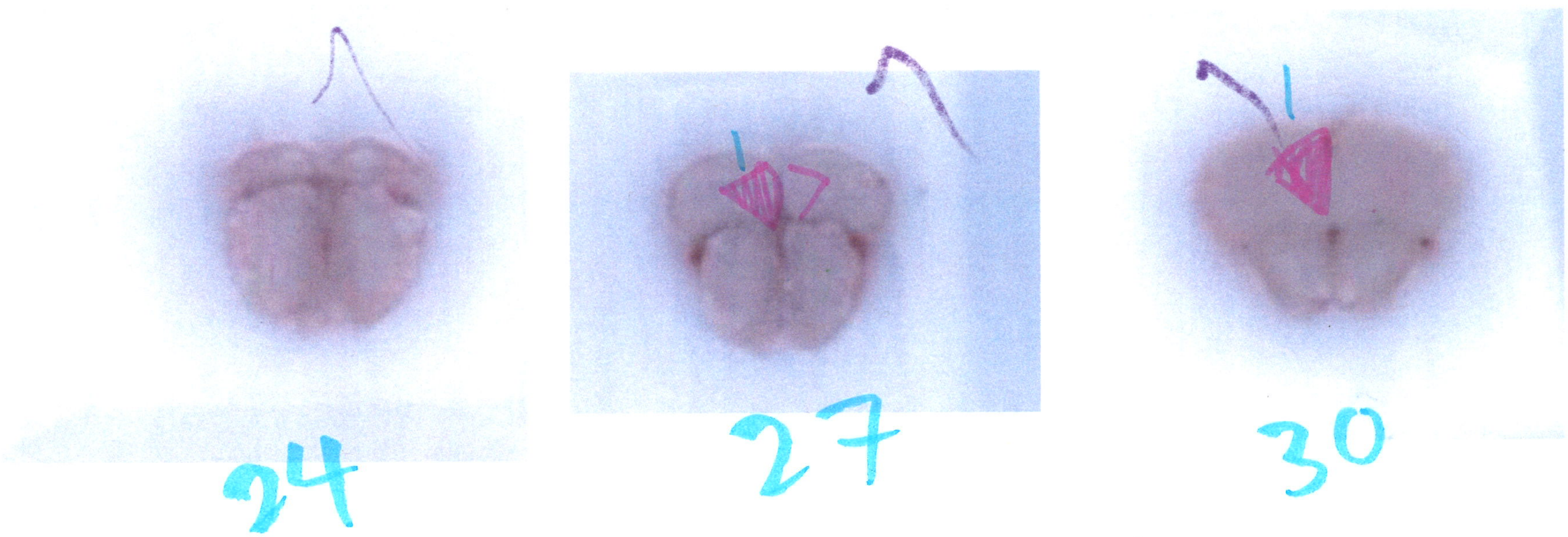

Sectioned from anterior : left side is right side

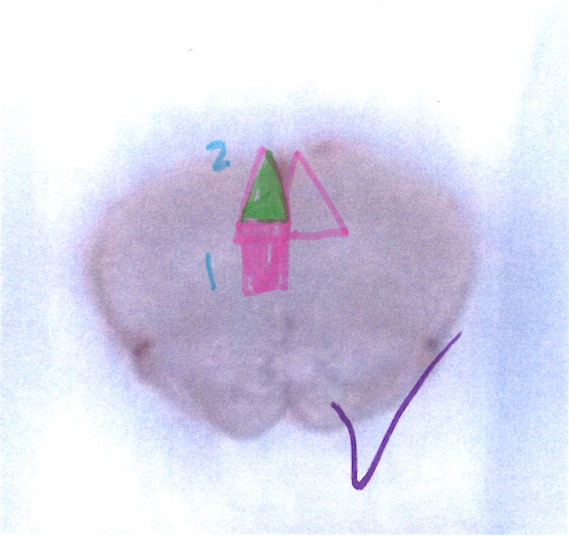


33

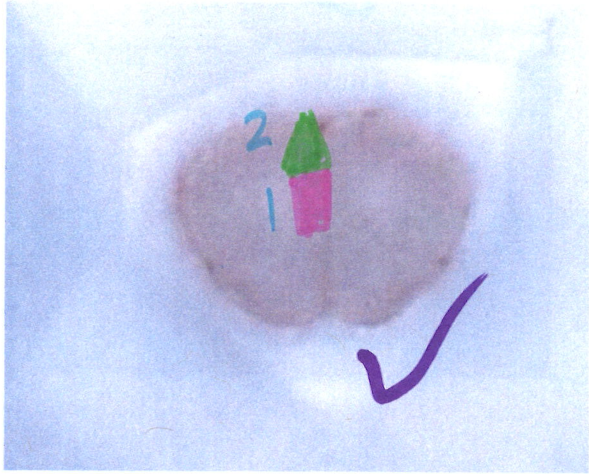


36

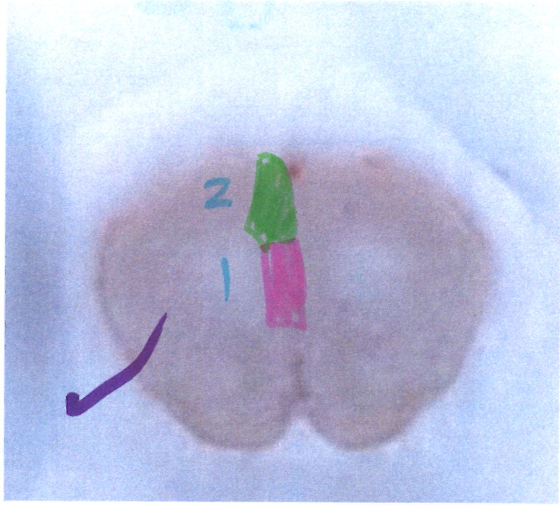


39

Sectioned from anterior : left side is right side

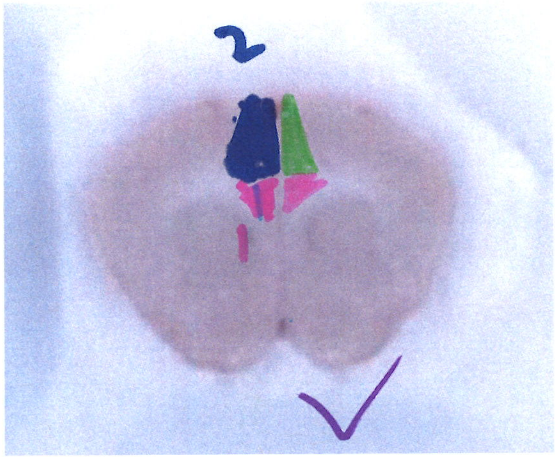


42

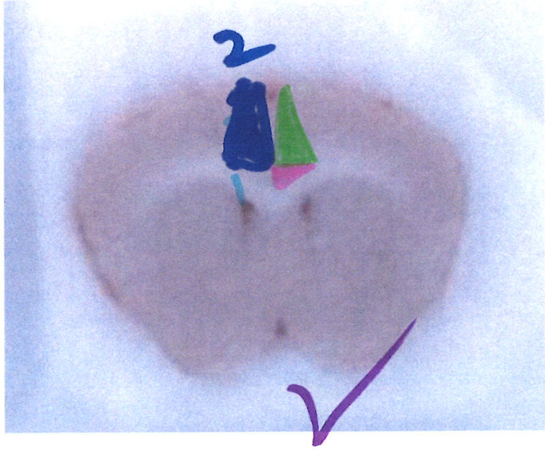


45
callosum connects

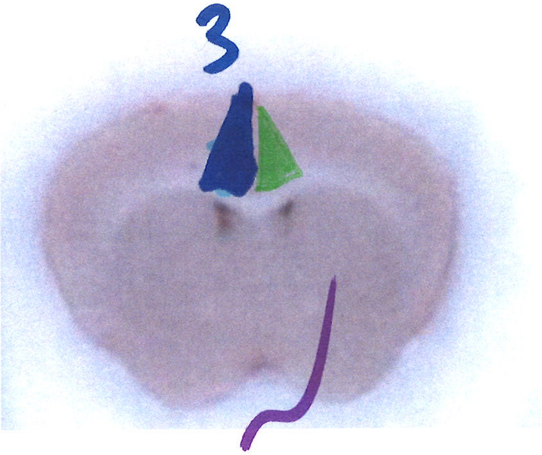


48

Sectioned from anterior : left side is right side

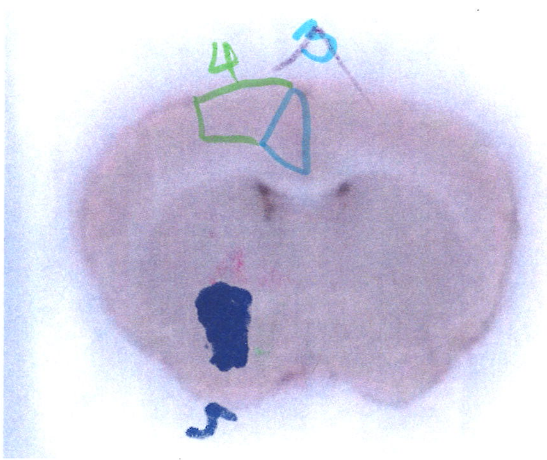


51

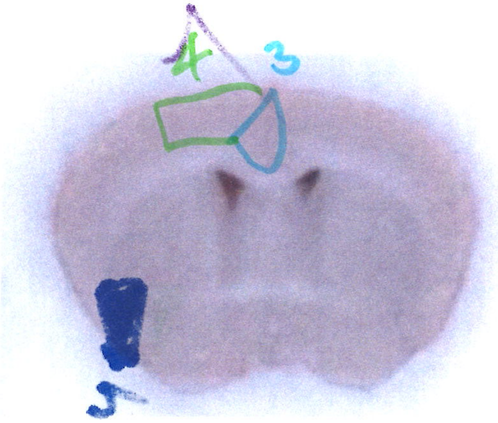


54

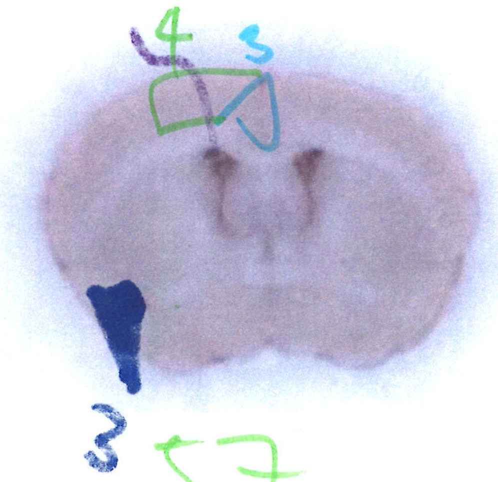


57

Sectioned from anterior : left side is right side

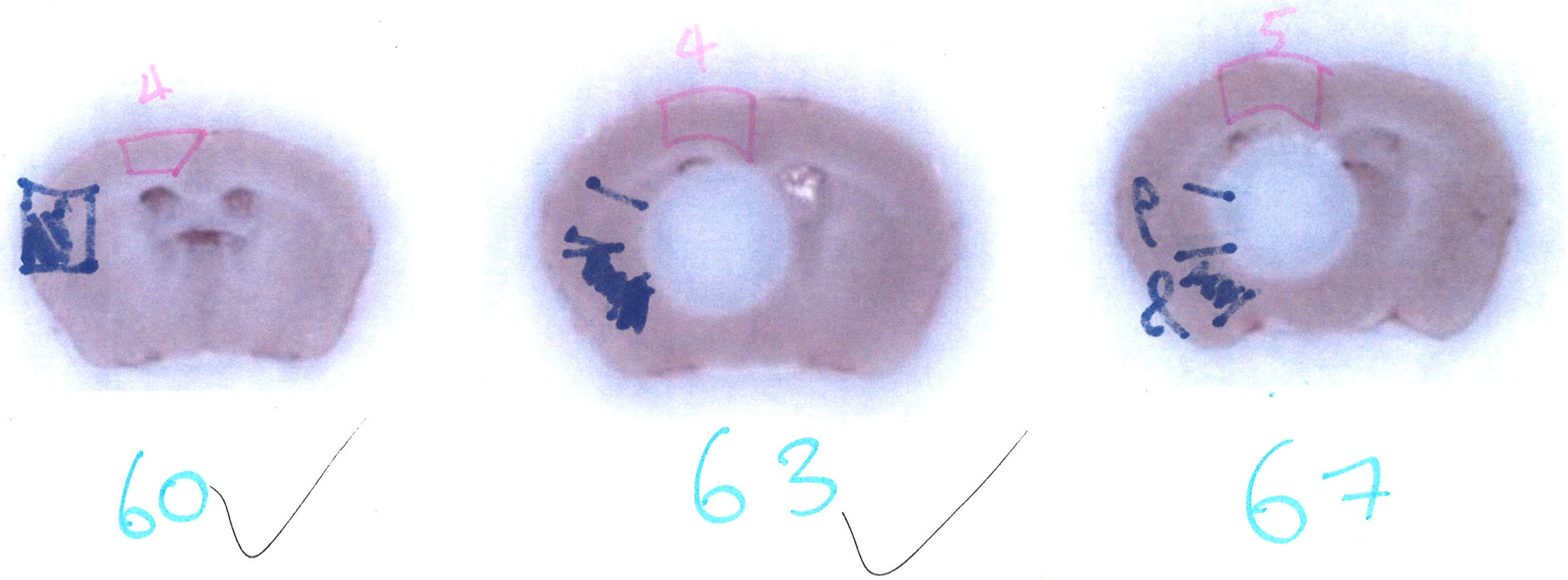

Sectioned from anterior : left side is right side

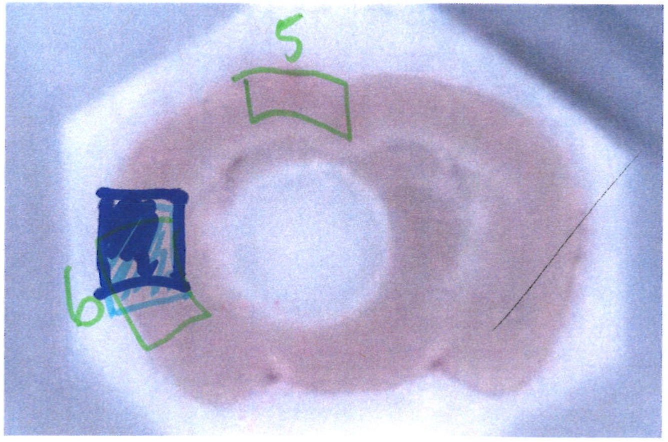


69

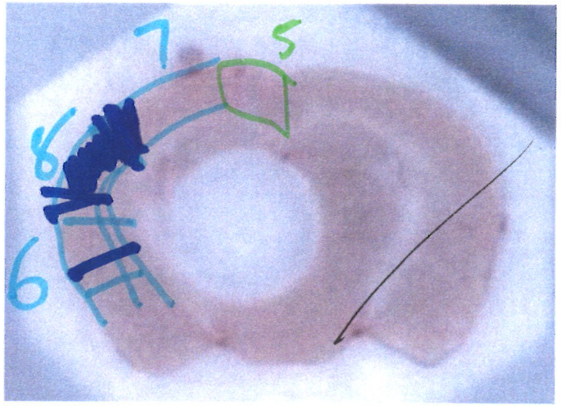


72

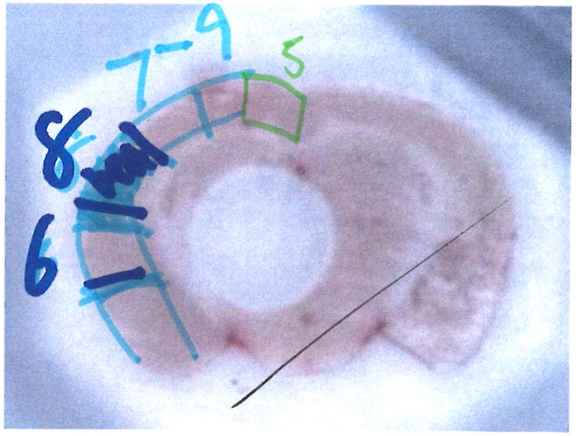


75

Notch appears

Sectioned from anterior : left side is right side

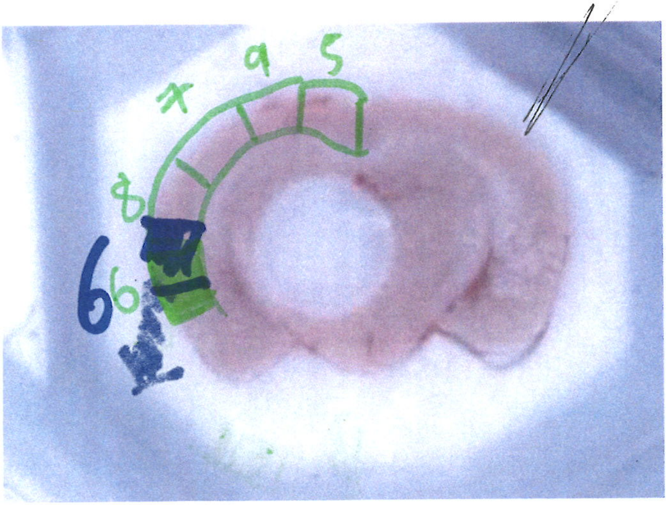

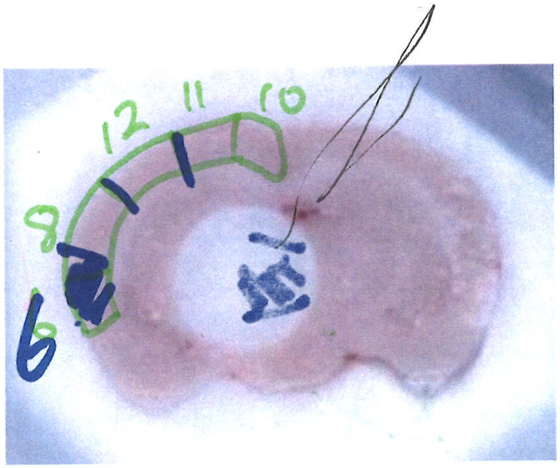

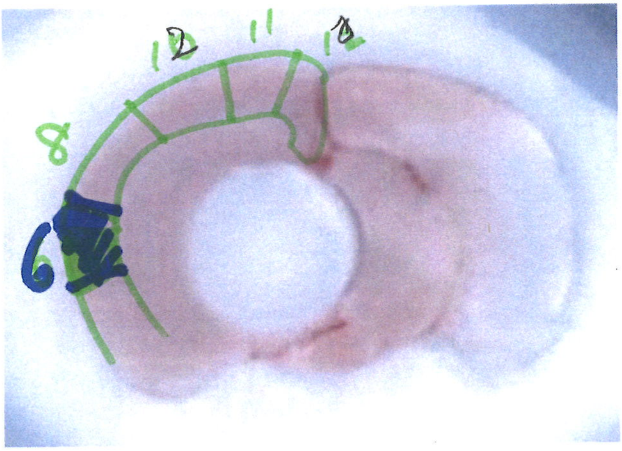

callosum connects:

78

p

81

84

Sectioned from anterior : left side is right side

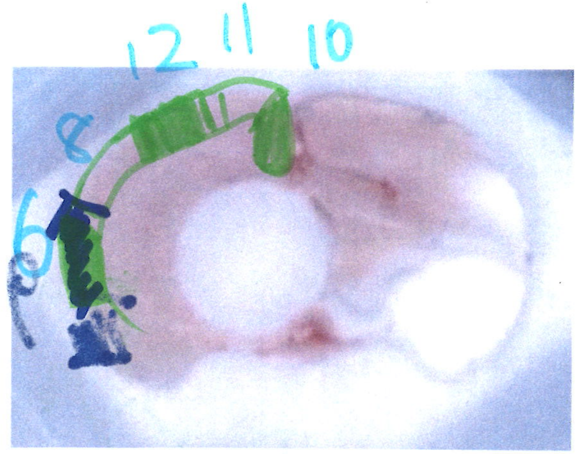


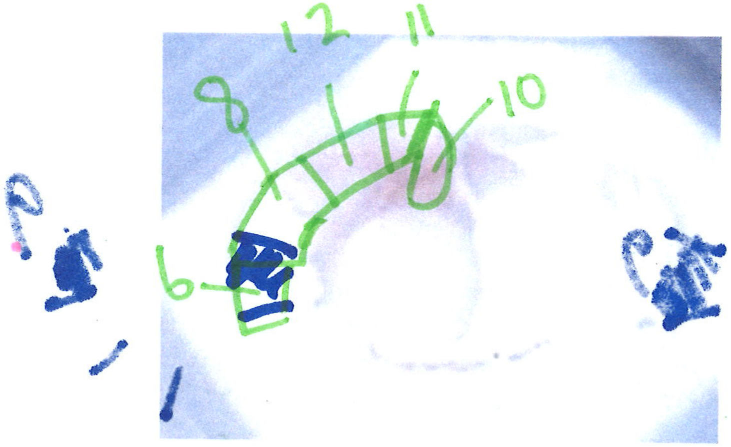


87

90

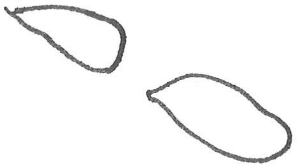

Sectioned from posterior : left side is left side

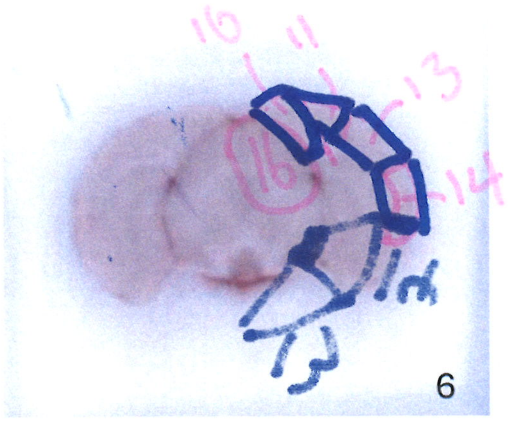


93

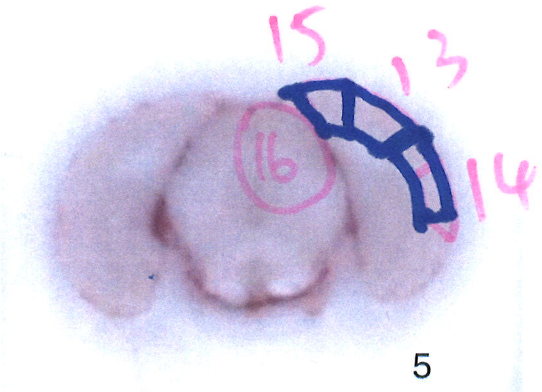


96

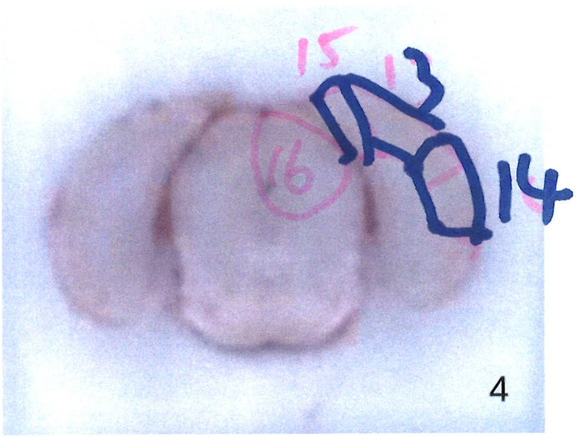


99

Sectioned from posterior : left side is left side

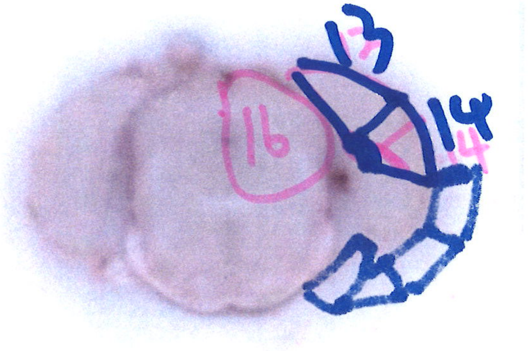


3

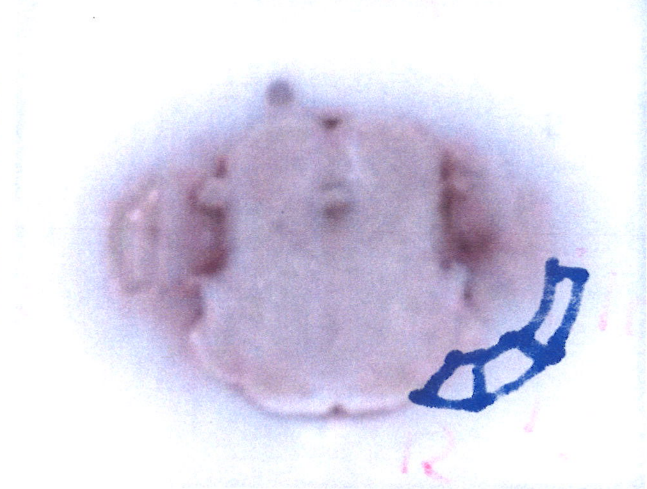

2

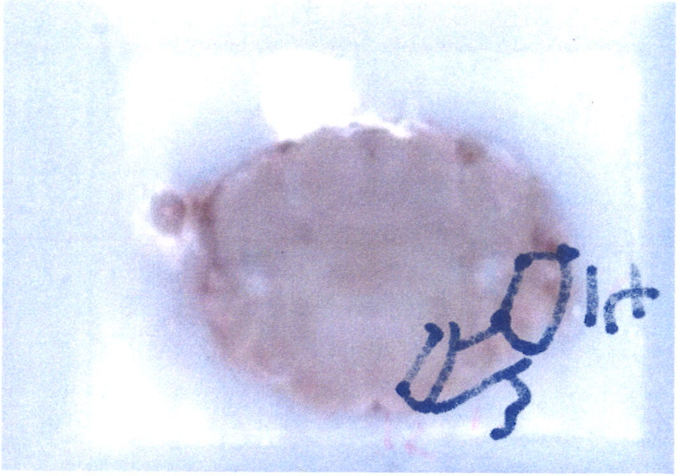


1

102
