# Supplementary material for: Conserved and specialized features of thalamocortical wiring revealed by single-cell projection mapping in mouse and marmoset
Source: bioRxiv. 2026 Jul 8:2026.07.07.736957. Preprint. [Version 1] doi: 10.64898/2026.07.07.736957 (PMC13371098; doi:10.64898/2026.07.07.736957)
Supplement: Supplement 6 — Supplementary File 1: zip file containing dissection slice images for the BARseq experiments [file media-6.zip › Supplementary File 1/Mouse Dissections/839168_dissection_annotation.pdf]

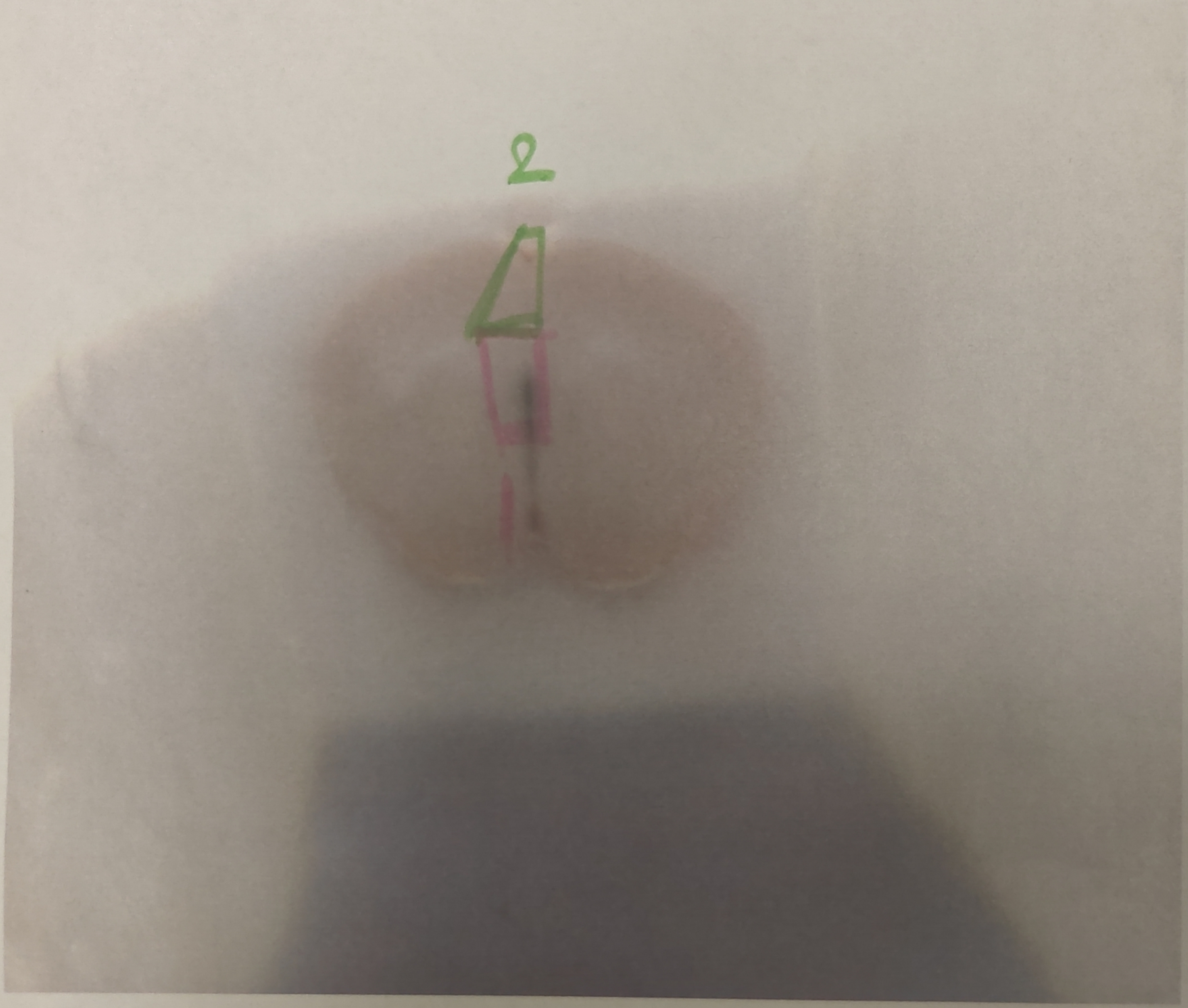

839168_11

CL40

Preceeding 2 sections

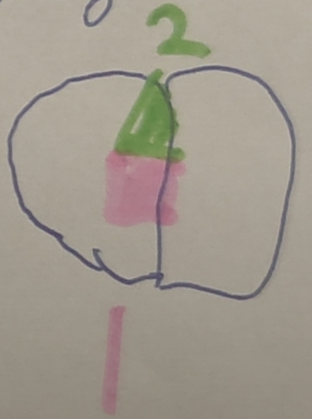

Preceeding 3-4 ~~3-5~~ sections

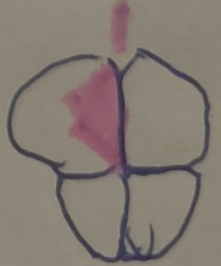

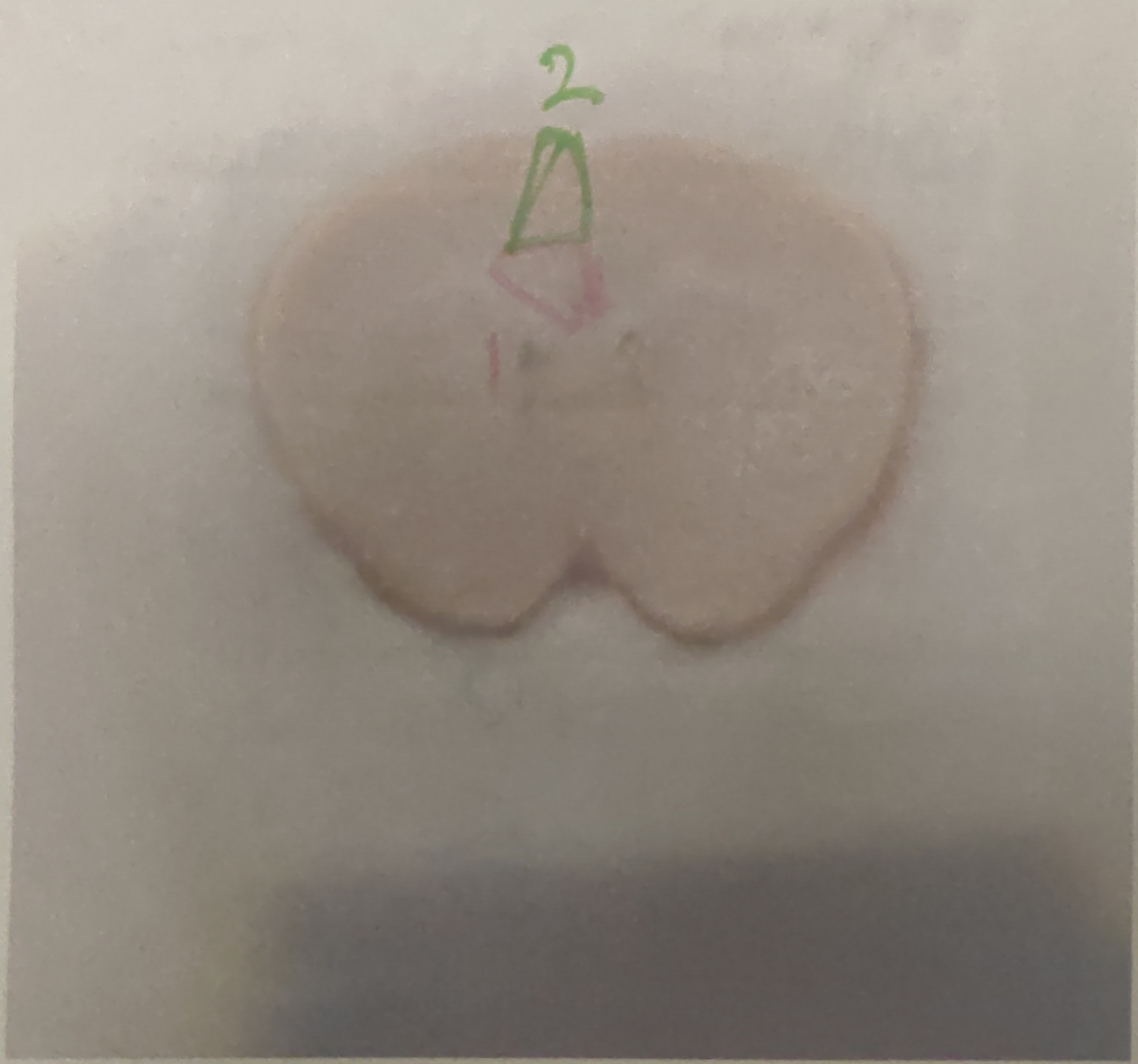

839168_21

*CL43

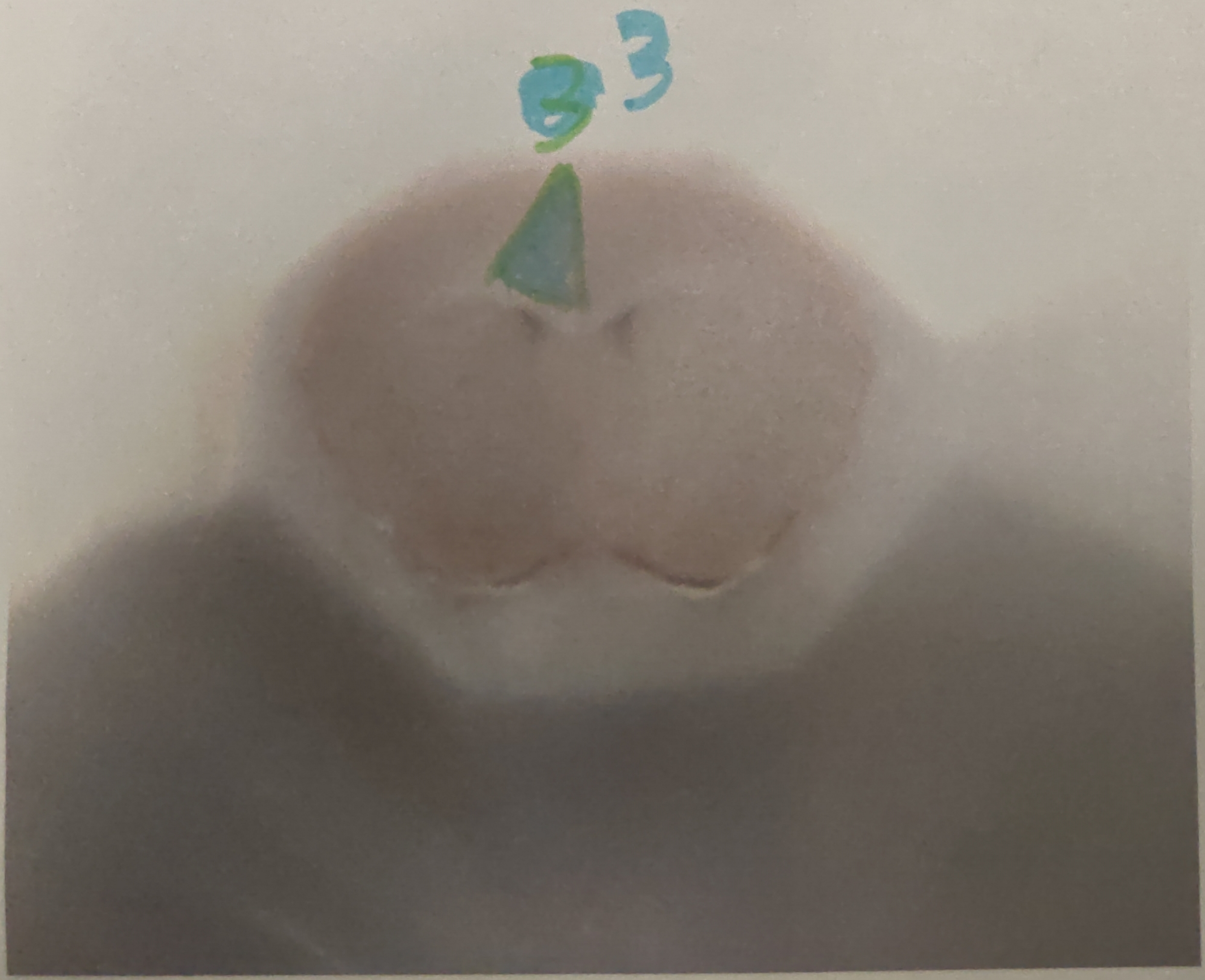

839168_3 1

CL47

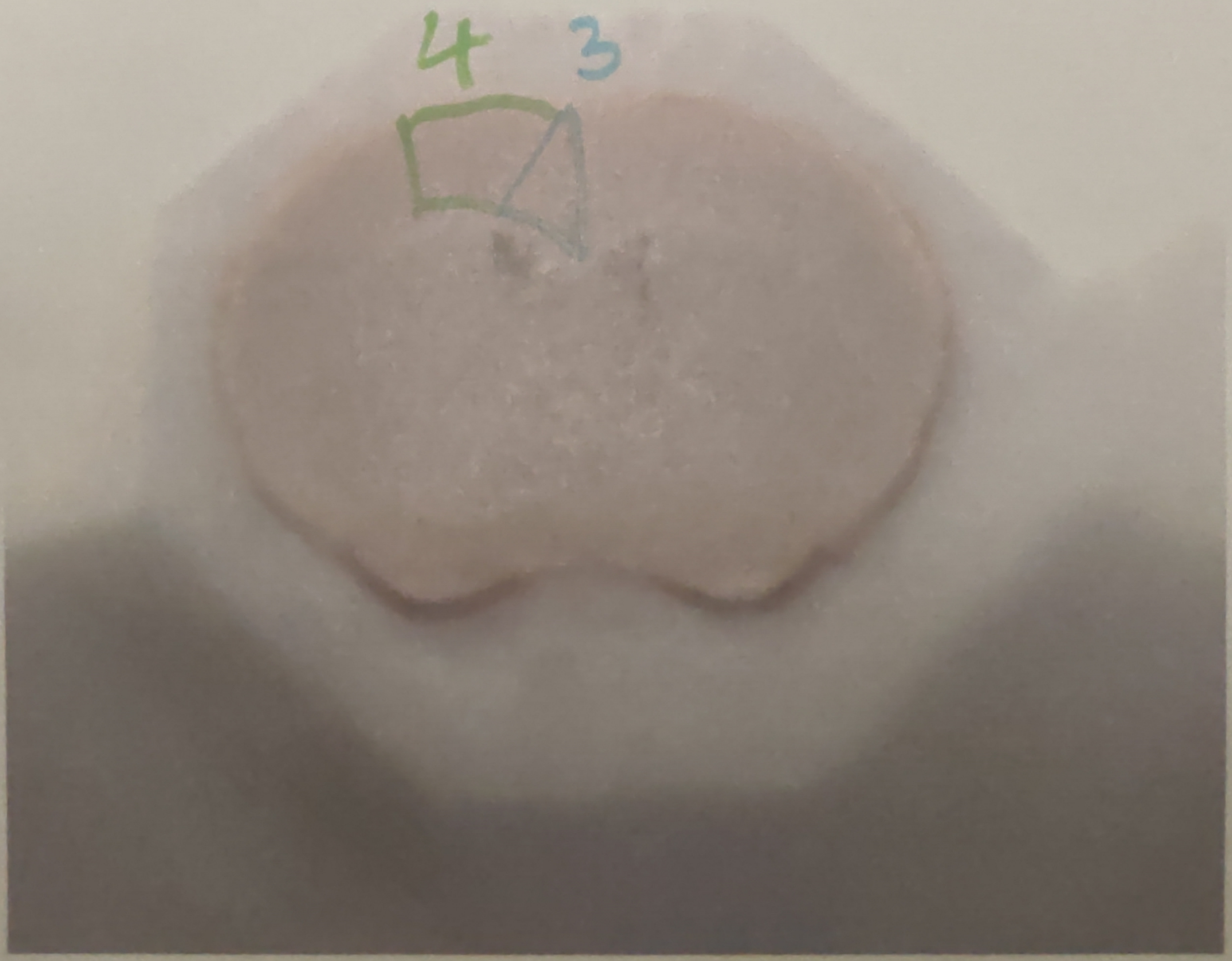

839168_4

CL50

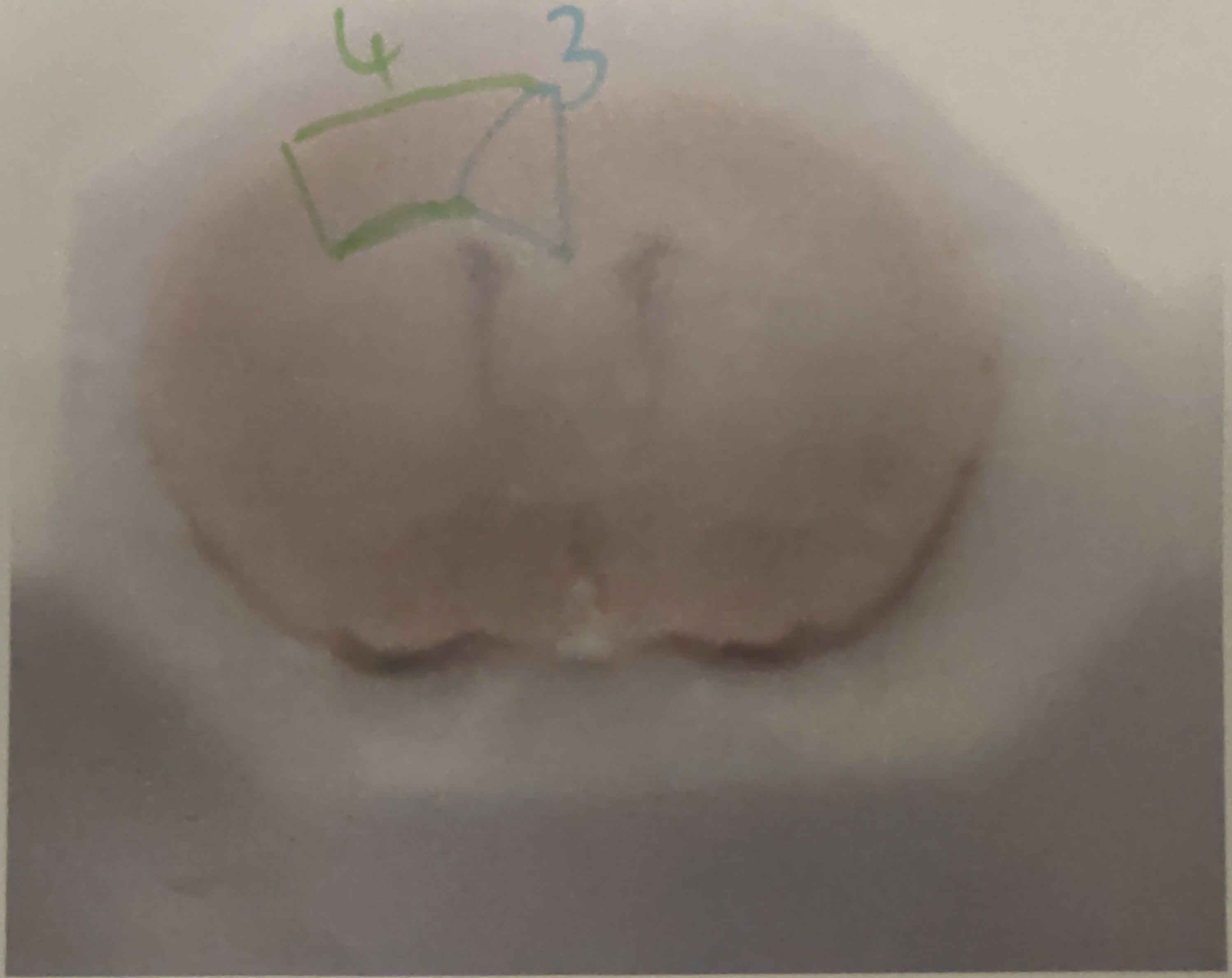

839168_5

CL53

Last section
from anterior

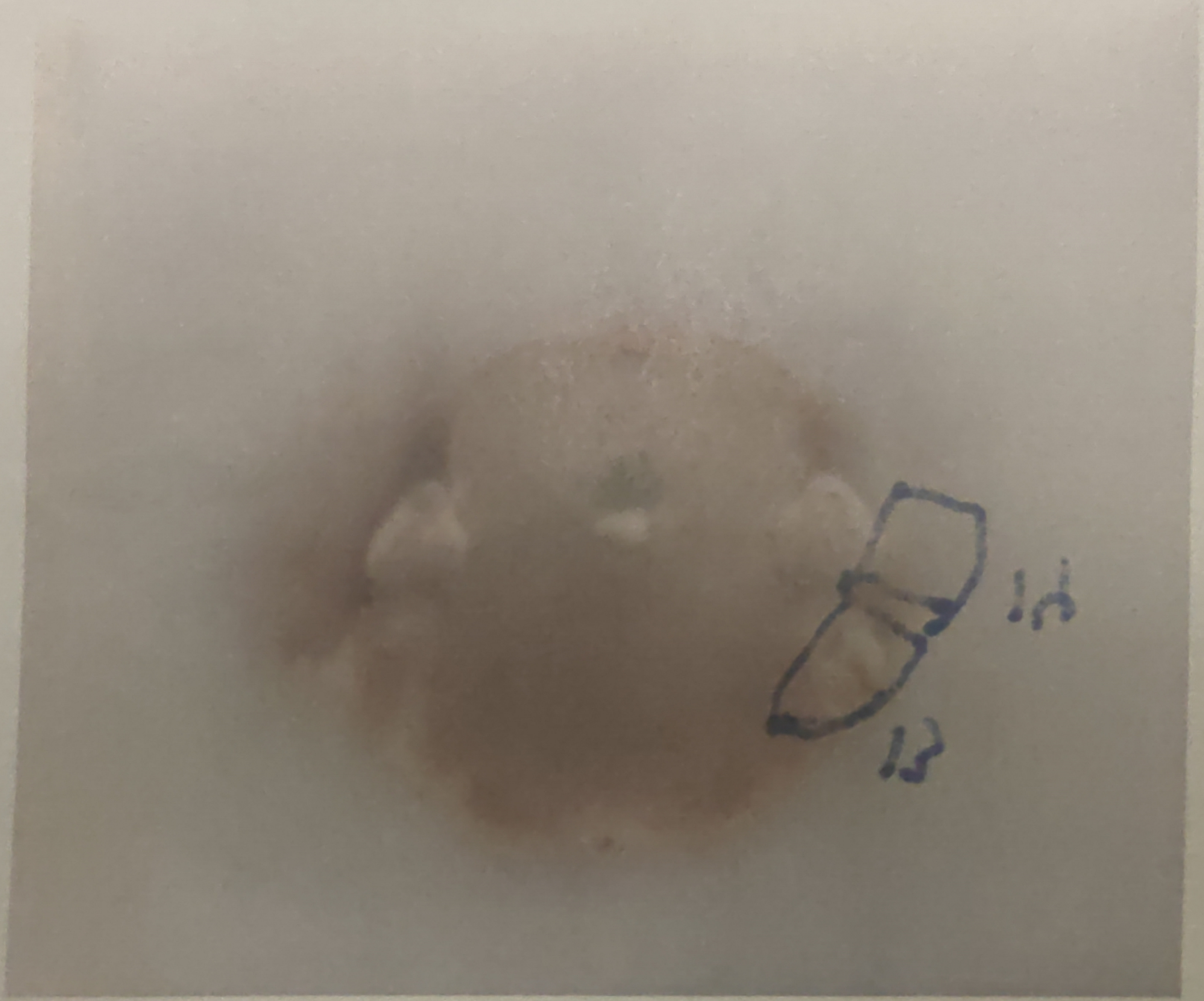

839168_2.1

CL 107

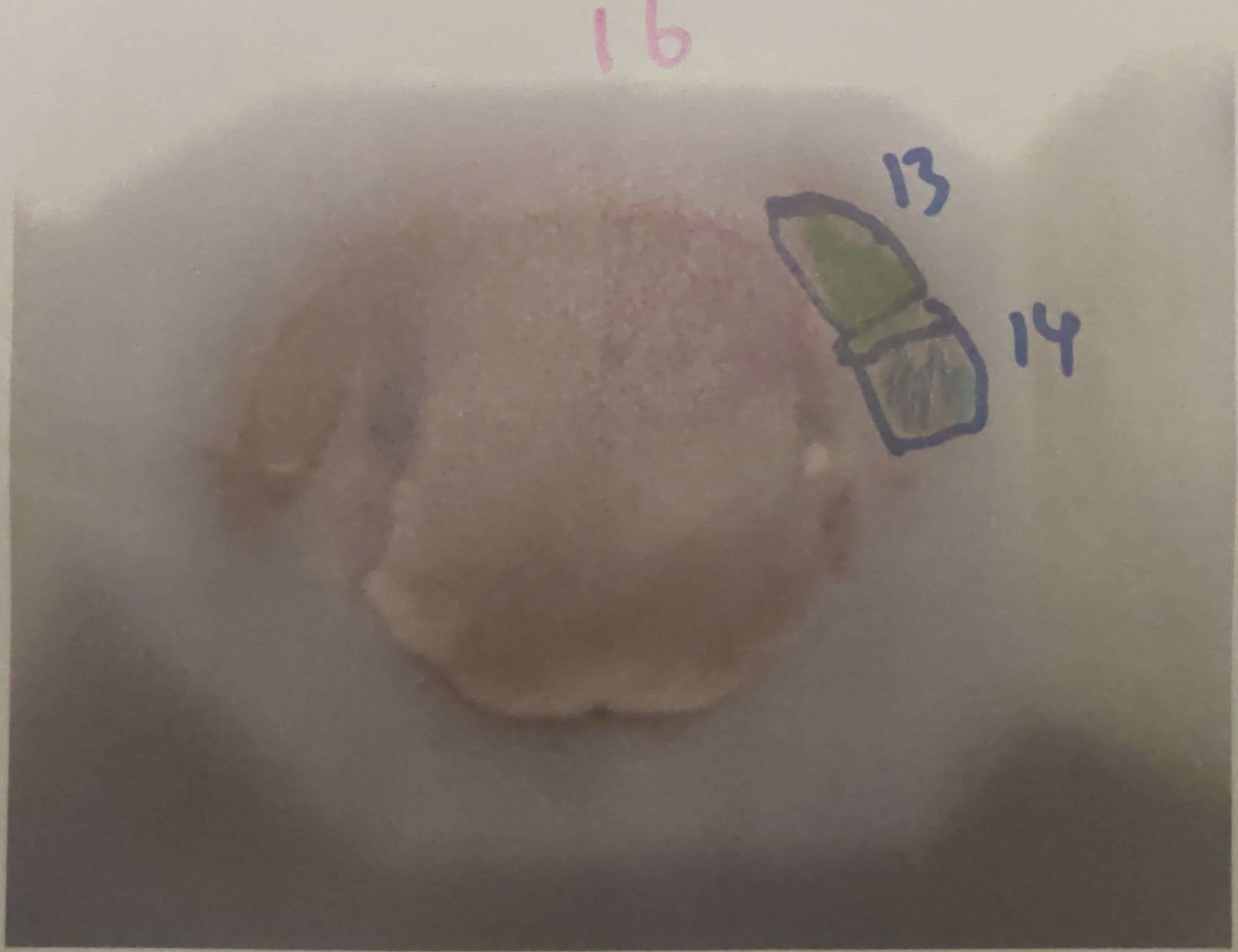

839168_2.2
1.tif

☆ CL~~[illegible]~~ 103

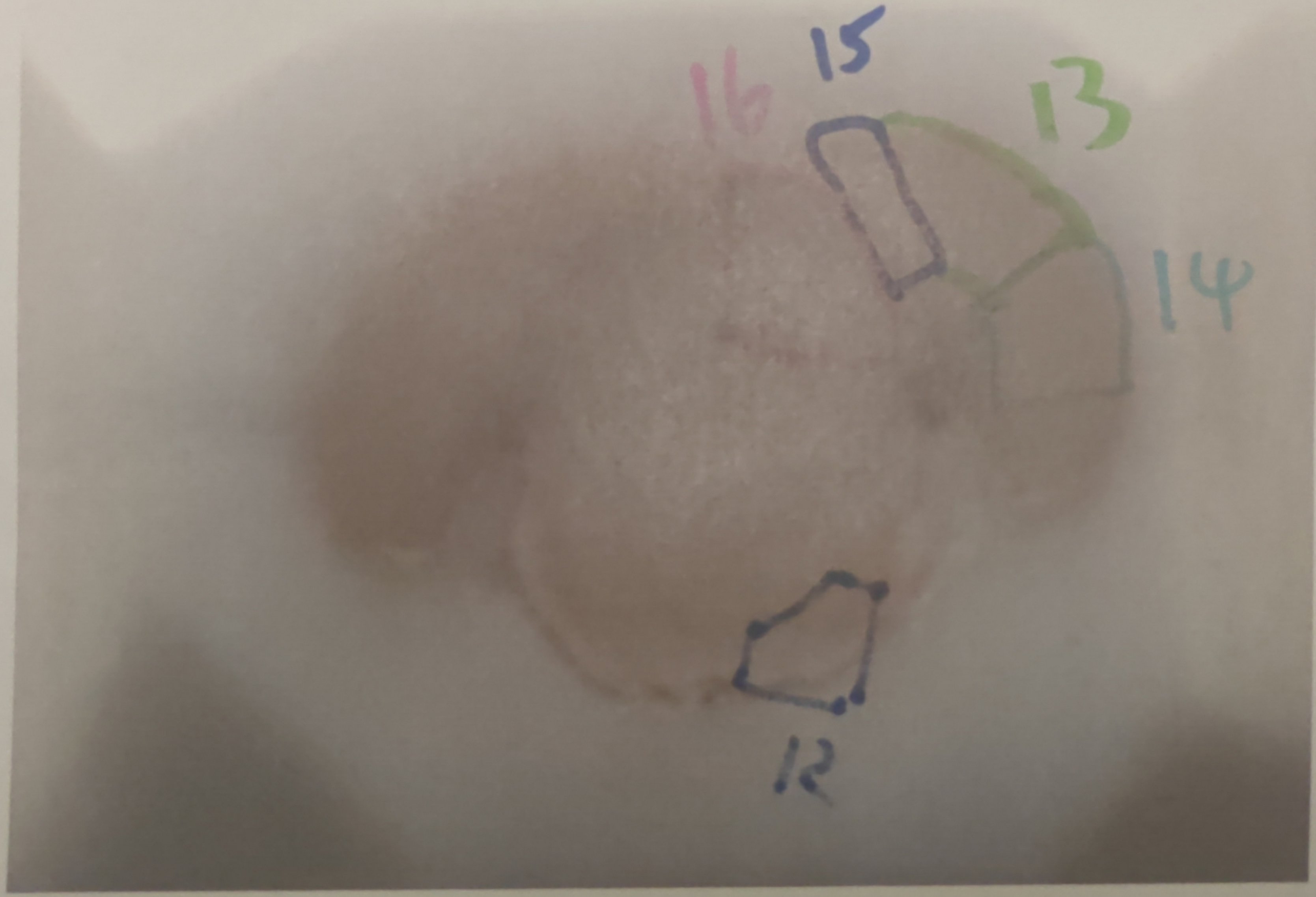

2.3

~~CL107 100?~~

CL95?

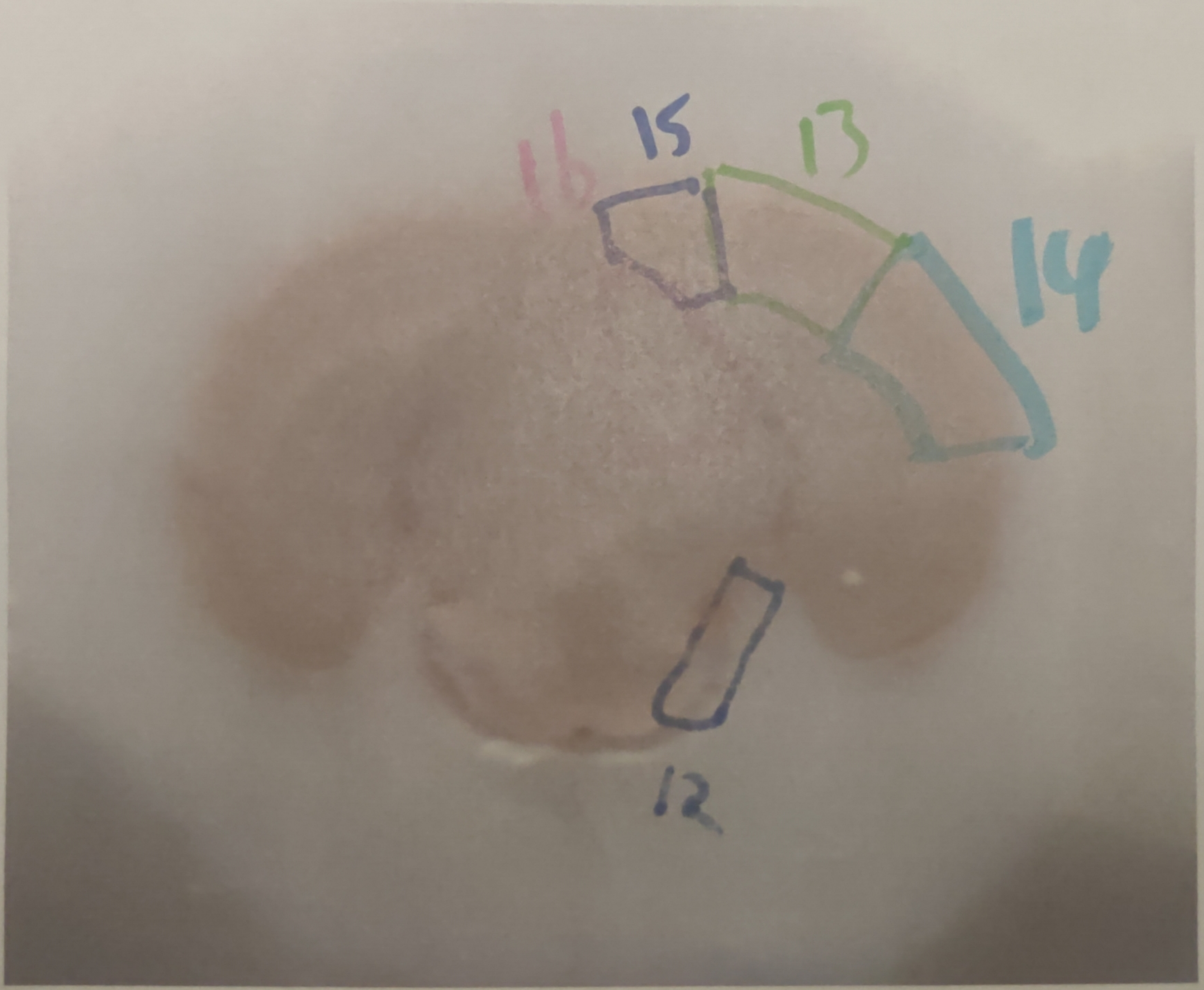

839168_2.4

*CL95

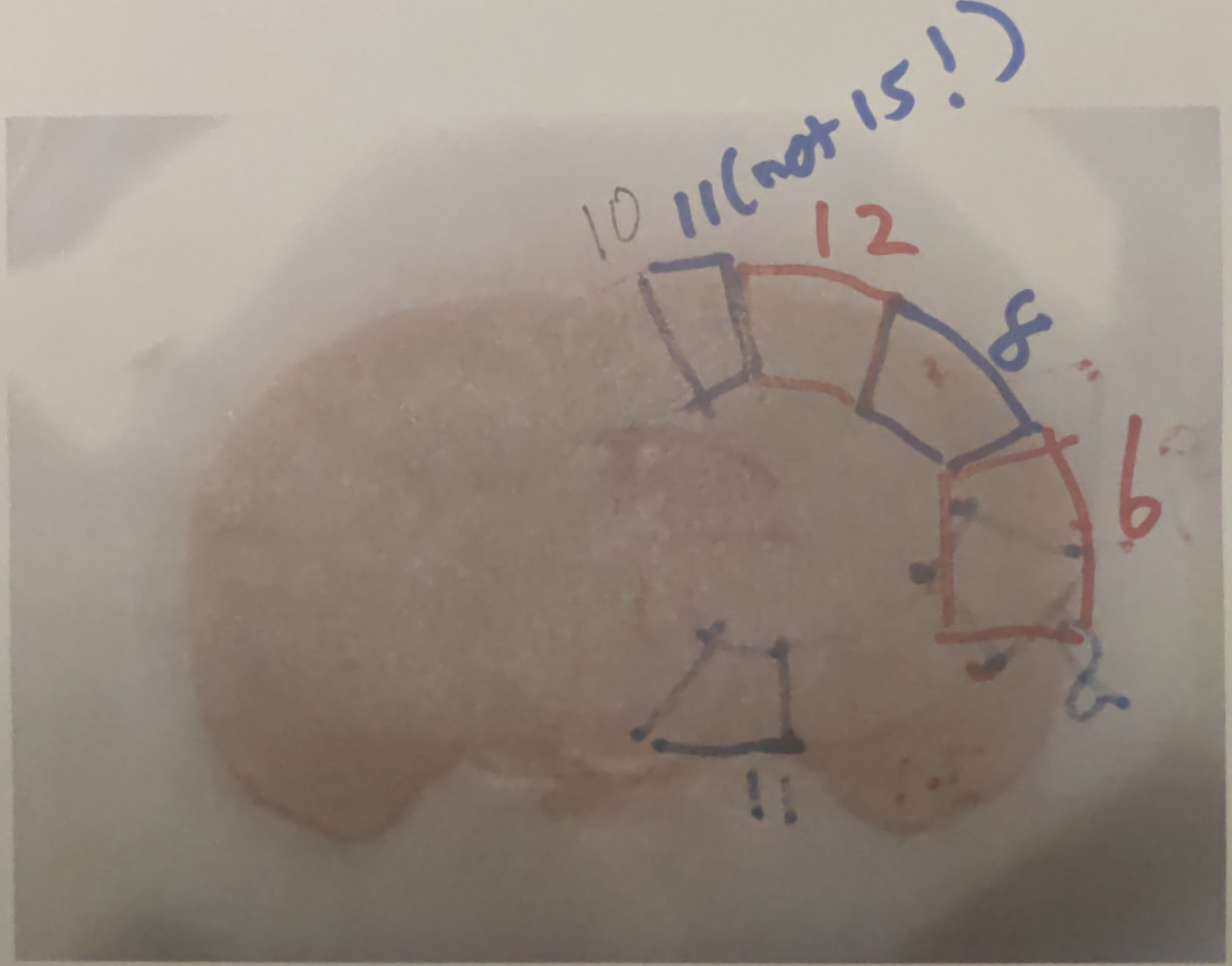

839168_2.5

CL 89?

Note the punched slices are collected P→A not A→P (as in 1st brain)
This causes 300μm shift in corresponding slice images

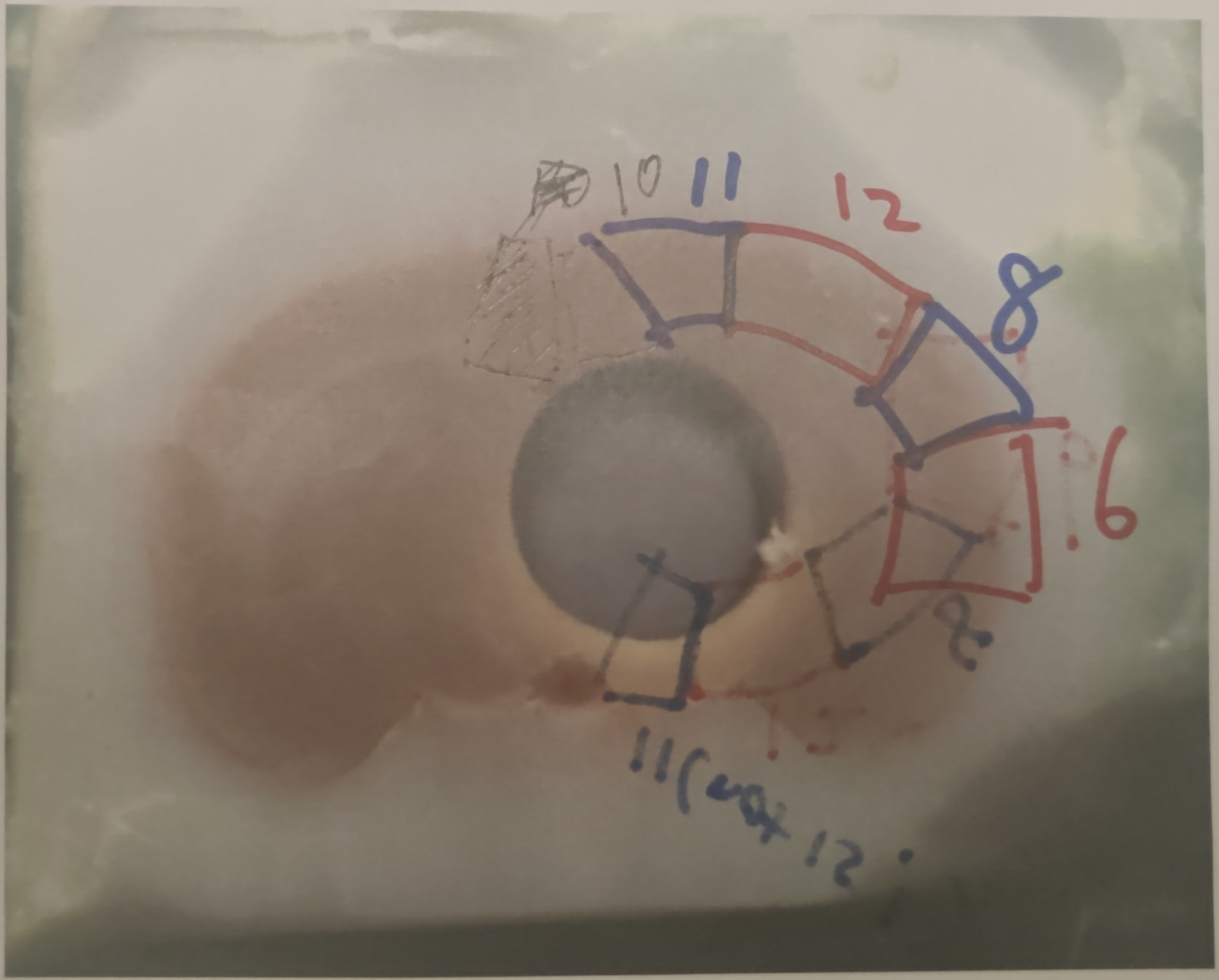

839168_3.1

* CL86?

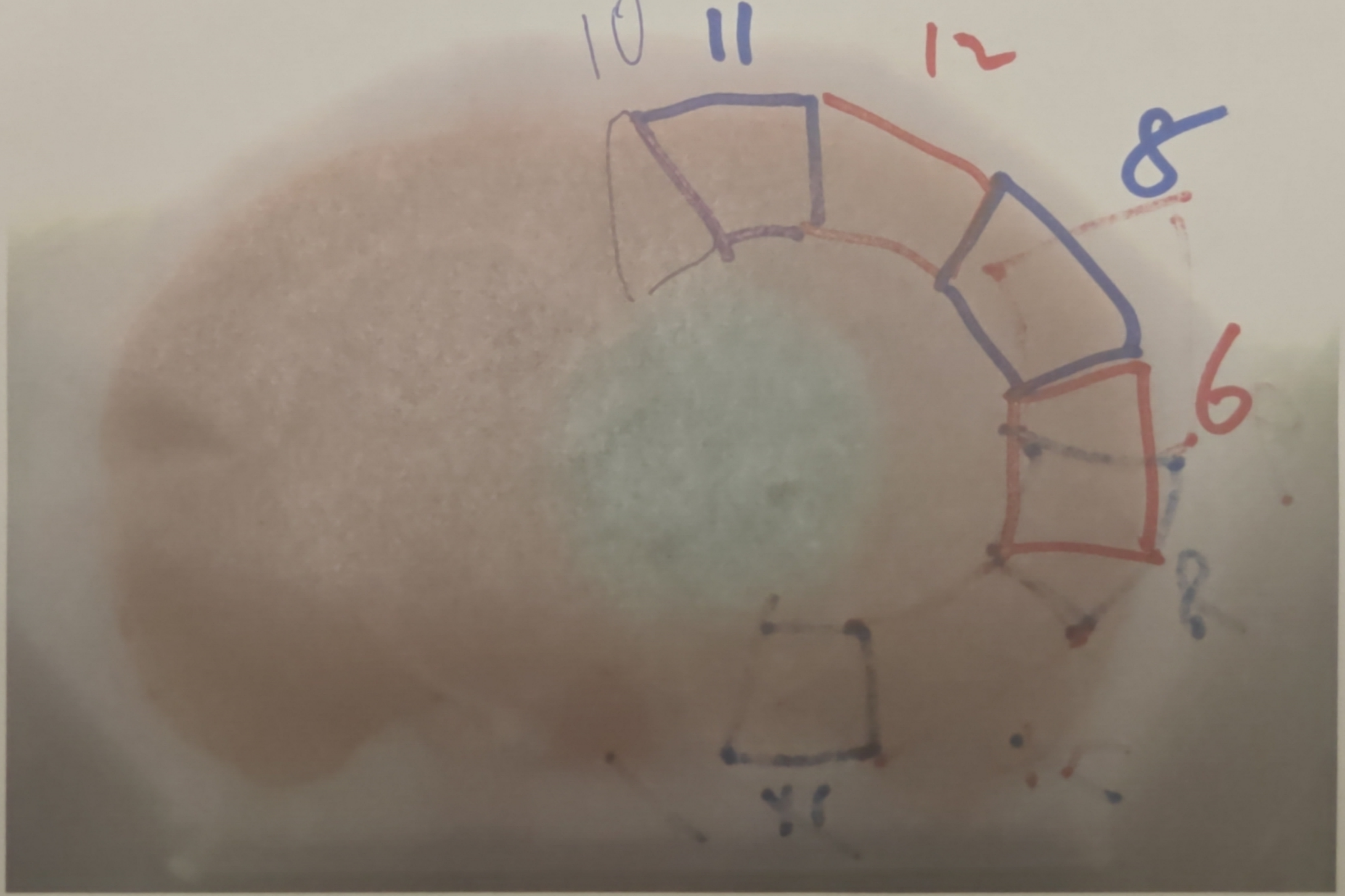

3.2

CL8[illegible]

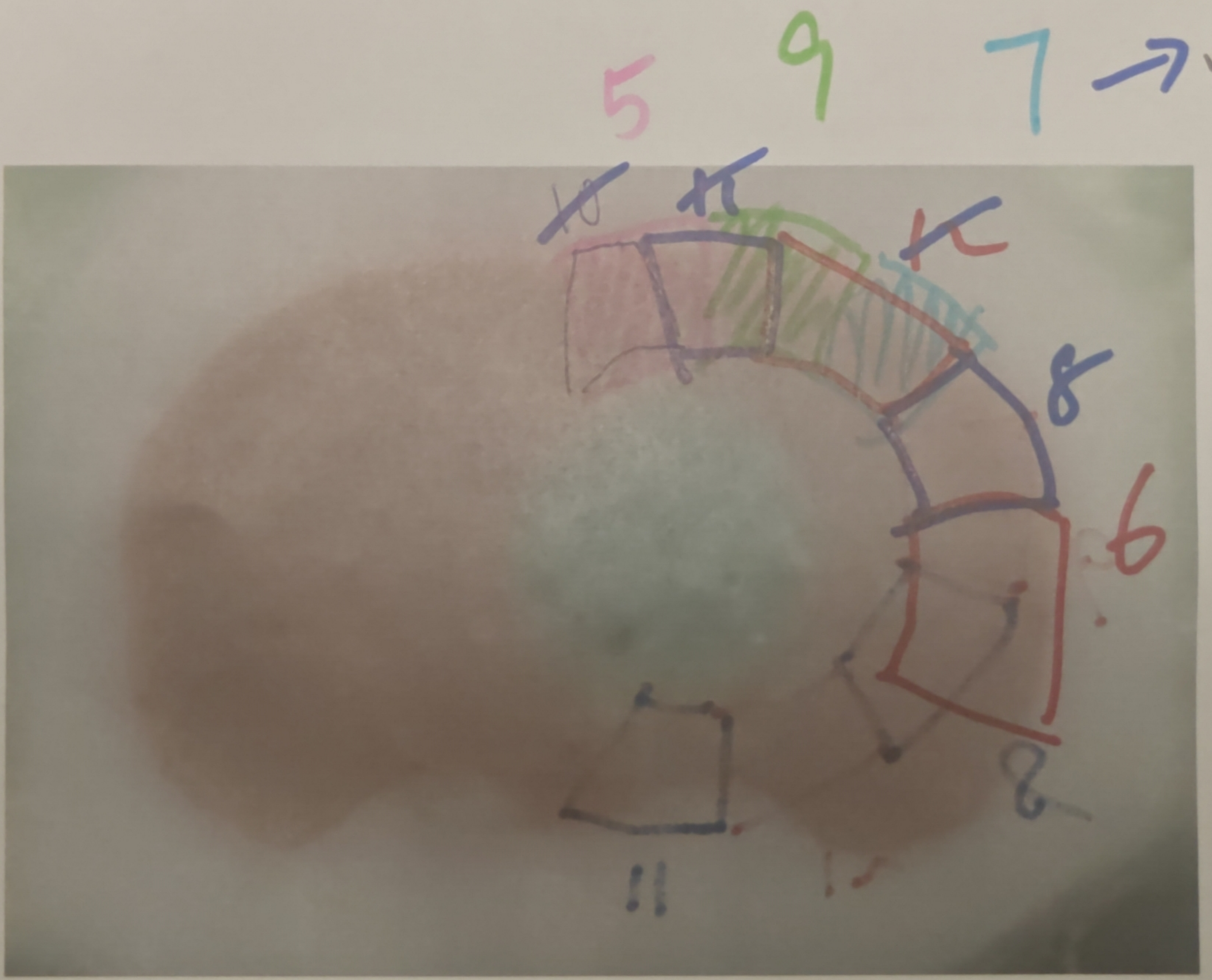

→ case because P→A, corresponds to CL 77 in first brain

3.3

CL 80

839168_3.4

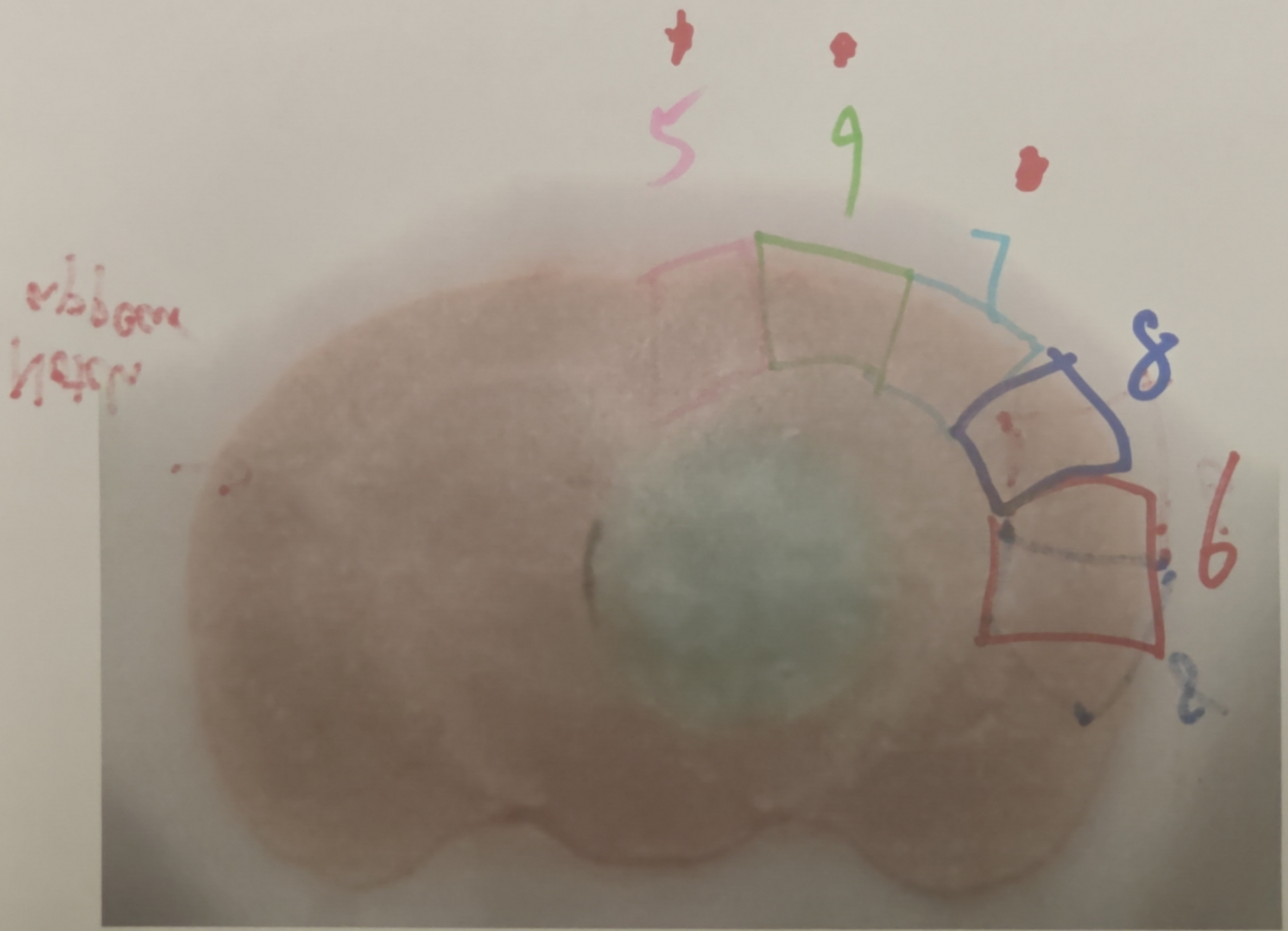

→ because this is P→A, so correspond to CL71 in previous brain

3.5

*CL74

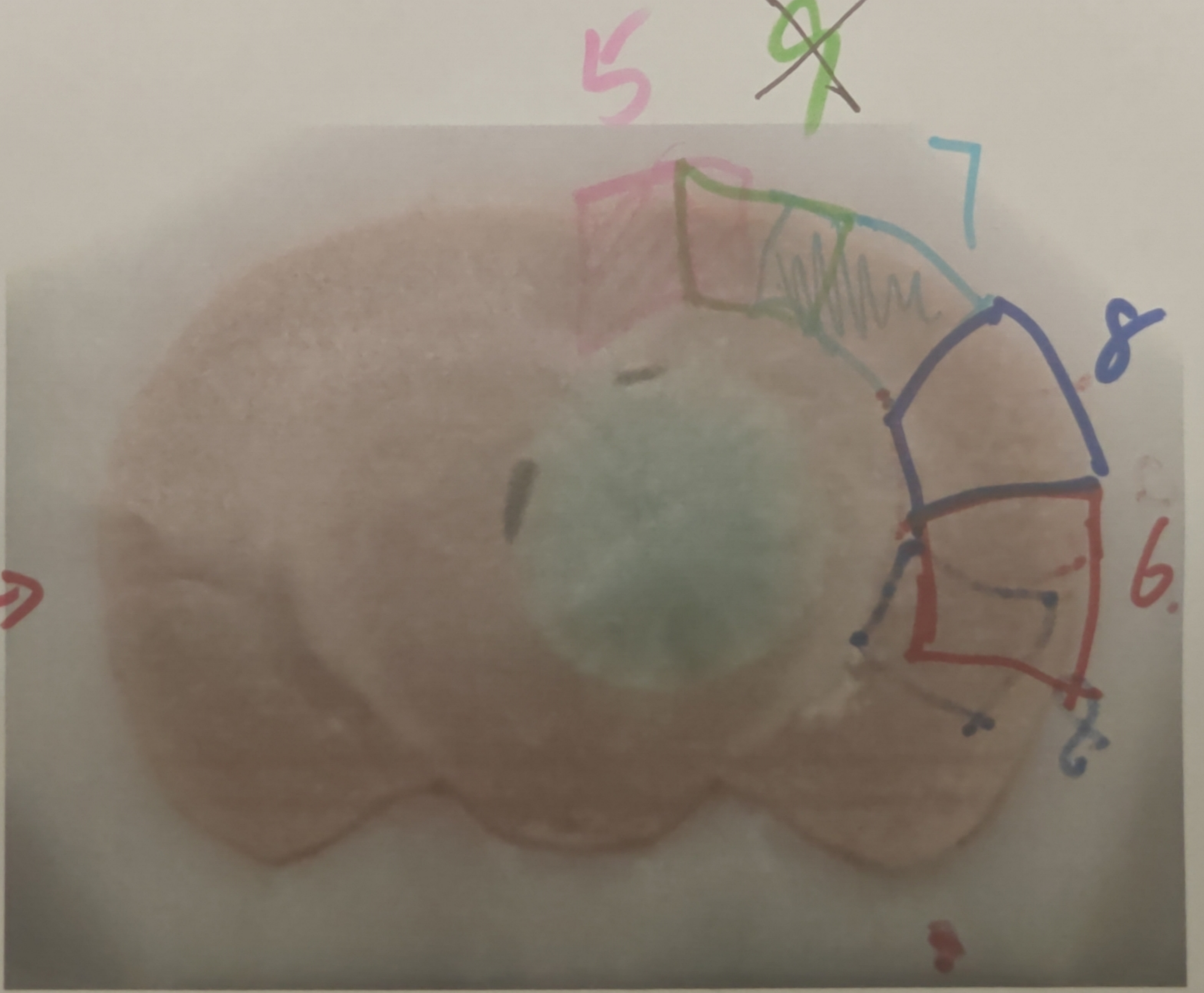

→ Notch appear

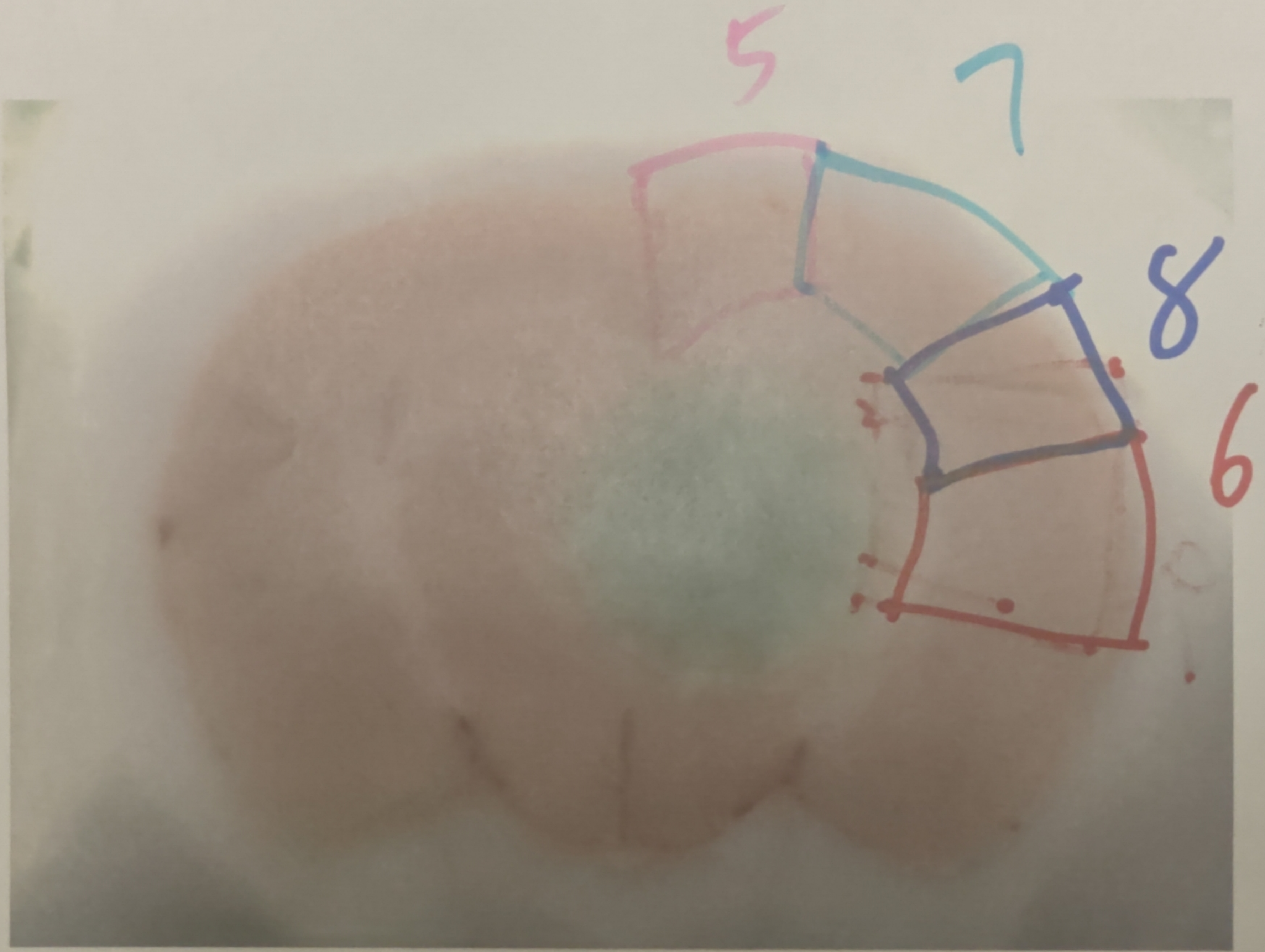

3.6

[illegible]

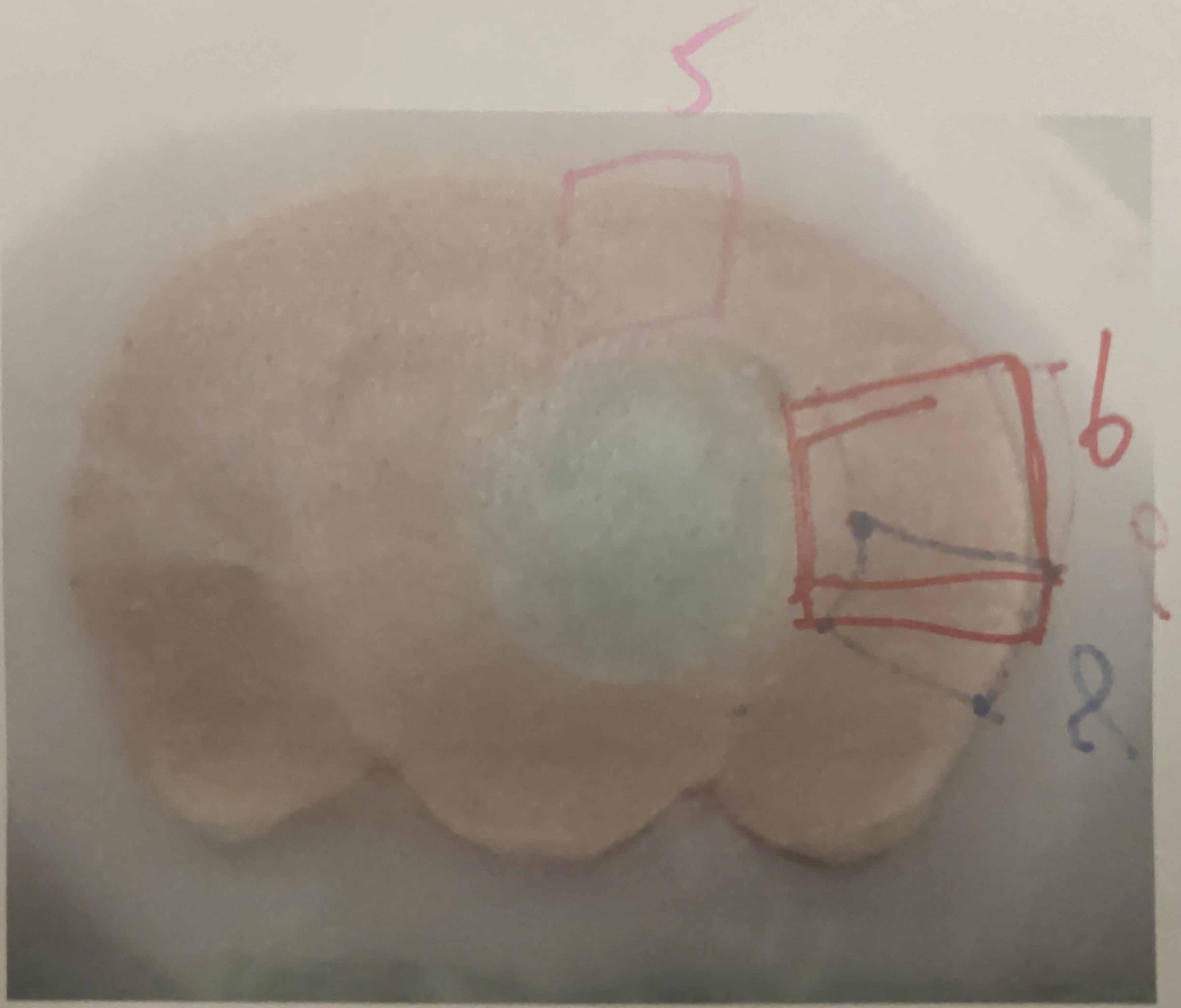

3.7

CL68

★★ see notes

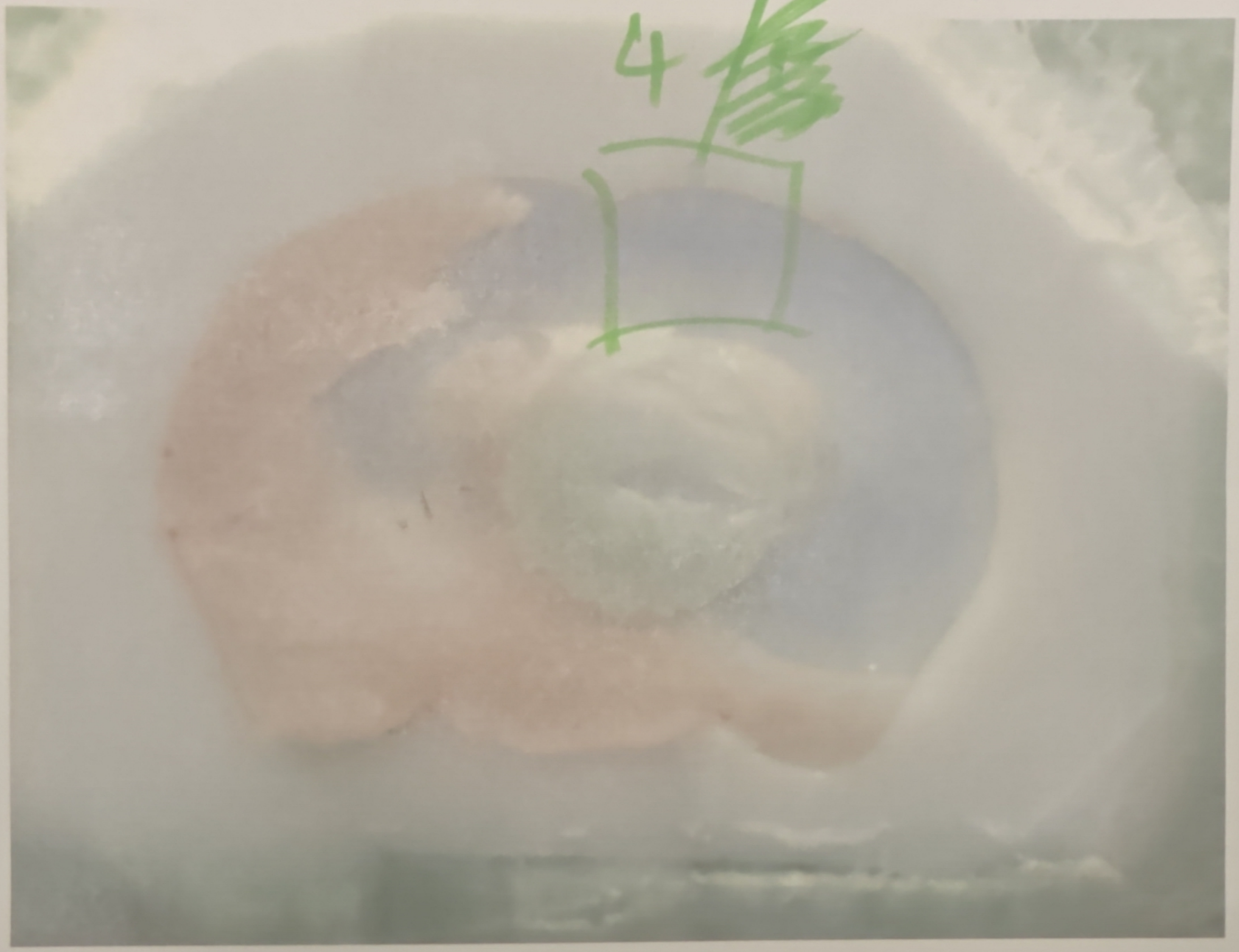

Note this brain the punched sections are cut P→A, whereas previous A→P, 300µm diff across slicing 3.8

CL 65

Missing 53–65 (1.2mm)

If last punched section is thick collect [4] not [5]

(4 is till 63

5 starts on 67

in first brain)
