# Supplementary figures and images for: Conserved and specialized features of thalamocortical wiring revealed by single-cell projection mapping in mouse and marmoset
Source: bioRxiv. 2026 Jul 8:2026.07.07.736957. Preprint. [Version 1] doi: 10.64898/2026.07.07.736957 (PMC13371098; doi:10.64898/2026.07.07.736957)

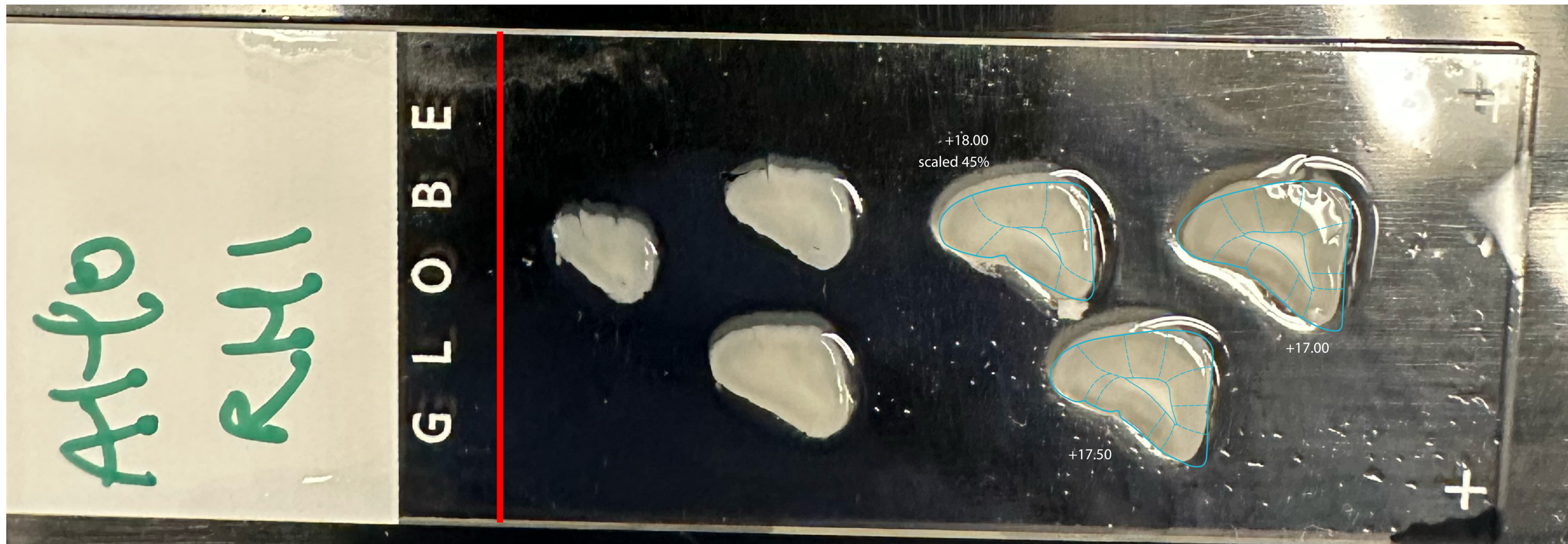

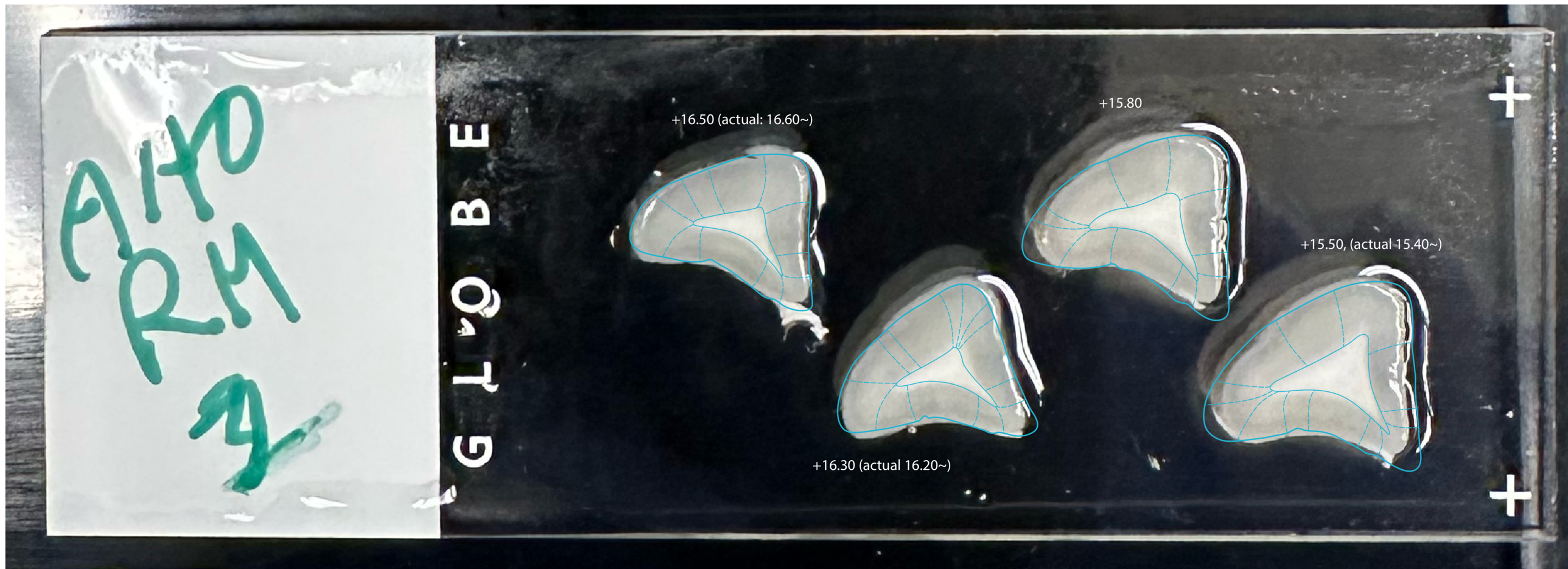

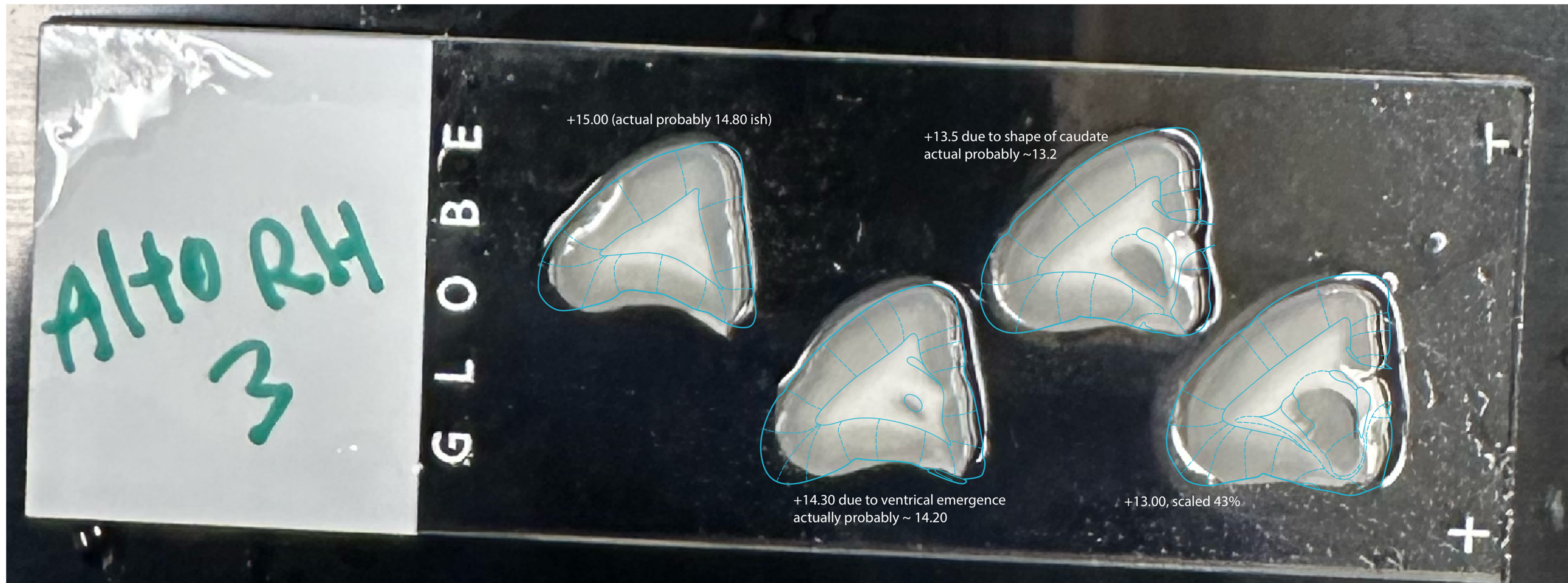

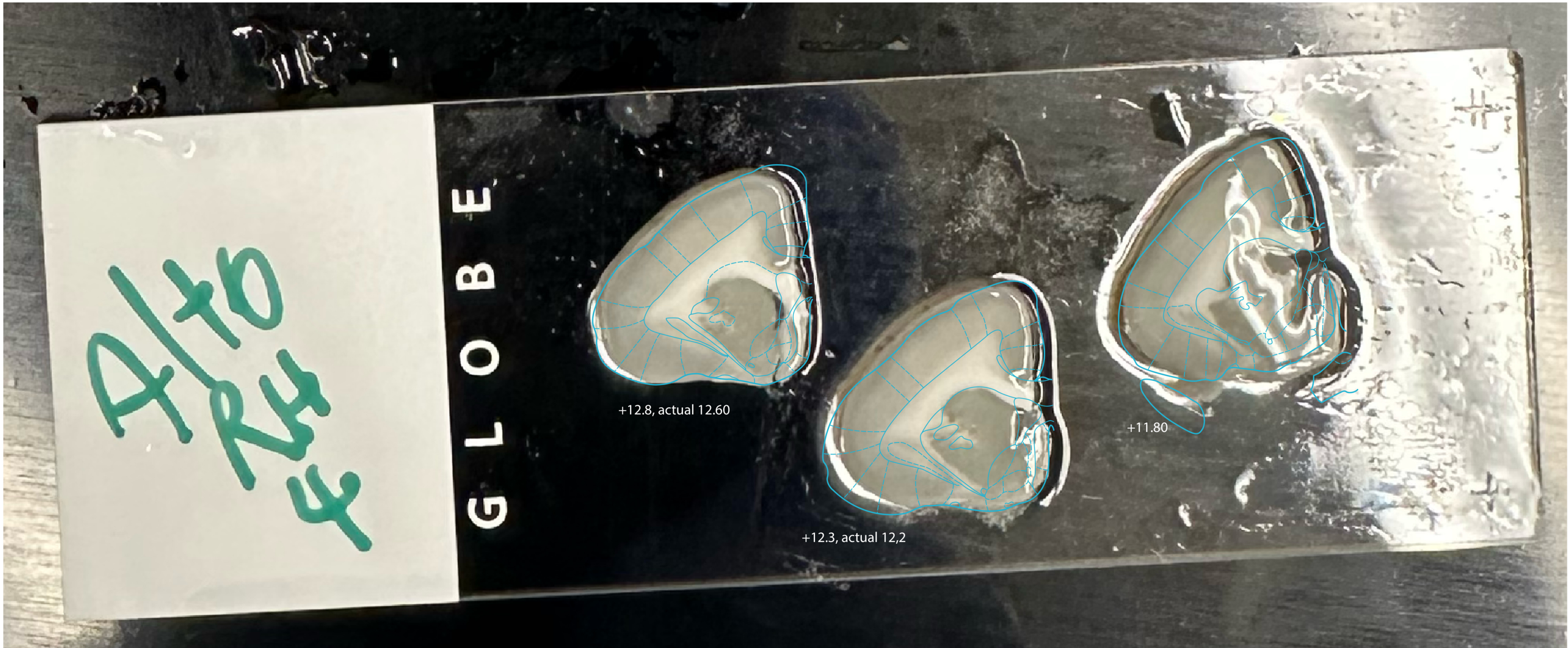

Alto\_RH\_4.jpg

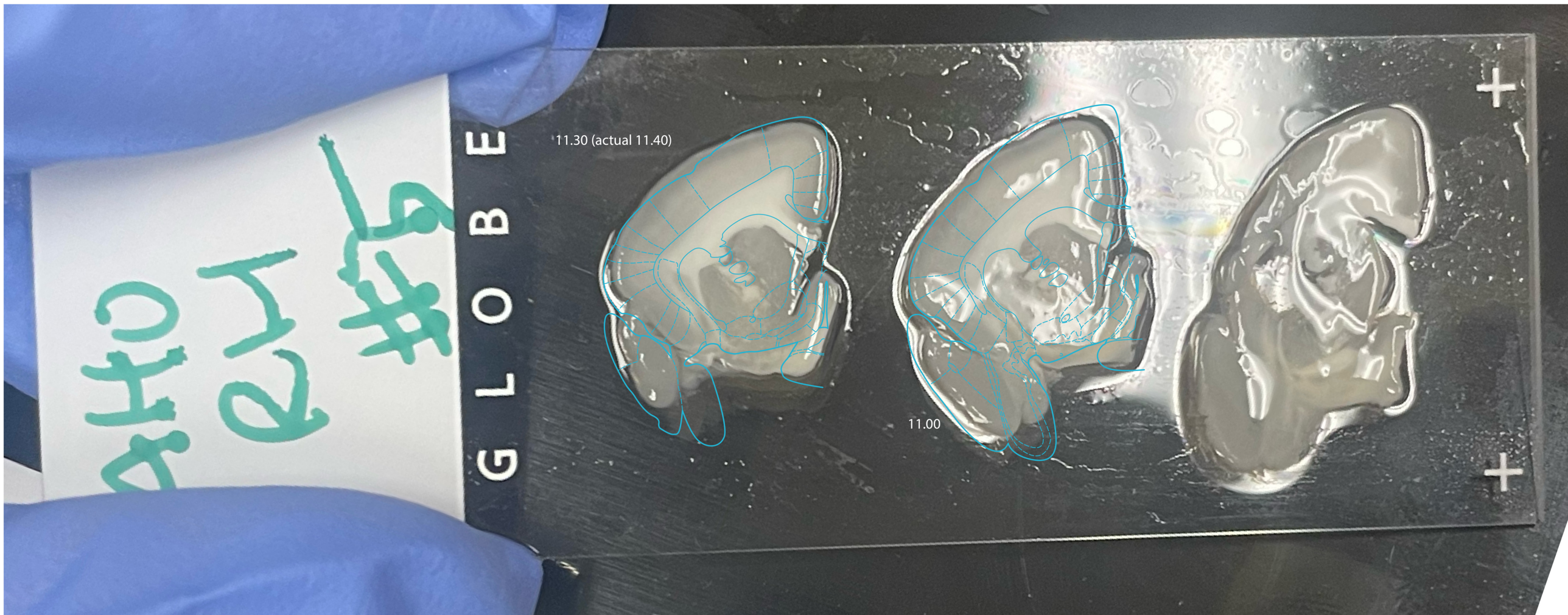

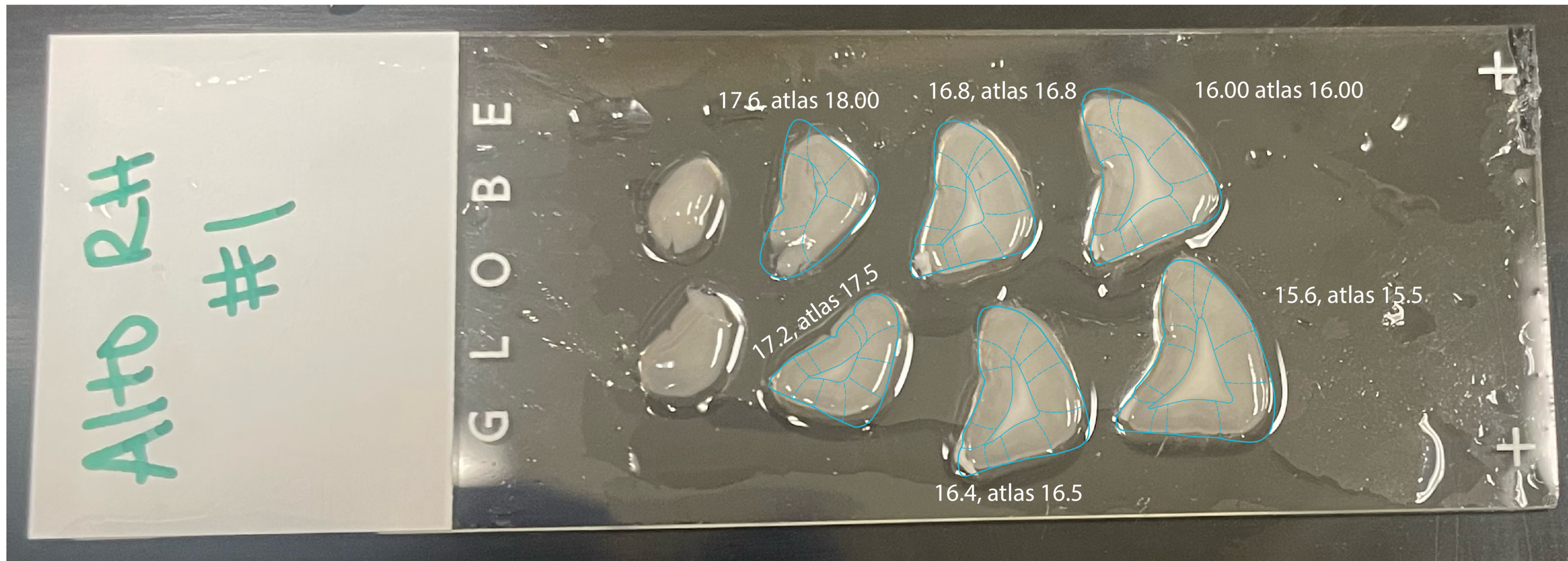

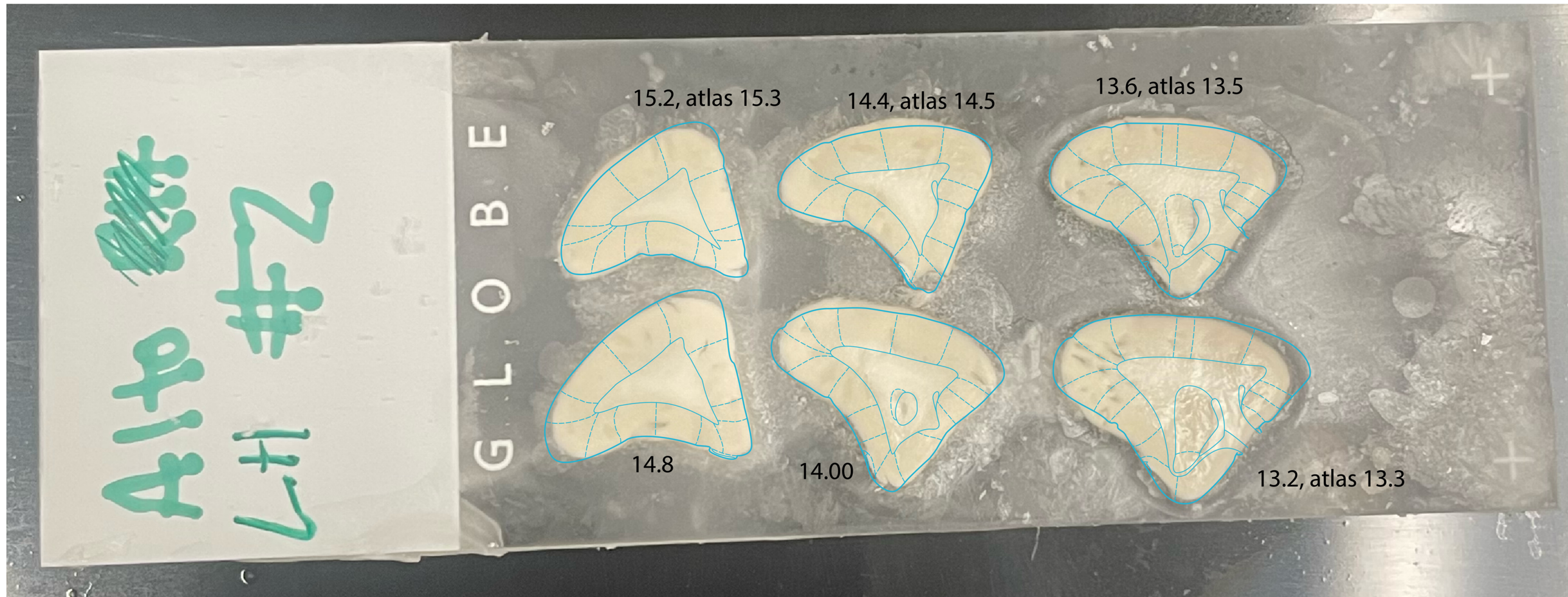

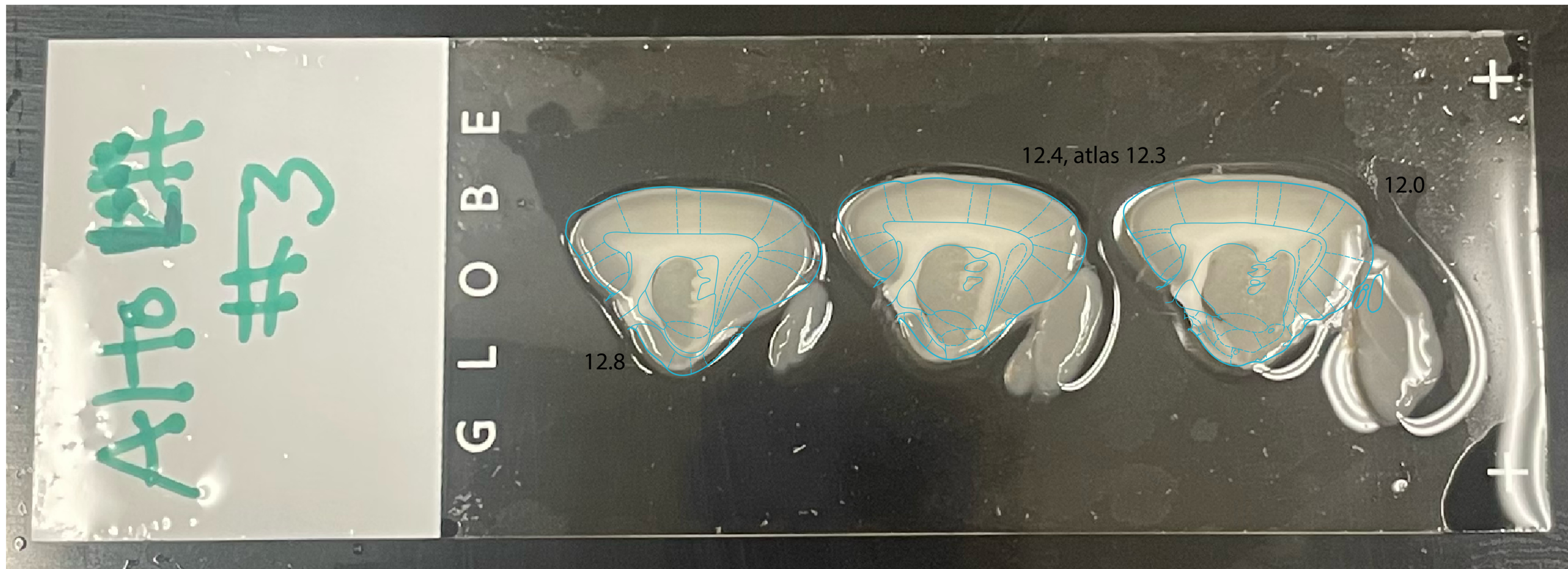

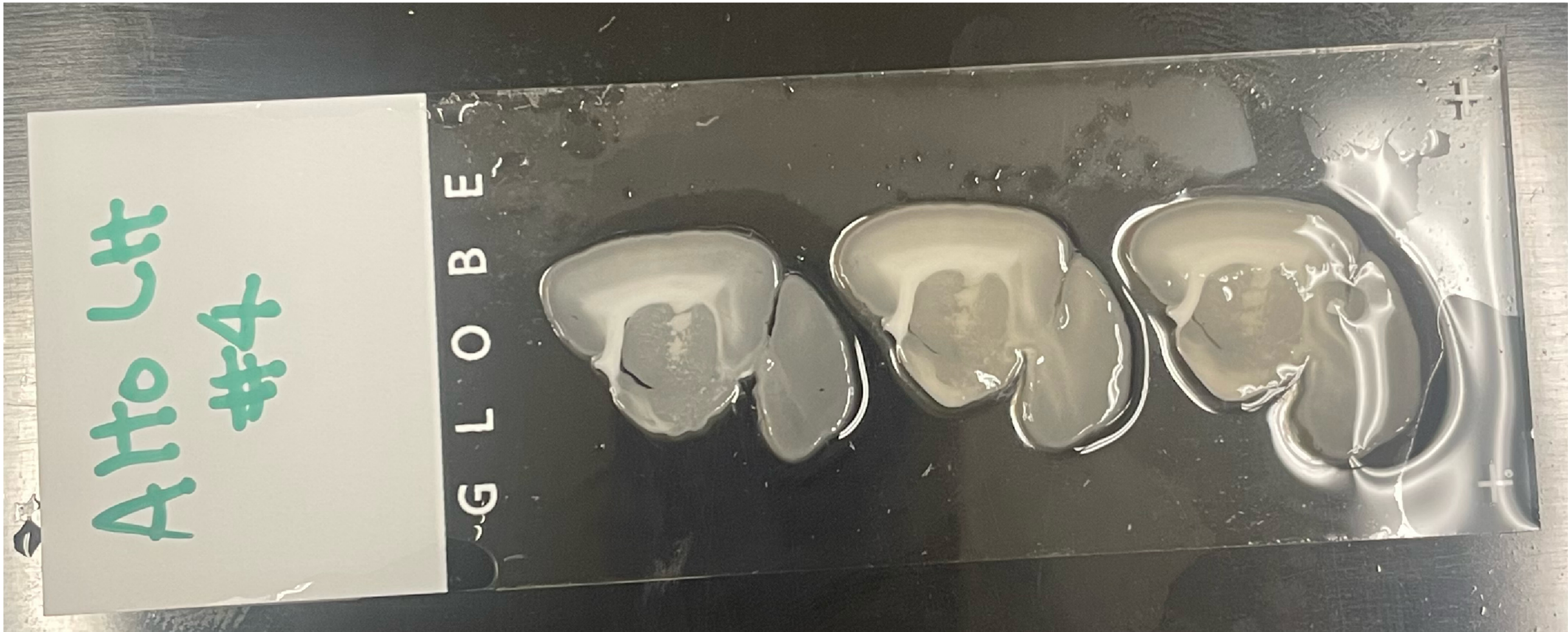

Alto\_LH\_4.jpg

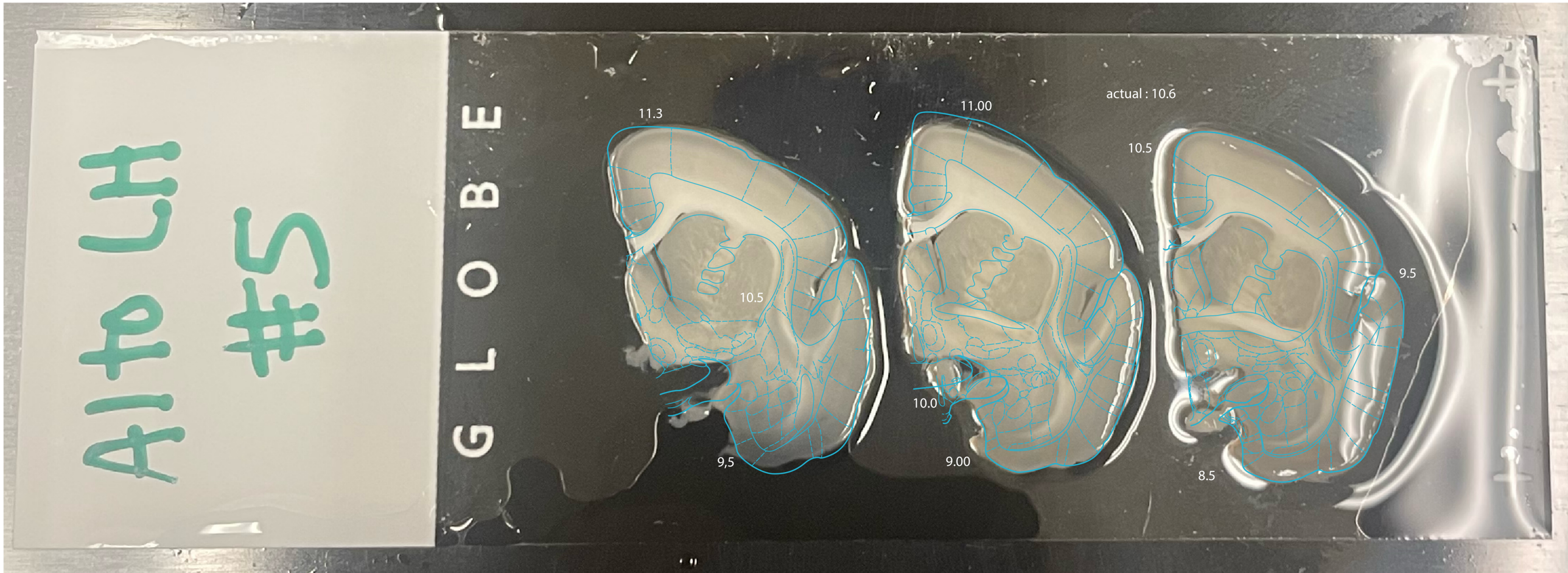

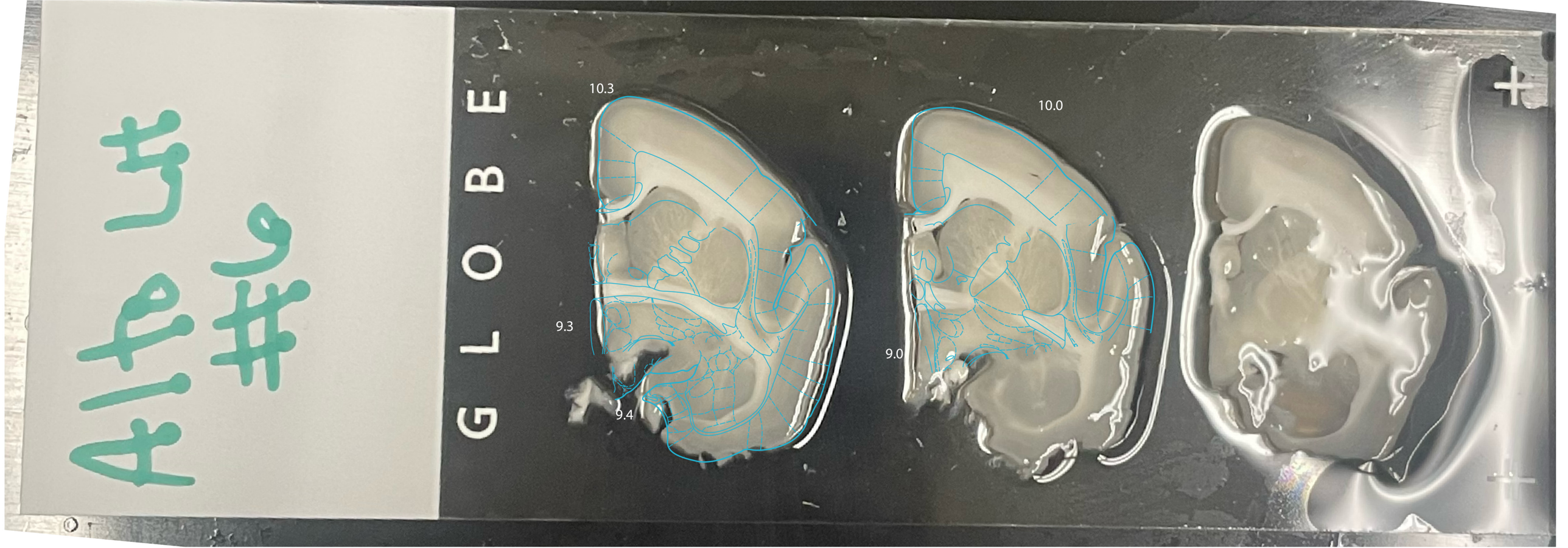

Supplement: Supplement 6 — Supplementary File 1: zip file containing dissection slice images for the BARseq experiments [file media-6.zip › Supplementary File 1/Marmoset Dissections/Alto_mapseq_dissection_annotations.pdf]

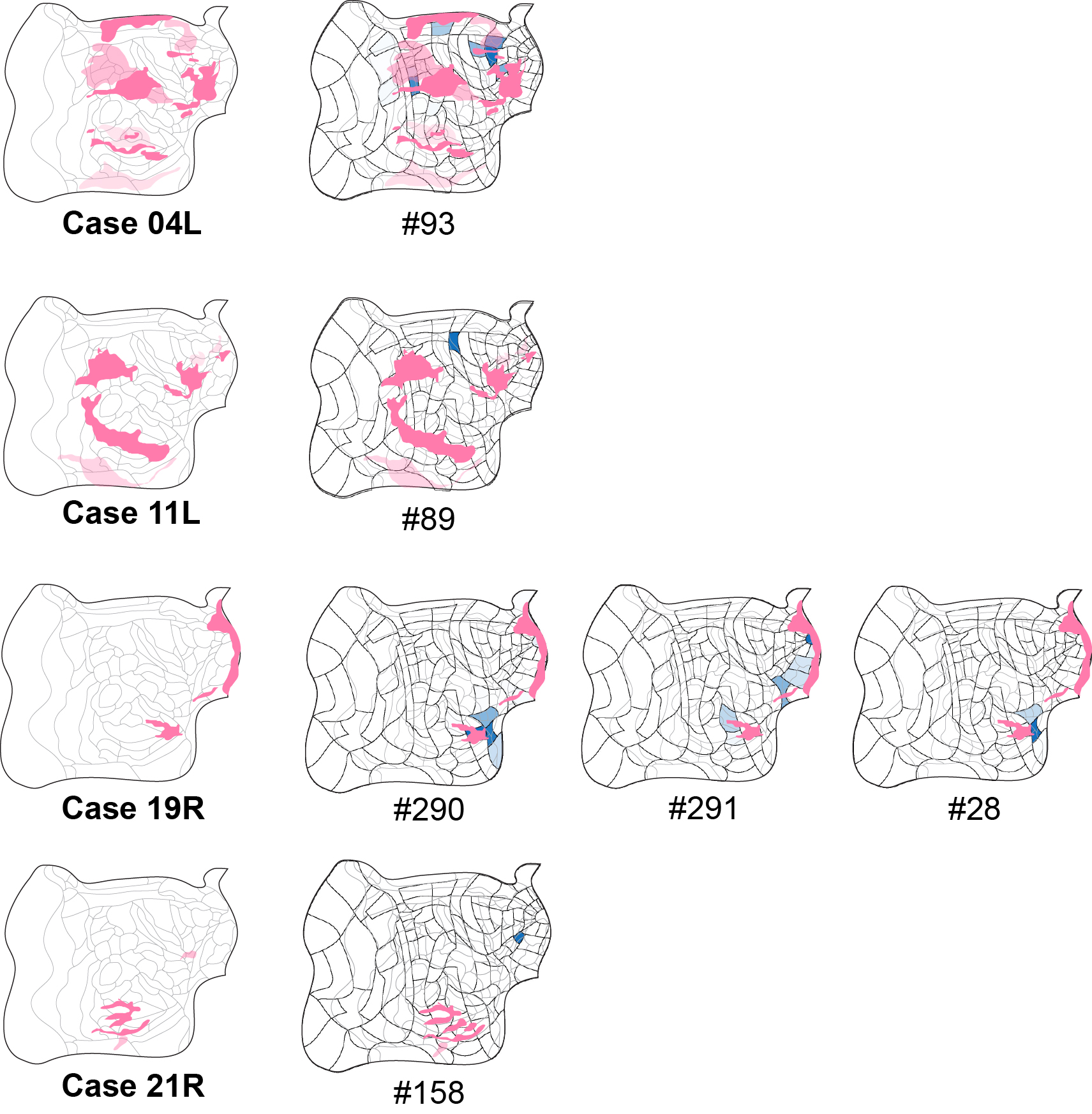

Supplement: Supplement 7 — Supplementary File 2: zip file containing flatmap representations of all matched neurons to (Córdoba-Claros et al., 2025b) [file media-7.zip › Supplementary File 2/case04L_11L_19R_21R.jpg]

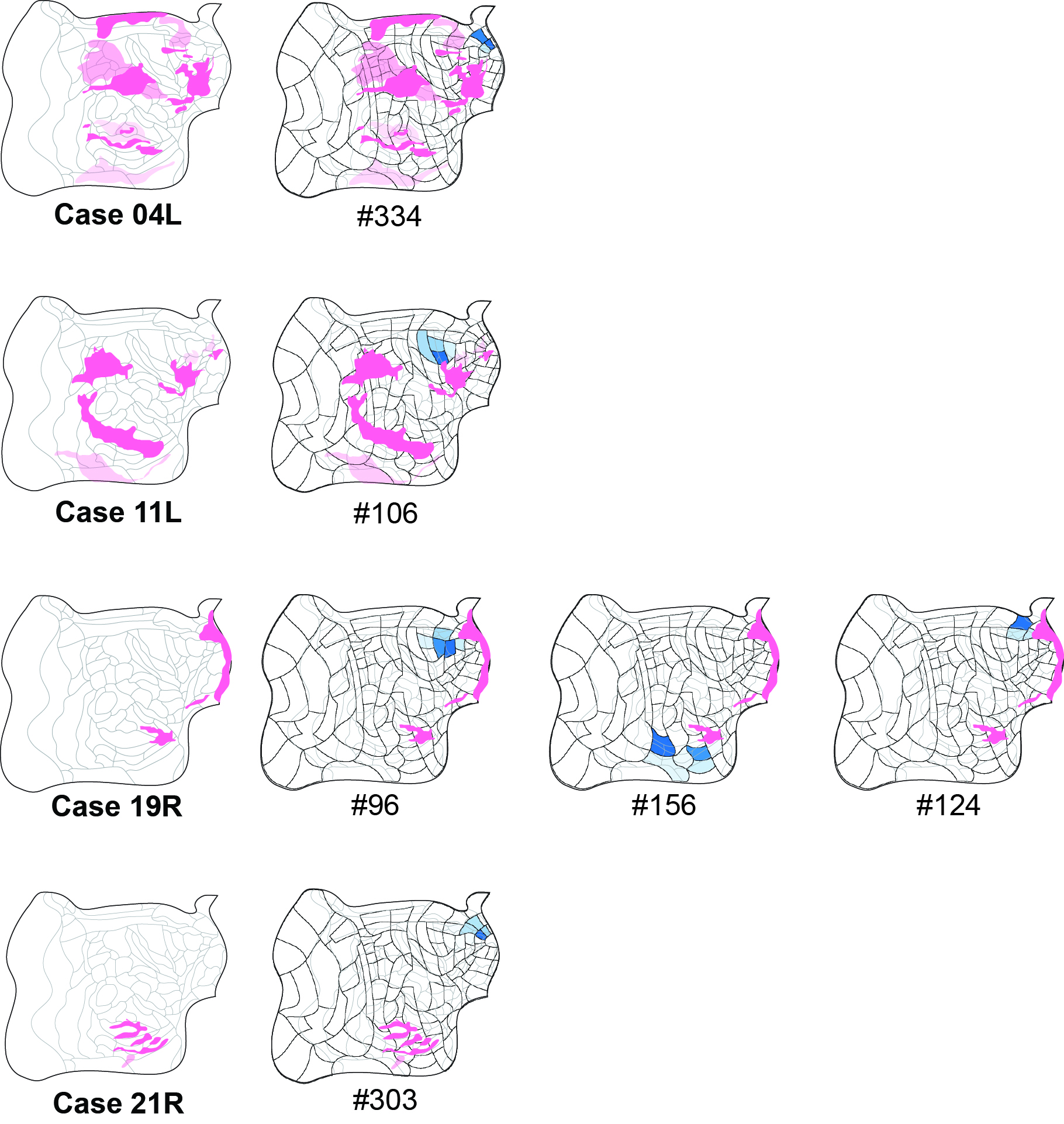

Supplement: Supplement 7 — Supplementary File 2: zip file containing flatmap representations of all matched neurons to (Córdoba-Claros et al., 2025b) [file media-7.zip › Supplementary File 2/case04L_11L_19R_21Rrandom.jpg]

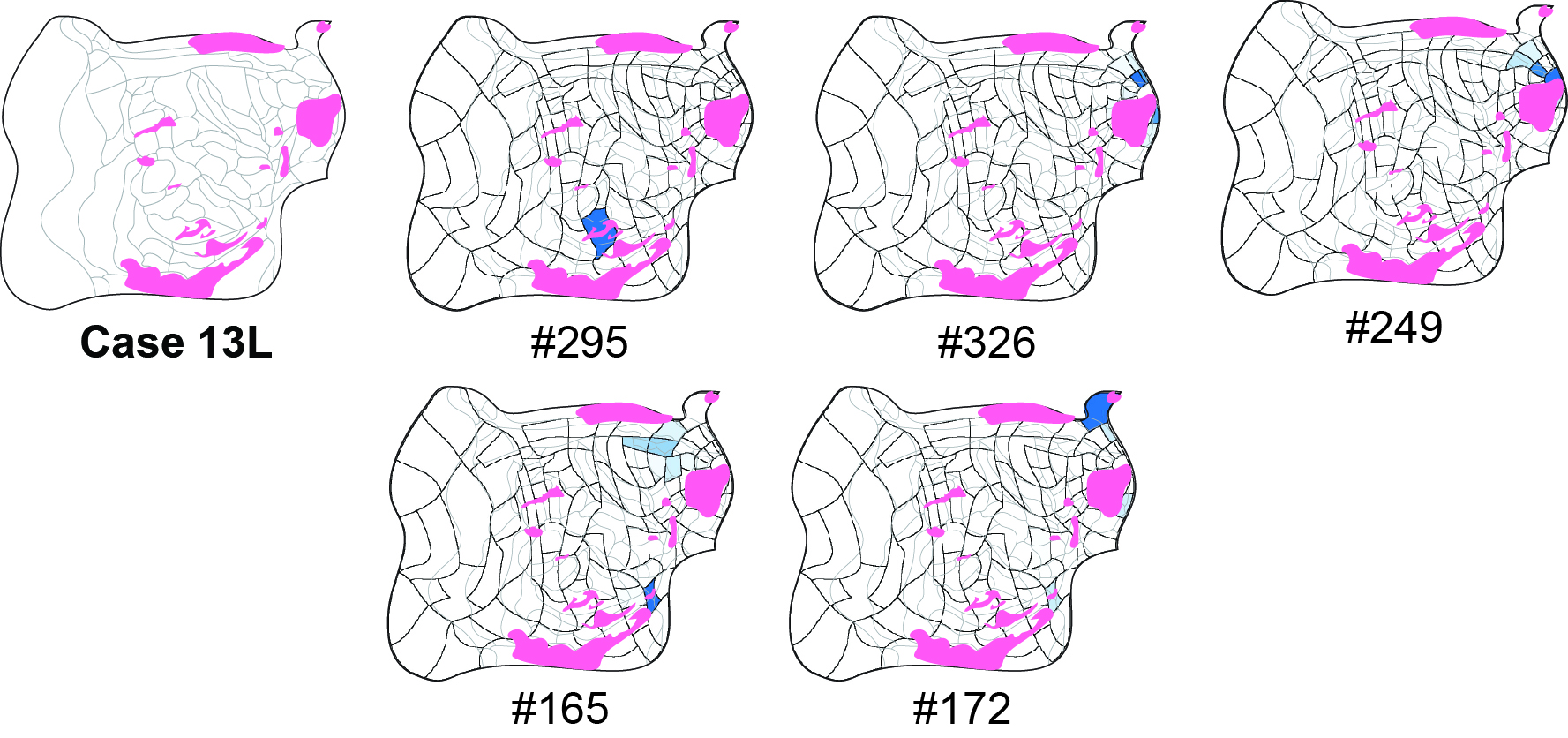

Supplement: Supplement 7 — Supplementary File 2: zip file containing flatmap representations of all matched neurons to (Córdoba-Claros et al., 2025b) [file media-7.zip › Supplementary File 2/case13L.jpg]

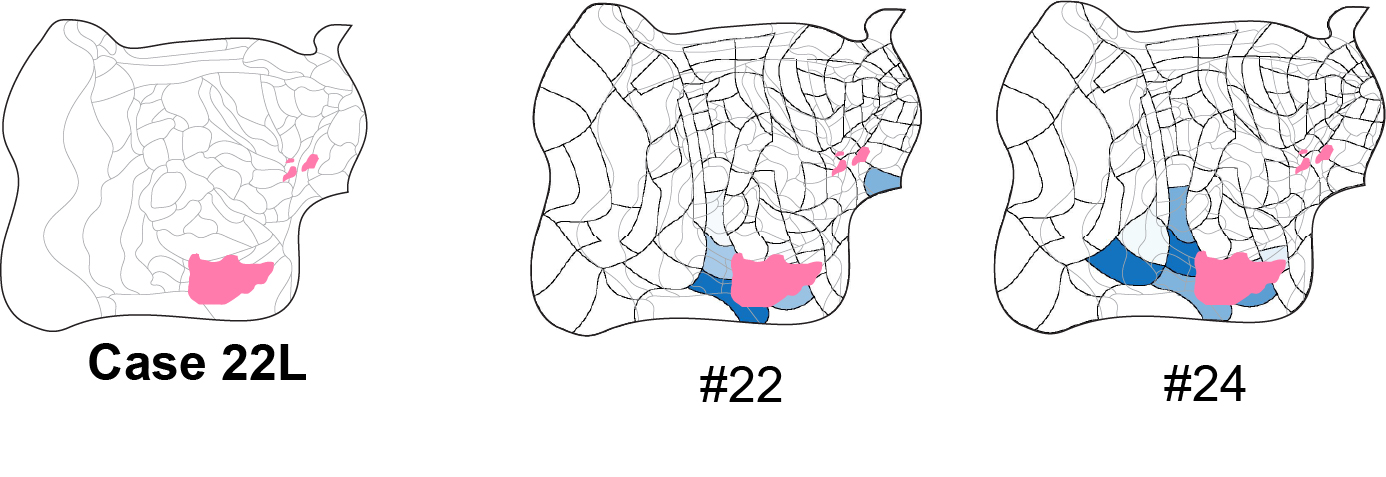

Supplement: Supplement 7 — Supplementary File 2: zip file containing flatmap representations of all matched neurons to (Córdoba-Claros et al., 2025b) [file media-7.zip › Supplementary File 2/case22L.jpg]

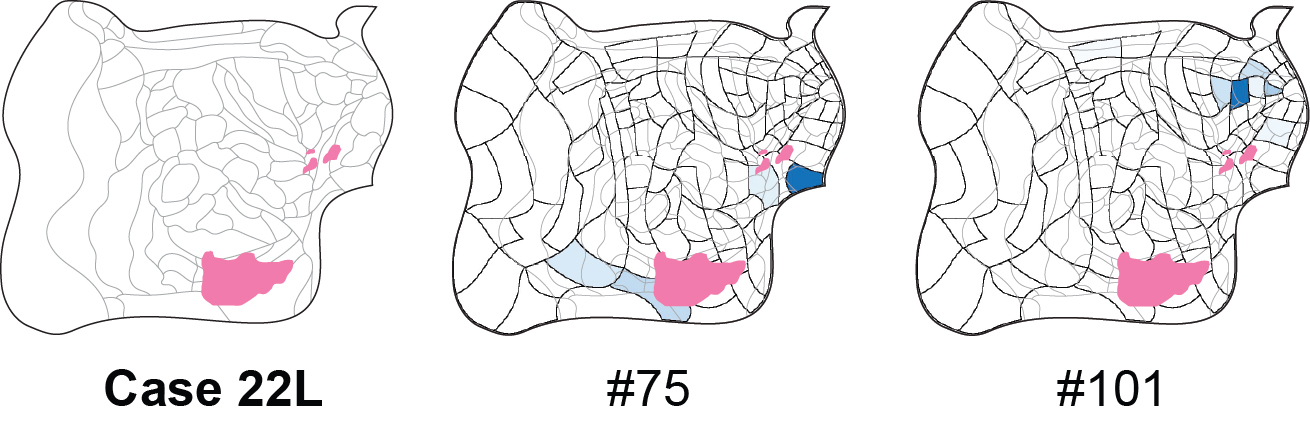

Supplement: Supplement 7 — Supplementary File 2: zip file containing flatmap representations of all matched neurons to (Córdoba-Claros et al., 2025b) [file media-7.zip › Supplementary File 2/case22Lrandom.jpg]

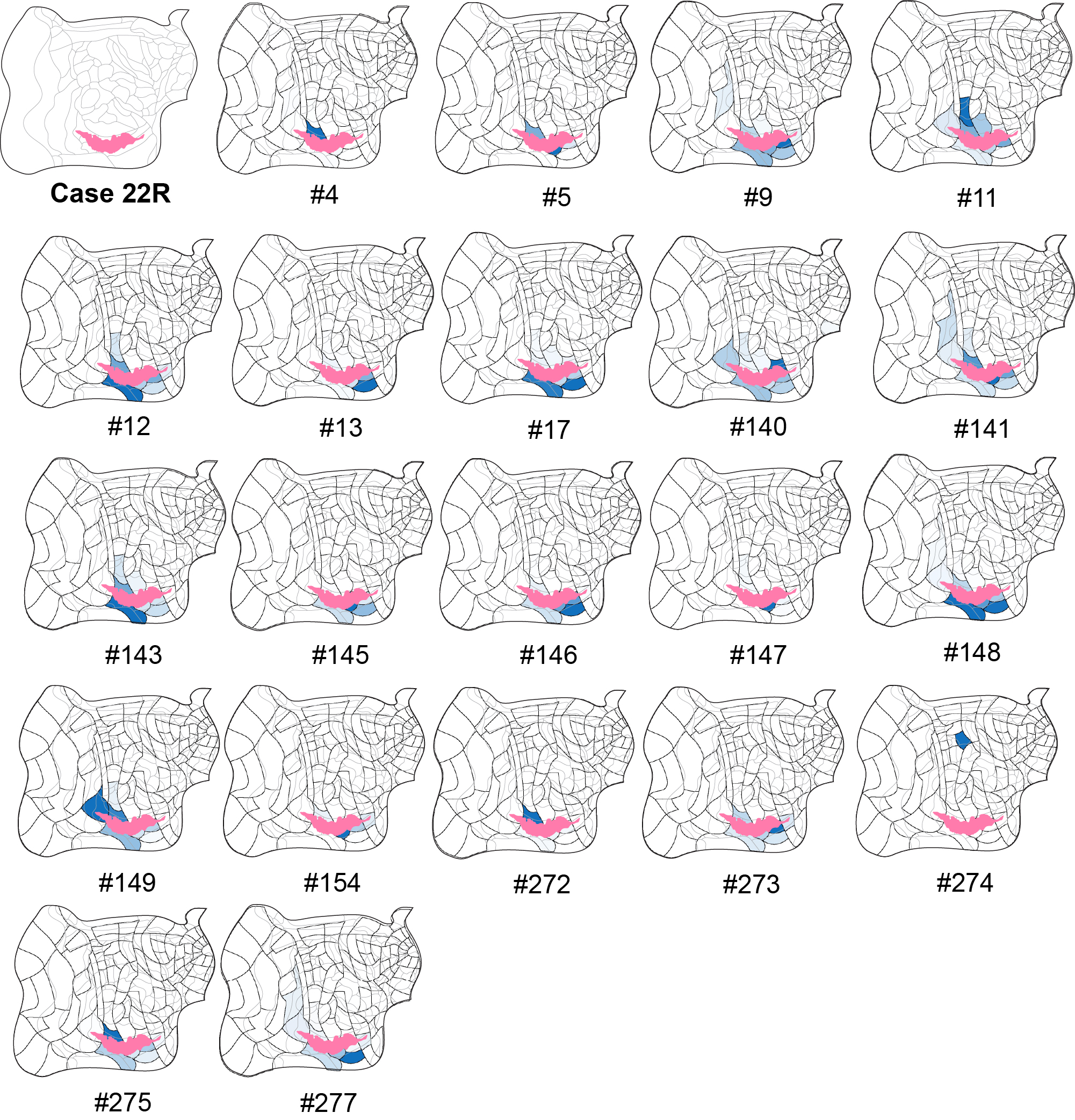

Supplement: Supplement 7 — Supplementary File 2: zip file containing flatmap representations of all matched neurons to (Córdoba-Claros et al., 2025b) [file media-7.zip › Supplementary File 2/case22R.jpg]

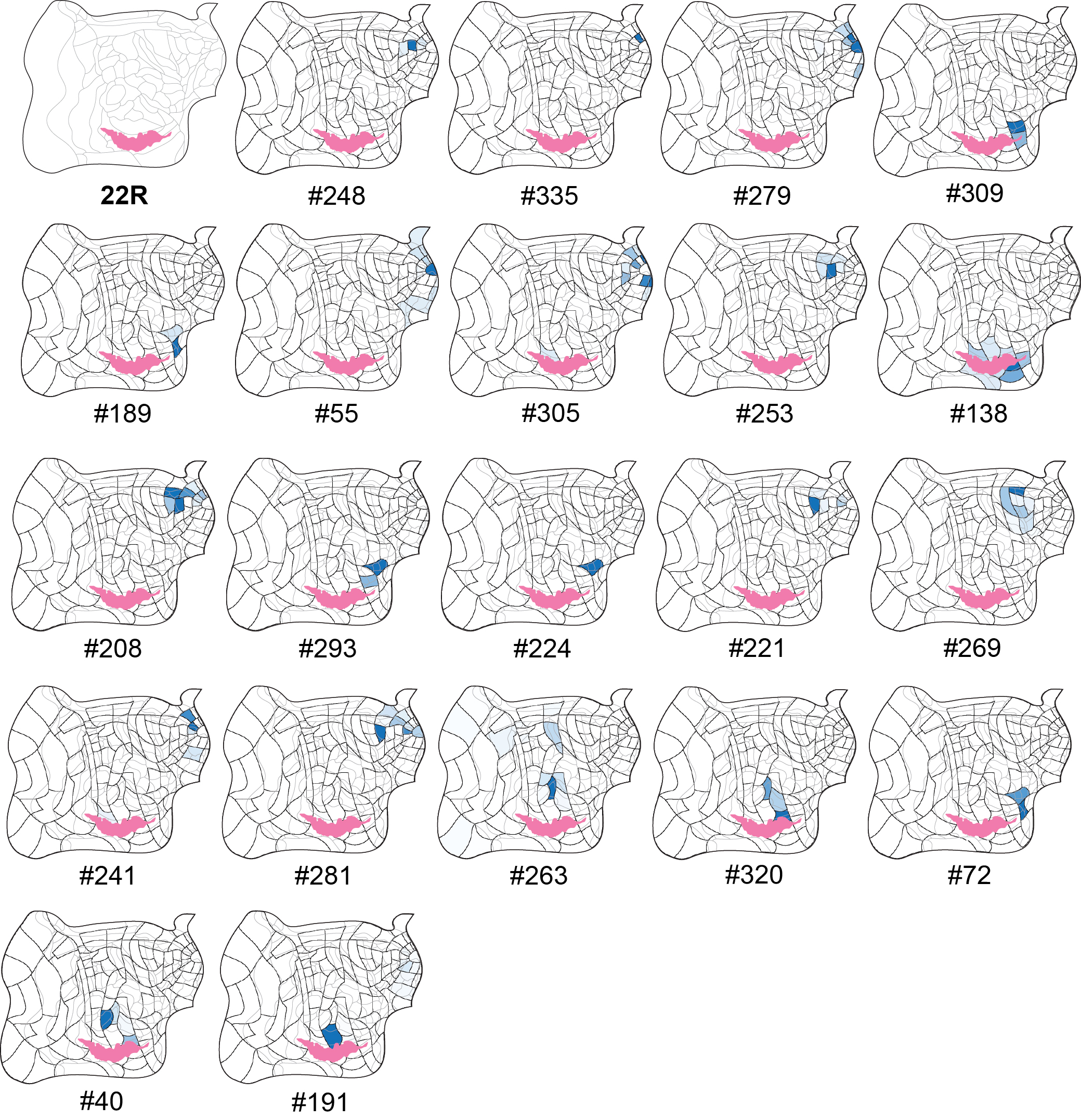

Supplement: Supplement 7 — Supplementary File 2: zip file containing flatmap representations of all matched neurons to (Córdoba-Claros et al., 2025b) [file media-7.zip › Supplementary File 2/case22Rrandom.jpg]

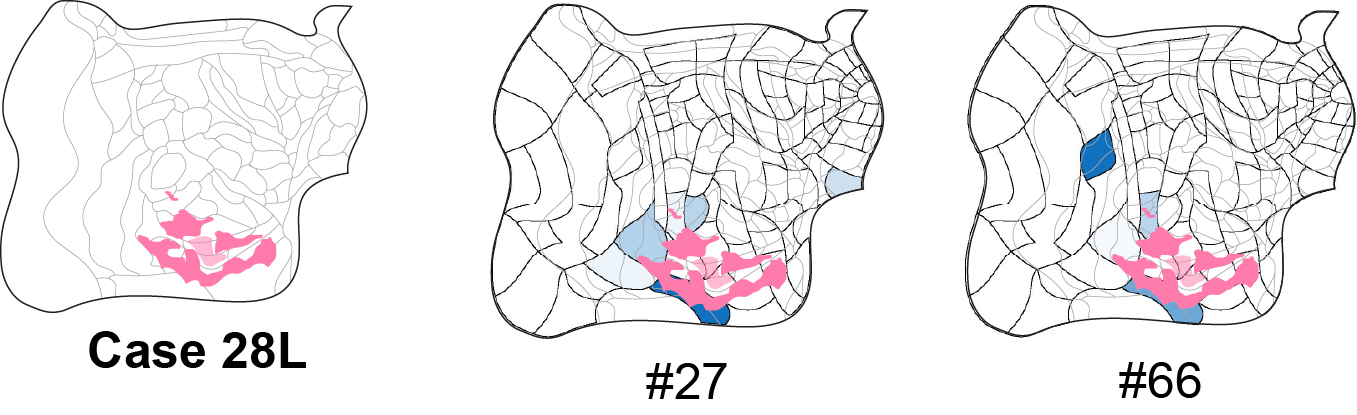

Supplement: Supplement 7 — Supplementary File 2: zip file containing flatmap representations of all matched neurons to (Córdoba-Claros et al., 2025b) [file media-7.zip › Supplementary File 2/case28L.jpg]

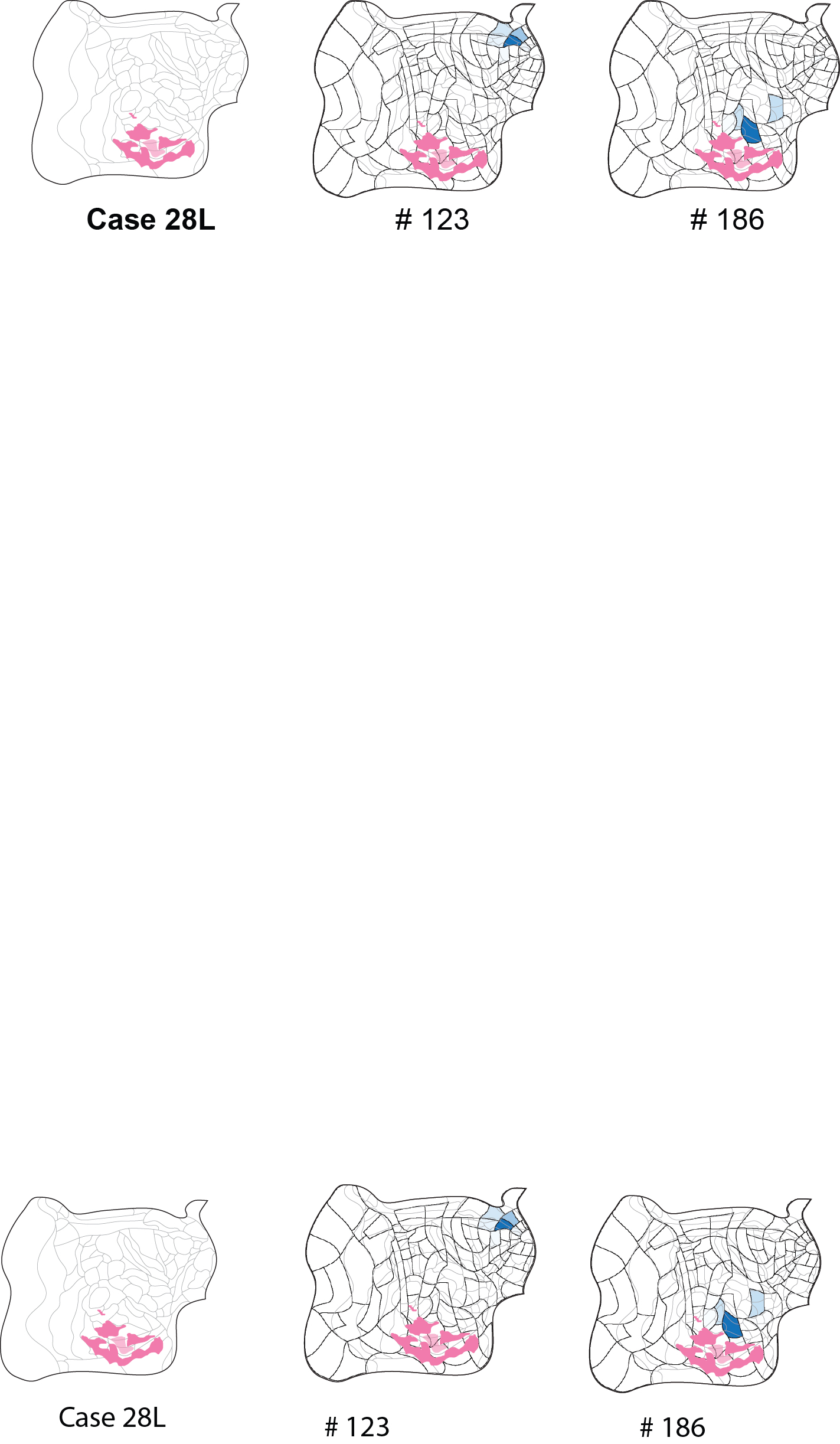

Supplement: Supplement 7 — Supplementary File 2: zip file containing flatmap representations of all matched neurons to (Córdoba-Claros et al., 2025b) [file media-7.zip › Supplementary File 2/case28L_random.jpg]
