# Supplementary material for: Conserved and specialized features of thalamocortical wiring revealed by single-cell projection mapping in mouse and marmoset
Source: bioRxiv. 2026 Jul 8:2026.07.07.736957. Preprint. [Version 1] doi: 10.64898/2026.07.07.736957 (PMC13371098; doi:10.64898/2026.07.07.736957)
Supplement: 8 [file NIHPP2026.07.07.736957v1-supplement-8.pdf]

bioRxiv preprint doi: https://doi.org/10.64898/2026.07.07.736957; this version posted July 8, 2026. The copyright holder for this preprint (which was not certified by peer review) is the author/funder, who has granted bioRxiv a license to display the preprint in perpetuity. It is made available under a CC-BY 4.0 International license.

Supplementary notes

Supplementary Note 1: Gene panel selection and validation

Because BARseq-style in situ sequencing, like other imaging-based spatial transcriptomic approaches, can interrogate a targeted panel of genes, we first selected genes to best capture gene expression variation in the marmoset thalamus based on two single-nucleus RNA sequencing (snRNA-seq) datasets (Dan et al., 2025; Krienen et al., 2023). We aimed to capture variation across the whole thalamus, with particular focus on resolving the transcriptomic heterogeneity of two nuclei: the pulvinar (Pul), a major higher-order sensory thalamic nucleus, and the mediodorsal nucleus (MD), a hub connecting sensory streams to the PFC. Because previous reports have shown that thalamic neurons contain both distinct transcriptomic clusters (Dan et al., 2025; Krienen et al., 2023; Z. Yao et al., 2023) and transcriptomic gradients (Huang et al., 2026; J. M. Phillips et al., 2019), we applied three complementary approaches **(ED Fig. 1a)** to capture both discrete and continuous variation.

We first used MetaMarkers (Fischer and Gillis, 2021) to identify cell type marker genes that are highly expressed and differentially expression either across coarse-level excitatory neuron types in the whole thalamus or across fine-grained cell types in MD and Pul. We subset the snRNA-seq dataset to the thalamic excitatory neurons by identifying clusters that highly express genes characteristic of thalamic excitatory neurons, such as *SLC17A6* (thalamic excitatory neuron marker), *PTPN3*, *KITLG*, *NTNG1*, *OTX2*, *MYO1B*, and *LEF1* (Kita et al., 2021; Shimogori et al., 2018). We identified clusters that are specifically enriched in the MD and Pul using a published Xenium spatial transcriptomic dataset that had been mapped to the aforementioned snRNA-seq dataset. This dataset used a gene panel that was optimized for the basal ganglia, not for the thalamus, but nonetheless distinguished cells across broad thalamic divisions. Because thalamic gene expression also vary continuously (Huang et al., 2026; J. W. Phillips et al., 2019), we next used PERSIST (Covert et al., 2023) to identify genes that best capture gene expression variation across the thalamus, irrespective of cell-type labels. We also included additional classic anatomical markers and select genes of interest in the thalamus, including CALB1, PVALB, *CACNA1C*, *DRD1*, *DRD2*, *DRD3*, *GRIN3A*, *NEGR1*, *OPMCL*, and *PTPRD*. These three sets of genes were then combined with a gene panel focused on distinguishing marmoset cortical neurons, which were designed using MetaMarkers (Fischer and Gillis, 2021).

To estimate how well the gene panel captured cellular diversity in the thalamus, we built a k-nearest-neighbor-based classifier to predict the cluster label of a neuron in the snRNA-seq dataset using various gene panels, and compared them to the performance that can be achieved by the top 1,000 highly variable genes (see **Methods**). Both specificity and sensitivity improved with more genes in the panel and approached the level achievable using highly variable genes, but plateaued between 143 and 179 genes (**ED Fig. 1b**)

bioRxiv preprint doi: https://doi.org/10.64898/2026.07.07.736957; this version posted July 8, 2026. The copyright holder for this preprint (which was not certified by peer review) is the author/funder, who has granted bioRxiv a license to display the preprint in perpetuity. It is made available under a CC-BY 4.0 International license.

The mouse gene panel is a combination of a 104-gene cortical panel (Chen et al., 2025), combined with a 84-gene thalamic panel, both of which were previously designed and validated.

Supplementary Note 2: Hierarchical clustering of in situ sequencing data

We clustered the neurons at three levels, which we denote as hierarchy 1, 2, and 3 (H1, H2, H3; **ED Fig. 2a**). At a coarse level (H1), clustering identified cell populations that were specific to brain regions, such as the thalamus, the thalamic reticular nucleus, the striatum, the hippocampus, and cortical layers (**ED Fig. 2a-c**). We then combined two H1 types that contained excitatory neurons in the thalamus, midbrain, and hypothalamus, and reclustered them into H2 types.

Clustering at the H2 type level initially resulted in seven clusters (**ED Fig. 2d**), but two clusters were of low-quality cells and were removed from downstream analysis. Neurons in the cluster TH_broad were found in the same locations as TH1 and TH2 type neurons with no obvious enrichment at specific locations (**ED Fig. 2e**), had lower read counts per cell compared to TH1 and TH2 (**ED Fig. 2f**), and highly expressed MBP (**ED Fig. 2g**), an oligodendrocyte marker. These characteristics suggest that TH_broad included segmentation errors in which portions of neurons and oligodendrocytes were segmented as single cells, which was confirmed by manual examination of a subset of TH_broad cells. We thus excluded this cluster from further analysis. Lowqual_HY contained a small group of cells at the ventral edge of the hypothalamus and no cells in the thalamus, and was also excluded from further analyses (**ED Fig. 2h**). Two of the remaining H2 types (TH1, TH2) were confined to the thalamus and enriched in higher-order and first-order nuclei, respectively, whilst the remaining three (ILM, HY_Hb, ILM_VA) included neurons in specific nuclei either close to the midline or in the anterior portion of the thalamus, along with neurons in the midbrain, and the hypothalamus.

We reclustered each H2 type again, resulting in 24 H3 thalamic excitatory types (**Fig. 1f**). At this level, neurons in the three mixed H2 types separated into eight H3 types that included neurons in the intralaminar nuclei (centrolateral, CL and centromedial, CM), the habenula (Hb), anterior dorsal nucleus (AD), parafasicular nuclei (PF), and portions of the medial side of the ventroanterior nucleus (VAm), and 21 H3 types that were mostly in the hypothalamus and the midbrain; these non-thalamic H3 types were excluded from further analyses.

Supplementary Note 3: Transcriptomic feature-based area demarcation

Generally, we used the mRNA expression patterns of classic anatomical markers, such as *CALB1* and *PVALB*, combined with neuron type distribution, to draw anatomical borders. Here we present detailed examples on the area demarcation in the marmoset in situ sequencing dataset (**Fig. 1** and **Fig. 2**). We first illustrate the rationale for demarcation on three representative slices

bioRxiv preprint doi: https://doi.org/10.64898/2026.07.07.736957; this version posted July 8, 2026. The copyright holder for this preprint (which was not certified by peer review) is the author/funder, who has granted bioRxiv a license to display the preprint in perpetuity. It is made available under a CC-BY 4.0 International license.

in detail (**ED Fig. 3a-c**), then show the demarcation on all slices (**ED Fig. 3d**). The same approach was also applied to demarcate the marmoset BARseq dataset (**Fig. 4**)

We first used major landmarks, such as the emergence and disappearance of major thalamic nuclei, to match slices to coronal planes. We matched BARseq slice 19 to AP coordinates +2.3 in the marmoset atlas (Paxinos et al., 2026) (**ED Fig. 3a**), based on the shape of the pulvinar, which was *CALB1* positive, the emergence of the MG, and its size relative to the pulvinar. The borders of the medial division of the inferior pulvinar (IPulm) was denoted by a lack of *CALB1* expression within the pulvinar (Paxinos et al., 2026). CA1 of the hippocampus showed a thick band of *CALB1* signals in its pyramidal cell layer (Py), and the dentate gyrus was characterized by a thin ribbon of *CALB1* signal in the granular cell layer (GrDG).

We matched BARseq slices 43 to AP coordinates +3.5 (**ED Fig. 3b**). *PVALB* revealed the LGN. In addition, a thin ribbon of *PVALB*-negative cells marked the LGN's koniocellular layer 3 (K3). K3 is the thickest koniocellular layer at this coronal section and separates the LGN magnocellular and parvocellular layers. At this coronal section, the brachium, a white matter bundle, passed through the pulvinar and connected to the dorsal LGN. Both the posterior limitans (PLi) and the thalamic reticular nucleus (TRN) were positive for *PVALB*, marking the medial-ventral and dorsal-lateral borders of the thalamic nuclei at this AP location. The medial portion of this slice was demarcated with the distinct localization of H3 types. For example, HY_Hb_1, HY_Hb_2, HY_Hb_3 were found in the medial habenula (mHb), and clusters ILM_1 and ILM_2 demarcated the centrolateral nucleus (CL) and intralaminar nuclei, which encased the MD and indicated the posterior end of the MD nucleus.

BARseq slice 91 was matched to an AP location of +5.5 (**ED Fig. 3c**). We used *CALB1* to identify the location of the lateral dorsal nucleus (LD) and the substantia nigra (SNr), both of which were *CALB1* positive. The remainder of the thalamus showed a relatively homogenous expression of *CALB1*, so we used neuron types to demarcate notable anatomical nuclei. Again, ILM_1 and ILM_2 revealed the location of the CL, but also the centromedial (CMn) and posterior parvoventricular nucleus (PVP) (**ED Fig. 3c-i**). The CMn could be demarcated by its own cell type (**ED Fig. 3c-ii**). The MD was encased by the CL/CMn nuclei, and the 3 subdivisions (MDm, MDc, MDl) could be differentiated by differential enrichment of TH1 subtypes (**ED Fig. 3c-iii**). Because the remaining nuclei and subdivisions were less distinct, we did not demarcate their borders.

This same approach was used to demarcate all hemi-coronal sections from the marmoset in-situ sequencing dataset (**ED Fig. 3d**) and the marmoset BARseq dataset. The mouse ABC atlas was pre-registered to the Allen Common Coordinate Framework v3 (CCFv3)(Wang et al., 2020), which allowed us to directly use the CCF labels.

bioRxiv preprint doi: https://doi.org/10.64898/2026.07.07.736957; this version posted July 8, 2026. The copyright holder for this preprint (which was not certified by peer review) is the author/funder, who has granted bioRxiv a license to display the preprint in perpetuity. It is made available under a CC-BY 4.0 International license.

Supplementary Note 4: BARseq in mouse thalamus

Although we performed most of the gene expression analysis using cluster-agnostic approaches, we initially clustered the neurons hierarchically to examine the quality of the data. We mapped the clusters to cell types in reference snRNA-seq datasets (Z. Yao et al., 2023) using a kNN-based approach, and found that each mouse BARseq neuron type corresponded to one or a small number of reference clusters (**ED Fig. 5a**), indicating that this dataset recapitulated the diversity in gene expression in the reference snRNA-seq dataset. These clusters were then used to facilitate delineation of thalamic nuclei (**ED Fig. 5b**).

Because the projection sites were dissected from 300 µm thick sections, whereas in situ sequencing requires 20 µm sections, we developed an approach to allow cryosectioning the thalamus and the cortex on the same coronal planes at different thickness (**ED Fig. 6a**). We first cut 200 µm cryo-sections from both anterior and posterior ends of the brain until we saw the thalamus (Coronal level 67 for the anterior end of the thalamus, 78 for the posterior end). We punched out the thalamus with a 3 mm biopsy punch while it is still frozen, and continued to section the remainder of the brain to 200 µm sections. We sectioned the punched-out thalamus to 20 µm for in situ sequencing. We dissected all areas that receive projections from areas around the LP, based on the Allen Connectivity Atlas, into 17 cubelets (Harris et al., 2019). These include the Superior colliculus/periaqueductal gray (SC/PAG), 15 cortical regions, and the main olfactory bulb (MOB) as a negative control. All images of slices with the dissection areas marked out are provided in **Supplementary File 1**. The block-face image of each section was first matched to coronal planes in the Allen Reference Atlas based on major landmarks. We drew the dissection areas onto each matched CCF coronal plane, then transformed them into cortical flatmaps (**ED Fig. 6a**)

We first applied a linguistic complexity threshold to filter out soma barcodes with repetitive sequences (**ED Fig. 6b**). These “barcodes” were usually background fluorescence instead of true barcodes. To match the filtered soma barcodes to those in the projection sites, we first examined the distribution of minimum hamming distance between a soma barcode and projection barcodes, and compared the distribution to randomly shuffled soma barcodes (**ED Fig. 6c**). We found that a sizeable fraction of soma barcodes were perfect matches to projection barcodes (i.e., had minimal hamming distance of 0). In contrast, few shuffled barcodes were perfect matches (0.18% ± 0.04%, mean ± standard deviation across 200 shuffled barcode sets), indicating that the soma barcodes did not match by chance.

To exclude the possibility that barcodes in passing axons, dendrites, and/or nearby somas were assigned to a soma by mistake, we manually examined the sequencing images for each barcoded cell to identify 1,578 barcodes in 1,809 high confidence barcoded somas. Of these somas, 60 barcodes were found in multiple cells, 13 barcodes were found in the same cells but were imaged multiple times, because they were in the overlapping areas between adjacent imaging tiles. We filtered out barcodes that were found in multiple cells, and removed the “extra” cells that were

bioRxiv preprint doi: https://doi.org/10.64898/2026.07.07.736957; this version posted July 8, 2026. The copyright holder for this preprint (which was not certified by peer review) is the author/funder, who has granted bioRxiv a license to display the preprint in perpetuity. It is made available under a CC-BY 4.0 International license.

imaged multiple times, leaving 1,518 cells with unique barcodes for downstream analysis. The observed barcode collision rate (i.e., the same barcode labelling multiple cells) was 60 / 1578 = 4%, which was consistent with previous studies (Chen et al., 2019; Sun et al., 2021). Because all of the detected double labelled barcodes have been removed from the filtered data, we consider the observed rate as an upper bound in the filtered data, and the effect on the downstream analyses was minimal due to the filtering. No barcode was found in the negative control site (MOB), or found in the brain other than the one that the soma was found in, indicating that the barcodes detected in the projection sites were specific.

Supplementary Note 5: Non-negative matrix factorization of projection data in both species

Individual mouse neurons projected to 4.1 ± 2.5 target areas (mean ± standard deviation) (**ED Fig. 7f**), and the dominant target accounted for 61% ± 23% (mean ± standard deviation) of barcode molecules per neuron (**ED Fig. 7g**). Furthermore, certain areas were often co-innervated by the same neurons (**ED Fig. 7h**), suggesting that projection data may lie in a lower-dimensional space than the number of cubelets. This is typical of biological measurements in the nervous system, such as gene expression and neuronal activity, where the measured variables are far more numerous than the latent biological processes that generate them. Dimensionality reduction is a natural step for analyzing such data, as it can recover this underlying structure while suppressing noise. Recovering a shared low-dimensional structure also enables more accurate cross-species comparison, since the dissection resolutions of mouse and marmoset differed substantially (17 vs. 94 cubelets) and were not directly comparable in the original cubelet space. We thus applied NMF to group cubelets that were frequently co-innervated into target domains. In both mouse and marmoset datasets, we selected the number of domains based on how well they reflect prior biological knowledge. In mouse, we chose a value that resolved the major functional modalities of the mouse cortex (**ED Fig. 7i**). These included motor cortex (MOs), the auditory and temporal association areas (TE), the frontal cortex (PL/IL), and anterior cingulate cortex (ACA). Given the spatial extent of visual-associated cortex, visual areas were subdivided into three target domains: VIS-caudal (VIS-c), which encompasses retrosplenial cortex and constitutes the most posterior cortical domain; VIS-rostral (VIS-r), which contained primary visual cortex; and a third domain encompassing VIS-anteromedial and adjacent SSp cubelets (VIS-am/SSp). The superior colliculus and periaqueductal grey, dissected as a unit, formed an additional target domain.

Because the marmoset cortex was dissected at sub-area resolution, we selected the lowest number of target domains that resolved the full complement of cortical targets innervated by barcoded neurons across the pulvinar subdivisions and neighboring nuclei (**ED Fig. 10b**). Because we could not match the left and right hemisphere cubelet-to-cubelet, we performed NMF separately for the two hemispheres, and combined the data afterwards. In the right hemisphere, we identified three target domains in the temporal cortex, four in the prefrontal

bioRxiv preprint doi: https://doi.org/10.64898/2026.07.07.736957; this version posted July 8, 2026. The copyright holder for this preprint (which was not certified by peer review) is the author/funder, who has granted bioRxiv a license to display the preprint in perpetuity. It is made available under a CC-BY 4.0 International license.

cortex, and one in the motor/premotor area. Of the three domains in the temporal cortex, two of them corresponded to the dorsal visual stream (TE-dorsal, including MT, FST, MST) and the ventral visual stream (TE-vent, including V4, TEO, TE), and a third domain that was more anterior to them (TE-ant, including STR and anterior TE areas). The dlPFC was further subdivided into two domains: a posterior domain (dlPFCp) encompassing the frontal eye fields (8Ad and 8Av), and an anterior domain (dlPFCa) encompassing areas 46d and 46v. Two medial prefrontal domains were also identified: a dmPFCa domain containing area 9, and a broader mPFC domain encompassing anterior cingulate (area 32) and medial orbital prefrontal cortex (areas 13/14). Motor and premotor cortex, consistent with their known connectivity with the posterior nucleus (PO), formed a separate domain. In the left hemisphere, we obtained eight domains: dlPFCa, TE-vent, TE-dorsal, TE-ant1, TE-ant2, IP-motor, V1, and vlPFC. The first three overlapped with those in the right hemisphere; TE-ant1 and TE-ant2 corresponded to TE-ant in the right hemisphere; IP-motor corresponded to Motor in the right hemisphere, but included additional intraparietal areas. The differences in the domains between the two hemispheres likely reflected differences in labelling, which captured neurons that projected to different cortical areas. For analysis purposes, we typically pooled the two hemispheres together by combining corresponding target domains. For analysis involving gene expression, we used the right hemisphere only, because the majority of the left hemisphere were sequenced with a smaller gene panel that did not fully resolve the gene-projection relationship.

Supplementary Note 6: BARseq reveals MD – PFC projection topography in the marmoset

To validate BARseq in the marmoset, we mapped the MD projections to the PFC because of its well-documented topography in primates. We used an MRI-guided surgery robot to inject a barcoded pseudotyped Sindbis bilaterally into the MD nucleus of a pilot animal (**ED Fig. 8a**, **Methods**). We injected a variant of the Sindbis virus that was pseudotyped with the structural protein derived from the Eastern Equine Encephalitis virus (EEEV). This pseudotyped Sindbis virus has been previously optimized for barcoded projection mapping in primate brains (J Kebschull, unpublished data).

After tissue collection 48 hours post-injection, we used a vibrating microtome to section the frontal cortex of the animals into 400 μm slabs. These were frozen on microscope slides, and images were matched to the Stereotaxic atlas of the marmoset brain to demarcate dissection locations.

We sequenced the injection site for both barcodes and a small gene panel (**Supp. Table 2**). This panel was not sufficient to resolve finer differences in thalamic neurons, but was useful to parcellate thalamic nuclei. To consolidate the frontal cortical dissections with published literature, we aggregated cubelets into six regions of the PFC; Area 10 (most anterior portion of the frontal cortex), dlPFC (Area 46D, 46V, 8aV, 8aD), dmPFC (Area 9), mPFC (Area 32), oPFC (Area 13,

bioRxiv preprint doi: https://doi.org/10.64898/2026.07.07.736957; this version posted July 8, 2026. The copyright holder for this preprint (which was not certified by peer review) is the author/funder, who has granted bioRxiv a license to display the preprint in perpetuity. It is made available under a CC-BY 4.0 International license.

14, 11), and vlPFC (Area 45, 47). Projections to the frontal cortex can cover multiple contiguous regions, but usually one region had the most dominant projection (**ED Fig. 8d**). We thus categorized neurons based on their strongest projections.

Our data revealed projection differences across all three spatial axes across the MD. In the right hemisphere, where the injection was slightly more posterior, most neurons projected to the dlPFC. This dominance of dlPFC projections from the posterior end of the MD was consistent with previous imaging studies in the macaque (Saalmann et al., 2012). In the left hemisphere, where the labelled neurons were more anterior, neurons projecting to vlPFC, dlPFC, mPFC, and oPFC occupied ventral, lateral, dorsal, and medial quadrants, respectively (**Fig. 4c**). This circular topography was consistent with that described in a previous marmoset retrograde tracing study (Roberts et al., 2007). Thus, BARseq data accurately recapitulated the previously described topography of marmoset MD-PFC connectivity.

Supplementary Note 7: Marmoset BARseq validation

To compare the marmoset projection data to existing bulk-tracing data quantitatively, we identified neurons in our data with somas that overlapped with injection sites in bulk-tracing data, then assessed overlap in the projections. To match somas in our data to bulk-tracing injections, we identified the AP locations of the centers of published injection sites, found the sections that corresponded to the same AP locations in our data, then re-traced the injection site boundary on our sections. We then identified BARseq neurons within that boundary, extended 100 µm in both the anterior and posterior direction. Because each injection site spanned about 500 µm – 800 µm within the coronal section, we expected that the real injection site would span a similar amount on the AP axis. Thus, our estimate of the injection site was conservative.

Because the published flatmap representation of the bulk tracing (Córdoba-Claros et al., 2025b) differed from the MRI-based surface projection used in our data, we needed to transform the two datasets into a common space for comparison. We manually retraced all bulk projection zones in the published dataset onto our flatmap template based on annotations in the Paxinos atlas. For each BARseq neuron, we then identified how many "targets" it projected to; each projection target was defined as a continuous zone of barcodes in dissection cubelets with a single local maximum. For each projection target in each neuron, we counted how many cubelets away it was to the closest area labelled by the matching bulk tracing. For neurons that projected to multiple targets, we used the mean of all targets (**Supp. Table 5**; **Supp. File 2**).
